# ER quality control of Crumbs2 and Notch proteins by GXYLT1-dependent xylosyl elongation of *O*-glucose glycans

**DOI:** 10.1016/j.jbc.2026.113556

**Published:** 2026-09-15

**Authors:** Yukihiro Mita, Soomin Cho, Yohei Tsukamoto, Wataru Saiki, Mio Okamura, Yuki Fujita, Nima Niknejad, Daichi Funada, Fuga Suzuki, Kensuke Ohkawa, Taiki Jo, Yusuke Urata, Yuuki Kurebayashi, Akira Minami, Tadanobu Takahashi, Hideharu Hibi, Tetsuya Okajima, Hamed Jafar-Nejad, Hideyuki Takeuchi

**Affiliations:** 1 Department of Molecular and Cellular Biology, Nagoya University Graduate School of Medicine, Nagoya, Japan; 2 Department of Oral and Maxillofacial Surgery, Nagoya University Graduate School of Medicine, Nagoya, Japan; 3 Department of Molecular & Human Genetics, Baylor College of Medicine, Houston, Texas, USA; 4 Development, Disease Models & Therapeutics Graduate Program, Baylor College of Medicine, Houston, Texas, USA; 5 Department of Biochemistry, School of Pharmaceutical Sciences, University of Shizuoka, Shizuoka, Japan; 6 Department of Functional Morphology, Faculty of Pharmacy, Juntendo University, Chiba, Japan; 7 Institute for Glyco-core Research (iGCORE), Nagoya University, Nagoya, Japan

**Keywords:** EGF repeats, endoplasmic reticulum, ER quality control, glycosylation, glycosyltransferase, mass spectrometry, crumbs, Notch, *O*-glucosylation, posttranslational modification, xylose

## Abstract

Protein *O*-glucosyltransferase 1 (POGLUT1/Rumi) transfers an *O*-linked glucose (*O*-Glc) monosaccharide from UDP-Glc to a serine residue in epidermal growth factor (EGF)-like repeats with a specific consensus sequence. In mammals, GXYLT1 and GXYLT2 transfer xylose to this *O*-Glc monosaccharide. Previous studies showed that *Poglut1* deletion in mice leads to embryonic lethality with defects in crumbs family member 2 (CRB2) trafficking and NOTCH1 signaling. Both CRB2 and Notch receptors have multiple EGF repeats with the *O*-Glc modification site. However, the roles of xylosyl elongation of *O*-Glc in protein trafficking and mammalian embryonic development are unknown. Here, we demonstrated that xylosyl elongation of *O*-Glc occurs in the endoplasmic reticulum and comprehensively analyzed *O*-Glc glycosylation on CRB2, NOTCH1, and NOTCH2 expressed in HEK293T cells by mass spectrometry. Most EGF repeats containing the consensus sequence were modified with *O*-Glc glycans, while xylosyl elongation varied among different EGF repeats, suggesting EGF repeat-specific regulation. *GXYLT1* KO cells showed reduced or lost xylosyl elongation, but *GXYLT2* KO cells did not. Cell-based assays demonstrated decreased secretion of substrate proteins in *GXYLT1* KO cells, which was rescued by WT GXYLT1. Analysis of *Gxylt1* and *Gxylt2* KO mice revealed that *Gxylt1* deletion, but not *Gxylt2* deletion, caused embryonic lethality. Altogether, our data suggest that GXYLT1 plays a major role in xylosyl elongation of *O*-Glc glycans and thereby contributes to the endoplasmic reticulum quality control and trafficking of CRB2, NOTCH1, and NOTCH2 in HEK293T cells while GXYLT1 and GXYLT2 have distinct biological functions during mouse embryonic development.

The endoplasmic reticulum (ER) is an intracellular organelle responsible for various cellular functions, including protein biosynthesis and posttranslational modification, calcium storage and release, lipid metabolism, phospholipid membrane supply, and drug metabolism (1). Proper ER function is vital for cellular homeostasis, and defects are associated with a host of human diseases (2). Protein *N*-glycosylation is a well-established key player in the ER protein quality control (3, 4). The functional significance of *O*-linked glucose (*O*-Glc) glycans, particularly in the quality control of proteins containing EGF repeats, has also been recognized.

*O*-Glc glycans were first identified on epidermal growth factor (EGF)-like repeats of blood coagulation factors and later on NOTCH1 (5, 6). EGF repeats consist of approximately 40 amino acids with six conserved cysteine residues that form three disulfide bonds in a specific pattern, stabilizing the 3D structure of the EGF repeat. *O*-Glc monosaccharide is transferred from UDP-Glc to a serine residue within the *O*-Glc consensus sequence (C^1^-X-S-X-P/A-C^2^) of an EGF repeat by the protein *O*-glucosyltransferase 1 (POGLUT1, Rumi in *Drosophila*) (Fig. 1) (7, 8, 9). Human and mouse gene database searches reveal over 50 extracellular or transmembrane-bound proteins harboring the POGLUT1 consensus sequence, including crumbs family proteins and Notch pathway ligands and receptors (8, 10). Loss of *Poglut1* in mice leads to embryonic lethality, defects in epithelial-to-mesenchymal transition due to loss of cell surface expression of the crumbs family member 2 (CRB2) protein, and *Notch*-like phenotypes (8, 11). Moreover, recessive pathogenic variants in human *POGLUT1* have been reported to cause a novel form of limb-girdle muscular dystrophy called LGMDR21, which is associated with reduced NOTCH1 signaling in patient muscle samples and in myogenic progenitors derived from LGMDR21 patient induced pluripotent stem cells (12, 13, 14). In addition, conditional KO studies in mouse and cell-based signaling assays in the C2C12 myoblast cell line showed that *Poglut1* is required for early postnatal muscle development, establishment and maintenance of the adult muscle stem cells, and signaling mediated by all three Notch receptors expressed in muscle stem/progenitors, that is, NOTCH1, NOTCH2, and NOTCH3 (15). These observations indicate that POGLUT1 plays critical roles in mammalian embryonic development, myogenesis, and muscle maintenance, at least in part by regulating the trafficking of some of its target proteins.

**Figure 1:**
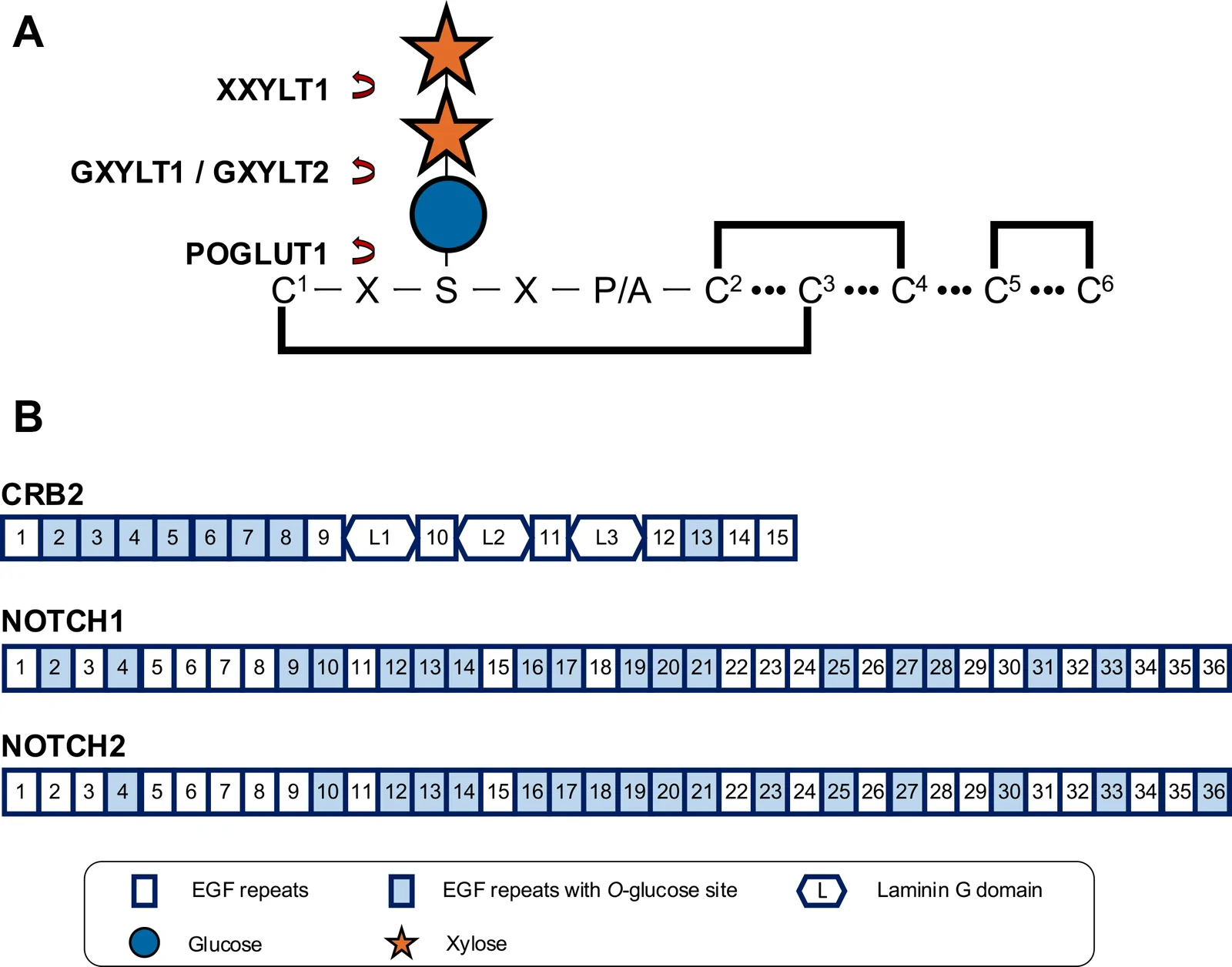
**Schematic presentation of POGLUT1-mediated *O*-glucosylation on EGF repeats in the extracellular domain of CRB2, NOTCH1, and NOTCH2.***A*, the *O*-Glc consensus sequence (C^1^-X-S-X-P/A-C^2^) within a single EGF domain containing six conserved cysteine residues. Specific pairs of cysteine residues forming disulfide bonds are indicated. *Blue circle*, glucose; *orange star*, xylose. *B*, distribution map of EGF repeats with predicted *O*-Glc sites (*blue*) in the extracellular domains of CRB2, NOTCH1, and NOTCH2. The mouse CRB2 extracellular domain comprises 15 EGF repeats (*rectangles*) and three Laminin G domains (*hexagons*). The mouse NOTCH1 and NOTCH2 extracellular domains contain 36 EGF repeats each. CRB2, crumbs family member 2; EGF, epidermal growth factor; POGLUT1, protein O-glucosyltransferase 1.

In mammals, GXYLT1 and GXYLT2 transfer the first xylose to *O*-Glc, while XXYLT1 transfers the second xylose (Fig. 1) (16, 17). POGLUT1 has a KTEL ER retention signal at its C terminus, and the POGLUT1 enzymatic activity is found in the ER fraction (9). When overexpressed in CHO cells, XXYLT1 is localized to the ER (17). Thus, the biosynthesis of *O*-Glc trisaccharides on EGF repeats is assumed to occur in the ER (18). However, whether this is indeed the case has not been formally examined. In *Poglut1* KO mouse embryos, CRB2 accumulates in the ER (11). Moreover, a mutation of the *O*-Glc modification site in EGF6 of human CRB2 causes a defect in its trafficking to the cell surface in HEK293T cells (19). Similarly, a previous study showed that genetic deletion of *POGLUT1* in HEK293T cells reduced the cell surface expression of endogenous NOTCH1 (20), although this effect seems to be cell type-specific, as *Poglut1* knockdown in C2C12 mouse myoblasts did not reduce NOTCH1 cell surface expression despite a severe reduction in NOTCH1 cleavage (8). Genetic deletion of both *GXYLT1* and *GXYLT2* in HEK293T cells reduced the cell surface expression of overexpressed NOTCH1 and NOTCH2, but not endogenous NOTCH1 or NOTCH2 (21). Together, these data suggest that xylosyl elongation might serve as a quality control mechanism in cells expressing high levels of substrate proteins like Notch receptors. However, the impact of GXYLT1 *versus* GXYLT2 on the trafficking of substrate proteins and mouse embryonic development is not known.

In this study, we show that xylosyl elongation of *O*-Glc glycans occurs in the ER by analyzing glycopeptides modified with both *O*-Glc glycans and *N*-glycans by mass spectrometry. Systematic analysis of GXYLT1- and GXYLT2-dependent xylosyl elongation on CRB2, NOTCH1, and NOTCH2 in HEK293T cells indicates differential roles of these two enzymes in EGF repeat xylosylation. Cell-based secretion assays and mass spectrometry indicate a predominant role for GXYLT1 in the proper trafficking of these substrate proteins when overexpressed. Furthermore, genetic deletion of *Gxylt1*, but not *Gxylt2*, in mice caused embryonic lethality. These observations indicate that despite their similar biochemical function, there is a major difference in the roles of GXYLT1 and GXYLT2 in ER protein quality control and mammalian embryonic development.

## Results

### Xylosyl elongation of *O*-Glc occurs in the ER

To date, the subcellular localization of xylosyl elongation of *O*-Glc glycans on EGF repeats remains unclear. Previously, it was reported that overexpressed XXYLT1 is localized to the ER (17). Given that XXYLT1 is responsible for the addition of the distal xylose residue on *O*-Glc, it is presumed that GXYLT1 and GXYLT2 transfer the first xylose to *O*-Glc in the ER as well. To determine if xylosylation occurs in the ER in the endogenous condition, we used mass spectrometry to ask whether an endoglycosidase (endo) H-sensitive *N-*glycopeptide from CRB2 is modified with xylosyl elongated *O*-Glc glycans. We chose CRB2 because POGLUT1-mediated glycosylation of CRB2 is essential for its proper trafficking to the plasma membrane (11, 19). CRB2 possesses a putative *N*-glycosylation site at Asn-239 on EGF5. Therefore, we purified the EGF1-8 portion of mouse CRB2 from both cell lysate and culture medium and examined their sensitivity to digestion by Endo H and PNGase F (Fig. 2*A*). As expected, PNGase F digestion produced a faster migrating form of CRB2 EGF1-8 from both sources, confirming the presence of at least one *N-*glycan. CRB2 EGF1-8 from culture media was resistant to Endo H treatment, suggesting that the *N-*glycan(s) on CRB2 EGF1-8 mature to the complex form through the secretory pathway. On the other hand, CRB2 EGF1-8 derived from cell lysates was sensitive to Endo H-mediated deglycosylation, suggesting that CRB2 EGF1-8 carries *N*-glycan with two terminal mannose residues (22). These data suggest that CRB2 EGF1-8 purified from the cell lysate is ER-localized.

**Figure 2:**
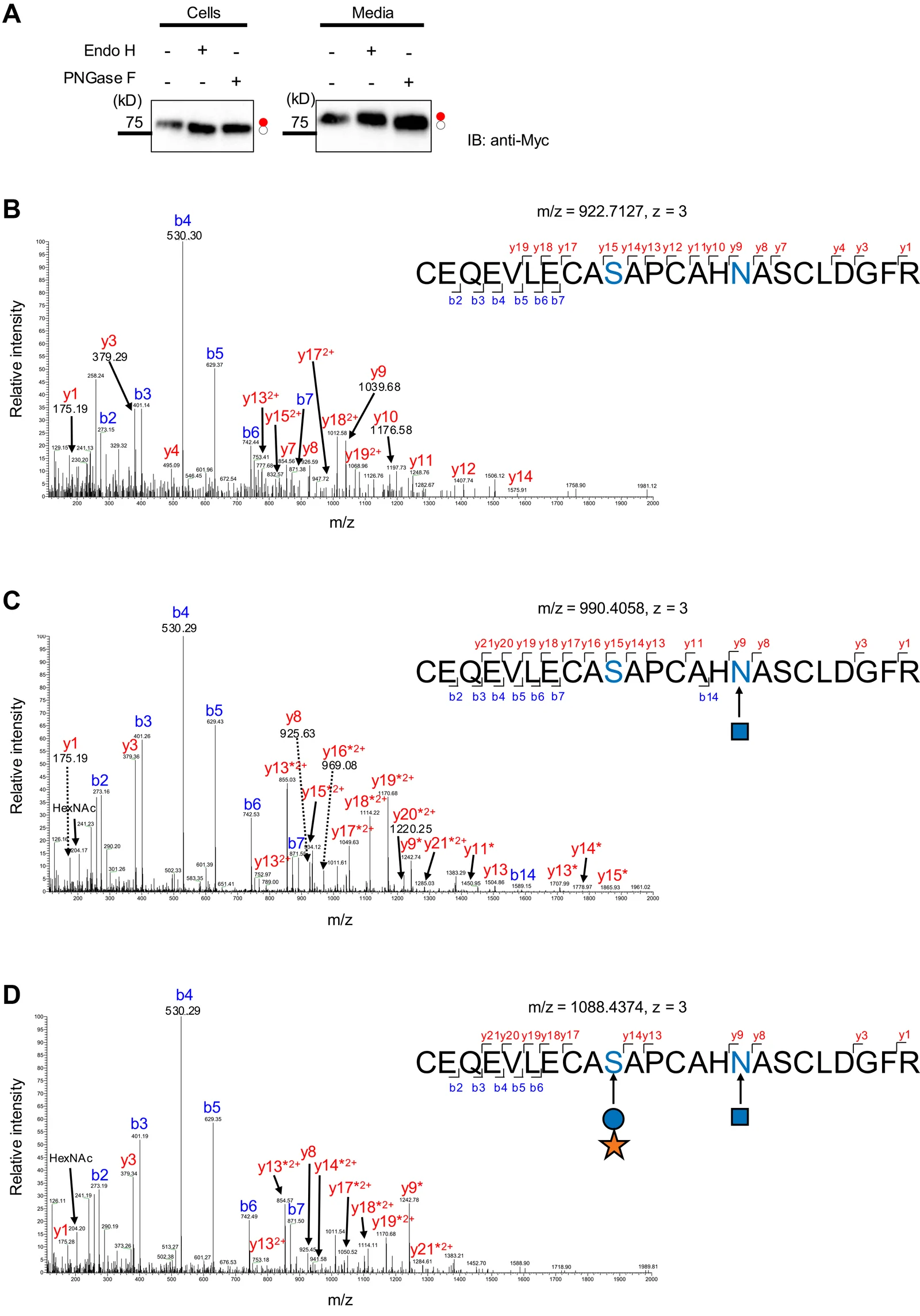
**Xylosyl elongation of *O-*Glc is suggested to occur in the ER.***A*, the C-terminally MycHis-tagged EGF1-8 portion of CRB2 was expressed in HEK293T cells, purified, treated with PNGase F or Endo H, and analyzed by immunoblotting using anti-Myc (9E10) antibody. Glycosylated (*red-filled circle*) and de-glycosylated (*open circle*) bands are indicated. *B*, MS/MS spectrum of a triply charged ion at 922.7127, matching the unmodified 224-CEQEVLECASAPCAHNASCLDGFR-247 peptide from CRB2 EGF5. The b- and y-fragment ions confirm the peptide identity. *C*, MS/MS spectrum of a triply charged ion at 990.4058, matching the HexNAc-modified 224-CEQEVLECASAPCAHNASCLDGFR-247 peptide from CRB2 EGF5. Several b ions, similarly observed in *B*, and the presence of y ions still carrying the HexNAc residue (indicated with an *asterisk*) support the identification of the peptide sequence and the modification at Asn-239. *D*, MS/MS spectrum of a triply charged ion at 1088.4374, matching the 224-CEQEVLECASAPCAHNASCLDGFR-247 peptide modified with the HexNAc, Hex, and Pen residues. The MS/MS fragmentation pattern and the sequential neutral loss from the precursor ion confirm both a HexNAc and a Hex-Pen disaccharide. *Blue circle*, glucose; *blue square*, GlcNAc; *orange star*, xylose. CRB2, crumbs family member 2; EGF, epidermal growth factor; ER, endoplasmic reticulum; MS, mass spectrometry; *O*-Glc, *O*-linked glucose.

Next, we performed tryptic digestion and Endo H-mediated deglycosylation of CRB2 EGF1-8 derived from cells and analyzed the resulting glycopeptides by mass spectrometry to examine whether or not Endo H-sensitive glycopeptides are xylosylated. We searched for the specific glycopeptide corresponding to the first half of EGF5 of CRB2, 224-CEQEVLECASAPCAHNASCLDGFR-247, because this peptide carries the consensus sequences for both *N-*glycosylation and POGLUT1-mediated *O-*glucosylation (underlined N and S, respectively). Numerous b- and y-ions resulting from the high-energy collision-induced fragmentation confirmed that the triply charged ion (m/z: 922.7127) is derived from this peptide without any glycan modifications (Figs. 2*B* and S1, Table S1). The fragmentation patterns led to the identification of another ion (m/z: 990.4058) derived from the same peptide (Figs. 2*C* and S1, Table S1). The mass difference of 67.6931 between these ions matches the triply charged mass of HexNAc (67.6931, monoisotopic mass). Several fragment ions corresponding to y ions (y10-y22) that carry the HexNAc modification strongly suggest that HexNAc modification occurs at Asn-239, supporting the conclusion that an *N-*linked GlcNAc is attached to this peptide. Following these observations, we successfully identified several ions derived from glycopeptides which are modified with both *N*-GlcNAc and *O-*Glc glycan (Glc, Xyl-Glc, and Xyl-Xyl-Glc), which is evident upon the neutral loss of hexose and pentose from precursor ions (Figs. 2*D* and S1, Table S1). These results strongly suggest that *O-*glucosylation and subsequent xylosyl elongation on EGF repeats occur while *N-*glycans remain in their immature form, namely, while in the ER.

To further examine whether xylosyl elongation of *O*-Glc occurs in the ER, we analyzed the intracellular localization of CRB2 EGF1-8 by immunofluorescence staining. CRB2 EGF1-8 showed substantial colocalization with the ER marker calnexin but little overlap with the *cis*-Golgi marker GM130 (Fig. 3, *A* and *B*), indicating that intracellular CRB2 EGF1-8 was predominantly localized in the ER. Moreover, we analyzed CRB2 EGF1-8 immunopurified from HEK293T cell lysates by mass spectrometry and detected the EGF6 glycopeptide carrying *O*-Glc extended with two xylose residues (Fig. 3*C*).

**Figure 3:**
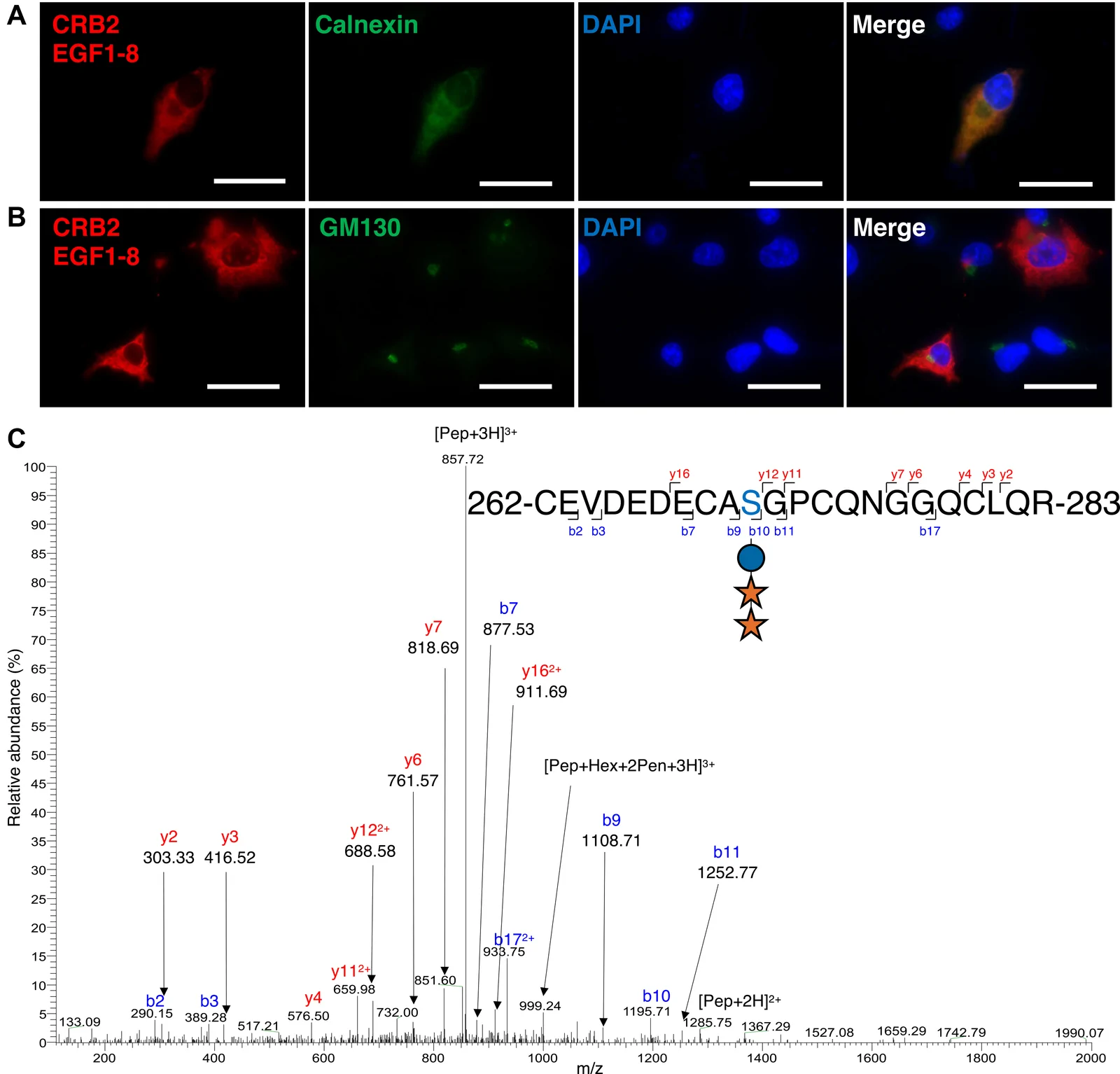
**ER localization and xylosyl elongation of *O*-Glc on CRB2 EGF repeats.***A* and *B*, the C-terminally MycHis-tagged EGF1-8 portion of CRB2 was expressed in COS-7 cells and analyzed by immunofluorescence staining. CRB2 EGF1-8 was detected using an anti-Myc antibody (*red*). Calnexin (*A*) and GM130 (*B*) were used as markers for the ER and *cis*-Golgi, respectively (*green*). Nuclei were stained with DAPI (*blue*). Merged images are shown in the *right panels*. The scale bars represent 50 μm. *C*, the C-terminally MycHis-tagged EGF1-8 portion of CRB2 was expressed in HEK293T cells. CRB2 EGF1-8 purified from the cell lysates was digested with trypsin and analyzed by mass spectrometry. MS/MS spectrum of a triply charged ion at 999.3788, matching the peptide 262-CEVDEDECASGPCQNGGQCLQR-283 from CRB2 EGF6 modified with one Hex and two Pen residues. The b- and y-fragment ions confirm the peptide identity, and the MS/MS fragmentation pattern and sequential neutral loss from the precursor ion support the presence of a Xyl-Xyl-Glc trisaccharide. *Blue circle*, glucose; *orange star*, xylose. CRB2, crumbs family member 2; EGF, epidermal growth factor; ER, endoplasmic reticulum; *O*-Glc, *O*-linked glucose.

We next examined the subcellular localization of GXYLT1 and GXYLT2, the glycosyltransferases responsible for the first xylose addition to *O*-Glc, by immunofluorescence staining. Full-length human GXYLT1 and GXYLT2 were transiently expressed in COS-7 cells and compared with the ER marker calnexin and the *cis*-Golgi marker GM130. GXYLT1 showed substantial colocalization with calnexin but little overlap with GM130, indicating its predominant localization in the ER (Fig. 4, *A* and *B*). In contrast, GXYLT2 showed substantial colocalization with GM130 and little overlap with calnexin, indicating its predominant localization in the *cis*-Golgi (Fig. 4, *C* and *D*). Taken together, the predominant ER localization of intracellular CRB2 EGF1-8, the presence of xylosyl-elongated *O*-Glc on intracellular CRB2 EGF1-8, and the ER localization of GXYLT1 demonstrate that xylosyl elongation of *O*-Glc occurs in the ER.

**Figure 4:**
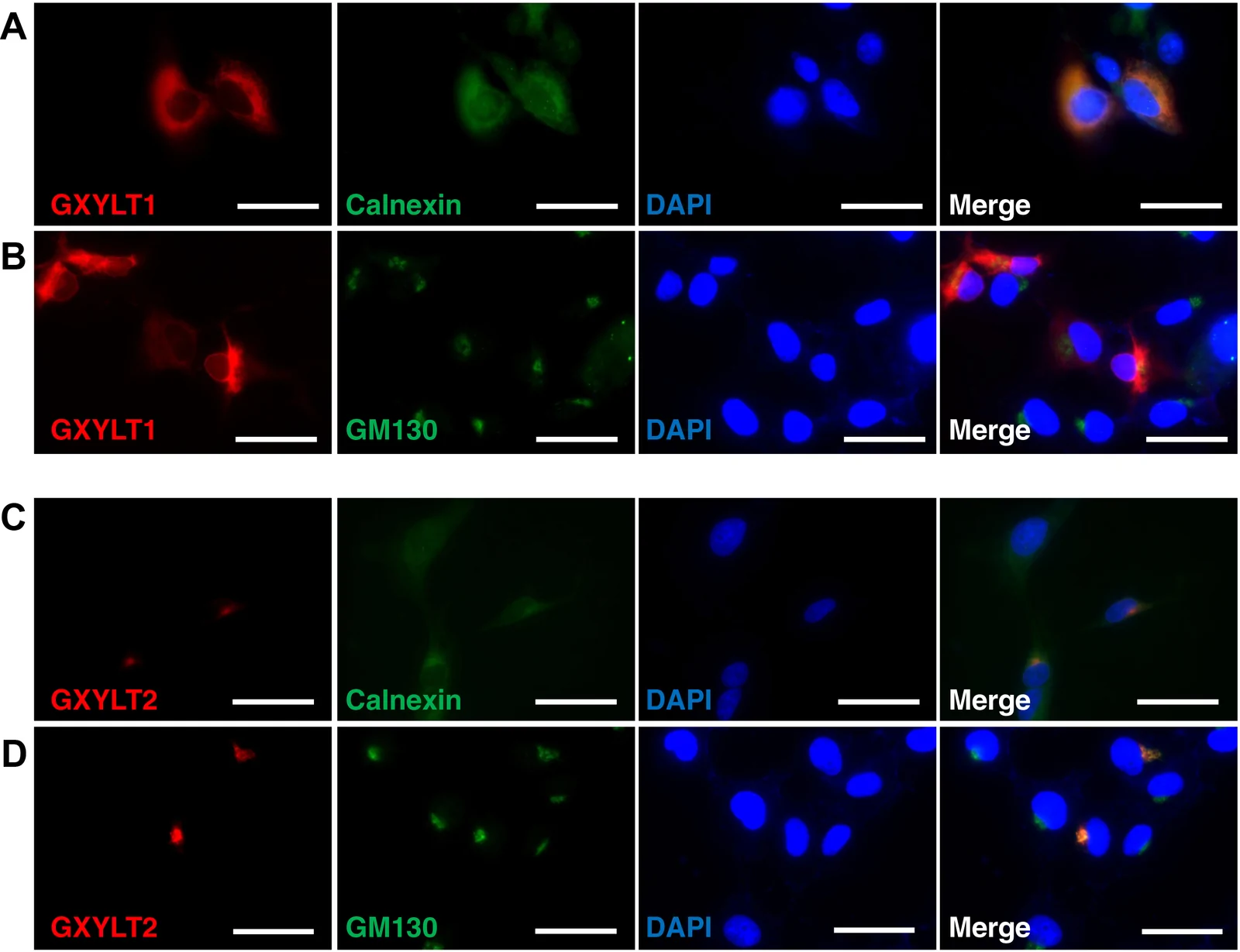
**Subcellular localization of GXYLT1 and GXYLT2 in COS-7 cells.** HA-tagged human GXYLT1 (*A* and *B*) or GXYLT2 (*C* and *D*) was transiently expressed in COS-7 cells and analyzed by immunofluorescence staining. GXYLT1 and GXYLT2 were detected using an anti-HA antibody (*red*). Calnexin (*A* and *C*) and GM130 (*B* and *D*) were used as markers for the ER and *cis*-Golgi, respectively (*green*). Nuclei were stained with DAPI (*blue*). Merged images are shown in the *right panels*. The scale bar represents 50 μm, applies to all images. DAPI, 4′,6-diamidino-2-phenylindole; ER, endoplasmic reticulum; HA, hemagglutinin.

### Contribution of GXYLT1 and GXYLT2 to O-glucosylation of CRB2 and Notch proteins

A previous report showed that EGF6 of mouse CRB2 expressed in embryoid body cells is modified with xylosylated *O*-Glc glycans (11). However, a systematic analysis of mammalian CRB2 *O*-glucosylation has not been reported. There are 15 EGF repeats in the extracellular domain (ECD) of CRB2, eight of which contain the *O*-Glc consensus sequence (Fig. 1). We overexpressed the ECD of mouse CRB2 tagged with a C-terminal MycHis sequence in HEK293T cells. The CRB2 ECD was then purified from the culture media, digested by proteases, and subjected to mass spectrometry (Table S2). A representative analysis of the CRB2 ECD protein, expressed in WT HEK293T cells, is shown in Figure 5. Tryptic peptides from CRB2 EGF5 containing the *O*-Glc consensus sequence with various *O*-Glc glycoforms eluted at 29 to 30 min during liquid chromatography (LC) separation (Fig. 5*A*). The MS/MS fragmentation pattern of the peak (m/z: 1070.61) contained a number of b- and y-ions and sequential loss of a pentose (xylose), another pentose (xylose), and a hexose (Glc) from the parental ion, confirming the identity of the peptide and the presence of an *O*-Glc trisaccharide attached to this peptide (Fig. 5*A*). The extracted ion chromatograms (EICs) showed that CRB2 EGF5 is predominantly modified with *O*-Glc mono- or disaccharides (Fig. 5*B*).

**Figure 5:**
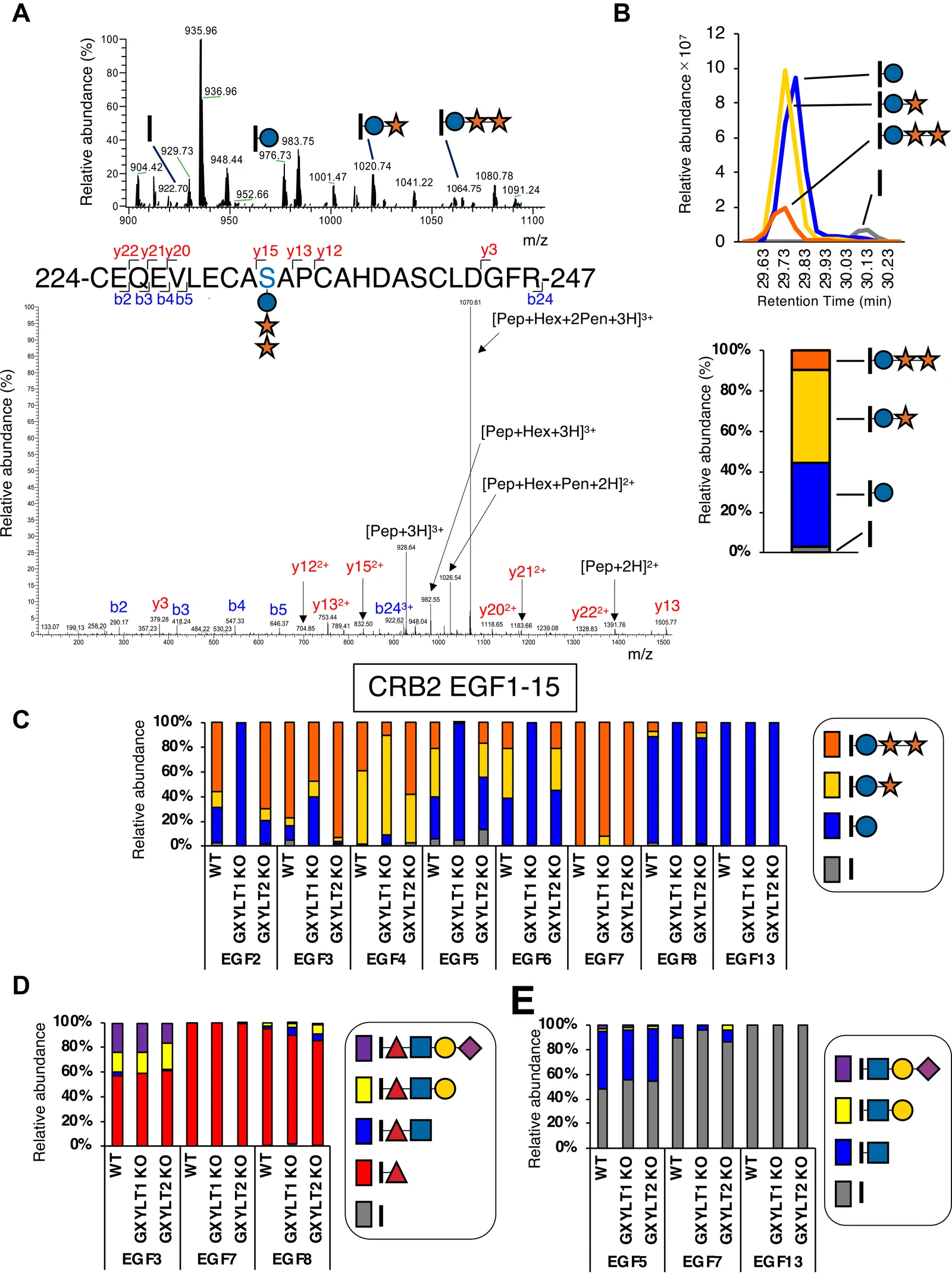
***O*-Glycosylation in CRB2 expressed in WT, *GXYLT1* KO, and *GXYLT2* KO HEK293T cells.** An example of analysis of peptides derived from CRB2 EGF5 in WT HEK293T cells (*A* and *B*). *A*, the *upper figure* shows the mass spectrum obtained at a retention time of 29 to 30 min. The figure below represents MS/MS. The identity of the peptide 224-CEQEVLECASAPCAHDASCLDGFR-247 was confirmed by numerous b- and y-ions. *B*, the *top graph* displays extracted ion chromatograms (EICs) for (glyco)peptides with various glycoforms. The *bottom graph* quantifies the ratios of (glyco)peptides with different glycoforms, with the total (glyco)peptides normalized to 100%. *C*, *O*-Glc glycans on mouse CRB2 produced in HEK293T cells were analyzed by mass spectrometry. Samples were prepared as described in the Experimental procedures section by transfecting a plasmid encoding the extracellular domain (EGF1-15) of mouse CRB2 into WT control HEK293T cells, *GXYLT1* KO cells, and *GXYLT2* KO cells. MS/MS spectra confirmed the identity of (glyco)peptides based on peptide-specific b- and y-ions and neutral loss of glycans. Quantification was performed using EIC heights, with the total amount of detected peptides or glycopeptides with different glycoforms normalized to 100% (N = 2). The *O*-fucose (*D*) and *O*-GlcNAc (*E*) glycosylation patterns of CRB2 in the aforementioned sample are shown (N = 2). *Blue circle*, glucose; *orange star*, xylose; *orange triangle*, fucose; *blue square*, GlcNAc; *yellow circle*, galactose; *purple diamond*, Neu5Ac. MS/MS spectra are shown in Figure S2. Peptide sequences, predicted and measured masses (m/z), and charge states are listed in Tables S2 and S5. CRB2, crumbs family member 2; EGF, epidermal growth factor; EIC, extracted ion chromatogram.

All eight EGF repeats containing the *O*-Glc consensus sequence of the CRB2 ECD from the culture media derived from WT control HEK293T cells were found to be modified with *O*-Glc glycans efficiently (Figs. 5*C* and S2, Table S3). The levels of xylosyl elongation of *O*-Glc glycans varied among the EGF repeats. Most *O*-Glc glycans on EGF3, EGF4, and EGF7 were xylosylated. 60 to 70% of *O*-Glc glycans on EGF2, EGF5, and EGF6 were xylosylated. Approximately 10% of *O*-Glc glycans on EGF8 were xylosylated. These data indicate broad xylosyl elongation of *O*-Glc on CRB2 EGF repeats, albeit at variable stoichiometry.

To interrogate the contribution of GXYLT1 and GXYLT2 to the *O*-glucosylation in CRB2 expressed in HEK293T cells, we performed CRISPR-mediated genome editing to generate *GXYLT1* or *GXYLT2* KO cells. We previously generated HEK293T cells with genetic deletions of both *GXYLT1* and *GXYLT2* (21). We utilized previously generated *GXYLT1* KO cells and newly created *GXYLT2* single KO cells using the same gRNAs as for *GXYLT1/GXYLT2* double KOs. Successful genome editing was confirmed by direct sequencing (Fig. S3). As in the case of WT control cells, the CRB2 ECD from the culture media derived from the *GXYLT1* or *GXYLT2* KO cells was analyzed. Genetic deletion of *GXYLT1* resulted in complete loss of xylosyl elongation in *O*-Glc glycans on EGF2, EGF5, EGF6, and EGF8, and a marked reduction in *O*-Glc trisaccharide structures on EGF3 and EGF4. On the other hand, there were no obvious changes in the *O*-glucosylation status of CRB2 derived from *GXYLT2* KO cells compared to that from the WT control cells (Fig. 5*C*). Of note, other types of EGF repeat *O*-glycosylation, such as *O*-fucosylation and *O*-GlcNAcylation, were largely unaffected by *GXYLT1* or *GXYLT2* KO (Figs. 5*D*, *E* and S2, Tables S4 and S5). Together, these data suggest a specific, nonredundant role for GXYLT1 in the elongation of *O*-Glc glycans on some CRB2 EGF repeats in HEK293T cells.

To gain further insight into the substrate specificity of xylosyl elongation of *O*-Glc glycans, we transiently expressed MycHis-tagged NOTCH1 EGF1-36 in *GXYLT1* or *GXYLT2* KO HEK293T cells, purified the secreted proteins from the culture media, and analyzed the *O*-glucosylation status of their EGF repeats (Figs. 6*A* and S4, Tables S6 and S7). Compared to those from WT control, *GXYLT1* KO decreased the percentage of xylosylated *O*-Glc glycans by more than 30% at EGF4, EGF14, EGF16, EGF19, EGF20, EGF21, and EGF33, whereas *GXYLT2* KO did not lead to an obvious change in the percentage of *O*-Glc monosaccharides at any sites. We have observed galactose-sialic acid elongation at the POGLUT1 site of NOTCH1 EGF10 (21, 23). Interestingly, the ratio of the glycoforms with galactosyl elongation at NOTCH1 EGF10 was increased in the sample from *GXYLT1* KO cells compared to that from WT control or *GXYLT2* KO cells. Again, other types of EGF repeat *O*-glycosylation, such as *O*-fucosylation and *O*-GlcNAcylation, were largely unaffected by *GXYLT1* or *GXYLT2* KO (Fig. S4, Tables S8 and S9).

**Figure 6:**
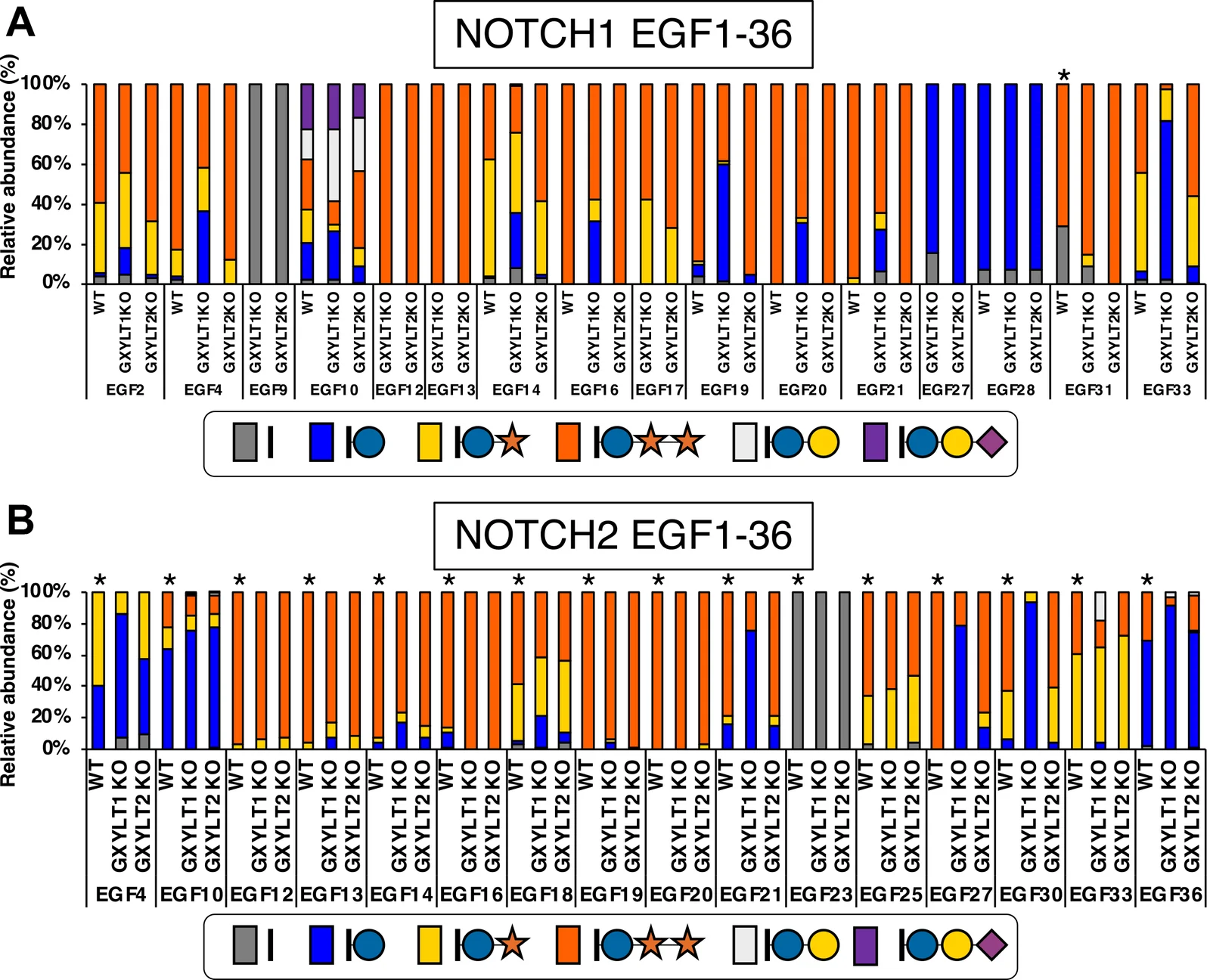
***O*-Glc glycosylation in NOTCH1 and NOTCH2 expressed in WT, *GXYLT1* KO, and *GXYLT2* KO HEK293T cells.***A*, relative abundance of *O*-Glc glycoforms in each EGF repeat from NOTCH1. *O*-Glc glycans on mouse NOTCH1 produced in HEK293T cells were analyzed by mass spectrometry. An asterisk indicates that the EGF31 data from WT cells in our previous report (21) are included for comparison with the results in the present study. Samples were prepared as described in the Experimental procedures section by transfection of a plasmid encoding the extracellular domain (EGF1-36) of mouse NOTCH1 in *GXYLT1* KO and *GXYLT2* KO HEK293T cells. MS/MS spectra identified (glyco)peptides based on peptide-specific b- and y-ions and the neutral loss of glycans. Quantification was performed using EIC height, with the total (glyco)peptides normalized to 100% (N = 2). MS/MS spectra are shown in Figure S4. Peptide sequences, predicted and measured masses (m/z), and charge states are listed in Tables S6 and S9. *B*, *O*-Glc glycans on mouse NOTCH2 produced in HEK293T cells were analyzed by mass spectrometry. *Asterisks* indicate that the data from WT cells in our previous report (21) are included for comparison with the present results. Samples were prepared as described in the Experimental procedures section by transfection of a plasmid encoding the extracellular domain (EGF1-36) of mouse NOTCH2 in *GXYLT1* KO and *GXYLT2* KO cells. MS/MS spectra identified (glyco)peptides based on peptide-specific b and y ions and neutral loss of glycans. Quantification was performed using EIC height, with the total (glyco)peptides normalized to 100% (N = 2). *Blue circle*, glucose; *orange star*, xylose; *yellow circle*, galactose; *purple diamond*, Neu5Ac. MS/MS spectra are shown in Figure S5. Peptide sequences, predicted and measured masses (m/z), and charge states are listed in Tables S10 and S11. EGF, epidermal growth factor; MS, mass spectrometry; *O*-Glc, *O*-linked glucose.

We also transiently expressed MycHis-tagged NOTCH2 EGF1-36 in *GXYLT1* or *GXYLT2* KO HEK293T cells, purified the secreted proteins from the culture media, and performed mass spectrometric analysis to examine the effect of *GXYLT1* or *GXYLT2* KO on the *O*-glucosylation status of 16 potential *O*-Glc sites in NOTCH2 (Figs. 6*B* and S5, Tables S10 and S11). We compared the *O*-glucosylation status of the EGF repeats in NOTCH2 expressed in *GXYLT1* or *GXYLT2* KO cells with that in WT control HEK293T cells as previously reported by us (21). As in the case of NOTCH1, the effect of the loss of *GXYLT1* on the levels of xylosyl elongation was observed in some EGF repeats. Namely, *GXYLT1* KO decreased the percentage of xylosylated *O*-Glc glycans by approximately 40%, 15%, 60%, 80%, 50%, and 20% at EGF4, EGF18, EGF21, EGF27, EGF30, and EGF36, respectively. In addition, the glycoforms with hexosyl elongation were detected on EGF10, EGF33, and EGF36 in the sample from *GXYLT1* KO cells. In the samples from *GXYLT2* KO cells, there were no obvious changes in the percentage of *O*-Glc monosaccharides at most EGF repeats compared to those in the WT control cells; however, a change of approximately 10% was observed on EGF4 and EGF10. A subtle change in galactosyl elongation of *O*-Glc on EGF10 and EGF36 was also observed in *GXYLT2* KO cells. We conclude that in HEK293T cells, the loss of *GXYLT1* has a much bigger impact on NOTCH2 glycosylation compared to the loss of *GXYLT2*. Together with the previously-mentioned mass spectrometric analysis on CRB2, these observations suggest that the xylosyl elongation of *O*-Glc glycans on the EGF repeats of mammalian proteins is primarily mediated by GXYLT1.

### The enzymatic activity of GXYLT1 is required for the regulation of CRB2, NOTCH1, and NOTCH2 trafficking

We previously reported that NOTCH1 and NOTCH2 trafficking to the cell surface were significantly decreased when they were overexpressed in HEK293T cells lacking both *GXYLT1* and *GXYLT2* (21). To determine the contribution of each enzyme to the trafficking of CRB2, NOTCH1, and NOTCH2, we performed secretion assays in which either MycHis-tagged CRB2 EGF1-15, NOTCH1 EGF1-36, or NOTCH2 EGF1-36 was expressed together with human IgG as a control in WT, *GXYLT1*, and *GXYLT*2 KO HEK293T cells. We found that GXYLT1 was necessary for the proper trafficking of CRB2, NOTCH1, and NOTCH2 when they were overexpressed in HEK293T cells (Fig. S6). To gain mechanistic insight into the requirement of GXYLT1 for the proper trafficking of CRB2, NOTCH1, and NOTCH2, we next asked whether GXYLT1 exerts its function in an enzymatic activity-dependent manner. A DXD motif is the conserved active center of the catalytic domain in many glycosyltransferases (24). We previously reported that Q330 in the DQD motif in mouse XXYLT1 is required for its enzymatic activity (25). This DQD sequence is conserved among GXYLT1, GXYLT2, and XXYLT1, with the 312-DQD-314 motif in mouse GXYLT1 corresponding to the 329-DQD-331 motif in mouse XXYLT1 (17). Accordingly, we utilized three mutant versions of GXYLT1, each with a single amino acid substitution in the 312-DQD-314 motif, in cell-based rescue experiments.

We performed a secretion assay of CRB2 ECDs with WT and DQD-mutated GXYLT1 (Fig. 7). Two bands were detected, corresponding to the monomeric and dimeric forms of CRB2 ECD, consistent with the previously reported homodimerization of CRB2 (26, 27). These bands were observed even under reducing SDS-PAGE conditions, suggesting that the dimerization is mediated by noncovalent interactions. Secretion of CRB2 EGF1-15 in the culture media was decreased in *GXYLT1* KO cells compared to WT HEK293T cells, although no differences were observed in the cell lysates (Fig. 7, *A* and *B*). This secretion defect was rescued by coexpression of WT GXYLT1, although that was not a complete rescue (Fig. 7, *A* and *B*). The secretion defect was not rescued by co-expression of GXYLT1 D312A or D314A mutants (Fig. 7, *A* and *B*). Unexpectedly, coexpression of GXYLT1 Q313A mutant rescued the secretion defect of CRB2 EGF1-15 in *GXYLT1* KO cells (Fig. 7, *A* and *B*).

**Figure 7:**
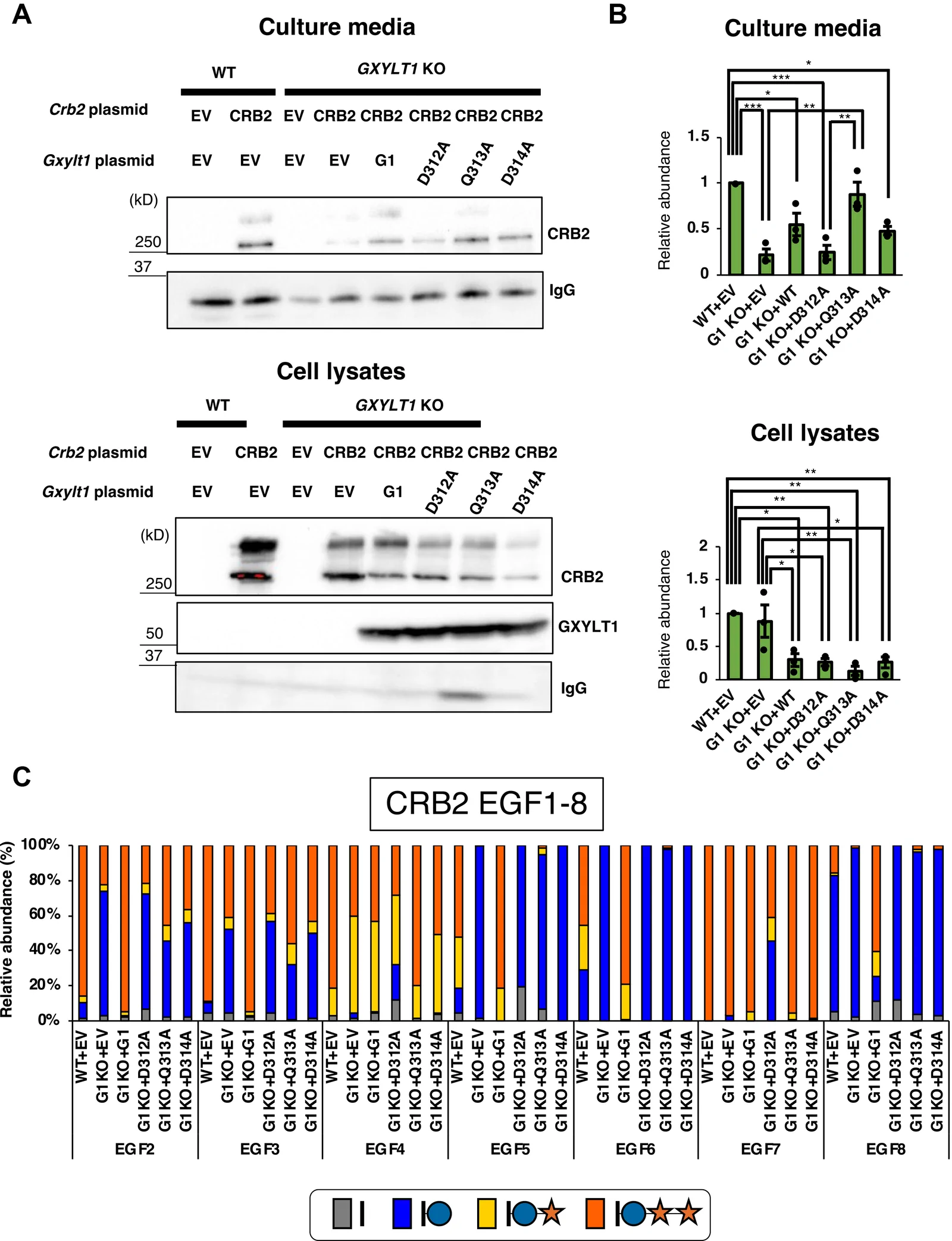
**Xylosyl elongation of *O*-Glc glycans by GXYLT1 contributes to the trafficking of CRB2 in HEK293T cells.***A*, Western blotting analysis of cell lysates and culture media after the plasmids encoding MycHis-tagged CRB2 EGF1-15 were transfected in HEK293T cells. The plasmid encoding human IgG was cotransfected for normalization. Rescue experiments with cotransfection of an empty vector (EV), WT GXYLT1 (G1), its three DQD mutants, namely, D312A, Q313A, and D314A, were also performed. Three independent experiments were performed. *B*, quantification of band intensities of the data from (*A*). One-way ANOVA followed by Tukey’s multiple comparisons test was performed. ∗: *p* < 0.05, ∗∗: *p* < 0.01, ∗∗∗: *p* < 0.001. Only statistically significant comparisons are indicated. *C*, mass spectrometric analysis. Relative abundance of *O*-Glc glycoforms at the indicated EGF repeats from CRB2 EGF1-8 expressed in WT or *GXYLT1* KO HEK293T cells cotransfected with WT GXYLT1 or its D312A, Q313A, or D314A mutants. MS/MS spectra confirmed the identity of the (glyco)peptides based on the presence of peptide-specific b- and y-ions and the predicted neutral loss of glycans. Quantification was performed using EIC height, with the total amount of detected peptides or glycopeptides with different glycoforms as 100% (N = 2). *Blue circle*, glucose; *orange star*, xylose. MS/MS spectra are shown in Figure S7. Peptide sequences, predicted and measured masses (m/z), and charge states are listed in Table S12. CRB2, crumbs family member 2; EGF, epidermal growth factor; EIC, extracted ion chromatogram; MS, mass spectrometry; *O*-Glc, *O*-linked glucose.

The unexpected rescue of CRB2 trafficking by coexpression of GXYLT1 Q313A mutant can be due either to a nonenzymatic function of GXYLT1 or residual xylosyltransferase activity of this mutant. To distinguish between these alternative scenarios, we performed mass spectrometric analysis on CRB2 EGF1-8 coexpressed with WT or the DQD-mutated GXYLT1 because the yield of CRB2 EGF1-8 was higher than that of CRB2 EGF1-15 and because it contained all EGF repeats that were modified with xylosyl elongated forms of *O*-Glc glycans as shown in Figure 5. As in the analysis of CRB2 EGF1-15 (Fig. 5*C*), the levels of xylosyl elongation at EGF2, EGF3, EGF4, EGF5, and EGF6 on CRB2 EGF1-8 expressed in *GXYLT1* KO cells were decreased compared to those in WT control cells (Figs. 7*C* and S7, Table S12). Coexpression of WT GXYLT1 in *GXYLT1* KO cells increased the levels of xylosyl elongation on EGF2, EGF3, EGF5, EGF6, EGF7, and EGF8, but not on EGF4, compared to the control transfection of the empty vector. Coexpression of GXYLT1 D312A mutant in *GXYLT1* KO cells did not rescue the defect in xylosyl elongation but rather decreased the levels of xylosyl elongation on EGF2, EGF4, and EGF7. Notably, the xylosyl elongation on EGF4 expressed in *GXYLT1* KO cells was fully rescued by coexpression of GXYLT1 Q313A mutant to the same extent as that in WT control cells. The levels of xylosyl elongation on EGF2 and EGF3 were rescued by coexpression of GXYLT1 Q313A approximately 20% and 10%, respectively, and a slight increase in xylosyl elongation on EGF7 was also observed. We did not observe any change in the levels of xylosyl elongation on EGF5, EGF6, and EGF8 upon coexpression of GXYLT1 Q313A. Coexpression of GXYLT1 D314A in *GXYLT1* KO cells caused an increase in the level of xylosyl elongation only at EGF2 in CRB2 EGF1-8 by approximately 20%. These results indicate that both GXYLT1 Q313A and D314A mutants retain residual enzymatic activity, which likely explains their unexpected promotion of CRB2 secretion. Altogether, our cell culture and mass spectrometry data indicate that the enzymatic activity of GXYLT1 is required for proper trafficking of CRB2 ECDs in HEK293T cells.

Next, NOTCH1 and NOTCH2 were also analyzed. Secretion of NOTCH1 and NOTCH2 ECD was significantly decreased in *GXYLT1* KO HEK293T cells compared to that in WT cells and was rescued to the same level as in WT cells by cotransfection of WT GXYLT1 (Fig. 8, *A* and *B*). None of the GXYLT1 DQD mutants significantly rescued the secretion defect in *GXYLT1* KO cells (Fig. 8, *A* and *B*).

**Figure 8:**
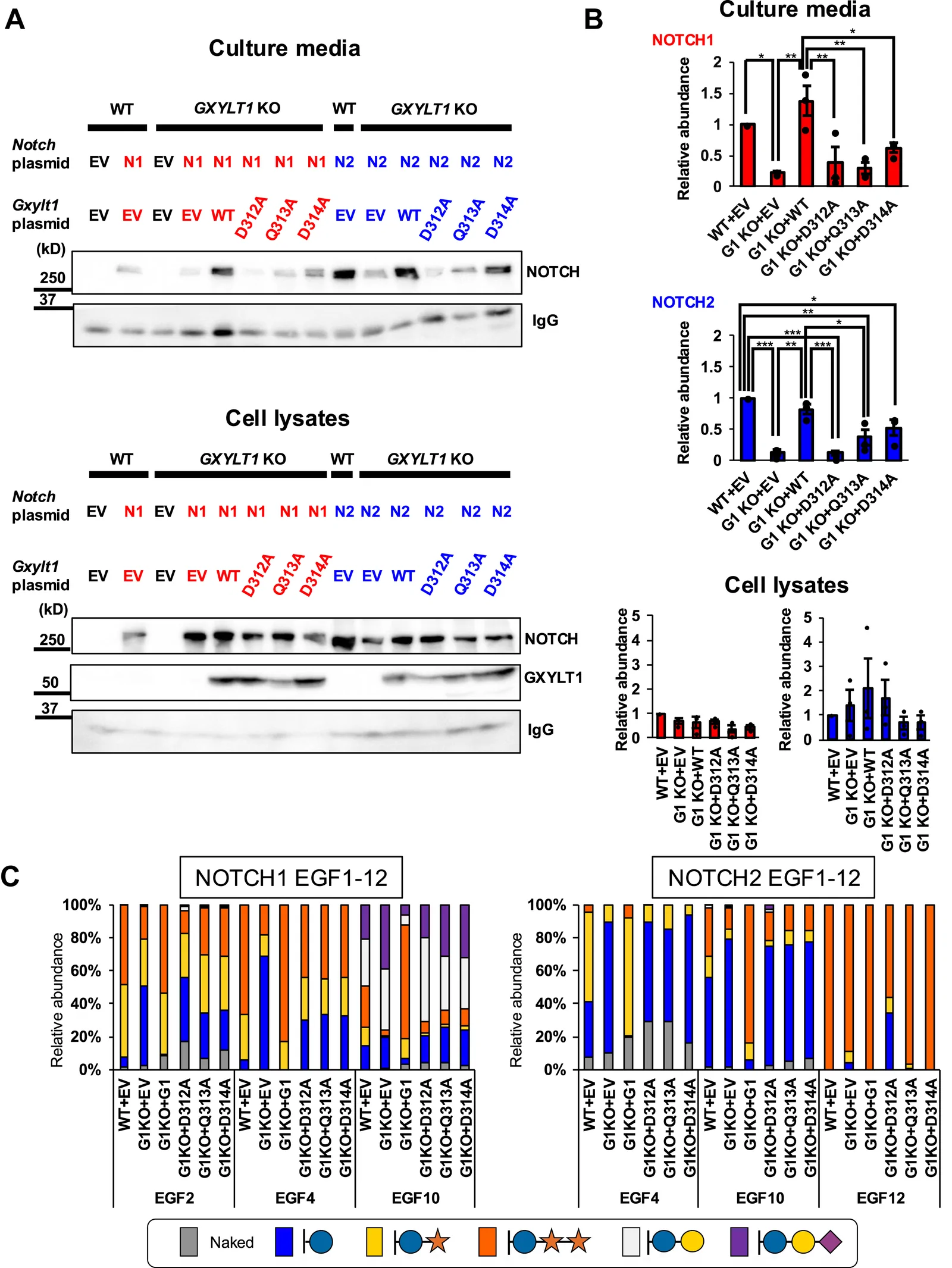
**Xylosyl elongation of *O*-Glc glycans by GXYLT1 contributes to the trafficking of NOTCH1 and NOTCH2 in HEK293T cells.***A*, Western blotting analysis of cell lysates and culture media after the plasmids encoding the MycHis-tagged EGF1-36 of NOTCH1 (N1) or NOTCH2 (N2) were transfected in WT or *GXYLT1* KO HEK293T cells. The plasmid encoding human IgG was cotransfected for normalization. Rescue experiments with cotransfection of an empty vector (EV), WT GXYLT1 (G1), its three DQD mutants, namely, D312A, Q313A, and D314A, were also performed. Three independent experiments were performed. *B*, quantification of band intensities of the data from (*A*). One-way ANOVA followed by Tukey’s multiple comparisons test was performed. ∗: *p* < 0.05, ∗∗: *p* < 0.01, ∗∗∗: *p* < 0.001. Only statistically significant comparisons are indicated. *C*, mass spectrometric analysis. Relative abundance of *O*-Glc glycoforms at EGF2, EGF4, or EGF10 from NOTCH1 EGF1-12, and those at EGF4, EGF10, and EGF12 from NOTCH2 EGF1-12 expressed in WT or *GXYLT1* KO HEK293T cells cotransfected with WT GXYLT1 or its D312A, Q313A, or D314A mutants. MS/MS spectra confirmed the identity of the (glyco)peptides based on the presence of peptide-specific b- and y-ions and the neutral loss of glycans. Quantification was performed using EIC height, with the total amount of detected peptides or glycopeptides with different glycoforms as 100% (N = 2). *Blue circle*, glucose; *orange star*, xylose; *yellow circle*, galactose; *purple diamond*, Neu5Ac. MS/MS spectra are shown in Figures S8 and S9. Peptide sequences, predicted and measured masses (m/z), and charge states are listed in Tables S13 and S14. EGF, epidermal growth factor; MS, mass spectrometry; *O*-Glc, *O*-linked glucose.

To examine the *O*-glucosylation status of NOTCH1 and NOTCH2 coexpressed with WT or mutated versions of GXYLT1 in *GXYLT1* KO cells, we performed mass spectrometric analysis using a shorter version of Notch ECDs, EGF1-12, since its yield was much higher than that of longer ECDs (*e.g.*, EGF1-36). We confirmed that the *O*-glucosylation patterns in NOTCH1 EGF1-12 and EGF1-36 expressed in WT cells were very similar (Figs. 8*C*, 6*A* and S8, Table S13). As expected, upon coexpression of WT GXYLT1, the levels of xylosyl elongation of *O*-Glc glycans on EGF2 and EGF4 of NOTCH1 EGF1-12 in *GXYLT1* KO cells became comparable to those on NOTCH1 EGF1-12 from WT cells (Fig. 8*C*). As for EGF10 of NOTCH1, where galactosyl elongation was observed in WT cells, the xylosyl elongated forms of *O*-Glc glycans became the predominant glycoform upon coexpression of WT GXYLT1 (Fig. 8*C*). Coexpression of each of the three GXYLT1 mutants only slightly increased the levels of xylosyl elongation of *O*-Glc glycans on EGF2, EGF4, and EGF10 of NOTCH1 EGF1-12 in *GXYLT1* KO cells compared to those in *GXYLT1* KO cells cotransfected with the empty vector (Fig. 8*C*), suggesting that these mutations indeed severely impact the xylosyltransferase activity of GXYLT1.

As in the previous case of NOTCH1, the *O*-glucosylation status in NOTCH2 EGF1-12 and EGF1-36 expressed in WT cells was very similar (Figs. 8*C*, 6*B* and S9, Table S14). We observed a clear decrease in the levels of xylosyl elongation on EGF4 and EGF10 and a subtle decrease in that on EGF12 of *GXYLT1* KO cells compared to WT cells. Coexpression of WT GXYLT1 rescued the levels of xylosyl elongation of *O*-Glc glycans on all three EGF repeats in *GXYLT1* KO cells, which became higher than those on EGF4 and EGF10 from WT cells, probably due to the overexpression of the enzyme (Fig. 8*C*). Coexpression of each of the three mutated GXYLT1 failed to restore the levels of xylosyl elongation of *O*-Glc glycans on these EGF repeats in NOTCH2 EGF1-12 in *GXYLT1* KO cells (Fig. 8*C*). Together, the results from secretion assays and mass spectrometry indicate that the xylosyltransferase activity of GXYLT1 is critical for the trafficking of NOTCH1 and NOTCH2 ECDs and that each amino acid within the 312-DQD-314 motif in GXYLT1 is important for its enzymatic activity.

### Gxylt1 is essential for mouse embryonic development but Gxylt2 is not

To examine the contribution of *Gxylt1* and *Gxylt2* to mouse embryonic development, we used the CRISPR-Cas9 system to delete two exons in each gene and to generate loss-of-function alleles (Fig. 9, *A*–*C*). Heterozygous animals were viable and fertile without any gross morphological abnormalities. We next set heterozygous intercrosses to examine whether *Gxylt1* and *Gxylt2* are required for mouse embryonic development. *Gxylt2*^*−/−*^ animals reached the weaning stage at the expected Mendelian ratio (Fig. 9*D*) and did not show any gross morphological phenotypes despite a complete loss of *Gxylt2* mRNA (Fig. 9*E*). However, we did not obtain any *Gxylt1*^*−/−*^ animals at the weaning stage out of 111 progeny from *Gxylt1*^*+/−*^ intercrosses, indicating that *Gxylt1* is an essential gene (Fig. 9*D*). We next set timed mating between *Gxylt1*^*+/−*^ animals and analyzed several litters at around E10. At this stage, we obtained *Gxylt1*^*−/−*^ embryos at the expected Mendelian ratio (Fig. 9*D*). WT and *Gxylt1*^*+/−*^ embryos looked normal and showed the features expected at this developmental stage, including distinct hindbrain, midbrain, and forebrain regions and recognizable olfactory placode and lens pit (Fig. 9*F*). However, *Gxylt1*^*−/−*^ embryos showed a significantly smaller crown-rump length (CRL) compared to their WT and heterozygous siblings (Fig. 9*G*) and exhibited developmental delay and morphological abnormalities, including an underdeveloped brain region, lack of an olfactory placode, and a shallow optic pit compatible with earlier developmental stages (Fig. 9*F*). However, all *Gxylt1*^*−/−*^ embryos completed the turning process, suggesting that their development is stalled between E8.5 and E9. We conclude that *Gxylt1* plays a key role in mouse embryonic development, while *Gxylt2* is dispensable for this process.

**Figure 9:**
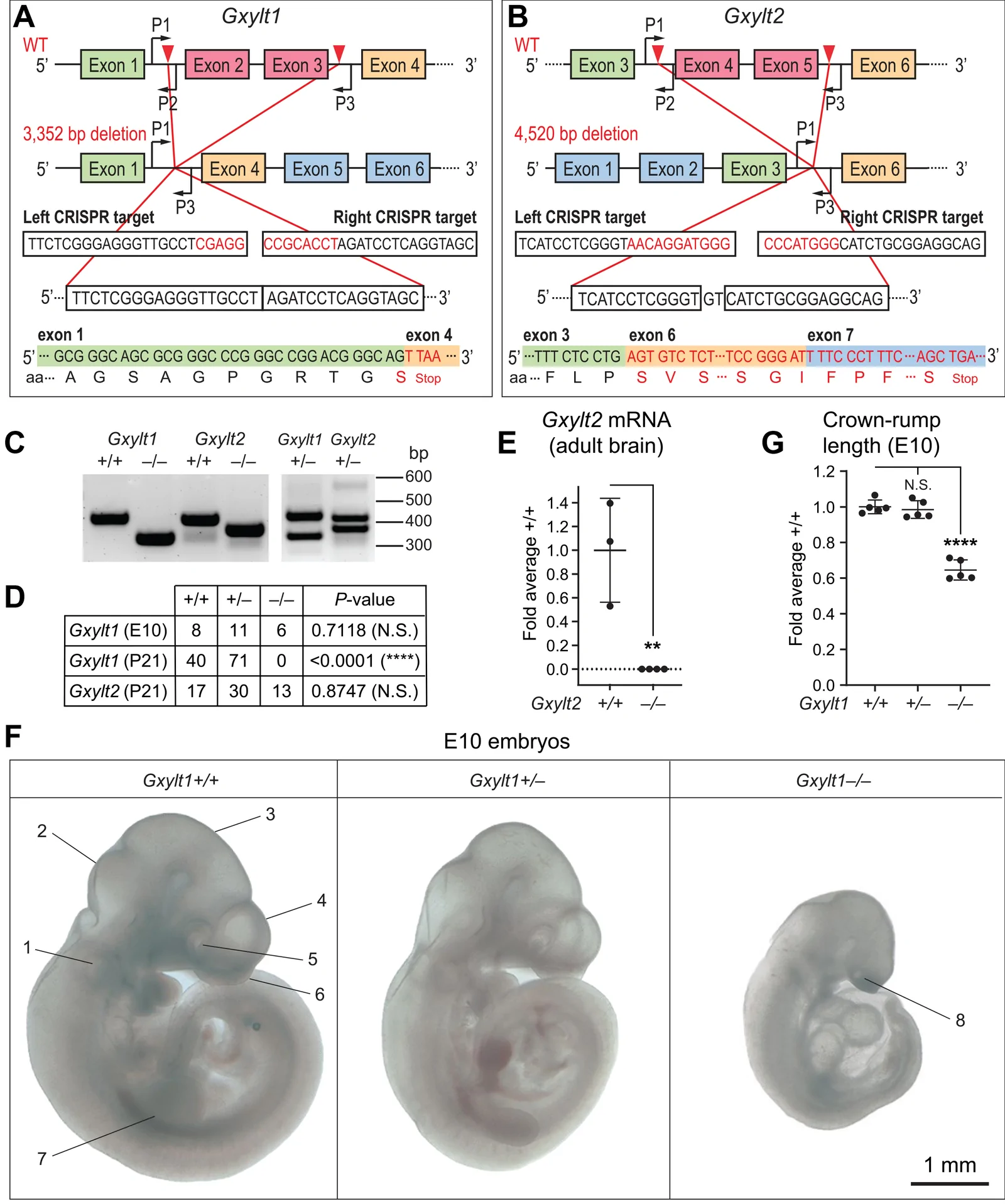
***Gxylt1* is essential for mouse embryonic development but *Gxylt2* is not.***A* and *B*, schematic of the strategy used to generate *Gxylt1* (*A*) and *Gxylt2* (*B*) mutant alleles. Genomic regions are not drawn to scale. *Red triangles* show sgRNA binding sites. *Pink boxes* show exons that are deleted by Cas9 endonuclease activity. Base pairs in *red* in *left* and *right* CRISPR target sequences are those that are deleted in the final allele upon non-homologous end joining. Base pairs and amino acids in *red* in the codons at the *bottom* of each panel denote out-of-frame regions of mRNA/protein in each allele. P1–P3, primer locations. *C*, PCR genotyping gels showing distinct bands for WT and mutant alleles for each gene. *D*, table indicates the number of progeny for each genotype at the indicated ages, along with *p* values determined with Chi-square test. N.S., not significant; ∗∗∗∗*p* < 0.0001. E10, embryonic day 10; P21, postnatal day 21. *E*, qRT-PCR data on adult brain lysates from the indicated genotypes. Each circle represents an animal. *Horizontal lines* show mean ± SD. An unpaired one-tailed *t* test was performed. ∗∗*p* < 0.01. *F*, Brightfield images of E10 embryos of the indicated genotypes. 1, otic vesicle; 2, region overlaying future hindbrain; 3, region overlaying future midbrain; 4, region overlaying future forebrain; 5, lens pit; 6, region of olfactory placode; 7, forelimb bud; 8, optic pit. The scale bar applies to all three images. *G*, crown-rump length measurements of the indicated genotypes. Each *circle* represents an embryo. *Horizontal lines* show mean ± SD. One-way ANOVA followed by Dunnett’s multiple comparisons test was performed. N.S., not significant; ∗∗∗∗*p* < 0.0001. qRT-PCR, quantitative reverse transcription polymerase chain reaction.

## Discussion

In the present study, we showed that of the two mammalian enzymes capable of adding xylose to *O*-Glc on EGF repeats (GXYLT1 and GXYLT2), one of them (GXYLT1) plays a major role in xylosyl elongation of *O*-Glc glycans on CRB2, NOTCH1, and NOTCH2 overexpressed in HEK293T cells. This GXYLT1-mediated modification in the ER is required for the trafficking of these proteins to the cell surface. Furthermore, we showed that *Gxylt1* KO mice are embryonically lethal, whereas *Gxylt2* KO mice are viable and do not exhibit any overt morphological phenotypes. Therefore, despite the high degree of sequence similarity and comparable enzymatic activities of these two enzymes *in vitro* (16), our work suggests a significant difference in their functional importance in protein quality control and mammalian development.

We examined the role of xylosyl elongation of CRB2, NOTCH1, and NOTCH2 in protein quality control of the secretory pathway and the contribution of GXYLT1 and GXYLT2 to this process in HEK293T cells. Secretion assays revealed reduced secretion of CRB2 and NOTCH1/2 in *GXYLT1* KO cells, but not in *GXYLT2* KO cells. Mass spectrometric analysis showed that endogenous GXYLT1 makes a major contribution to xylosyl elongation, with complete loss of xylosylation on CRB2 EGF5 and EGF6 in *GXYLT1* KO cells. These results indicate that the relative contributions of endogenous GXYLT1 and GXYLT2 vary among individual EGF repeats and that GXYLT1 is the predominant enzyme responsible for modification of some *O*-Glc sites in HEK293T cells. Structural analysis of enzyme-substrate complexes, as performed for POGLUT1 and POFUT1 (28, 29, 30, 31), could clarify the key structural basis for EGF specificity. Both GXYLT1 and GXYLT2 require intact EGF repeat structures for activity, a feature shared by ER-localized, EGF repeat-modifying glycosyltransferases such as POFUT1, POGLUT1, EOGT, and XXYLT1 (32). Importantly, the predominant contribution of GXYLT1 in HEK293T cells does not imply an absence of biochemical redundancy between GXYLT1 and GXYLT2. Our previous study showed that either GXYLT1 or GXYLT2 can restore NOTCH1 and NOTCH2 secretion in *GXYLT1/GXYLT2* double KO cells, demonstrating overlapping biochemical activities of the two enzymes (21). Nevertheless, GXYLT1 showed greater rescue efficiency than GXYLT2 despite the higher expression level of *GXYLT2* mRNA in HEK293T cells (21). Together with the present findings, these observations suggest that the predominant contribution of GXYLT1 in HEK293T cells may reflect differences in protein abundance, subcellular localization, substrate accessibility, or intrinsic enzymatic properties between GXYLT1 and GXYLT2.

Despite their overlapping biochemical activities, *GXYLT1* and *GXYLT2* are not biologically redundant during mouse development. The distinct phenotypes of *Gxylt1* and *Gxylt2* KO mice may reflect differences in their temporal or spatial expression, subcellular localization, accessibility to physiological substrates, or substrate preferences. Consistent with this possibility, our immunofluorescence analyses revealed distinct subcellular localization patterns of the two enzymes: GXYLT1 was predominantly localized to the ER, whereas GXYLT2 showed substantial colocalization with the *cis*-Golgi marker GM130 and little overlap with the ER marker calnexin. Such differences in subcellular localization may influence the access of GXYLT1 and GXYLT2 to physiological substrates. Further studies of their spatiotemporal expression, subcellular localization, and substrate preferences during development will be required to determine the molecular basis for their distinct biological functions.

In *Poglut1* KO embryos, both CRB2 and NOTCH1 are affected, leading to lethality before the midgestation turning process occurs (8, 11). In contrast, the *Gxylt1* KO embryos consistently complete the midgestation turning. These observations strongly suggest that the phenotype of *Gxylt1* KO mice is less severe than that of *Poglut1* KO mice. This suggests that *O*-Glc monosaccharides on specific EGF repeats may function independently of xylosyl elongation, akin to the distinct roles of *O*-fucose monosaccharide *versus* GlcNAc elongation in Notch signaling (33). The molecular mechanism underlying the embryonic lethality of *Gxylt1* KO mice remains to be elucidated.

We have, for the first time, elucidated the complete landscape of the *O*-glucosylation status on all EGF repeats with the *O*-Glc consensus sequence in CRB2 overexpressed in HEK293T cells. All such repeats were efficiently modified with *O*-Glc glycans, showing distinct xylosyl elongation patterns specific to each EGF repeat. Notably, six out of the eight sites were predominantly modified with xylosyl forms, while EGF8 and EGF13 were almost exclusively modified with *O*-Glc monosaccharides. This is reminiscent of the xylosyl elongation of *O*-Glc glycans observed on specific EGF repeats in *Drosophila* Notch and Crb (34, 35, 36). It remains unclear how the levels of xylosyl elongation of *O*-Glc glycans are regulated site-specifically. Analysis of CRB2 EGF1-8 and EGF1-15 revealed that EGF8 was predominantly modified with *O*-Glc monosaccharide in both cases, suggesting that the region from EGF9 through EGF15 does not affect xylosyl elongation on EGF8. The predominance of *O*-Glc monosaccharides in certain EGF repeats, observed in WT HEK293T cells (this study), *Drosophila* embryos (36), and *Drosophila* S2 cells (34, 35, 36), suggests that there might be a mechanism that inhibits the activity of GXYLT enzymes on specific EGF repeats. Variations in xylosylation efficiency may result from differences in binding strength between GXYLT enzymes and EGF repeats. While cocrystal structures of XXYLT1 with Xyl-Glc-EGF repeats have clarified the mechanism of the second xylose addition (25), structural details of the first xylose addition by GXYLT1 and GXYLT2 are unavailable, preventing evaluation of this mechanism. POGLUT1 recognizes amino acids in and around the *O*-Glc consensus sequence and an aromatic residue conserved at a site two amino acids before the fourth cysteine residue of properly folded EGF repeats (28, 29). Thus, delays in the folding of individual EGF repeats and inefficient *O*-Glc addition by POGLUT1 may both limit their availability for xylosylation before ER exit. The effects of mutations in the DQD motif of GXYLT1 on xylosyl elongation also varied among individual EGF repeats. Although these residues were predicted to be important for catalytic activity based on the structure of mouse XXYLT1 (25), increased xylosyl elongation above the levels observed in *GXYLT1* KO cells at selected sites suggests that the GXYLT1 DQD mutants retain some functional activity in cells. Residual catalytic activity of glycosyltransferase mutants carrying substitutions in DXD-like motifs is not unprecedented; mutations in the DXD-like motif of *Drosophila* Rumi/POGLUT1 reduced but did not completely abolish its *O*-glucosyltransferase activity (7). Interestingly, the D312A mutant decreased xylosyl elongation at several sites, suggesting a dominant-negative-like effect. One possible explanation is that a DQD mutant may compete with endogenous GXYLT2 for access to shared substrates while exhibiting altered catalytic activity, thereby reducing their modification by GXYLT2. However, our data do not demonstrate a direct interaction between GXYLT1 and GXYLT2, and further biochemical analyses of the catalytic activities and substrate specificities of the GXYLT1 DQD mutants will be required to clarify the underlying mechanism.

Although the present study demonstrates that xylosyl elongation of *O*-Glc glycans promotes the quality control and secretion of overexpressed CRB2 and Notch extracellular domains in HEK293T cells, caution should be exercised in extrapolating these findings directly to endogenous full-length proteins. The use of extracellular domain fragments enabled comprehensive site-specific glycoproteomic analyses by LC-MS/MS but might not fully recapitulate the trafficking and maturation of native membrane-bound proteins. Therefore, whether xylosyl elongation of *O*-Glc glycans similarly regulates the quality control and intracellular trafficking of endogenous CRB2 and Notch proteins under physiological conditions remains to be confirmed.

Previous studies showed that GXYLT deficiency has little effect on the trafficking of endogenous NOTCH1 and NOTCH2 proteins in HEK293T cells under physiological conditions (21), whereas the present study demonstrates a clear requirement for xylosyl elongation of *O*-Glc glycans in the secretion of overexpressed CRB2 and NOTCH1/2 extracellular domains in HEK293T cells. One possible explanation is that *O*-Glc xylosylation becomes particularly important when substrate proteins are produced at high levels, thereby increasing the demand on the ER protein quality control machinery. Thus, the contribution of *O*-Glc xylosylation to protein quality control and trafficking may depend on the expression level of substrate proteins and the cellular context. Under conditions of high substrate expression, *O*-Glc xylosylation may function as an additional layer of ER quality control to facilitate efficient folding and secretion of EGF repeat-containing proteins. Whether this mechanism also contributes to the maturation of endogenous substrates under physiological or pathological conditions remains an important question for future studies.

The subcellular localization of *N*-glycosylation biosynthesis, which is closely associated with quality control of *N*-glycosylated proteins, has been extensively investigated. In this study, multiple lines of evidence demonstrate that xylosyl elongation of *O*-Glc glycans occurs in the ER. In addition to the glycopeptide analyses showing xylosyl-elongated *O*-Glc glycans on proteins carrying Endo H-sensitive *N*-glycans, immunofluorescence analyses showed that intracellular CRB2 EGF1-8 was predominantly localized in the ER. Furthermore, xylosyl-elongated *O*-Glc was detected on CRB2 EGF1-8 purified from cell lysates. Consistent with these findings, GXYLT1 was predominantly localized in the ER. Fringe, localized in the Golgi apparatus, transfers GlcNAc to the *O*-fucose monosaccharide on Notch EGF repeats, controlling Notch–ligand interactions (37, 38). CRB2 and Notch have multiple consecutive EGF repeats, each containing six cysteine residues that form three disulfide bonds in a defined pattern within the ER, crucial for protein structural maturation. It has been reported that protein disulfide isomerase A3 plays an important role in CRB2 trafficking (39). Information regarding the suborganellar localization of glycosyltransferases modifying EGF repeats within the ER is limited, and research in this area is ongoing. It is a key challenge to investigate whether molecular chaperones, with distinct expression repertoires in different cells and tissues, cooperate with these glycosyltransferases.

The functions of *O*-Glc glycans on EGF repeats extend beyond protein quality control and trafficking. *O*-Glc glycans interact with a hydrophobic pocket of EGF repeats in the folded state, inhibiting nonspecific protein interactions (40), and also form intramolecular interactions, stabilizing glycosylated EGF repeat structure (20). Xylosyl transfer by GXYLT1, followed by another xylose addition by XXYLT1, might contribute to some of these roles, thereby modulating CRB2 and Notch protein activity. In addition, *O*-Glc glycans regulate Notch signaling after the receptor reaches the cell surface. Previous studies in *Drosophila* and mammalian cells showed that *O*-Glc glycans regulate Notch activation at a step downstream of ligand binding but upstream of proteolytic cleavage (7, 8). Thus, the effects of *O*-Glc glycans on protein maturation and trafficking and their effects on cell-surface activation are not necessarily mutually exclusive. Xylosyl elongation may contribute to efficient maturation and trafficking of Notch receptors, while *O*-Glc glycans on receptors that reach the cell surface may further regulate their competence for ligand-induced activation. Together, these multiple levels of regulation may determine the overall signaling activity of Notch receptors. Further studies will be required to define how xylosyl elongation of *O*-Glc glycans contributes to each of these processes.

## Experimental procedures

### Cell culture

HEK293T cells and COS-7 cells obtained from the American Type Culture Collection were cultured at 37 °C and 5% CO_2_ in a Dulbecco's modified Eagle's medium (DMEM) high-glucose medium supplemented with 5% fetal calf serum (FCS), penicillin, and streptomycin. *GXYLT1* KO HEK293T cells and *GXYLT2* KO HEK293T cells were generated using CRISPR/Cas9 technology as previously described (21). The Cas9-expression vector, pX330-Cas9-mEGFP, was kindly provided by Dr Taroh Kinoshita (41). The guide RNAs (gRNAs) targeting *GXYLT1* and *GXYLT2* are 5′-CGCAGCGCATCCCGGCGTGT-3′ and 5′-GGTGGCCTGTGGCAATCGGC-3′, respectively. Each gRNA was inserted into the BbsI site of pX330-Cas9-mEGFP. Successful insertion was confirmed by direct DNA sequencing. HEK293T cells grown in a 10-cm dish were transiently transfected with 5 μg of the Cas9 and gRNA expression plasmid using polyethyleneimine (PEI)-Max (Polysciences). After culturing at 37 °C for 24 h, EGFP-positive cells were single-cell sorted in 96-well plates using the FACS SORP Aria2 (BD Biosciences). Genomic DNAs of each clone were isolated using 50 mM NaOH. The gRNA-targeted regions were Polymerase Chain Reaction (PCR)-amplified using KOD Fx-neo (TOYOBO) and GoTaq (Promega) polymerases and the following primers: 5′- GGTTGCCCAATGTCACAAACT-3′ and 5′- GCGCATGACTGGGTCAAAAG-3′ for *GXYLT1*; 5′- TCCAAACAGTGACTCATCCTG-3′ and 5′- AACACTGTGCCATGATTTCTG-3′ for *GXYLT2.* To identify the heterozygosity of the KO alleles, PCR products were cloned into pMD20-T vector using Mighty TA-cloning kit (TaKaRa). Genomic DNA sequences of each allele were determined by the direct DNA sequencing of at least 12 TA clones.

### Plasmids and site-directed mutagenesis

NOTCH1 and NOTCH2 expression plasmids have been previously described (21, 42). The cDNAs encoding mouse CRB2 EGF1-15 and EGF1-8 were amplified using pCAGGS-full length mouse *Crb2* (11) as a template and two primer sets, 5′-ATATATAAGCTTTGGGGCTGGTTCCTCCAGAGACTC-3′ and 5′-ATCTAGGCGGCCGCTTCACAGAACTGGCCAGAGAATCCT-3′ and 5′-ATCTAGGCGGCCGCAGAACAGTCATGTCCACCCCAGG-3′, respectively. The amplified cDNAs were digested with restriction enzymes and cloned into pSecTag2c vector (Invitrogen, Thermo Fisher Scientific). The expression plasmid encoding C-terminally HA-tagged human full-length GXYLT1 in the pcDNA3.1 vector was purchased from GenScript (ID: OHu24264d). The expression plasmid encoding C-terminally HA-tagged human full-length GXYLT2 in the pcDNA3.1 vector was described previously (16). The expression plasmids encoding C-terminally MycHis-tagged mouse GXYLT1 and GXYLT2 in the pSecTag2c vector were described previously (17, 32). Mutations in mouse *GXYLT1* were introduced by a conventional PCR-based method using each plasmid as a template. The following primers were used for each mutant: GXYLT1 D312A: 5′- GTGGGGCGCTCAGGATCTGTTAAATATCGTGTTTTCC-3′ and 5′- GATCCTGAGCGCCCCACGTGATGTTCAGTTTGTA-3′, Q313A: 5′- GGGGCGATGCGGATCTGTTAAATATCGTGTTTTCCCACAATCC-3′ and 5′- ACAGATCCGCATCGCCCCACGTGATGTTCAGTTTGTACTTTTT-3′, and D314A: 5′- CGATCAGGCTCTGTTAAATATCGTGTTTTCCCACAATCCAG-3′ and 5′- TTAACAGAGCCTGATCGCCCCACGTGATGTTCAGTTT-3′. Introduced mutations and the absence of unintended mutations across the open reading frame were confirmed by direct DNA sequencing.

### Expression and purification of proteins

The expression plasmids used for the mass spectrometric analysis were pSecTag2c-mouse NOTCH1 EGF1-36-MycHis, pSecTag2c-mouse NOTCH1 EGF1-12-MycHis, pSecTag2c-mouse NOTCH1 EGF19-36-MycHis, pSecTag2c-mouse NOTCH2 EGF1-36-MycHis, pSecTag2c-mouse CRB2 EGF1-15-MycHis, and pSecTag2c-mouse CRB2 EGF1-8-MycHis. HEK293T cells were seeded in a 10-cm cell culture dish in a DMEM high-glucose medium supplemented with 5% FCS and transiently transfected with 5 μg/dish plasmids using PEI-Max. After 4 to 5 h, the medium was changed to 6 ml of OPTI-MEM I. The cells were cultured for another 3 days. For purification of MycHis-tagged CRB2 or Notch proteins in culture media, the media samples from the transfected cells were processed as previously described (21). For the purification of CRB2 EGF1-8 in cells, cells were washed with PBS twice and lysed in 10 mM Tris-HCl pH 8.0 containing 150 mM NaCl, 1 mM CaCl_2_, and 1% Triton X-100 supplemented with a protease inhibitor cocktail (Roche). Cell lysates were then adjusted to contain 10 mM Tris-HCl pH 8.0, 650 mM NaCl, 1 mM CaCl_2_, 10 mM imidazole, and 0.1% Triton X-100 and filtered through a 0.45 μm filter to remove any remaining debris. The sample was applied to 500 μl of preequilibrated Ni-NTA agarose (50% slurry) (Fujifilm Wako Pure Chemical Corporation). After washing with 10 mM Tris-HCl pH 8.0 containing 10 mM imidazole and 500 mM NaCl, the His-tagged proteins were eluted with 10 mM Tris-HCl pH 8.0 containing 150 mM NaCl and 250 mM imidazole. The eluate was then dialyzed twice against PBS for 4 h at 4 °C. The sample was stored at −80 °C until further use. The purified samples were analyzed by Western blotting using an anti-Myc antibody (Developmental Studies Hybridoma Bank [DSHB], University of Iowa, clone ID: 9E10, 1:2000 dilution) and GelCode blue stain (Thermo Fisher Scientific).

### Hypotonic cell lysis and in-solution sample preparation for LC-MS/MS

HEK293T cells were seeded in 10-cm culture dishes and transiently transfected with pSecTag2c-mouse CRB2 EGF1-8-MycHis using PEI-Max. After 4 to 5 h, the transfection medium was replaced with fresh DMEM supplemented with 10% FCS, and the cells were cultured for an additional 24 h. Cells were harvested, resuspended in hypotonic lysis buffer (20 mM Hepes pH 7.4, 1.5 mM MgCl_2_, 10 mM KCl) containing a protease inhibitor cocktail (Roche) on ice for 5 min, then sonicated and homogenized using a BioMasherSP homogenizer (Nippi). The lysates were clarified by centrifugation, and mouse CRB2 EGF1-8-MycHis was immunoprecipitated using an anti-Myc antibody (DSHB, clone ID: 9E10) coupled to Dynabeads Protein G (Thermo Fisher Scientific). After extensive washing with HBS (20 mM Hepes, 150 mM NaCl, pH 7.4), bound proteins were eluted with 8 M urea for 10 min at 65 °C. Following magnetic separation to remove the Dynabeads Protein G, the eluates were collected for subsequent sample preparation.

The eluted proteins were reduced with 10 mM Bond-Breaker TCEP Solution (Thermo Fisher Scientific) for 30 min at 37 °C and subsequently alkylated with 20 mM iodoacetamide for 30 min at RT in the dark. The urea concentration was diluted to below 2 M with ultra purewater prior to digestion with trypsin (Trypsin Gold; Promega) at an enzyme-to-substrate ratio of 1:50 (w/w). Proteins were digested overnight at 37 °C. Digestion was terminated by acidification with formic acid to a final concentration of 0.1%. The resulting peptides were desalted using ZipTip C18 pipette tips (MilliporeSigma) according to the manufacturer's instructions, dried in a vacuum centrifuge, and stored at −20 °C until LC-MS/MS analysis.

### Mass spectrometric analysis

Purified MycHis-tagged CRB2 and Notch proteins were reduced/alkylated using 10 mM Bond-Breaker TCEP Solution (Thermo Fisher Scientific) and iodoacetamide, separated on 10% SDS-PAGE, and visualized by GelCode blue staining (Thermo Fisher Scientific). The stained bands were excised, followed by in-gel protease digestion using trypsin (Trypsin Gold; Promega) in 20 mM diammonium phosphate pH 8.0 at 37 °C for 2 to 5 h, chymotrypsin (Roche, Basel, Switzerland) in 20 mM ammonium bicarbonate pH 7.8 at 37 °C for 2 to 5 h, or V8 (Glu-C; New England BioLabs) in 200 mM sodium phosphate pH 7.4 at 37 °C for 5 h. For the analysis of EGF2, EGF3, EGF4, EGF7, and EGF8 of CRB2, the sample was subjected to in-gel protease digestion with chymotrypsin, followed by acetone precipitation and then in-solution protease digestion with rAsp-N (rAsp-N; Promega) in 50 mM Tris-HCl pH 8.0 at 37 °C for 2 h. *N*-Glycan cleavage on the glycoproteins was performed using N-glycosidase F (Roche) in 50 mM Tris-HCl pH 8.0 containing 10 mM EDTA at 37 °C for 4 h. Sample pretreatment for the second set of samples regarding *GXYLT1* KO with overexpression of *GXYLT1* D312A (Fig. 7) and the samples of NOTCH1 and NOTCH2 (Fig. 8) was performed using a different method from that described previously. Proteins were reduced with 90 mM tris-(2-carboxyethyl) phosphine for 5 min at 50 °C and alkylated with 100 mM iodoacetamide for 30 min at RT in the dark. Protein digestion was performed using trypsin (Trypsin Gold; Promega) in 20 mM diammonium phosphate pH 8.0 at 37 °C for 2 h (42).

The resulting products were purified and concentrated using Zip-Tip C18 (Millipore) and injected into Orbitrap Fusion LC-MS (Thermo Fisher Scientific) equipped with an Ultimate3000 RSLCnano LC system (Dionex, Thermo Fisher Scientific) with a nano HPLC capillary column, 150 mm × 75 μm reverse phase column (Nikkyo Technos) as previously described (43). The MS spectra were recorded by an Orbitrap mass analyzer as described (42). For the detection of Endo H-sensitive peptides, precursor ions at particular m/z (928.3877, 982.4058, 1026.4198, 1070.4339, 996.0813, 1050.0989, 1094.1129, and 1138.1270) corresponding to the third isotopic peaks of the peptide resulting from tryptic digestion of CRB2: 224-CEQEVLECASAPCAHNASCLDGFR-247 with/without HexNAc modification in combination with Hex, HexPen, or HexPenPen modification were isolated for HCD-MS/MS fragmentation.

### Analysis of mass spectrometric data

The mass spectral data were processed using the PMI-Byonic software (Protein Metrics) as previously described (21, 42, 43, 44). For the Byonic software search, protein database files were generated from mouse CRB2 (Uniprot ID: Q80YA8), mouse NOTCH1 (Uniprot ID: Q01705), and mouse NOTCH2 (Uniprot ID: O35516) amino acid sequences obtained from Uniprot. Byonic (glyco)peptide search was conducted with the following instrument parameters. Precursor ion tolerance: 20 ppm, fragment ion tolerance: 0.5 Da, fragmentation type: QTOF/HCD. Cleavage sites were determined as follows, for tryptic digestion: C-terminal of K and R, for chymotryptic digestion: C-terminal of F, Y, and W, for V8 digestion: C-terminal of D and E, and for Asp-N digestion: N-terminal of D. Two missed cleavages were allowed. Carbamidomethylation on C was set as a fixed modification, oxidation on D, M, and N, deamidation on N-terminal of F, N, Q, and R, ammonia-loss of N-terminal C, N-terminal S, and N-terminal T were set as common variable modifications. Hex(1), Hex(1)Pen(1), Hex(1)Pen(2), Hex(2), Hex(2)NeuAc(1), Hex(2)Fuc(1), Pen(1), Pen(2), Pen(3), Fuc(1), HexNAc(1)Fuc(1), HexNAc(1)Hex(1)Fuc(1), HexNAc(1)Hex(1)Fuc(1)NeuAc(1), HexNAc(1), HexNAc(1)Hex(1), HexNAc(1)Hex(1)NeuAc(1) were set as rare variable glycan modifications. Two common modifications and two rare modifications were allowed for each peptide. (Glyco)peptides with Byonic scores >200 were analyzed. Correct assignments of MS/MS spectra were manually inspected using Xcalibur Qual browser v 4.1 (Thermo Fisher Scientific) by confirming major b/y ions and neutral loss ions. Lists of (glyco)peptides and their MS/MS spectra of each (glyco)peptide were provided in Tables S2, S6, and S10. EICs of all the identified (glyco)peptides were generated by selecting the higher peak of the monoisotopic or second isotopic ions, with 5 points Gaussian smoothing, and mass tolerance was set to 10 ppm. For semiquantification of (glyco)peptides, heights of EICs were normalized with isotopic abundance ratio (44).

### Endo H/PNGase F digestion of glycoprotein

Purified samples derived from culture media and cells were denatured with 0.5% SDS and 40 mM dithiothreitol at 95 °C for 5 min. For PNGase F digestion, 1/10 volume of 0.5 M sodium phosphate pH 7.5 and 10% NP-40 and 1/20 volume of PNGase F were added. For Endo H digestion, 1/10 volume of 10 × Endo H buffer and 1/20 volume of Endo H were added. Samples were incubated at 37 °C overnight. Digested samples were analyzed by western blotting using an anti-Myc (9E10) antibody.

### Immunofluorescence staining

COS-7 cells were transiently transfected with pSecTag2c-mouse CRB2 EGF1-8-MycHis using PEI-Max. After 4 to 5 h, the culture medium was replaced with fresh medium, and the cells were cultured overnight. The cells were detached and reseeded onto 10-well glass-bottom CELLview cell culture slides with an Advanced TC surface (Greiner Bio-One). After an additional 48 h of culture, the cells were fixed with 100% methanol at RT. For colocalization analysis of CRB2 with the ER marker calnexin or the *cis*-Golgi marker GM130, the cells were incubated for 1 h at RT with mouse anti-Myc antibody (DSHB, clone ID: 9E10), together with either rabbit anti-calnexin antibody (Medical & Biological Laboratories Co., Ltd.) or rabbit anti-GM130 antibody (Medical & Biological Laboratories Co., Ltd.), each diluted 1:250 in PBS. After washing with PBS, the cells were incubated for 1 h at RT with Alexa Fluor 555-conjugated goat anti-mouse IgG (Invitrogen), FITC-conjugated donkey anti-rabbit IgG (BioLegend, Poly4064), each diluted 1:500 in PBS, and DAPI (Dojindo Laboratories) at a dilution of 1:1000 in PBS. Images were acquired using an all-in-one fluorescence microscope (BZ-X710, KEYENCE) equipped with a 100× oil-immersion Plan Apochromat objective lens (NA 1.45), and image processing was performed using BZ-X Analyzer software (version 1.4.1.1, KEYENCE). Colocalization analyses of HA-tagged GXYLT1 and GXYLT2 using pcDNA3.1-human GXYLT1-HA and pcDNA3.1-human GXYLT2-HA were performed as described previously, except that mouse anti-HA antibody (ABclonal, clone ID: AMC0503) diluted 1:100 in PBS was used instead of the anti-Myc antibody.

### Secretion assays

The plasmids used for the secretion assays were pSecTag2c-mouse CRB2 EGF1-15-MycHis, pSecTag2c-mouse NOTCH1 EGF1-36-MycHis, or pSecTag2c-mouse NOTCH2 EGF1-36-MycHis. The empty pSecTag2c vector was used as a negative control. A plasmid encoding human IgG was used as a secretion control. HEK293T cells were seeded in 6-well dishes in DMEM supplemented with 5% FCS. The cells were transiently transfected with 1 μg/well pSecTag2c-mouse CRB2 EGF1-15-MycHis, pSecTag2c-mouse NOTCH1 EGF1-36-MycHis, NOTCH2 EGF1-36-MycHis, or an empty vector, and 0.05 μg/well IgG plasmid using PEI-Max. Expression plasmids of WT GXYLT1, its DQD mutants, and WT GXYLT2 for rescue experiments were used in the same amount (1 μg/well) as the expression plasmids of the ECDs of CRB2, NOTCH1, or NOTCH2. After 4 to 5 h, the medium was changed to 1 ml of OPTI-MEM I. The cells were cultured for another 3 days. Culture media samples and cell lysates were analyzed by SDS-PAGE and subsequent western blotting analysis as described below.

### Western blotting analysis

Membranes were incubated overnight with mouse anti-Myc antibody (DSHB, clone ID: 9E10) at a 1:2000 dilution in blocking solution. Secondary antibodies, anti-mouse IgG, HRP-Linked F(ab’)_2_ Fragment Sheep (GE Healthcare), and Goat Anti-Human IgG-Fc Fragment Antibody HRP Conjugated (BETHYL), were incubated with the membrane at dilutions of 1:10,000 and 1:500,000, respectively, for 1 h at RT. The bands were visualized using the iBright Imaging System FL1500 (Thermo Fisher Scientific).

### Animal breeding

The following strains were used: WT C57BL/6, *Gxylt1*^*+/−*^ (this study), *Gxylt2*^*+/−*^ (this study). Both mutant strains were generated and analyzed on a C57BL/6 background. The mice were kept in a pathogen-free barrier facility at Baylor College of Medicine. Mouse studies were performed in accordance with the guidelines of the Institutional Animal Care and Use Committee of Baylor College of Medicine and based on the approved animal protocol AN-6012.

### Generation of the Gxylt1 and Gxylt2 loss of function alleles and genotyping

The *Gxylt1*-mutant allele was generated by injecting CAS9 RNA and two guide (sgRNA) sequences TTCTCGGGAGGGTTGCCTCGAGG (intron 1) and GCTACCTGAGGATCTAGGTGCGG (intron 3), which resulted in a 3352-bp deletion encompassing exons 2 and 3. The *Gxylt2*-mutant allele was generated by injecting CAS9 RNA and two guide sequences TCATCCTCGGGTAACAGGATGGG (intron 3) and CTGCCTCCGCAGATGCCCATGGG (intron 5), resulting in a 4520-bp deletion encompassing exons 4 and 5. None of the sgRNAs had a predicted off-target with one or two mismatches. For each sgRNA, the top four predicted off-targets (three mismatches) were confirmed to be unaffected by Sanger sequencing. For each gene, founders were backcrossed to WT animals for two generations, and a single male progeny from the second generation crosses was used to establish the stock.

Genomic DNA from tails or yolk sacs was used for genotyping. For each allele, a three-primer PCR genotyping strategy was used, with a shared forward primer (P1) and reverse primers unique to WT (P2) and CRISPR-Cas9-mediated deletion (P3) alleles. The following primers were used: Gxylt1-P1: 5′- AGTGGTCTGATTTTGGGTTGA-3′, Gxylt1-P2: 5′-TCTCACCACAGGCAACAACA-3′, Gxylt1-P3: 5′-TCAGAAACTCTTTTGTGGTGGT-3′, Gxylt2-P1: 5′-CAGAGGGAAAGGCAAGTGGT-3′, Gxylt2-P2: 5′- GCTTCCAGATGTCGTCCACA-3′, Gxylt2-P3: 5′-TGAGACGACAGTAGAGGCCT-3′.

### Embryonic studies

Embryos were collected at 9.5 to 10 days post conception, were fixed in 4% paraformaldehyde in PBS, washed several times in PBS, and imaged using an Olympus SZX9 stereomicroscope, brightfield lighting, SPOT Insight CMOS camera, and SPOT Imaging software version 5.3. Images were processed with Adobe Photoshop and assembled with Adobe Illustrator.

### CRL measurement

CRL was measured using ImageJ software. The maximum total length from the cephalic pole to the caudal pole was measured from a longitudinal view, and two measurements were averaged for each embryo.

### Statistical analysis

An overall comparison of group means was conducted using one-way ANOVA, followed by Tukey’s *post hoc* test for pairwise comparisons to analyze the results of the secretion assay. ∗∗∗*p* < 0.001, ∗∗*p* < 0.01, ∗*p* < 0.05, not significant (n.s.) *p* ≥ 0.05. An unpaired one-tailed *t* test was performed to analyze the qRT-PCR results. One-way ANOVA followed by Dunnett’s multiple comparisons test was performed to analyze the CRLs. A Chi-square test was performed to analyze animal survival. Statistical analyses were performed using GraphPad Prism.

## Data availability

The glycoproteome data in this study were deposited in jPOSTrepo: an international standard data repository for proteomes (45) (Project ID: JPST003944, PXD066404, JPST004879).

## Supporting information

This article contains supporting information.

## Conflict of interest

One of the corresponding authors (H. T.) is an Editorial Board Member of JBC. The authors declare that they have no other conflicts of interest with the content of this article.

## Supporting information

Supplemenry Tables
