# Supplementary material for: ER quality control of Crumbs2 and Notch proteins by GXYLT1-dependent xylosyl elongation of O-glucose glycans
Source: J Biol Chem. 2026 Sep 15;302(11):113556. doi: 10.1016/j.jbc.2026.113556 (PMC13683433; doi:10.1016/j.jbc.2026.113556)
Supplement: Supplementary file 1 [file mmc2.pdf]

ER quality control of Crumbs2 and Notch proteins by GXYLT1-dependent xylosyl elongation of *O*-glucose glycans

Yukihiro Mita[1,2], Soomin Cho[3,4], Yohei Tsukamoto[1,5], Wataru Saiki[1], Mio Okamura[5], Yuki Fujita[5], Nima Niknejad[3], Daichi Funada[5], Fuga Suzuki[5], Kensuke Ohkawa[5], Taiki Jo[5], Yusuke Urata[2], Yuuki Kurebayashi[5], Akira Minami[5,6], Tadanobu Takahashi[5], Hideharu Hibi[2], Tetsuya Okajima[1,7,*], Hamed Jafar-Nejad[3,4], and Hideyuki Takeuchi[1,5,*]

[1]Department of Molecular and Cellular Biology, Nagoya University Graduate School of Medicine. Nagoya, Japan

[2]Department of Oral and Maxillofacial Surgery, Nagoya University Graduate School of Medicine, Nagoya, Japan

[3]Department of Molecular & Human Genetics, Baylor College of Medicine. Houston, USA

[4]Development, Disease Models & Therapeutics Graduate Program, Baylor College of Medicine. Houston, USA

[5]Department of Biochemistry, School of Pharmaceutical Sciences, University of Shizuoka. Shizuoka, Japan.

[6] Department of Functional Morphology, Faculty of Pharmacy, Juntendo University, Chiba, Japan

[7]Institute for Glyco-core Research (iGCORE), Nagoya University. Nagoya, Japan

***Correspondence:**

Tetsuya Okajima, Email: tokajima@med.nagoya-u.ac.jp;

Hideyuki Takeuchi, Email: htakeuchi@u-shizuoka-ken.ac.jp

Figure S1-1

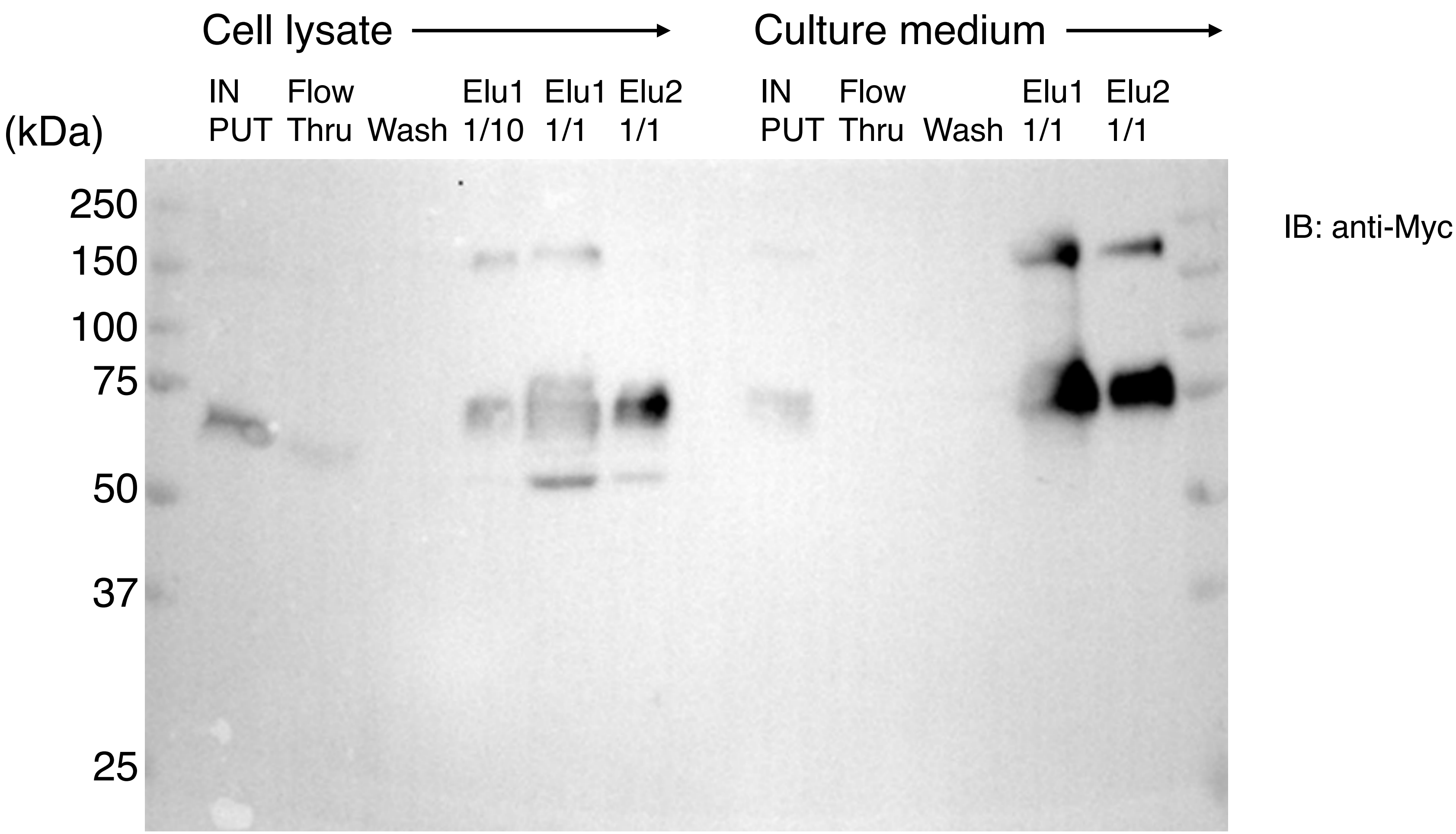

Cell lysate
Culture medium
IN PUT
Flow Thru
Wash
Elu1 1/10
Elu1 1/1
Elu2 1/1
IN PUT
Flow Thru
Wash
Elu1 1/1
Elu2 1/1
(kDa)
250
150
100
75
50
37
25
IB: anti-Myc

Figure S1-2

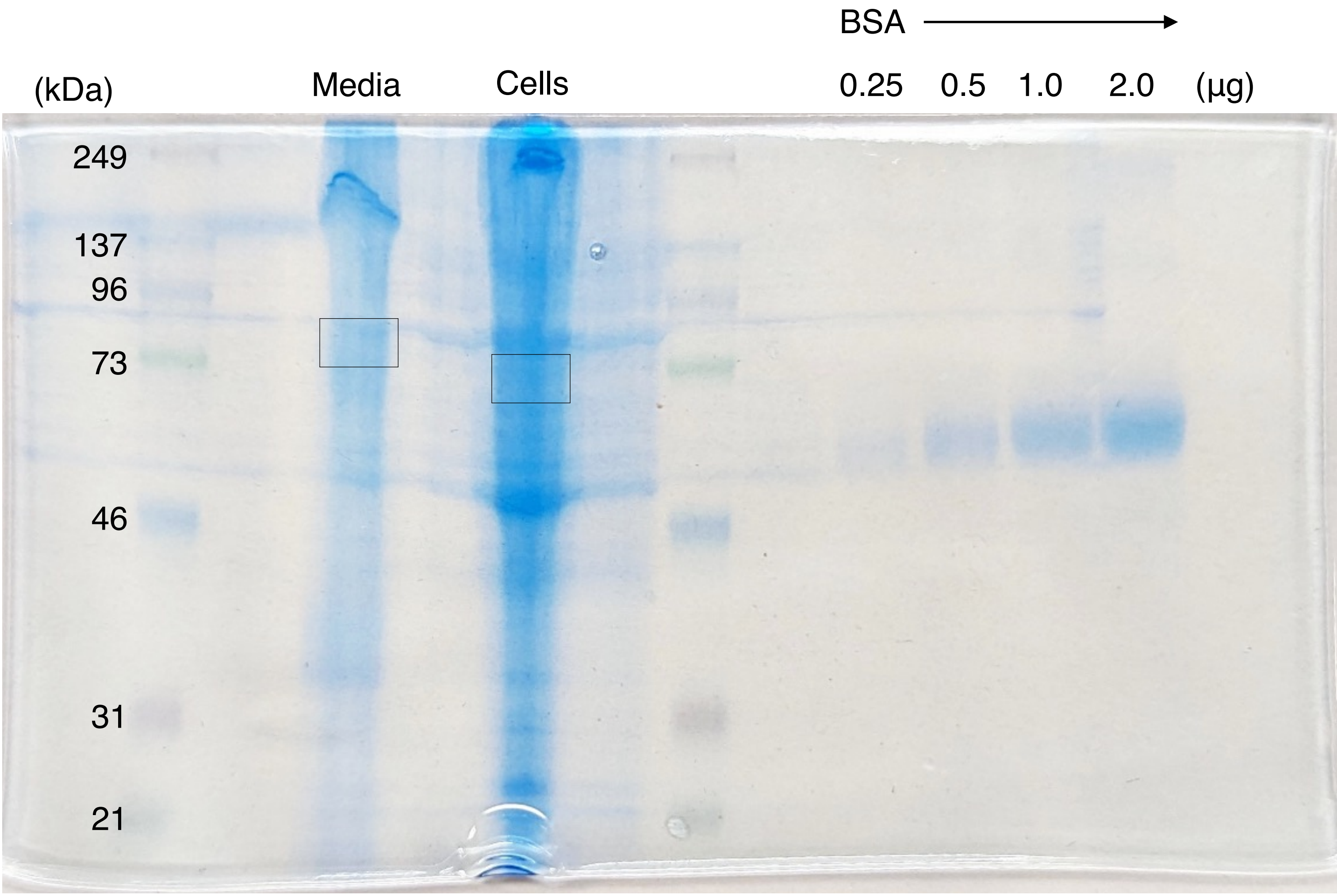

Figure S1-3

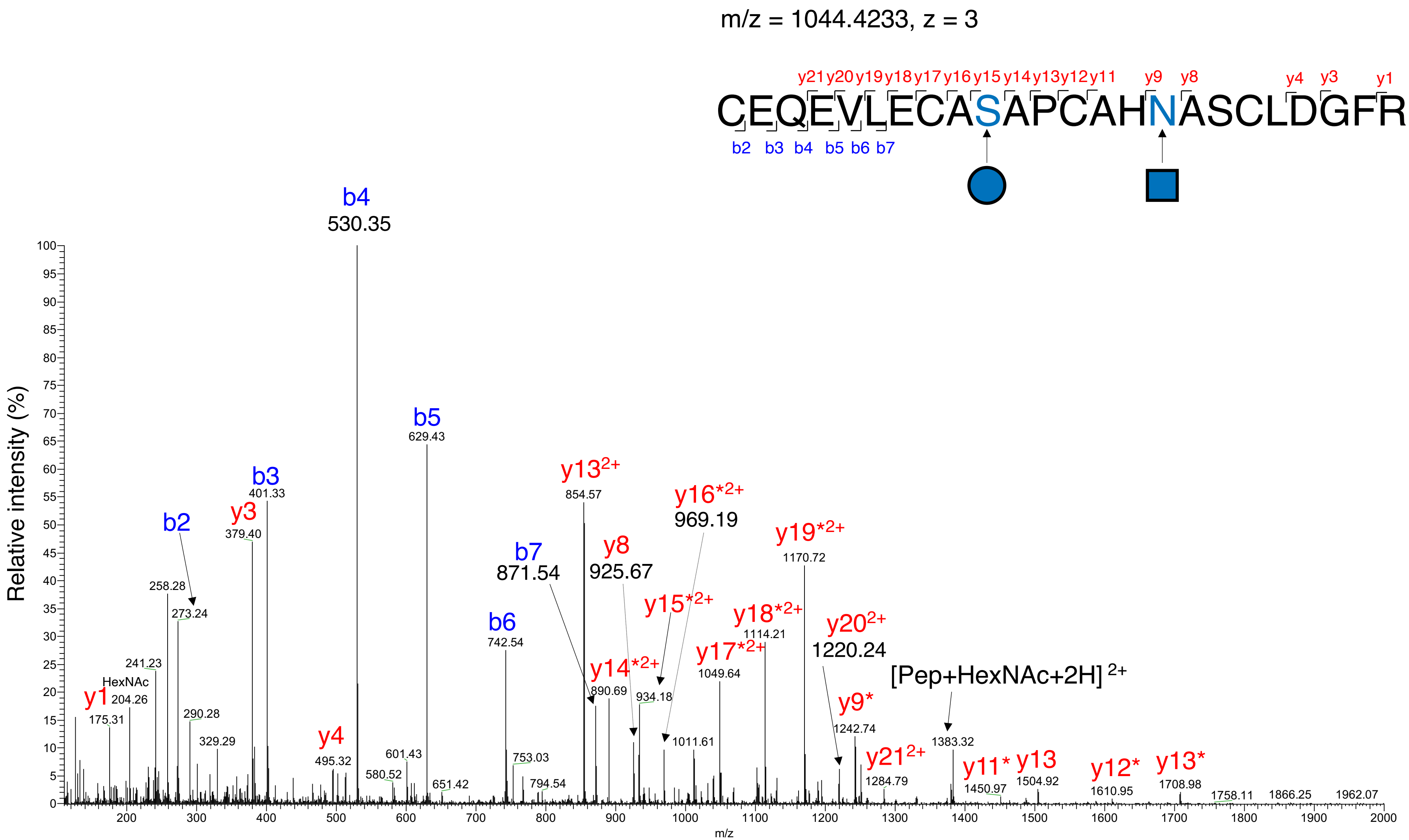

m/z = 1044.4233, z = 3
y21 y20 y19 y18 y17 y16 y15 y14 y13 y12 y11 y9 y8 y4 y3 y1
CEQEVLECASAPCAHNASCLDGFR
b2 b3 b4 b5 b6 b7
b4 530.35
b5 629.43
b3 401.33
y3 379.40
b2 273.24
258.28
241.23
HexNAc 204.26
y1 175.31
290.28
329.29
y4 495.32
580.52
601.43
651.42
b6 742.54
753.03
794.54
b7 871.54
y13^2+ 854.57
y8 925.67
y14*2+ 890.69
y15*2+ 934.18
y16*2+ 969.19
1011.61
y17*2+ 1049.64
y18*2+ 1114.21
y19*2+ 1170.72
y20^2+ 1220.24
y9* 1242.74
y21^2+ 1284.79
[Pep+HexNAc+2H] 2+ 1383.32
y11* 1450.97
y13 1504.92
y12* 1610.95
y13* 1708.98
1758.11
1866.25
1962.07
Relative intensity (%)
m/z

Figure S1-4

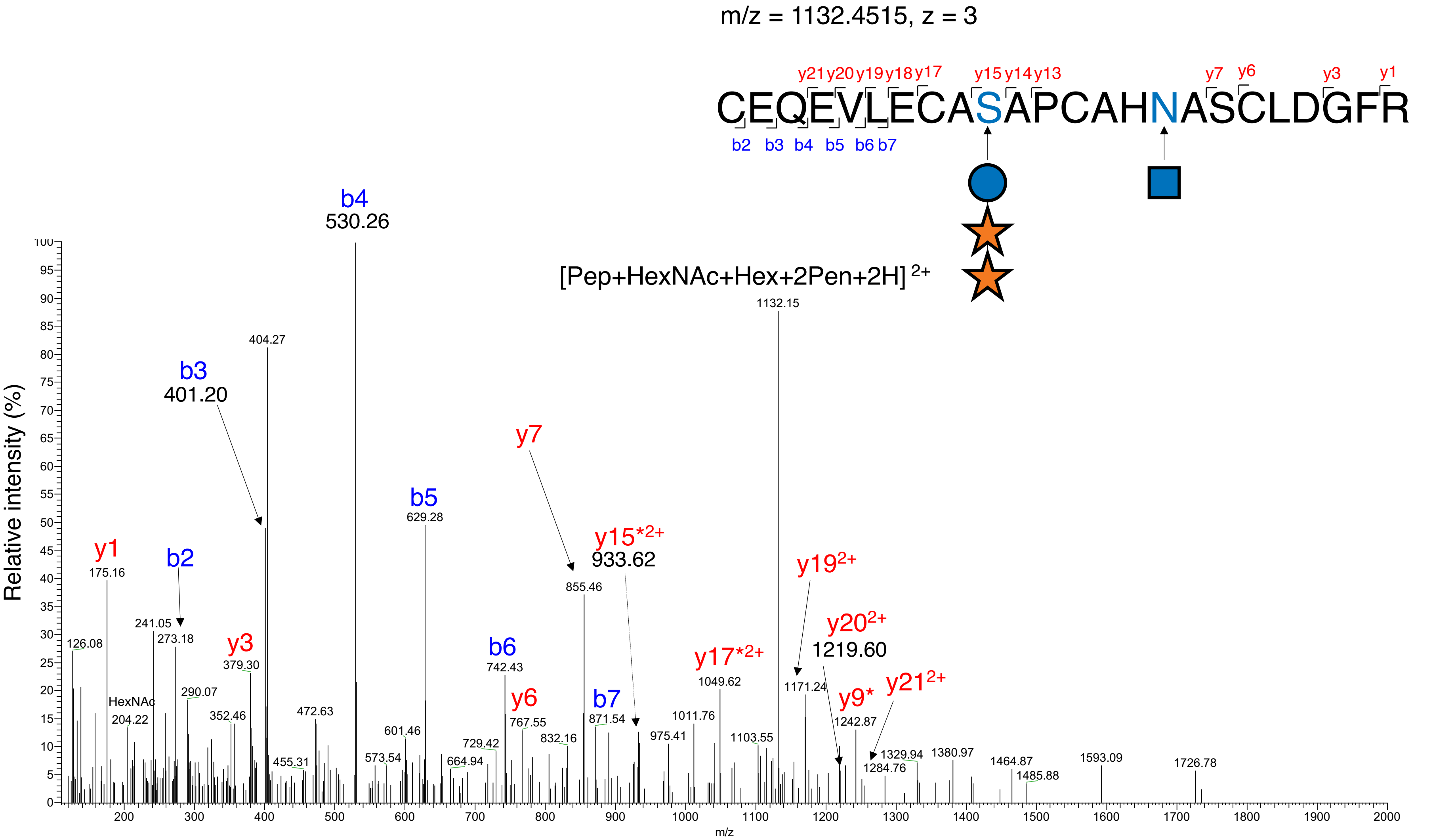
m/z = 1132.4515, z = 3
y21 y20 y19 y18 y17 y15 y14 y13 y7 y6 y3 y1
CEQEVLECASAPCAHNASCLDGFR
b2 b3 b4 b5 b6 b7
[Pep+HexNAc+Hex+2Pen+2H] 2+
1132.15
b4
530.26
404.27
b3
401.20
y7
855.46
b5
629.28
y15*2+
933.62
y1
175.16
b2
273.18
241.05
126.08
y3
379.30
290.07
HexNAc
204.22
352.46
472.63
455.31
601.46
573.54
b6
742.43
y6
767.55
b7
871.54
729.42
664.94
832.16
975.41
1011.76
y17*2+
1049.62
1103.55
y19 2+
1171.24
y20 2+
1219.60
y9*
1242.87
y21 2+
1284.76
1329.94
1380.97
1464.87
1485.88
1593.09
1726.78
Relative intensity (%)
m/z

Figure S1. Mass spectrometric analysis of CRB2 proteins purified from cell lysates.

(1-1) Western blot analysis using an anti-Myc antibody was performed to confirm the successful purification of CRB2 EGF1-8 from both cell lysates and culture media using Ni-NTA agarose. (1-2) 10% SDS-PAGE was performed to separate acetone-precipitated CRB2 proteins purified from culture media and cell lysates, using BSA as a standard. Protein bands were visualized with GelCode Blue Stain Reagent. The excised areas for tryptic digestion are indicated. (1-3, 4) MS/MS spectra of triply charged ions m/z 1044.4233 (3) or m/z 1132.4515 (4) corresponding to peptides with Glc or Glc-Xyl-Xyl modification in addition to HexNAc, respectively.

Figure S2-1

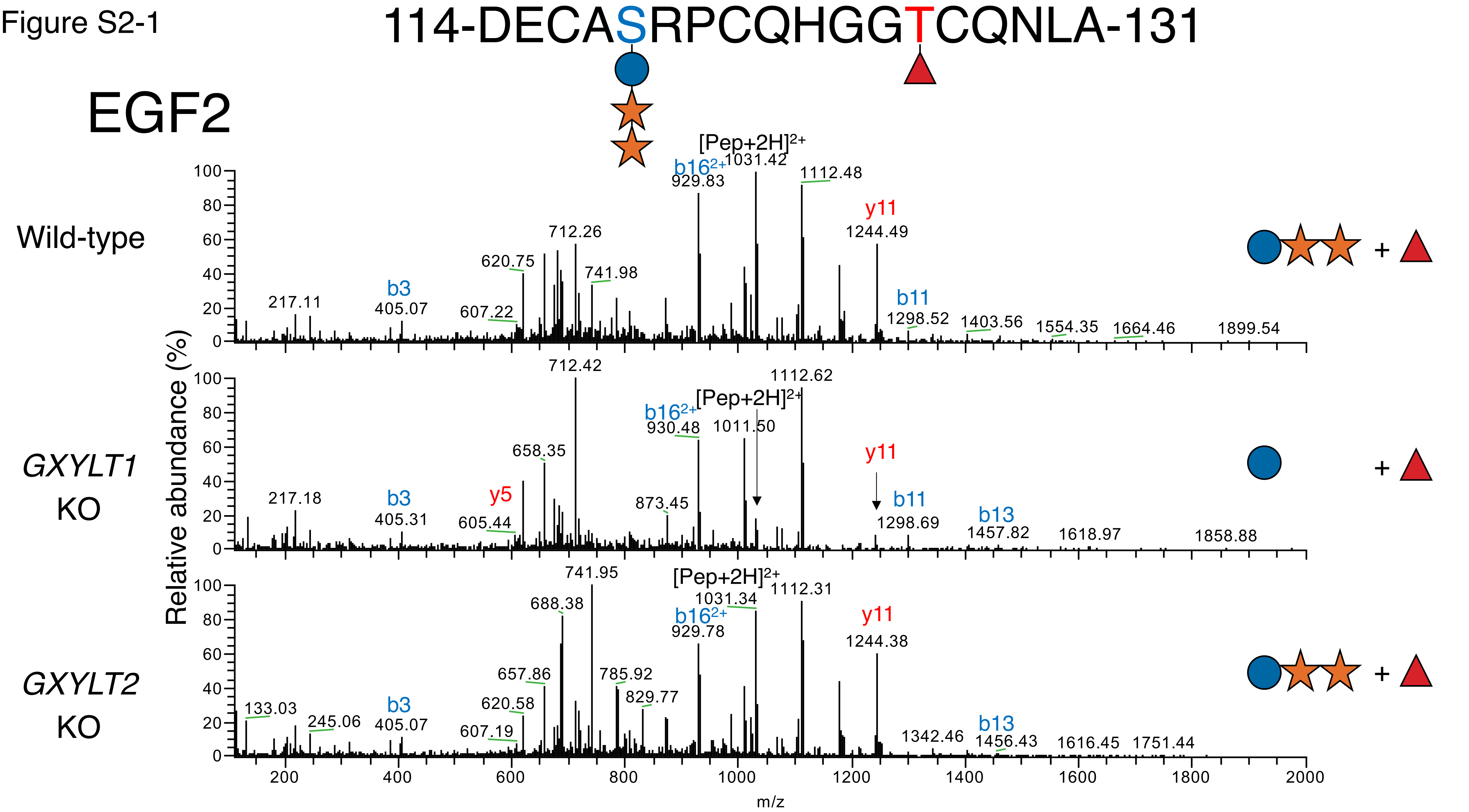

114-DECASRPCQHGGTCQNLA-131
EGF2
Wild-type
GXYLT1 KO
GXYLT2 KO
Relative abundance (%)
m/z
[Pep+2H]2+
b16 2+
1031.42
929.83
1112.48
y11
1244.49
712.26
620.75
741.98
b3
405.07
217.11
607.22
b11
1298.52
1403.56
1554.35
1664.46
1899.54
712.42
1112.62
930.48
1011.50
658.35
y5
605.44
405.31
217.18
873.45
1298.69
b13
1457.82
1618.97
1858.88
741.95
1031.34
1112.31
688.38
929.78
1244.38
657.86
785.92
829.77
620.58
133.03
245.06
607.19
1342.46
1456.43
1616.45
1751.44

Figure S2-2

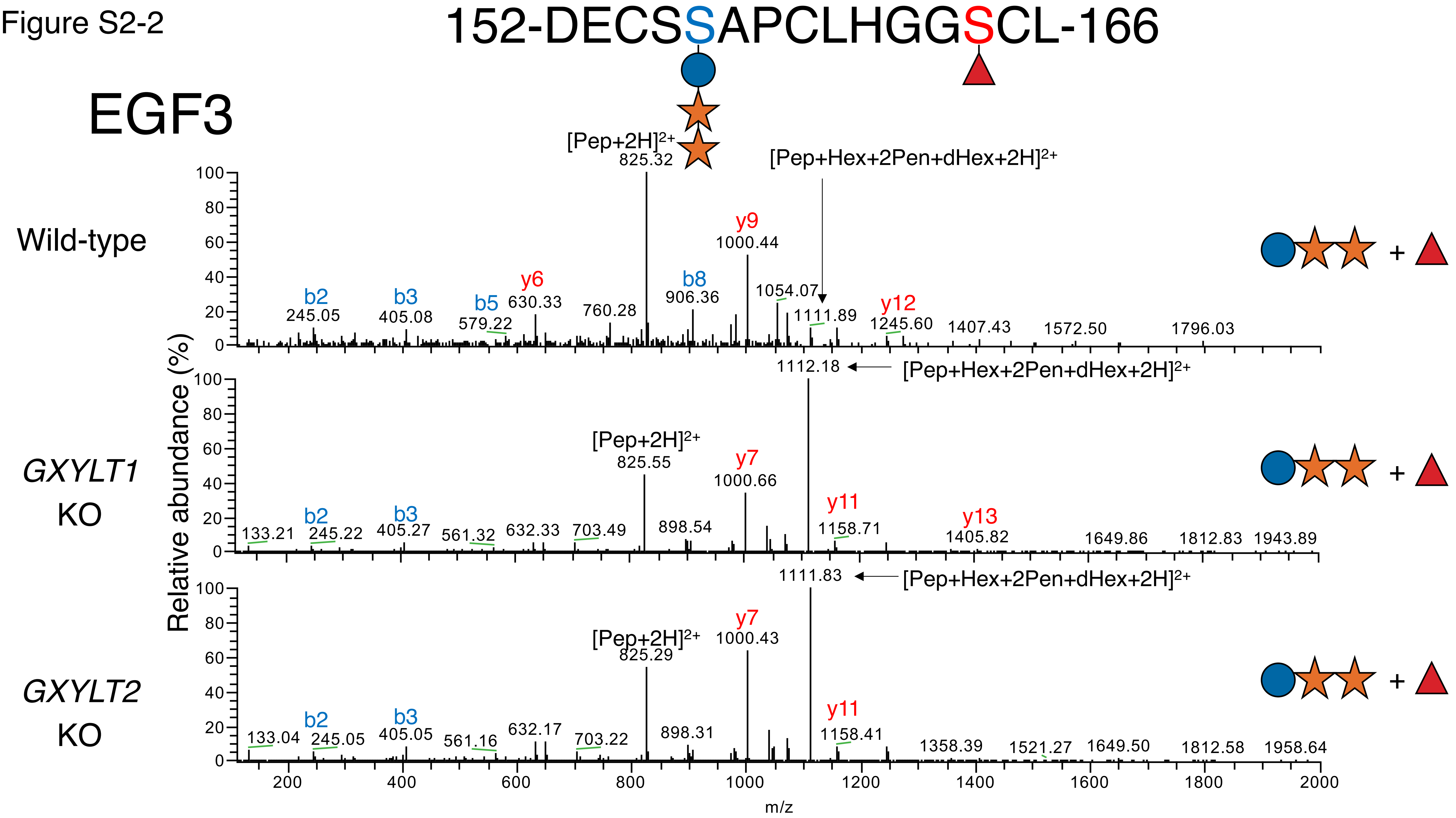
152-DECSSAPCLHGGSCL-166
EGF3
Wild-type
GXYLT1
KO
GXYLT2
KO
Relative abundance (%)
m/z
[Pep+2H]2+
825.32
[Pep+Hex+2Pen+dHex+2H]2+
b2
245.05
b3
405.08
b5
579.22
y6
630.33
760.28
b8
906.36
y9
1000.44
1054.07
1111.89
y12
1245.60
1407.43
1572.50
1796.03
1112.18
[Pep+Hex+2Pen+dHex+2H]2+
[Pep+2H]2+
825.55
y7
1000.66
133.21
b2
245.22
b3
405.27
561.32
632.33
703.49
898.54
y11
1158.71
y13
1405.82
1649.86
1812.83
1943.89
1111.83
[Pep+Hex+2Pen+dHex+2H]2+
[Pep+2H]2+
825.29
y7
1000.43
133.04
b2
245.05
b3
405.05
561.16
632.17
703.22
898.31
y11
1158.41
1358.39
1521.27
1649.50
1812.58
1958.64
+
0
20
40
60
80
100
200
400
600
800
1000
1200
1400
1600
1800
2000

Figure S2-3

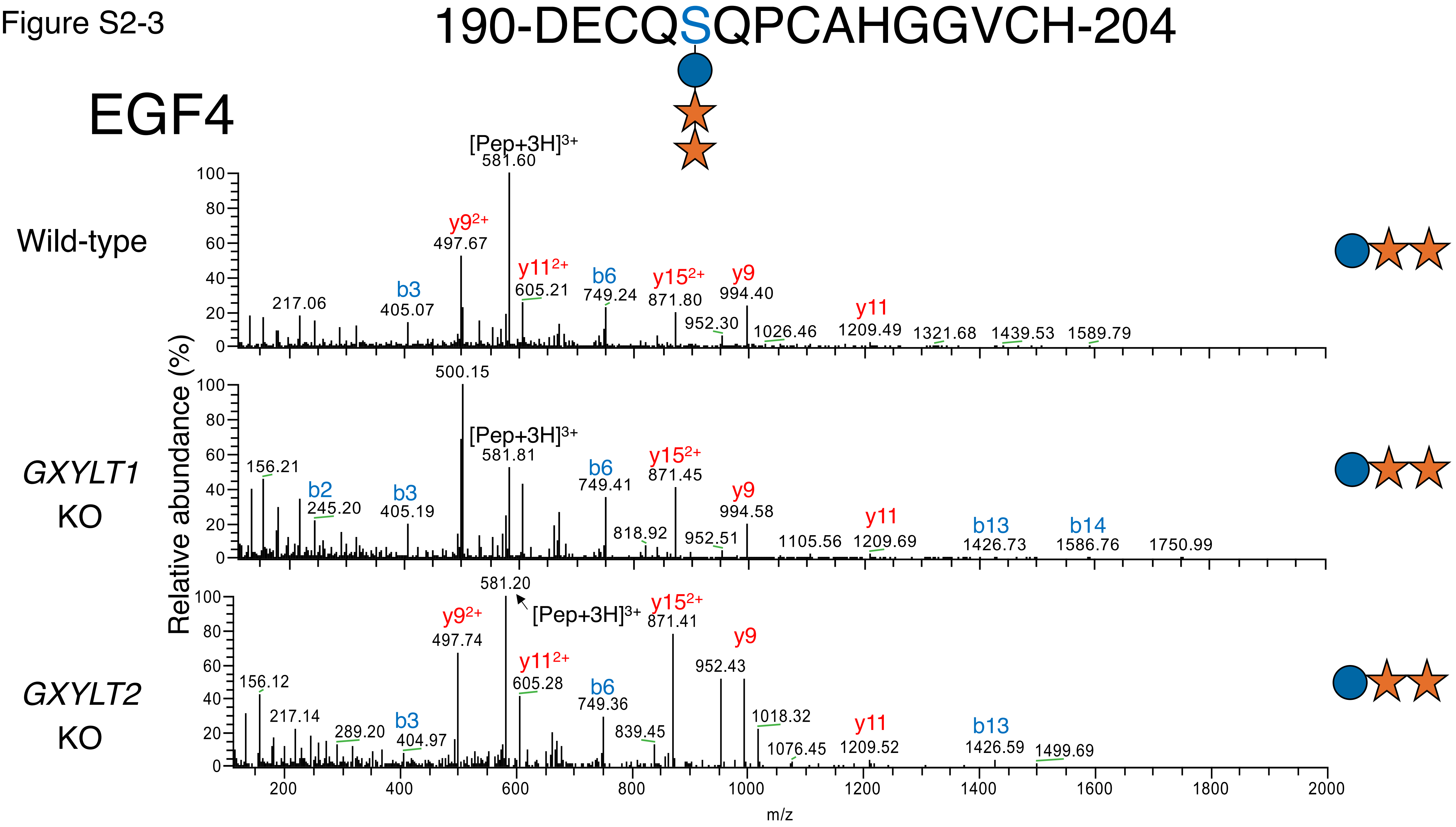
190-DECQSQPCAHGGVCH-204
EGF4
Wild-type
GXYLT1
KO
GXYLT2
KO
Relative abundance (%)
m/z
[Pep+3H]3+
581.60
y92+
497.67
y112+
605.21
b6
749.24
y152+
871.80
y9
994.40
217.06
b3
405.07
952.30
1026.46
y11
1209.49
1321.68
1439.53
1589.79
500.15
[Pep+3H]3+
581.81
156.21
b2
245.20
b3
405.19
b6
749.41
y152+
871.45
y9
994.58
818.92
952.51
1105.56
y11
1209.69
b13
1426.73
b14
1586.76
1750.99
581.20
[Pep+3H]3+
y92+
497.74
y152+
871.41
y9
952.43
y112+
605.28
156.12
217.14
289.20
b3
404.97
b6
749.36
839.45
1018.32
1076.45
y11
1209.52
b13
1426.59
1499.69
0
20
40
60
80
100
200
400
600
800
1000
1200
1400
1600
1800
2000

Figure S2-4

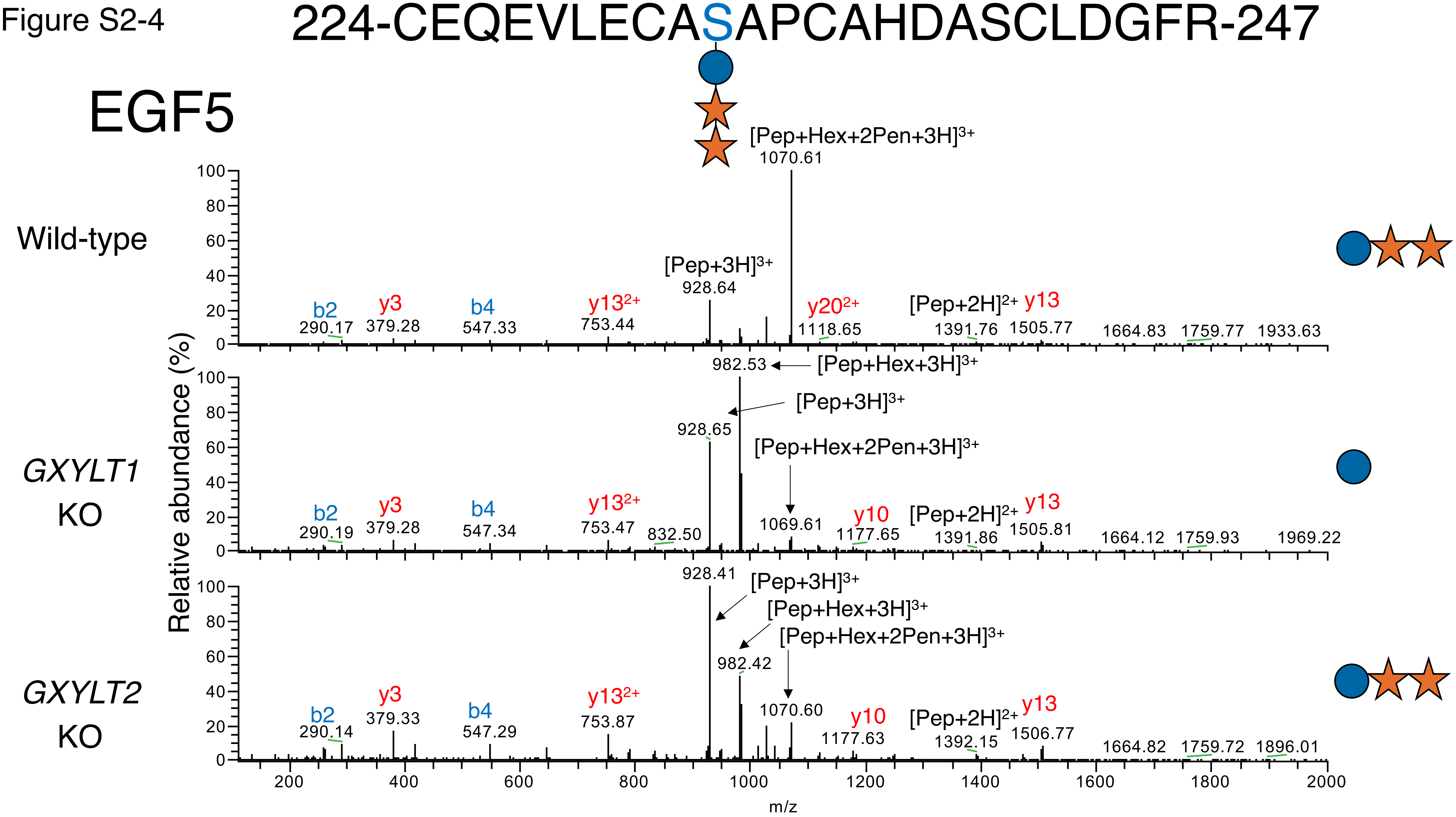

224-CEQEVLECASAPCAHDASCLDGFR-247
EGF5
Wild-type
GXYLT1 KO
GXYLT2 KO
Relative abundance (%)
m/z
[Pep+Hex+2Pen+3H]3+
1070.61
[Pep+3H]3+
928.64
b2
290.17
y3
379.28
b4
547.33
y13 2+
753.44
y20 2+
1118.65
[Pep+2H]2+
1391.76
y13
1505.77
1664.83
1759.77
1933.63
982.53
[Pep+Hex+3H]3+
[Pep+3H]3+
928.65
[Pep+Hex+2Pen+3H]3+
b2
290.19
y3
379.28
b4
547.34
y13 2+
753.47
832.50
1069.61
y10
1177.65
[Pep+2H]2+
1391.86
y13
1505.81
1664.12
1759.93
1969.22
928.41
[Pep+3H]3+
[Pep+Hex+3H]3+
[Pep+Hex+2Pen+3H]3+
982.42
1070.60
b2
290.14
y3
379.33
b4
547.29
y13 2+
753.87
y10
1177.63
[Pep+2H]2+
1392.15
y13
1506.77
1664.82
1759.72
1896.01

Figure S2-5

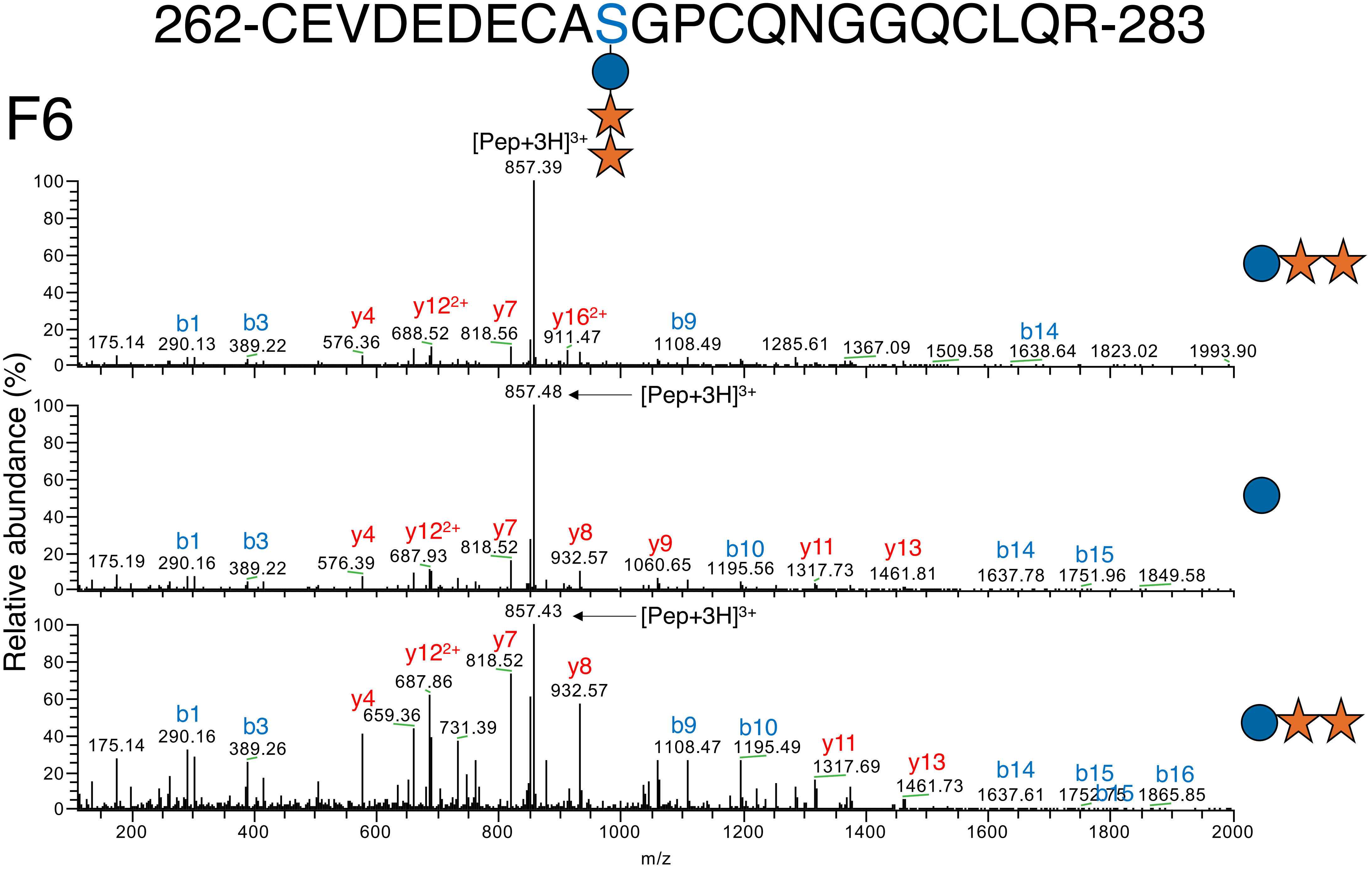
262-CEVDEDECASGPCQNGGQCLQR-283
EGF6
Wild-type
GXYLT1 KO
GXYLT2 KO
Relative abundance (%)
m/z
[Pep+3H]3+
857.39
857.48
857.43
b1
b3
b9
b10
b14
b15
b16
y4
y7
y8
y9
y11
y13
y12 2+
y16 2+
175.14
290.13
389.22
576.36
688.52
818.56
911.47
1108.49
1285.61
1367.09
1509.58
1638.64
1823.02
1993.90
175.19
290.16
576.39
687.93
818.52
932.57
1060.65
1195.56
1317.73
1461.81
1637.78
1751.96
1849.58
389.26
659.36
687.86
731.39
1108.47
1195.49
1317.69
1461.73
1637.61
1752.15
1865.85

Figure S2-6

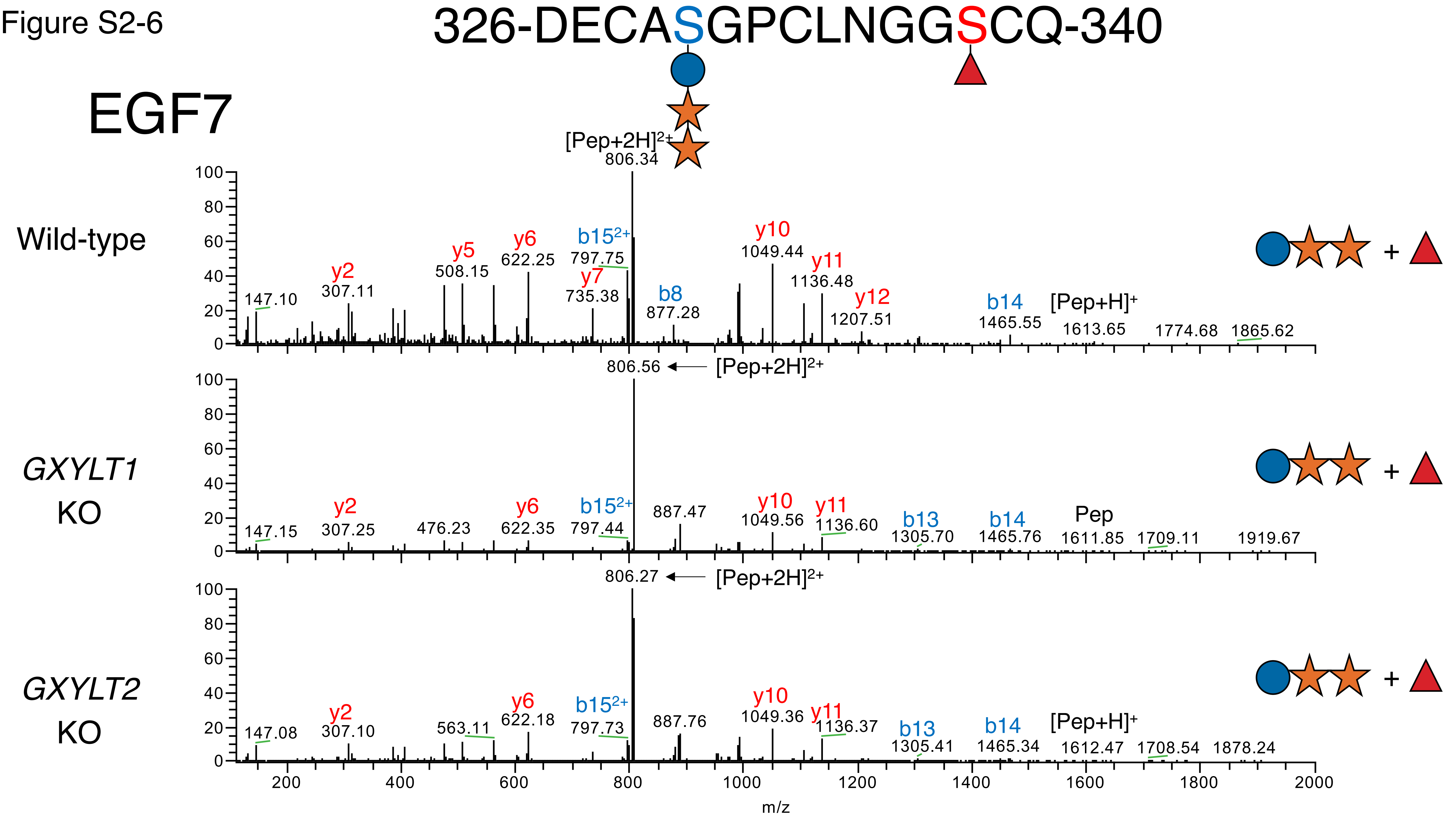

326-DECASGPCLNGGSCQ-340
EGF7
Wild-type
GXYLT1 KO
GXYLT2 KO
[Pep+2H]2+
806.34
147.10
y2
307.11
y5
508.15
y6
622.25
b15 2+
797.75
y7
735.38
b8
877.28
y10
1049.44
y11
1136.48
y12
1207.51
b14
1465.55
[Pep+H]+
1613.65
1774.68
1865.62
806.56
[Pep+2H]2+
147.15
y2
307.25
476.23
y6
622.35
b15 2+
797.44
887.47
y10
1049.56
y11
1136.60
b13
1305.70
b14
1465.76
Pep
1611.85
1709.11
1919.67
806.27
[Pep+2H]2+
147.08
y2
307.10
563.11
y6
622.18
b15 2+
797.73
887.76
y10
1049.36
y11
1136.37
b13
1305.41
b14
1465.34
[Pep+H]+
1612.47
1708.54
1878.24
m/z

Figure S2-7

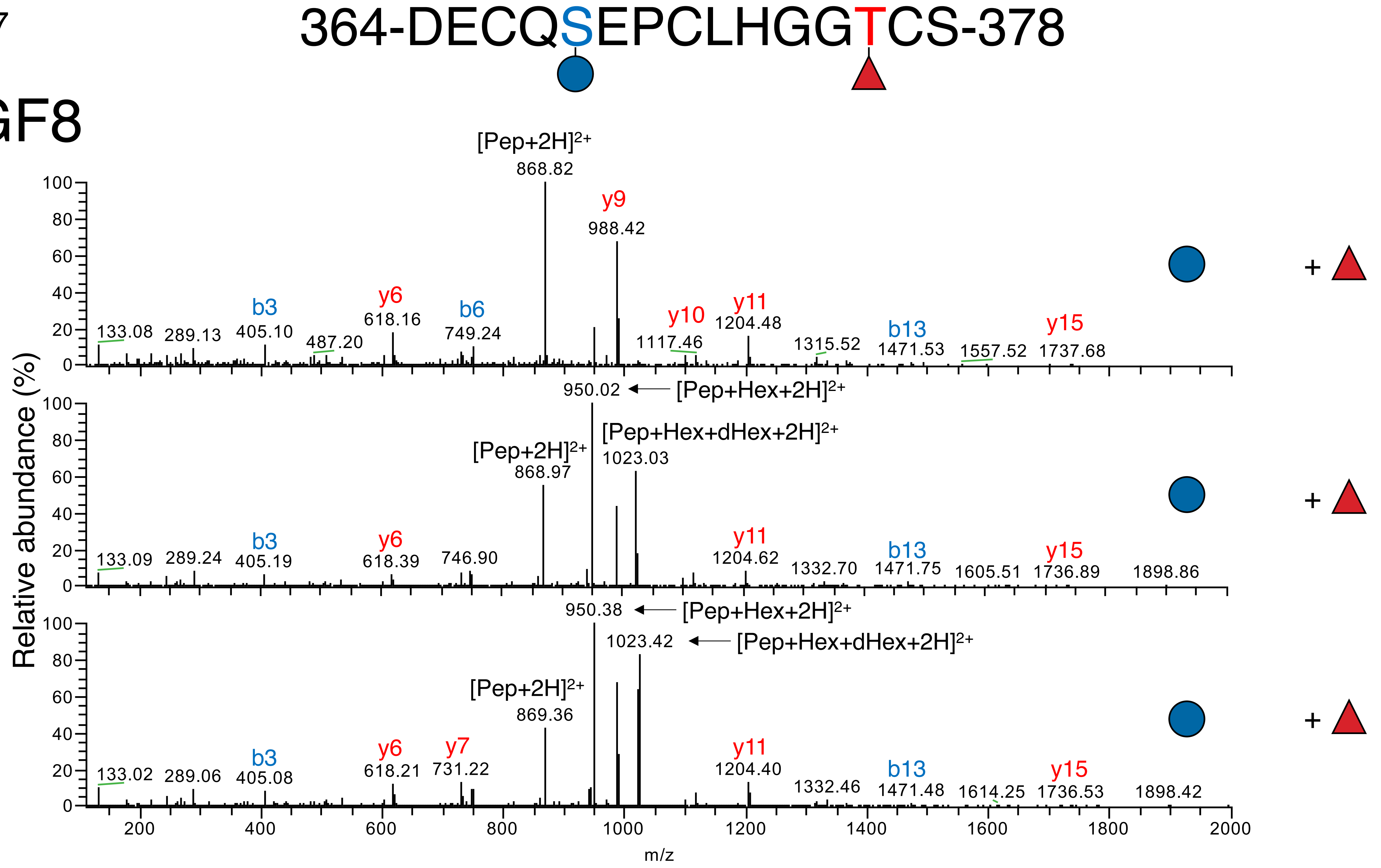

364-DECQSEPCLHGGTCS-378
EGF8
Wild-type
GXYLT1 KO
GXYLT2 KO
Relative abundance (%)
m/z
[Pep+2H]2+
868.82
y9
988.42
133.08
289.13
b3
405.10
487.20
y6
618.16
b6
749.24
y10
1117.46
y11
1204.48
1315.52
b13
1471.53
1557.52
y15
1737.68
950.02
[Pep+Hex+2H]2+
[Pep+Hex+dHex+2H]2+
1023.03
868.97
133.09
289.24
405.19
618.39
746.90
1204.62
1332.70
1471.75
1605.51
1736.89
1898.86
950.38
1023.42
869.36
133.02
289.06
405.08
618.21
y7
731.22
1204.40
1332.46
1471.48
1614.25
1736.53
1898.42

Figure S2-8

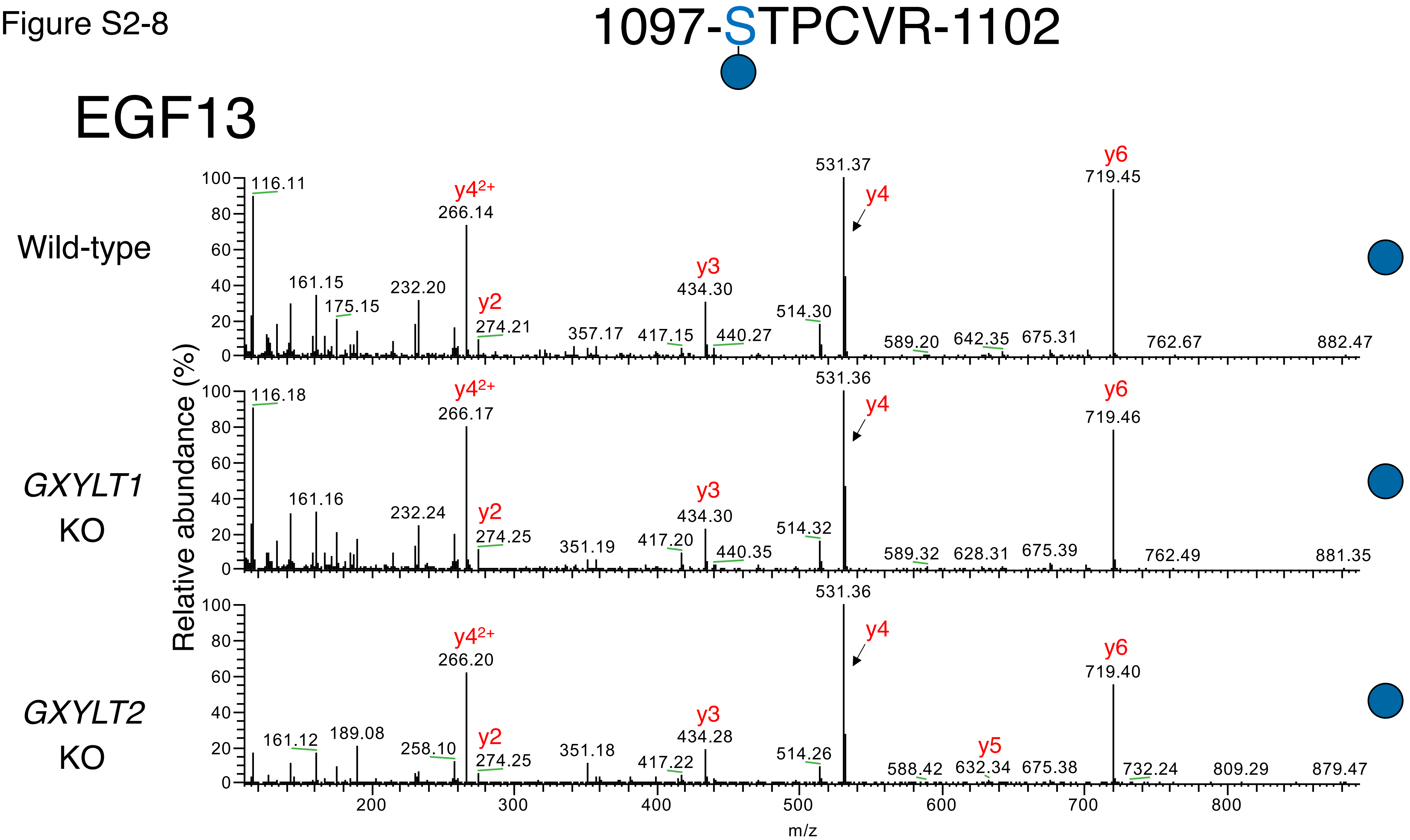

1097-STPCVR-1102
EGF13
Wild-type
GXYLT1 KO
GXYLT2 KO
Relative abundance (%)
m/z
116.11
y4²⁺
266.14
161.15
175.15
232.20
y2
274.21
357.17
417.15
y3
434.30
440.27
514.30
531.37
y4
589.20
642.35
675.31
y6
719.45
762.67
882.47
116.18
266.17
161.16
232.24
274.25
351.19
417.20
434.30
440.35
514.32
531.36
589.32
628.31
675.39
719.46
762.49
881.35
161.12
189.08
258.10
266.20
274.25
351.18
417.22
434.28
514.26
531.36
588.42
y5
632.34
675.38
719.40
732.24
809.29
879.47

Figure S2-9

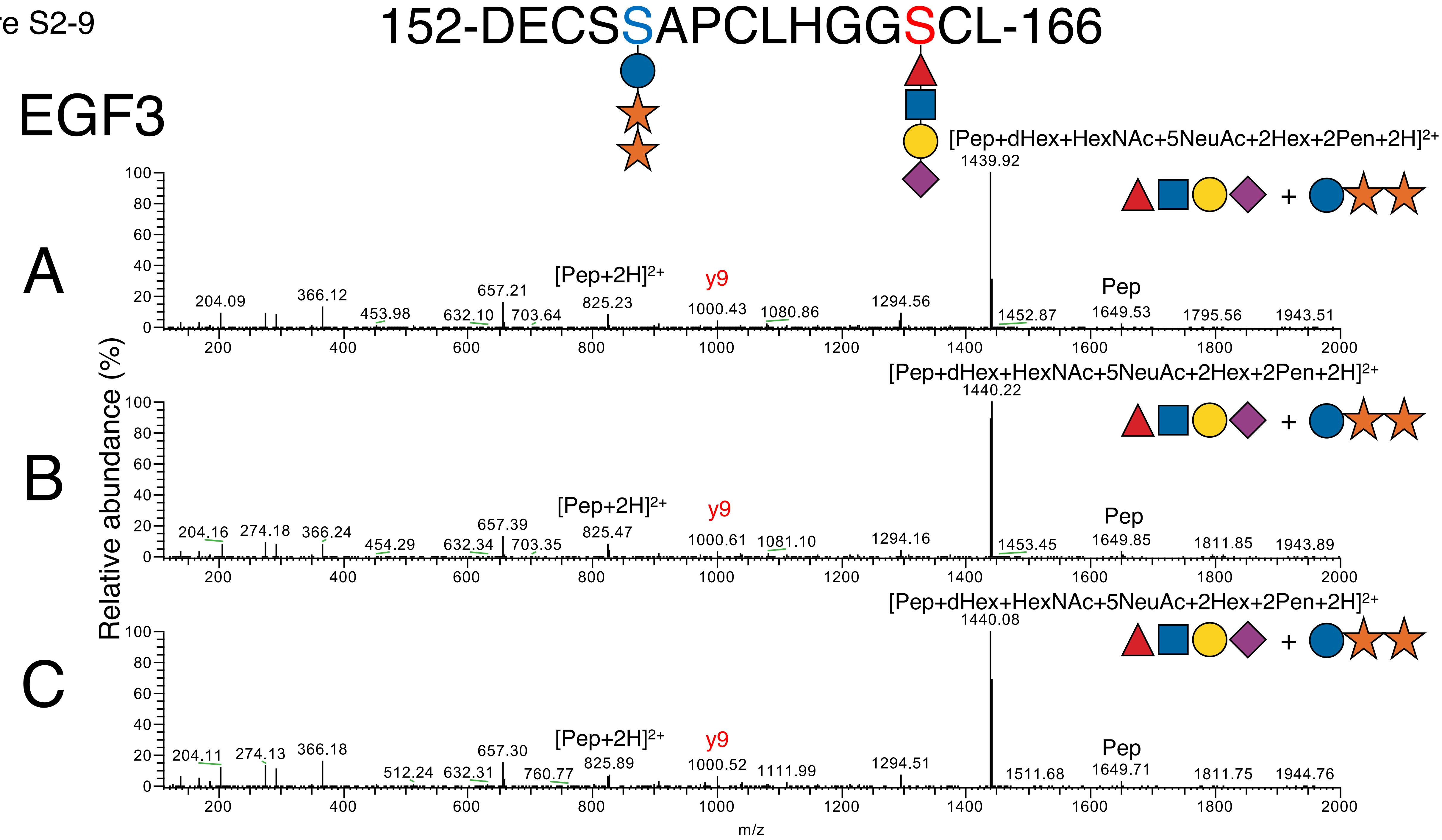
152-DECSSAPCLHGGSCL-166
EGF3
A
B
C
Relative abundance (%)
m/z
[Pep+dHex+HexNAc+5NeuAc+2Hex+2Pen+2H]2+
1439.92
[Pep+2H]2+
y9
Pep
204.09
366.12
453.98
632.10
657.21
703.64
825.23
1000.43
1080.86
1294.56
1452.87
1649.53
1795.56
1943.51
[Pep+dHex+HexNAc+5NeuAc+2Hex+2Pen+2H]2+
1440.22
204.16
274.18
366.24
454.29
632.34
657.39
703.35
825.47
1000.61
1081.10
1294.16
1453.45
1649.85
1811.85
1943.89
[Pep+dHex+HexNAc+5NeuAc+2Hex+2Pen+2H]2+
1440.08
204.11
274.13
366.18
512.24
632.31
657.30
760.77
825.89
1000.52
1111.99
1294.51
1511.68
1649.71
1811.75
1944.76

Figure S2-10

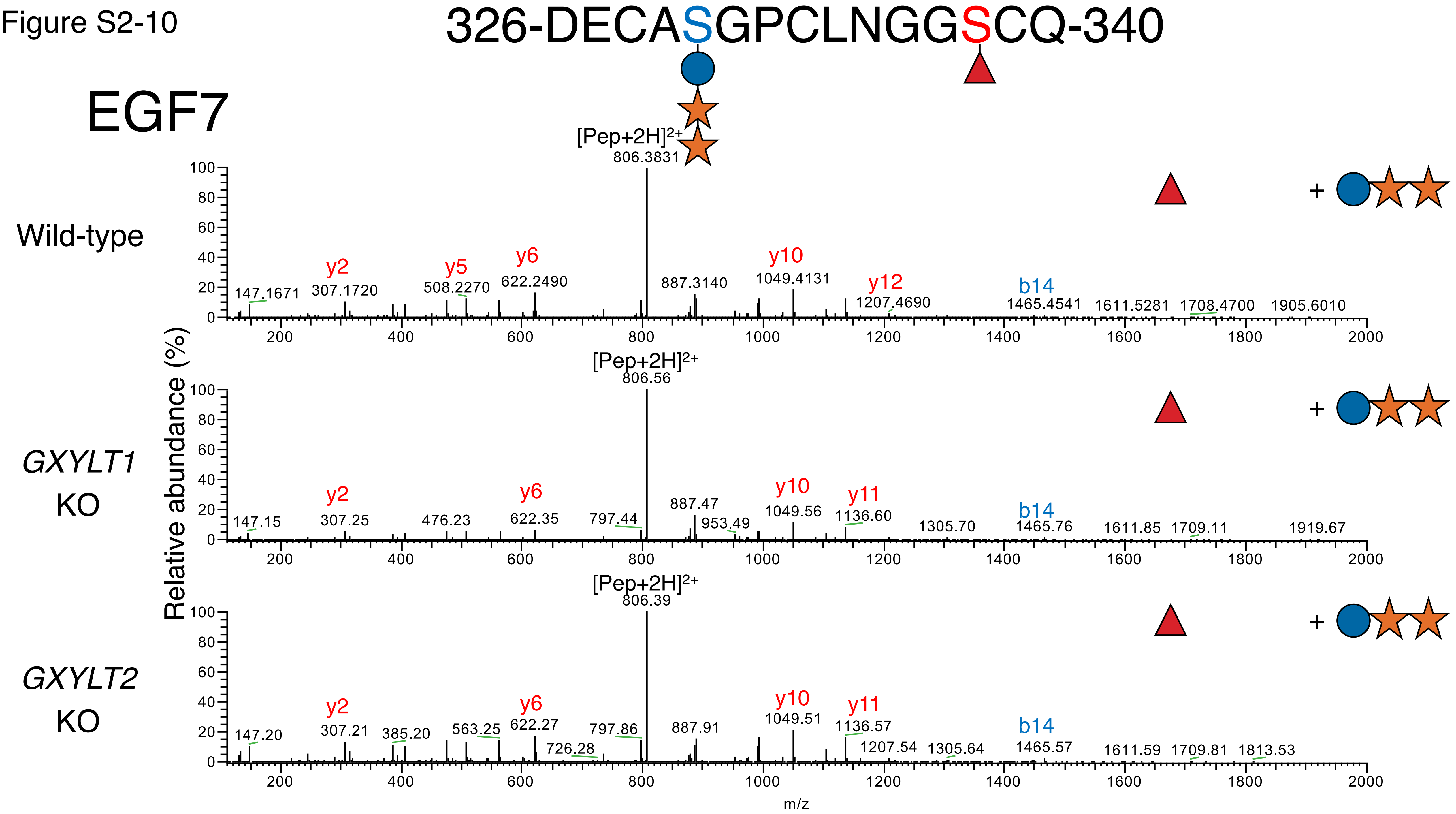

326-DECASGPCLNGGSCQ-340
EGF7
Wild-type
[Pep+2H]2+
806.3831
147.1671
y2
307.1720
y5
508.2270
y6
622.2490
887.3140
y10
1049.4131
y12
1207.4690
b14
1465.4541
1611.5281
1708.4700
1905.6010
+
GXYLT1
KO
[Pep+2H]2+
806.56
147.15
y2
307.25
476.23
y6
622.35
797.44
887.47
953.49
y10
1049.56
y11
1136.60
1305.70
b14
1465.76
1611.85
1709.11
1919.67
+
GXYLT2
KO
[Pep+2H]2+
806.39
147.20
y2
307.21
385.20
563.25
y6
622.27
726.28
797.86
887.91
y10
1049.51
y11
1136.57
1207.54
1305.64
b14
1465.57
1611.59
1709.81
1813.53
+
Relative abundance (%)
m/z

Figure S2-11

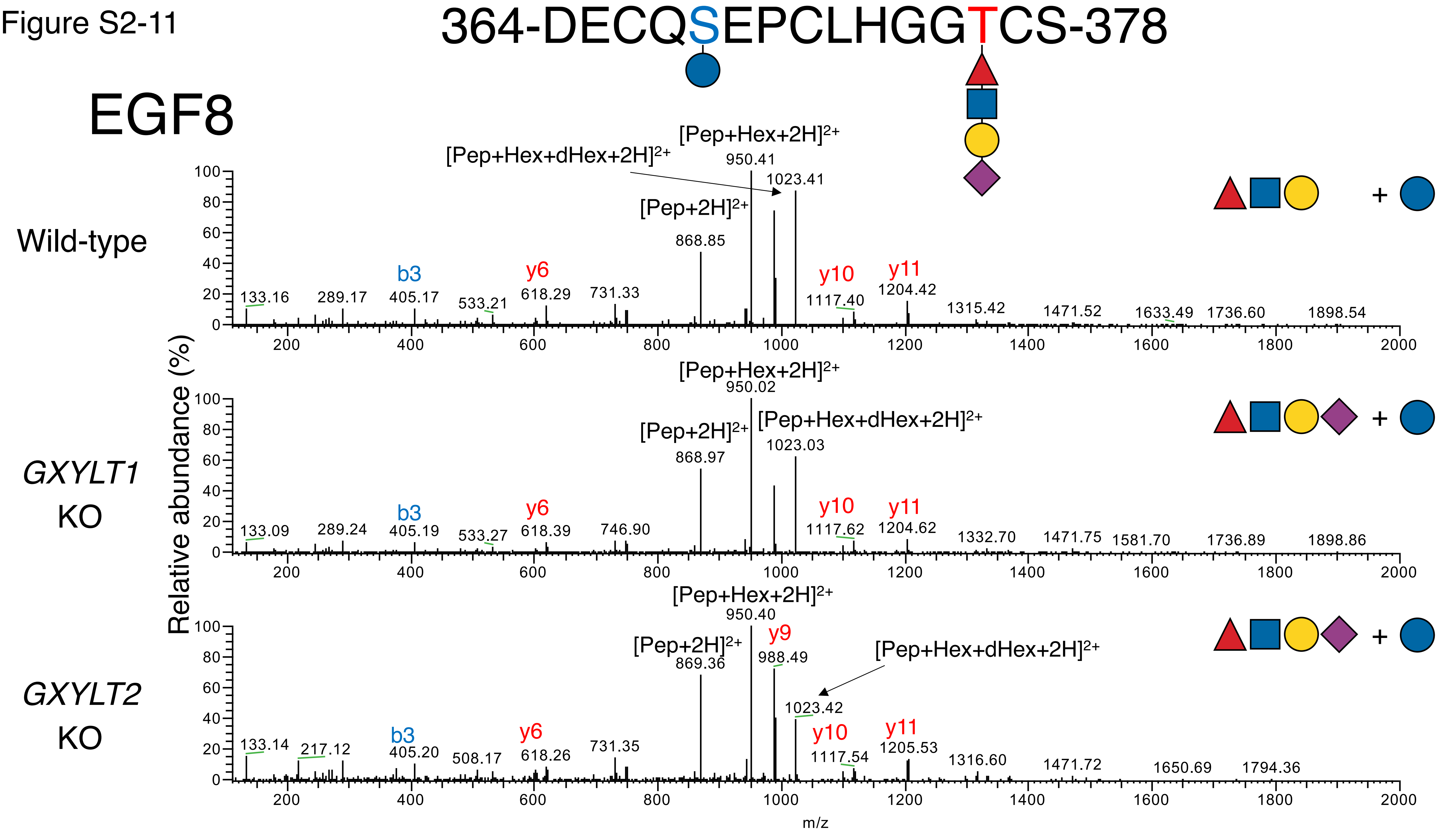

364-DECQSEPCLHGGTCS-378
EGF8
Wild-type
GXYLT1 KO
GXYLT2 KO
Relative abundance (%)
m/z
[Pep+Hex+2H]2+
950.41
[Pep+Hex+dHex+2H]2+
1023.41
[Pep+2H]2+
868.85
b3
405.17
y6
618.29
y10
1117.40
y11
1204.42
133.16
289.17
533.21
731.33
1315.42
1471.52
1633.49
1736.60
1898.54
950.02
1023.03
868.97
133.09
289.24
405.19
533.27
618.39
746.90
1117.62
1204.62
1332.70
1471.75
1581.70
1736.89
1898.86
950.40
869.36
y9
988.49
1023.42
133.14
217.12
405.20
508.17
618.26
731.35
1117.54
1205.53
1316.60
1471.72
1650.69
1794.36

Figure S2-12

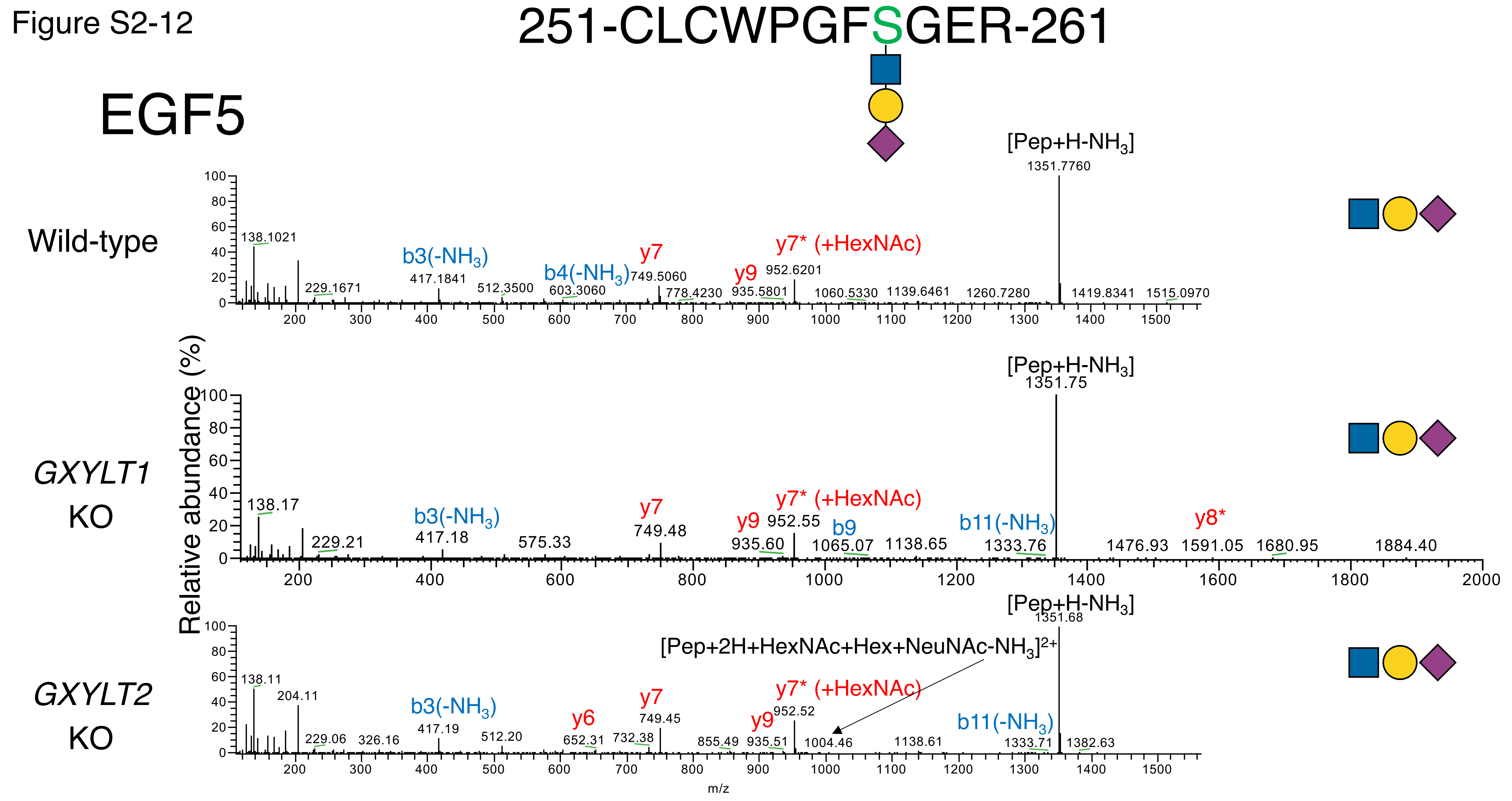
251-CLCWPGFSGER-261
EGF5
Wild-type
GXYLT1 KO
GXYLT2 KO
Relative abundance (%)
m/z
[Pep+H-NH3]
1351.7760
138.1021
229.1671
b3(-NH3)
417.1841
512.3500
b4(-NH3)
603.3060
y7
749.5060
778.4230
y9
935.5801
952.6201
y7* (+HexNAc)
1060.5330
1139.6461
1260.7280
1419.8341
1515.0970
1351.75
138.17
229.21
417.18
575.33
749.48
952.55
b9
1065.07
1138.65
b11(-NH3)
1333.76
1476.93
y8*
1591.05
1680.95
1884.40
1351.68
[Pep+2H+HexNAc+Hex+NeuNAc-NH3]2+
138.11
204.11
229.06
326.16
417.19
512.20
y6
652.31
732.38
749.45
855.49
935.51
952.52
1004.46
1138.61
1333.71
1382.63

Figure S2-13

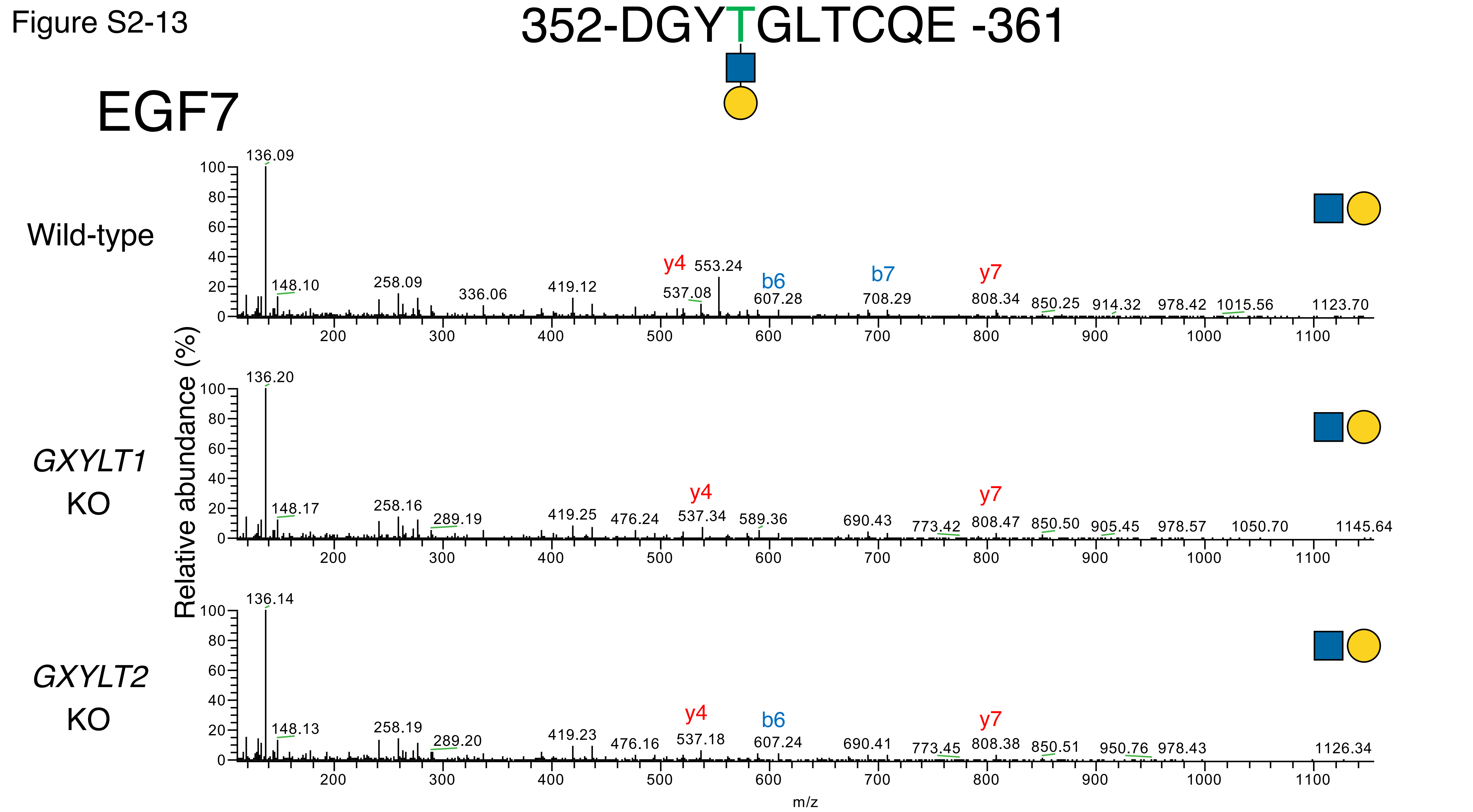

352-DGYTGLTCQE -361
EGF7
Wild-type
GXYLT1 KO
GXYLT2 KO
Relative abundance (%)
m/z
136.09
148.10
258.09
336.06
419.12
y4
537.08
553.24
b6
607.28
b7
708.29
y7
808.34
850.25
914.32
978.42
1015.56
1123.70
136.20
148.17
258.16
289.19
419.25
476.24
537.34
589.36
690.43
773.42
808.47
850.50
905.45
978.57
1050.70
1145.64
136.14
148.13
258.19
289.20
419.23
476.16
537.18
607.24
690.41
773.45
808.38
850.51
950.76
978.43
1126.34

Figure S2-14

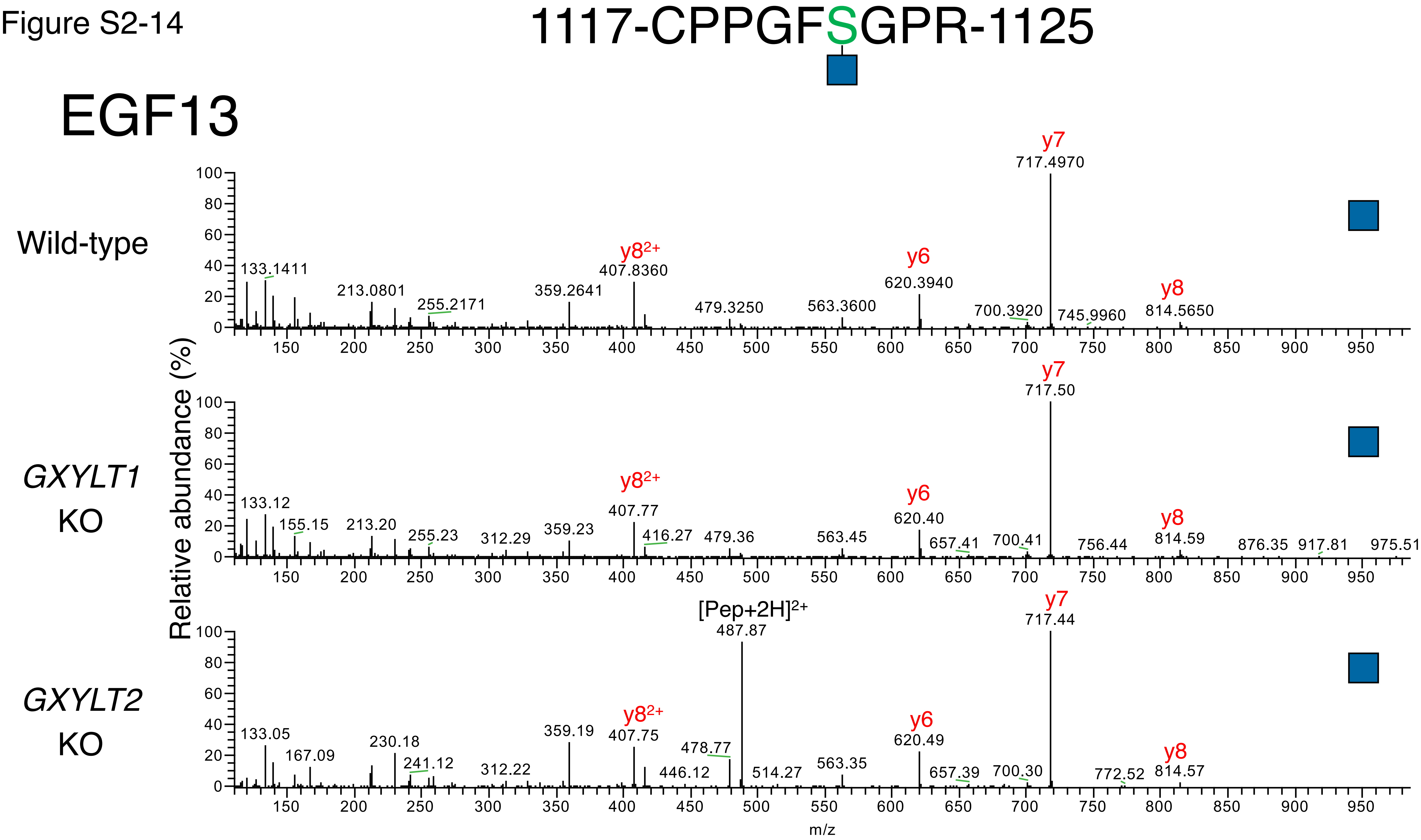
1117-CPPGFSGPR-1125
EGF13
Wild-type
GXYLT1 KO
GXYLT2 KO
Relative abundance (%)
m/z
y7
717.4970
y8^2+
407.8360
y6
620.3940
y8
814.5650
133.1411
213.0801
255.2171
359.2641
479.3250
563.3600
700.3920
745.9960
717.50
407.77
620.40
814.59
133.12
155.15
213.20
255.23
312.29
359.23
416.27
479.36
563.45
657.41
700.41
756.44
876.35
917.81
975.51
[Pep+2H]2+
487.87
717.44
407.75
620.49
814.57
133.05
167.09
230.18
241.12
312.22
359.19
446.12
478.77
514.27
563.35
657.39
700.30
772.52

Figure S2. MS/MS spectra of (glyco)peptides from CRB2.

Samples were generated in wild-type control HEK293T cells (*top*), *GXYLT1* KO cells (*middle*), or *GXYLT2* KO cells (*bottom*), transfected with a plasmid encoding mouse CRB2 EGF1-15 as described in the Experimental procedure section. MS/MS spectra confirmed the identity of (glyco)peptides based on the presence of peptide-specific b- and y-ions and neutral loss of predicted glycans. Amino acid sequences are shown with the identified fragment ions in the upper right corner. For each EGF repeat from CRB2, MS/MS spectra of glycopeptides modified with the most elongated glycoform derived from each cell clone are shown. The MS/MS spectra used for the analysis of *O*-Glc, *O*-Fuc, or *O*-GlcNAc glycans are shown in Figures S2-1 to 8, S2-9 to 11, or S2-12 to 14, respectively. The sequence of peptides, the predicted and measured mass-to-charge ratios, and the charge states are summarized in Tables S3 to S5.

Figure S3.Genomic sequence analysis of CRISPR-Cas9 targeted regions confirms that deletion of *GXYLT2* is successful.

GXYLT2 KO

1-bp insertion , or 4-bp deletion and 9-bp insertion in _GXYLT2_

Wild Type:GCTGTGGTGGCCTGTGGCA---------AT-CGGCTGGAG

Allele 1:GCTGTGGTGGCCTGTGGCA---------AT**C**CGGCTGGAG

Allele 2:GCTGTGGTGGCCTGTGGCA**CCTCCTCCA**-----GCTGGAG

Figure S3. Genomic sequence analysis of CRISPR-Cas9 targeted regions confirms the successful deletion of *GXYLT2*.

Genomic sequence analysis of *GXYLT2* in HEK293T clones. The analysis confirms the frameshift mutations in two alleles of *GXYLT2*, which cause truncation of the translational product.

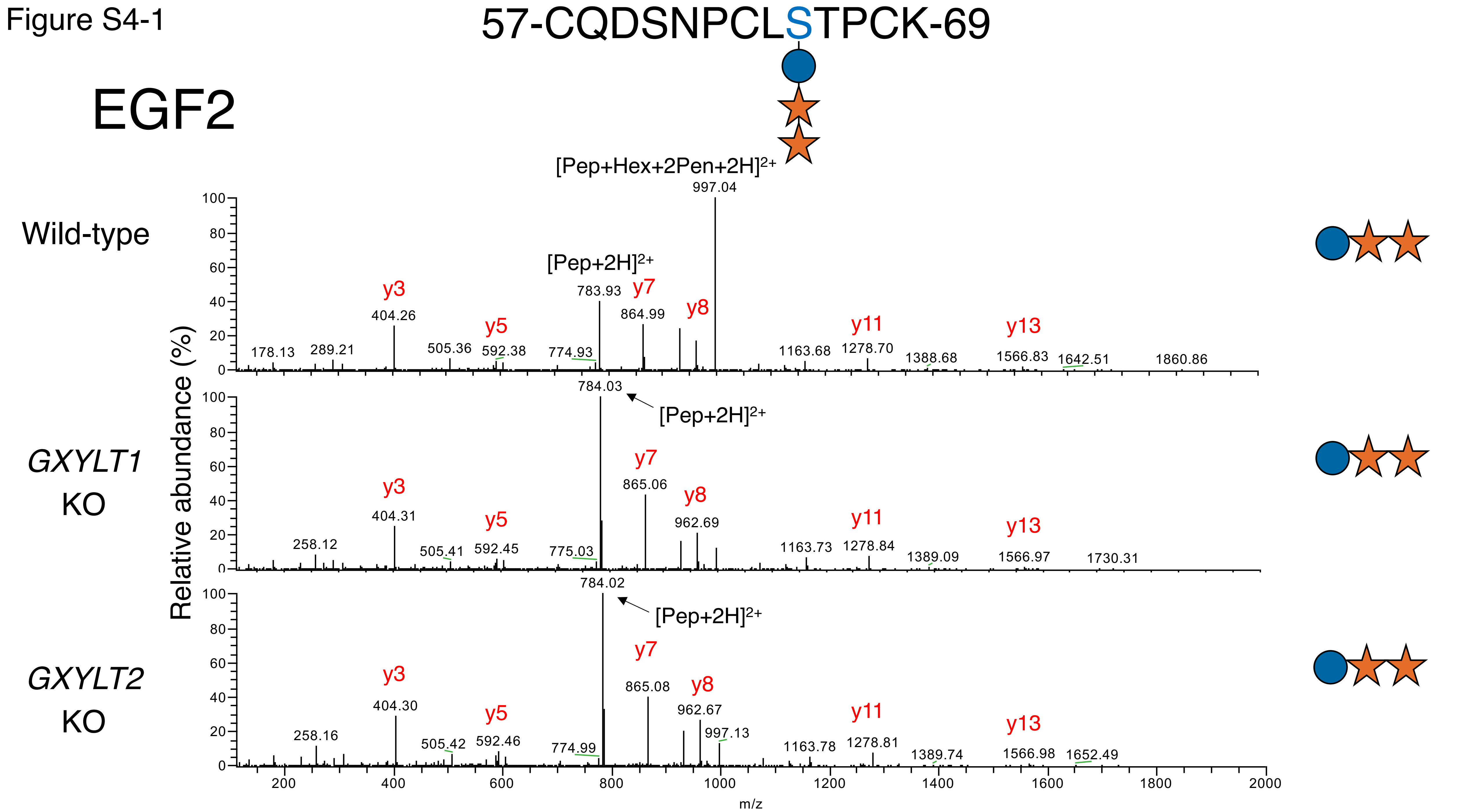
Figure S4-1
57-CQDSNPCLSTPCK-69
EGF2
[Pep+Hex+2Pen+2H]2+
997.04
Wild-type
[Pep+2H]2+
783.93
y7
y8
y3
404.26
864.99
y5
y11
y13
178.13
289.21
505.36
592.38
774.93
1163.68
1278.70
1388.68
1566.83
1642.51
1860.86
784.03
[Pep+2H]2+
GXYLT1 KO
y7
865.06
y3
y8
404.31
962.69
y5
y11
y13
258.12
505.41
592.45
775.03
1163.73
1278.84
1389.09
1566.97
1730.31
784.02
[Pep+2H]2+
GXYLT2 KO
y7
y3
865.08
y8
404.30
962.67
y5
y11
y13
997.13
258.16
505.42
592.46
774.99
1163.78
1278.81
1389.74
1566.98
1652.49
Relative abundance (%)
m/z

Figure S4-2

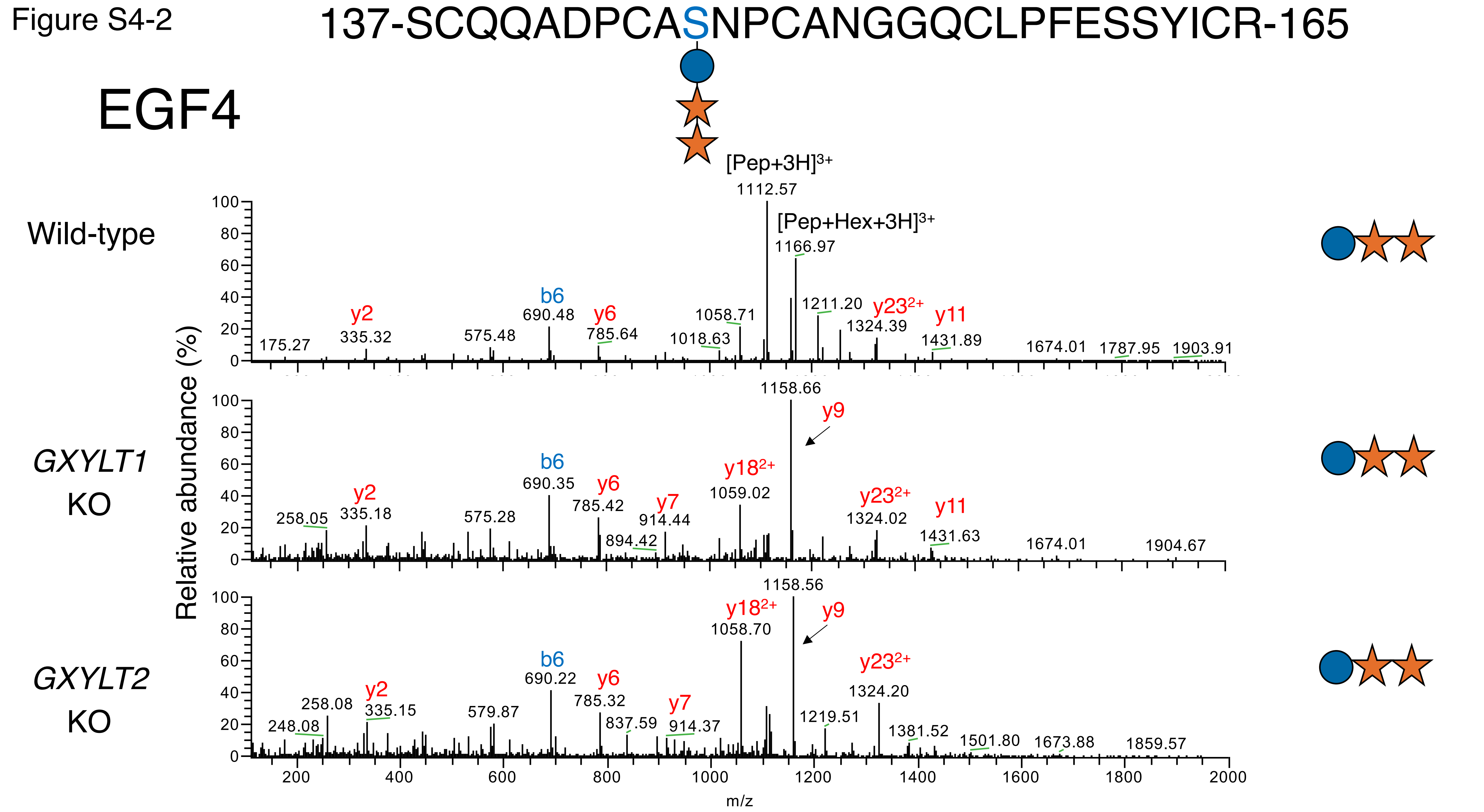

137-SCQQADPCASNPCANGGQCLPFESSYICR-165
EGF4
Wild-type
GXYLT1 KO
GXYLT2 KO
Relative abundance (%)
m/z
[Pep+3H]3+
1112.57
[Pep+Hex+3H]3+
1166.97
b6
690.48
y2
335.32
y6
785.64
y11
1431.89
y23 2+
1324.39
175.27
575.48
1018.63
1058.71
1211.20
1674.01
1787.95
1903.91
1158.66
y9
y18 2+
1059.02
690.35
335.18
785.42
y7
914.44
1324.02
1431.63
258.05
575.28
894.42
1904.67
1158.56
1058.70
690.22
335.15
785.32
914.37
1324.20
258.08
248.08
579.87
837.59
1219.51
1381.52
1501.80
1673.88
1859.57

Figure S4-3

328-TGEDCSENIDDCASAACF-345

EGF9

Wild-type

No data

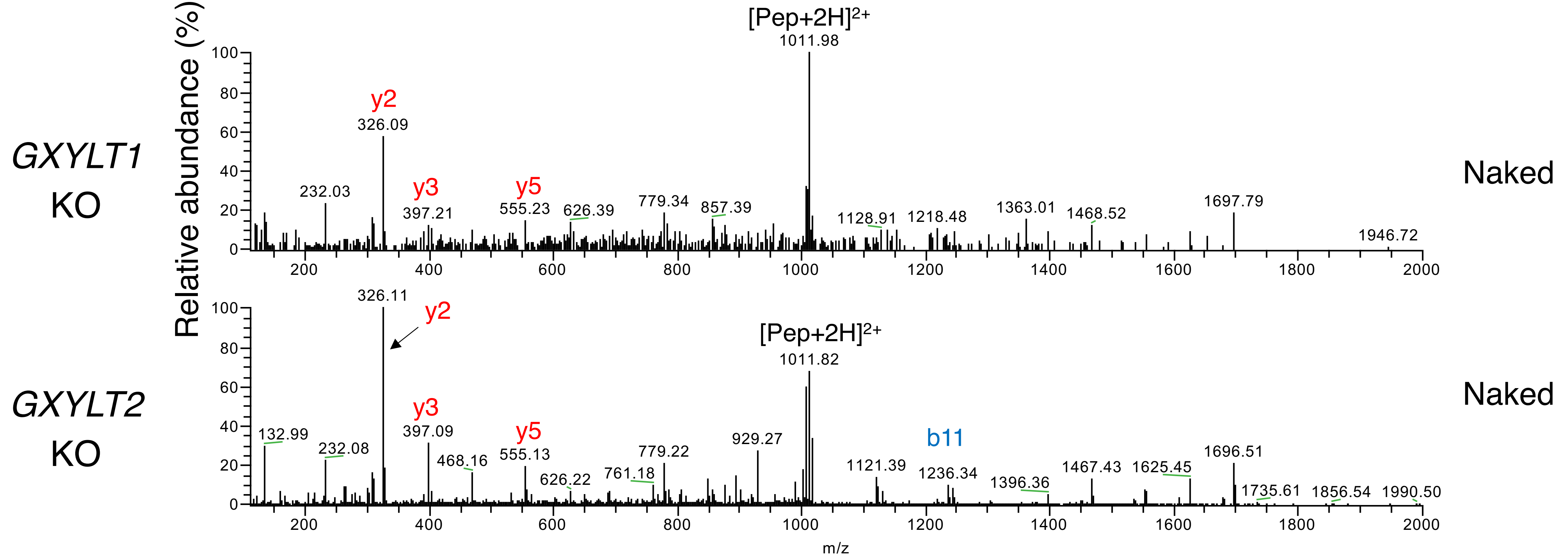

Figure S4-4

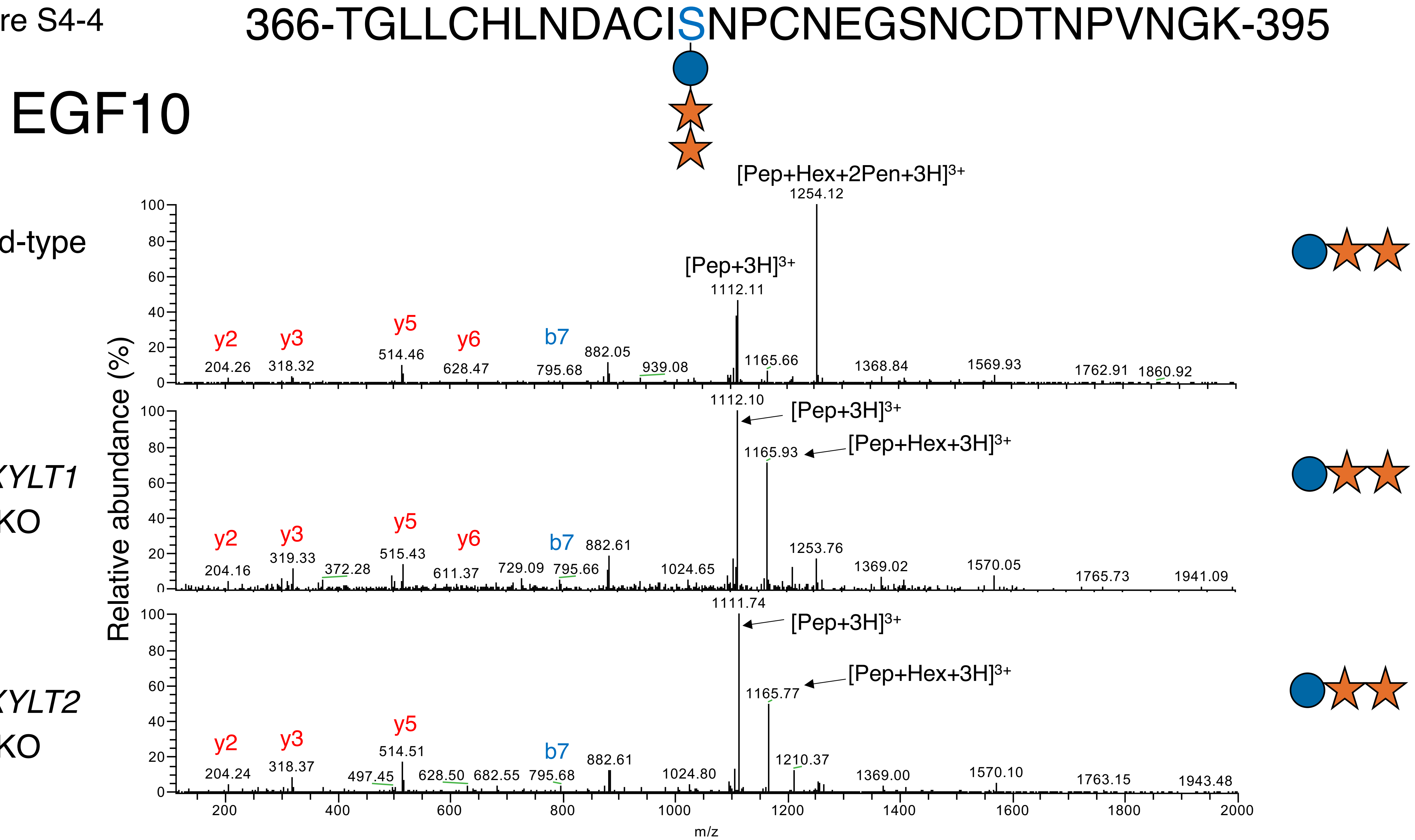
366-TGLLCHLNDACISNPCNEGSNCDTNPVNGK-395
EGF10
Wild-type
GXYLT1 KO
GXYLT2 KO
Relative abundance (%)
m/z
[Pep+Hex+2Pen+3H]3+
1254.12
[Pep+3H]3+
1112.11
y2
204.26
y3
318.32
y5
514.46
y6
628.47
b7
795.68
882.05
939.08
1165.66
1368.84
1569.93
1762.91
1860.92
1112.10
[Pep+3H]3+
[Pep+Hex+3H]3+
1165.93
204.16
319.33
372.28
515.43
611.37
729.09
795.66
882.61
1024.65
1253.76
1369.02
1570.05
1765.73
1941.09
1111.74
[Pep+3H]3+
[Pep+Hex+3H]3+
1165.77
204.24
318.37
497.45
514.51
628.50
682.55
795.68
882.61
1024.80
1210.37
1369.00
1570.10
1763.15
1943.48

Figure S4-5

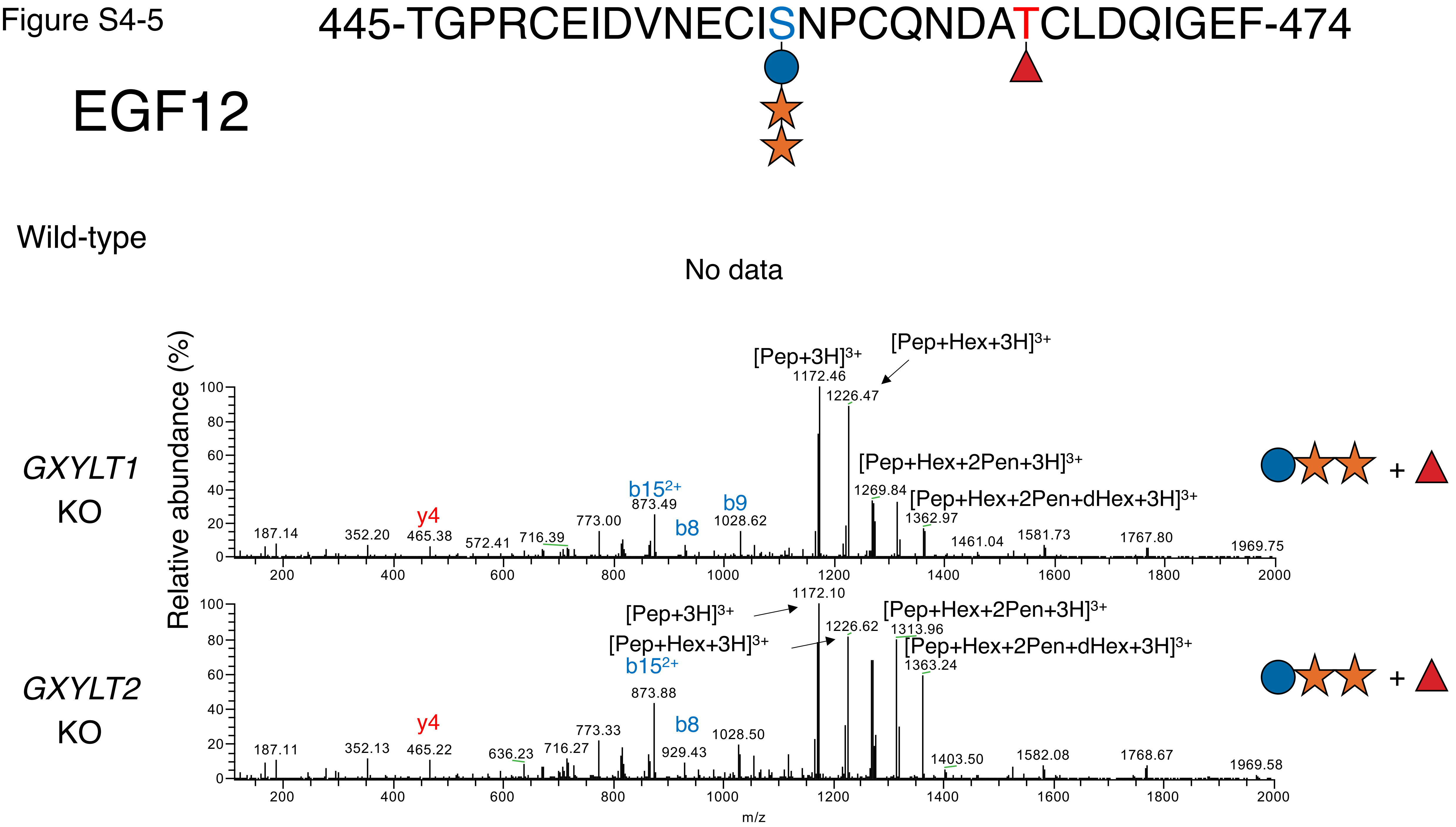

445-TGPRCEIDVNECISNPCQNDATCLDQIGEF-474
EGF12
Wild-type
No data
GXYLT1
KO
GXYLT2
KO
Relative abundance (%)
m/z
[Pep+3H]3+
1172.46
[Pep+Hex+3H]3+
1226.47
[Pep+Hex+2Pen+3H]3+
1269.84
[Pep+Hex+2Pen+dHex+3H]3+
1362.97
b15 2+
873.49
b9
1028.62
b8
y4
465.38
187.14
352.20
572.41
716.39
773.00
1461.04
1581.73
1767.80
1969.75
1172.10
[Pep+3H]3+
[Pep+Hex+3H]3+
1226.62
[Pep+Hex+2Pen+3H]3+
1313.96
[Pep+Hex+2Pen+dHex+3H]3+
1363.24
b15 2+
873.88
b8
929.43
1028.50
y4
465.22
187.11
352.13
636.23
716.27
773.33
1403.50
1582.08
1768.67
1969.58
+

Figure S4-6

487-CEINTDECASSPCLHNGHCMDKINEF-512

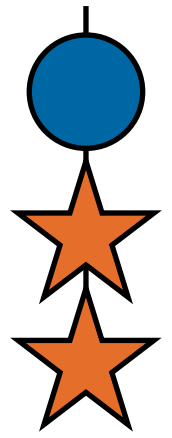

EGF13

Wild-type

No data

GXYLT1 KO

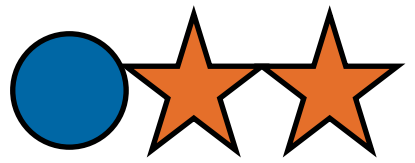

GXYLT2 KO

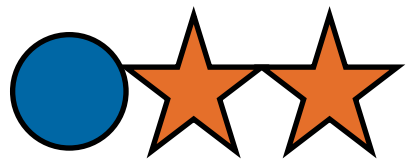

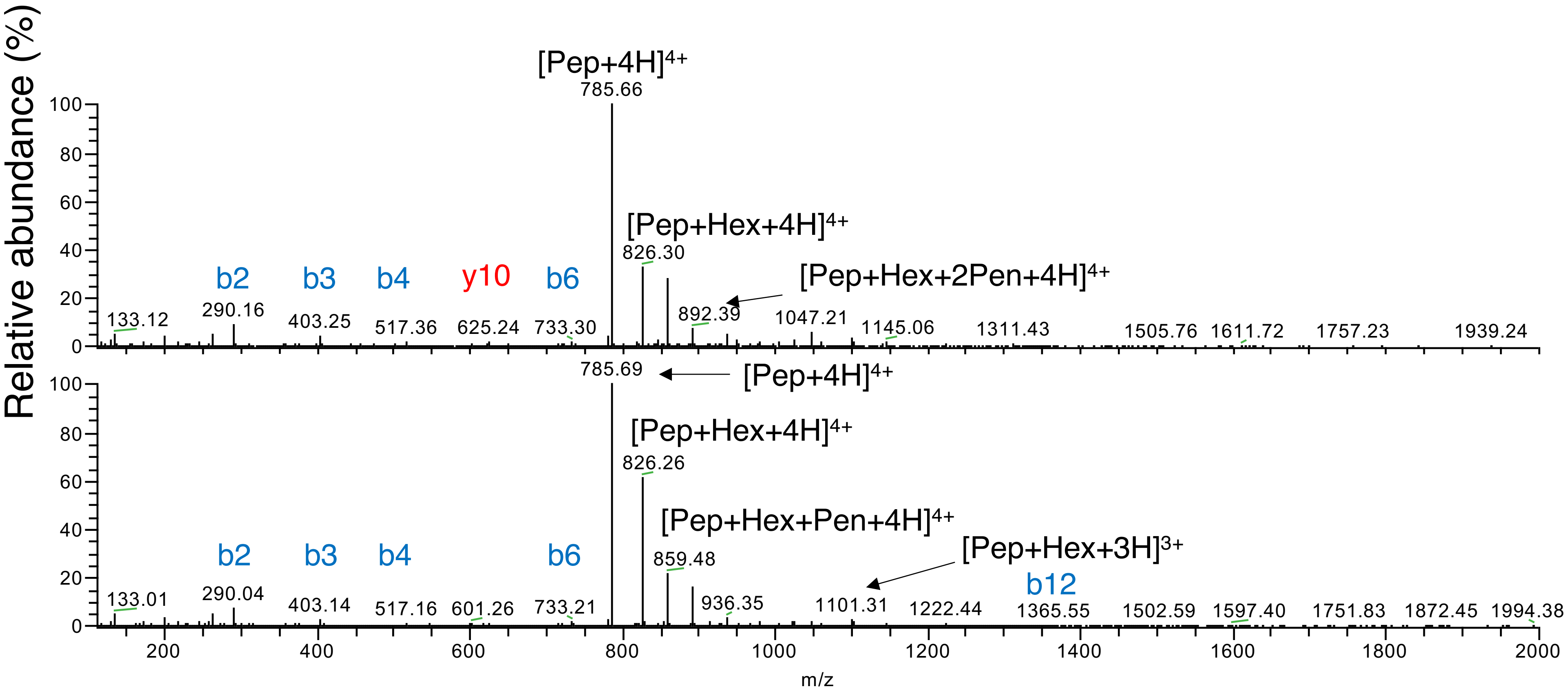

Figure S4-7

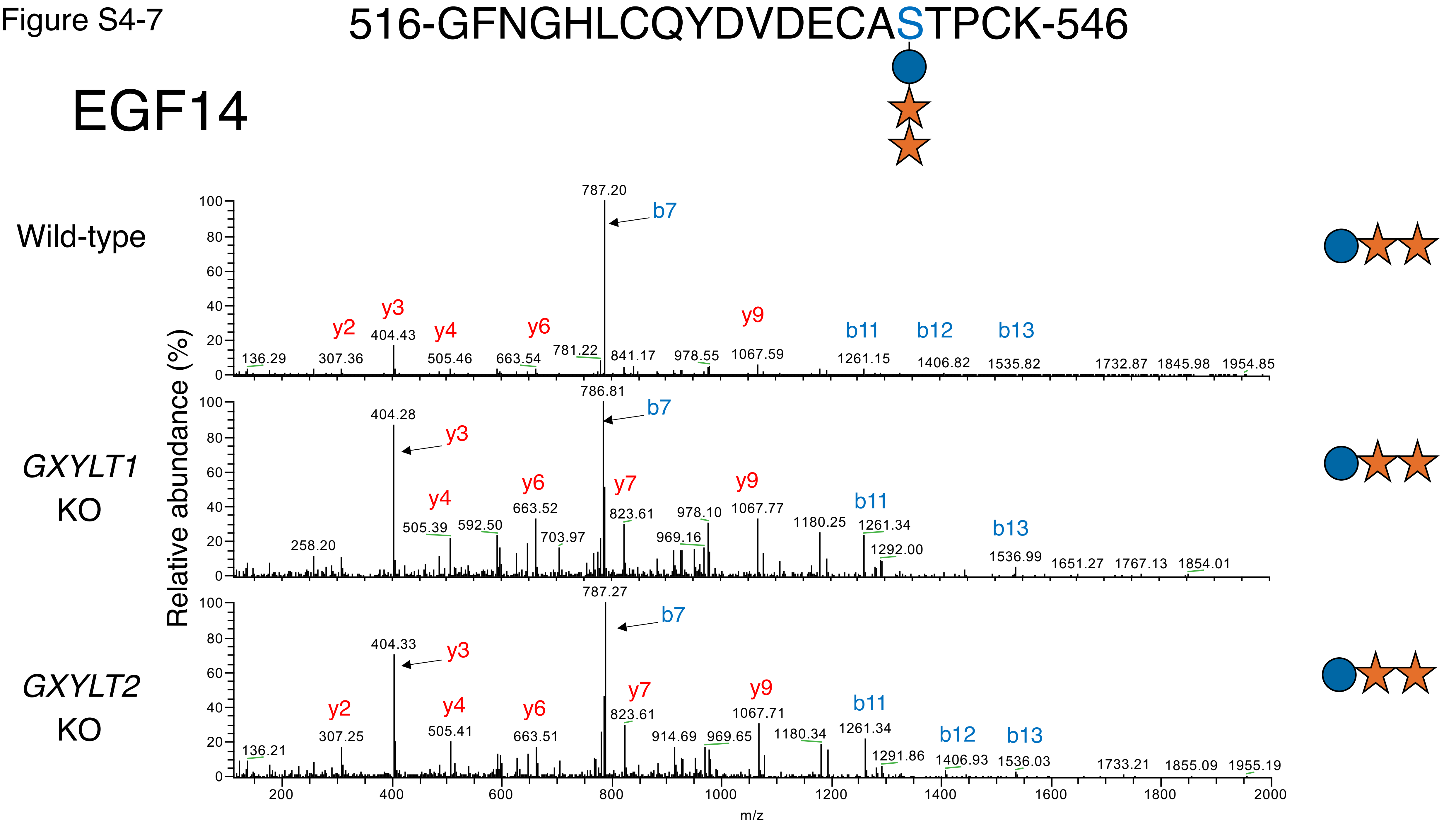
516-GFNGHLCQYDVDECASTPCK-546
EGF14
Wild-type
GXYLT1 KO
GXYLT2 KO
Relative abundance (%)
m/z
787.20
b7
y2
y3
y4
y6
y9
b11
b12
b13
136.29
307.36
404.43
505.46
663.54
781.22
841.17
978.55
1067.59
1261.15
1406.82
1535.82
1732.87
1845.98
1954.85
786.81
404.28
y7
258.20
505.39
592.50
663.52
703.97
823.61
969.16
978.10
1067.77
1180.25
1261.34
1292.00
1536.99
1651.27
1767.13
1854.01
787.27
404.33
136.21
307.25
505.41
663.51
823.61
914.69
969.65
1067.71
1180.34
1261.34
1291.86
1406.93
1536.03
1733.21
1855.09
1955.19

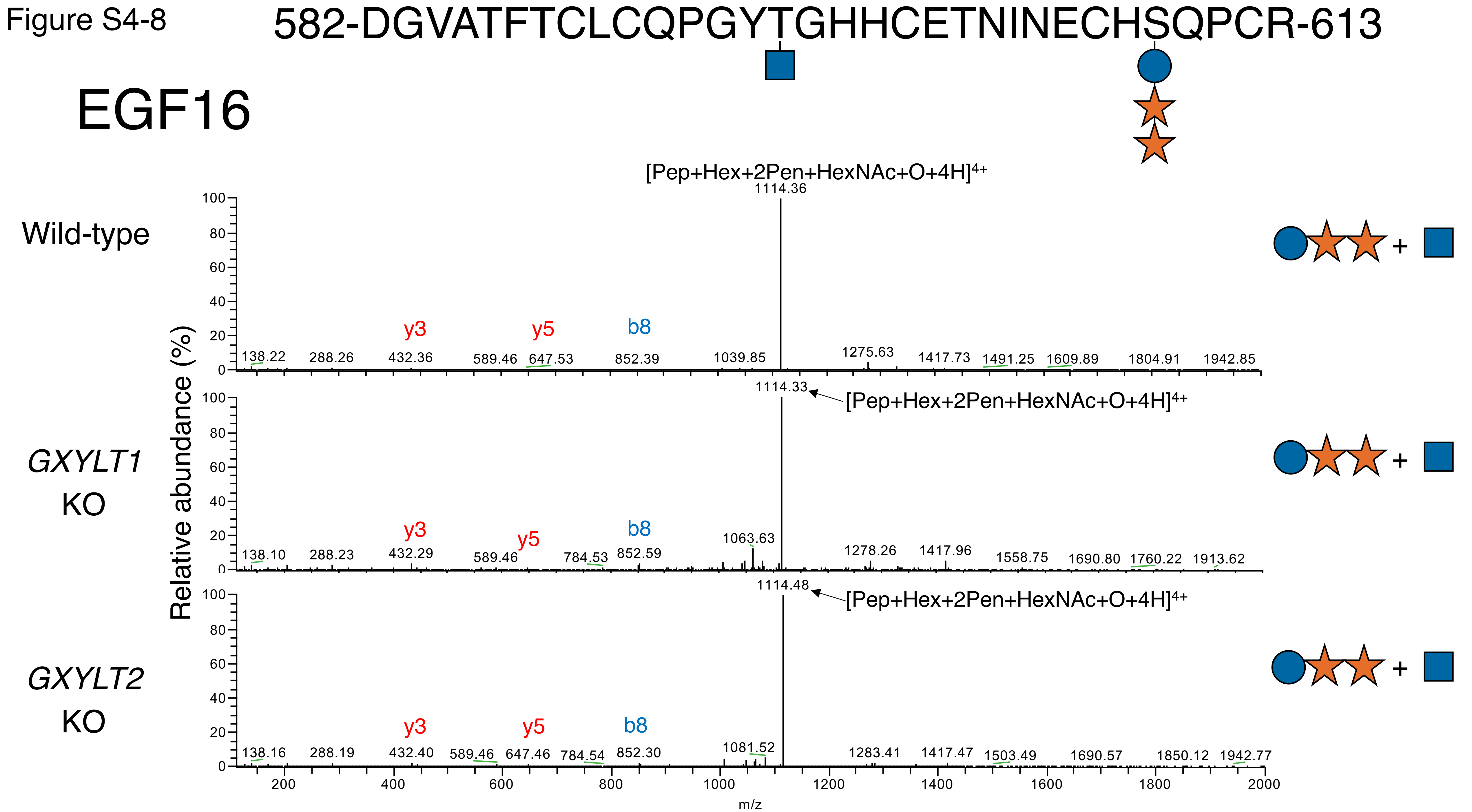

Figure S4-8
582-DGVATFTCLCQPGYTGHHCETNINECHSQPCR-613
EGF16
[Pep+Hex+2Pen+HexNAc+O+4H]4+
Wild-type
GXYLT1 KO
GXYLT2 KO
Relative abundance (%)
m/z
y3
y5
b8
1114.36
1114.33
1114.48
138.22
288.26
432.36
589.46
647.53
852.39
1039.85
1275.63
1417.73
1491.25
1609.89
1804.91
1942.85
138.10
288.23
432.29
589.46
784.53
852.59
1063.63
1278.26
1417.96
1558.75
1690.80
1760.22
1913.62
138.16
288.19
432.40
589.46
647.46
784.54
852.30
1081.52
1283.41
1417.47
1503.49
1690.57
1850.12
1942.77

Figure S4-9

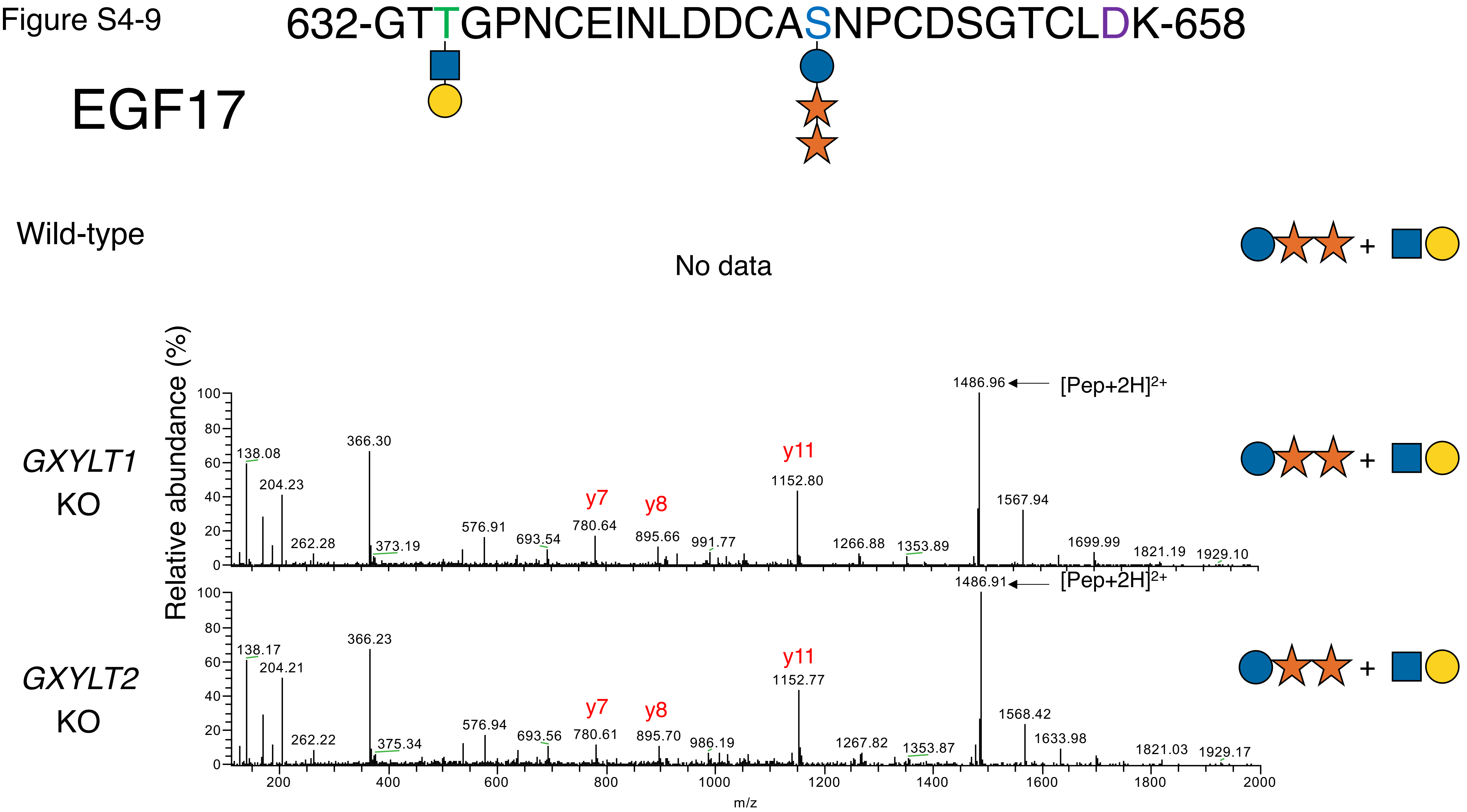
632-GTTGPNCEINLDDCASNPCDSGTCLDK-658
EGF17
Wild-type
No data
GXYLT1 KO
GXYLT2 KO
Relative abundance (%)
m/z
[Pep+2H]2+
1486.96
1486.91
y7
y8
y11
138.08
204.23
262.28
366.30
373.19
576.91
693.54
780.64
895.66
991.77
1152.80
1266.88
1353.89
1567.94
1699.99
1821.19
1929.10
138.17
204.21
262.22
366.23
375.34
576.94
693.56
780.61
895.70
986.19
1152.77
1267.82
1353.87
1568.42
1633.98
1821.03
1929.17

Figure S4-10

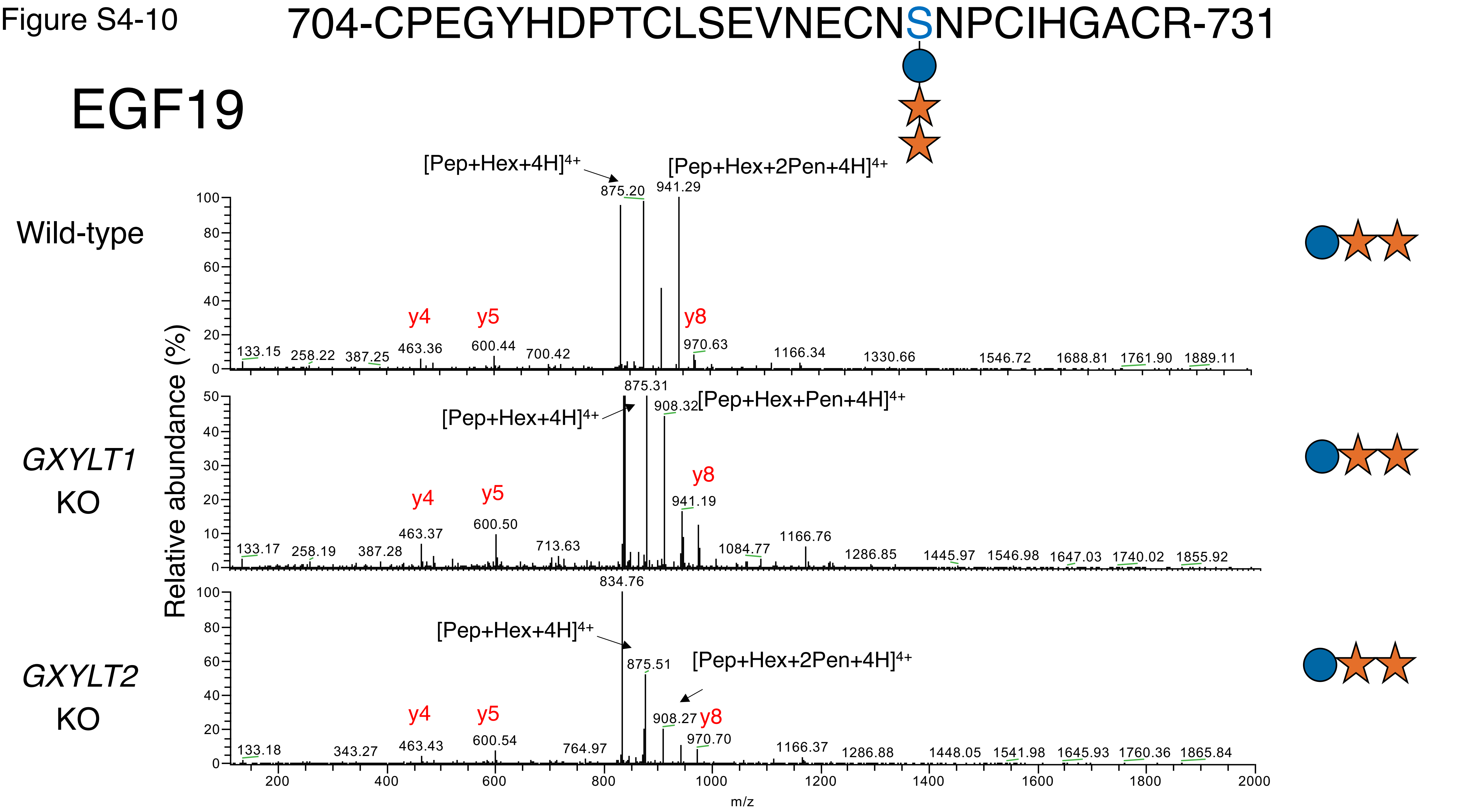
704-CPEGYHDPTCLSEVNECNSNPCIHGACR-731
EGF19
Wild-type
GXYLT1 KO
GXYLT2 KO
Relative abundance (%)
m/z
[Pep+Hex+4H]4+
[Pep+Hex+2Pen+4H]4+
[Pep+Hex+Pen+4H]4+
y4
y5
y8
875.20
941.29
875.31
908.32
834.76
875.51
908.27
970.63
941.19
970.70
463.36
600.44
463.37
600.50
463.43
600.54
133.15
258.22
387.25
700.42
1166.34
1330.66
1546.72
1688.81
1761.90
1889.11
133.17
258.19
387.28
713.63
1084.77
1166.76
1286.85
1445.97
1546.98
1647.03
1740.02
1855.92
133.18
343.27
764.97
1166.37
1286.88
1448.05
1541.98
1645.93
1760.36
1865.84

Figure S4-11

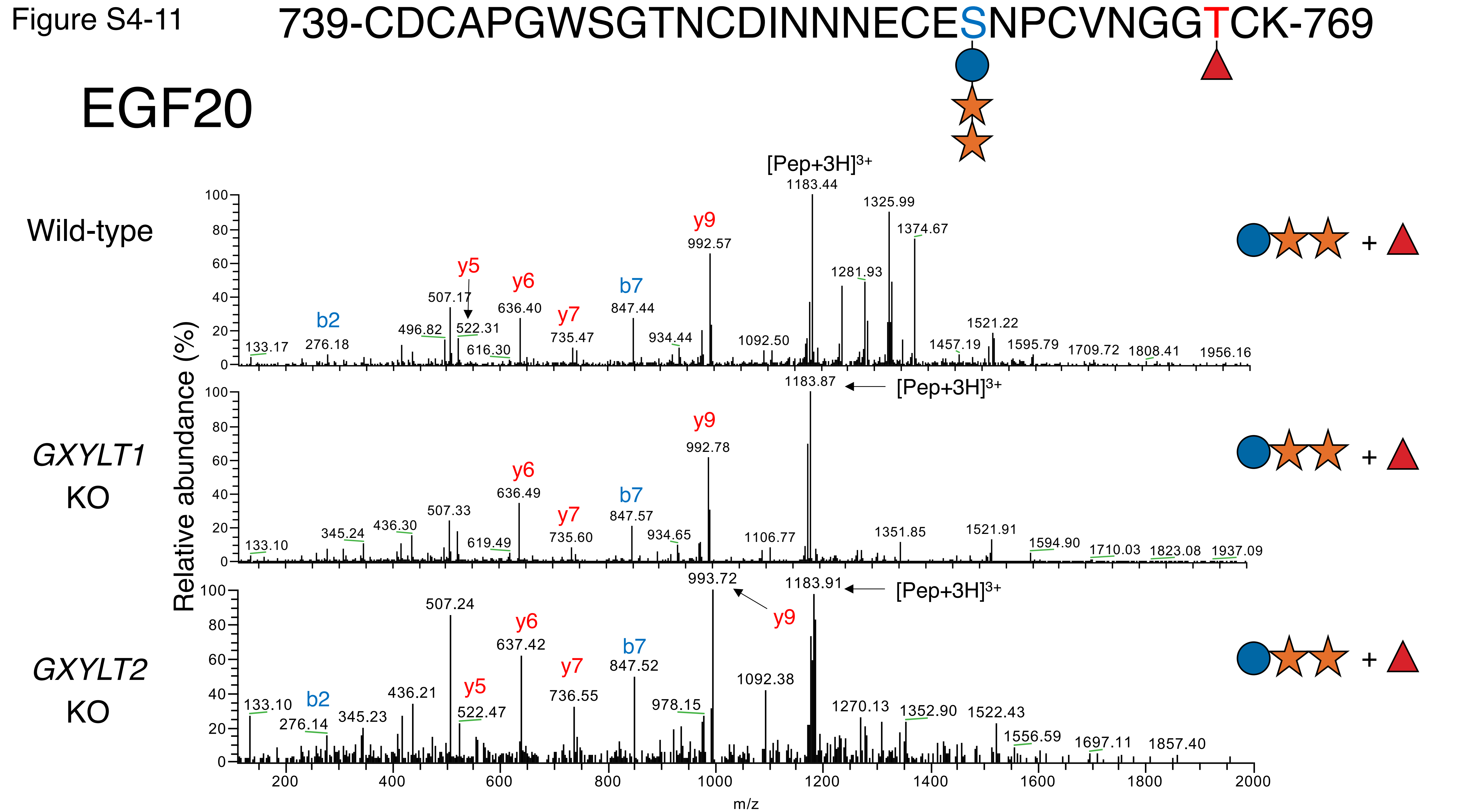

739-CDCAPGWSGTNCDINNNECESNPCVNGGTCK-769
EGF20
Wild-type
GXYLT1 KO
GXYLT2 KO
Relative abundance (%)
m/z
[Pep+3H]3+
1183.44
1325.99
1374.67
y9
992.57
1281.93
y5
y6
b7
b2
507.17
636.40
847.44
522.31
y7
496.82
276.18
735.47
934.44
1092.50
1521.22
133.17
616.30
1457.19
1595.79
1709.72
1808.41
1956.16
1183.87
992.78
636.49
847.57
507.33
735.60
436.30
934.65
345.24
619.49
1106.77
1351.85
1521.91
133.10
1594.90
1710.03
1823.08
1937.09
993.72
1183.91
507.24
637.42
847.52
736.55
436.21
1092.38
522.47
978.15
345.23
276.14
1270.13
1352.90
1522.43
1556.59
1697.11
1857.40

Figure S4-12

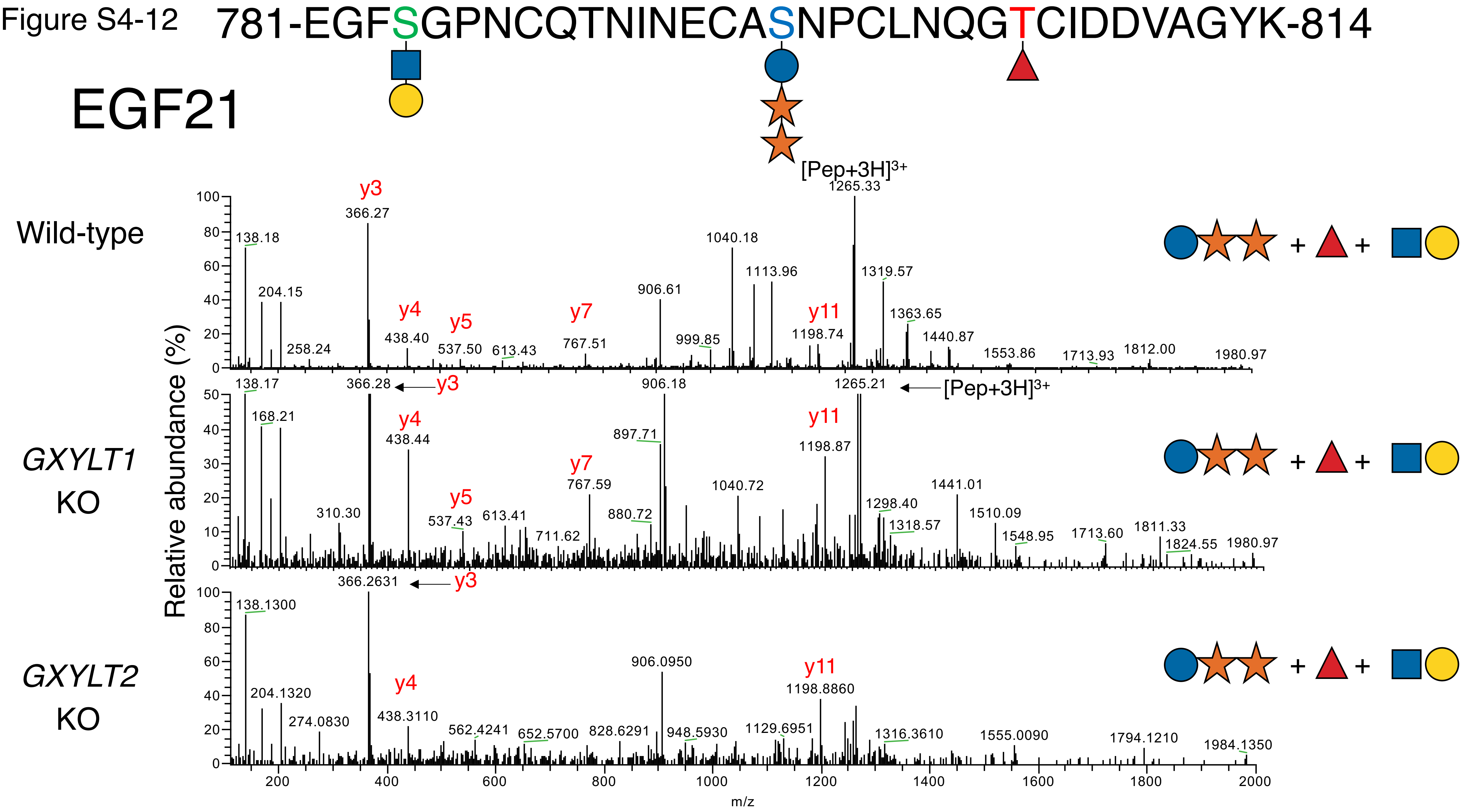

781-EGFSGPNCQTNINECASNPCLNQGTCIDDVAGYK-814
EGF21
[Pep+3H]3+
Wild-type
GXYLT1 KO
GXYLT2 KO
Relative abundance (%)
m/z
y3
y4
y5
y7
y11
138.18
204.15
258.24
366.27
438.40
537.50
613.43
767.51
906.61
999.85
1040.18
1113.96
1198.74
1265.33
1319.57
1363.65
1440.87
1553.86
1713.93
1812.00
1980.97
138.17
168.21
310.30
366.28
438.44
537.43
613.41
711.62
767.59
880.72
897.71
906.18
1040.72
1198.87
1265.21
1298.40
1318.57
1441.01
1510.09
1548.95
1713.60
1811.33
1824.55
1980.97
138.1300
204.1320
274.0830
366.2631
438.3110
562.4241
652.5700
828.6291
906.0950
948.5930
1129.6951
1198.8860
1316.3610
1555.0090
1794.1210
1984.1350

Figure S4-13

1021-DVNECDSRPCLHGGTCQDSYGTY-1043

EGF27

Wild-type

No data

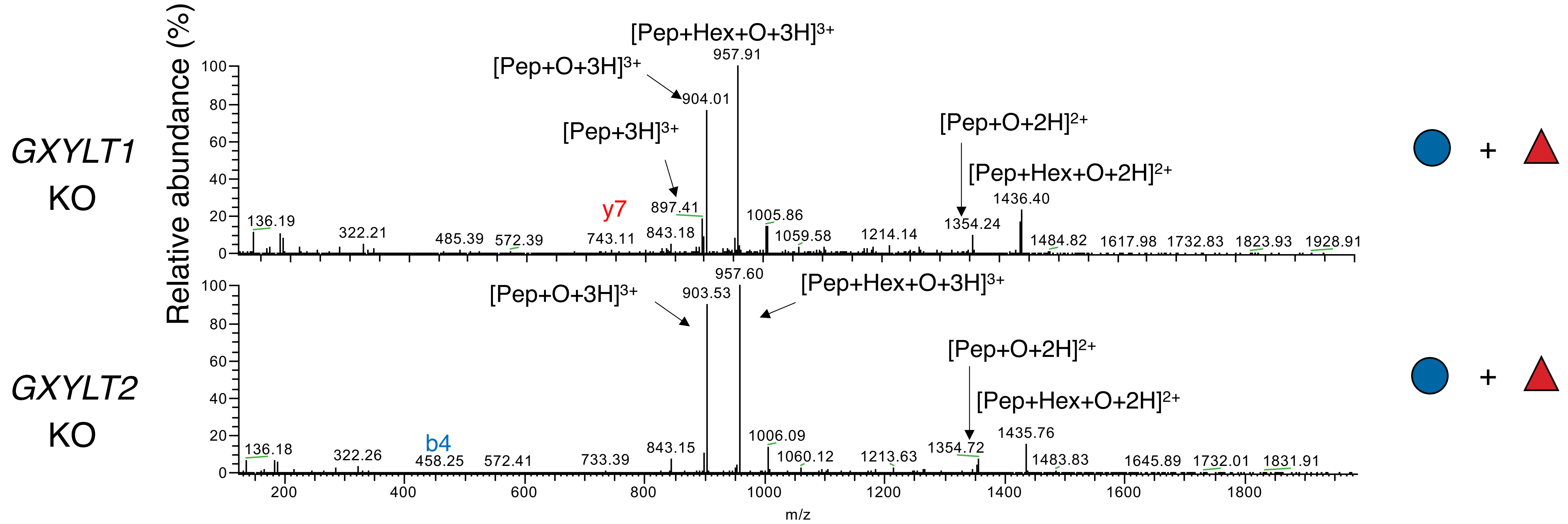

Figure S4-14

1062-WCDSAPCK-1069

EGF28

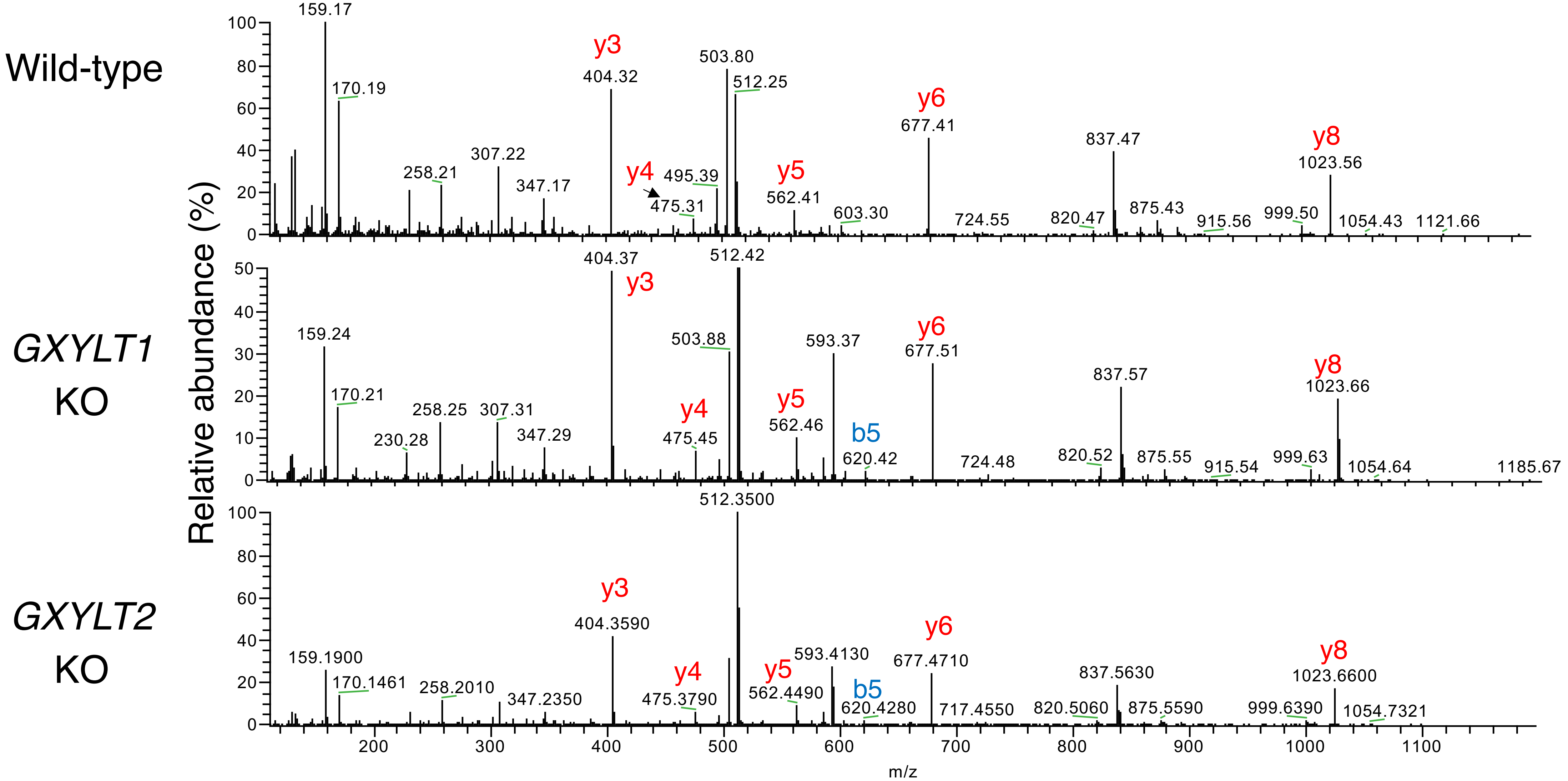

Figure S4-15

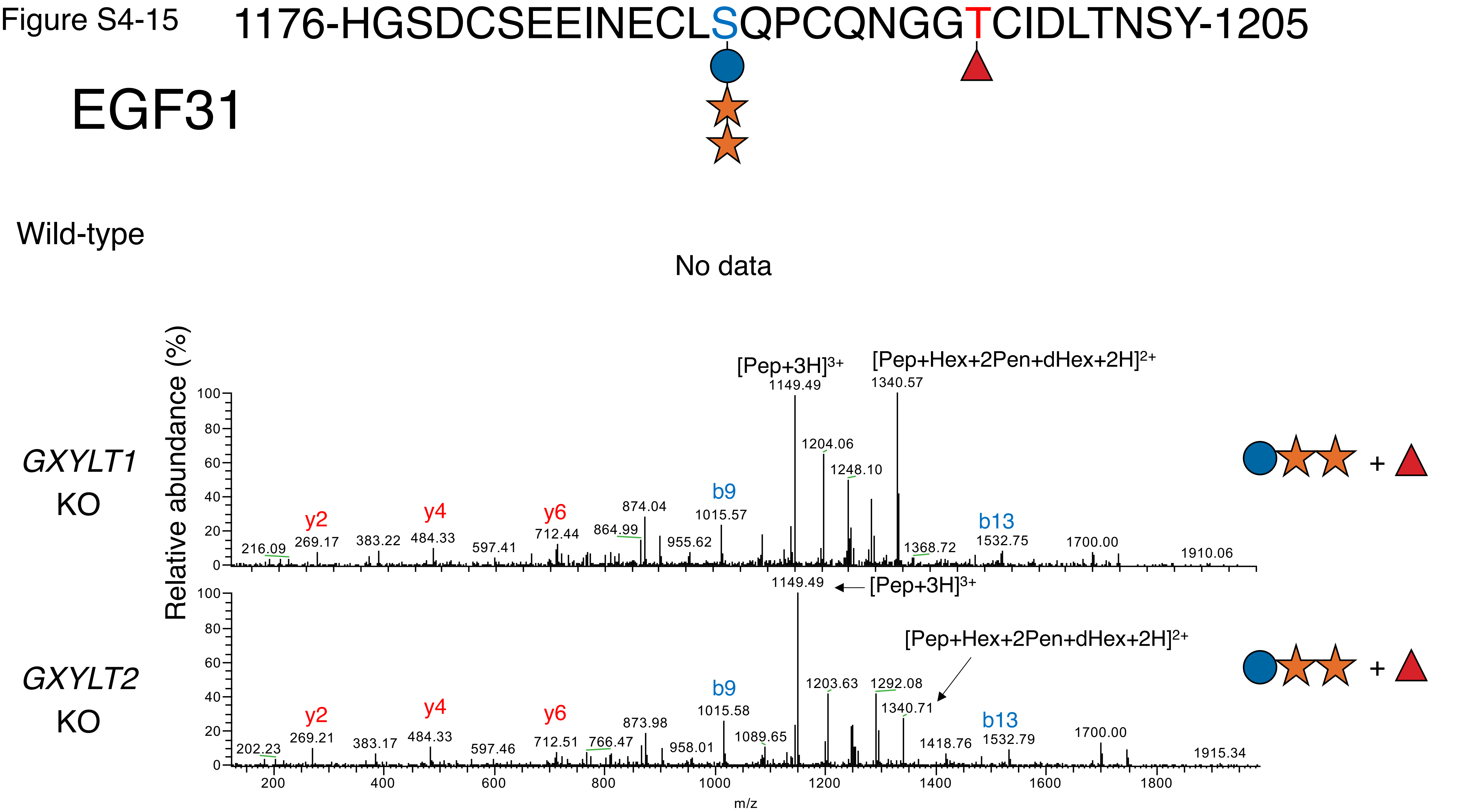

1176-HGSDCSEEINECLSQPCQNGGTCIDLTNSY-1205
EGF31
Wild-type
No data
GXYLT1 KO
GXYLT2 KO
Relative abundance (%)
m/z
[Pep+3H]3+
1149.49
[Pep+Hex+2Pen+dHex+2H]2+
1340.57
1204.06
1248.10
b9
1015.57
b13
1532.75
y2
269.17
y4
484.33
y6
712.44
216.09
383.22
597.41
864.99
874.04
955.62
1368.72
1700.00
1910.06
[Pep+3H]3+
1149.49
[Pep+Hex+2Pen+dHex+2H]2+
1340.71
1203.63
1292.08
b9
1015.58
b13
1532.79
y2
269.21
y4
484.33
y6
712.51
202.23
383.17
597.46
766.47
873.98
958.01
1089.65
1418.76
1700.00
1915.34

Figure S4-16

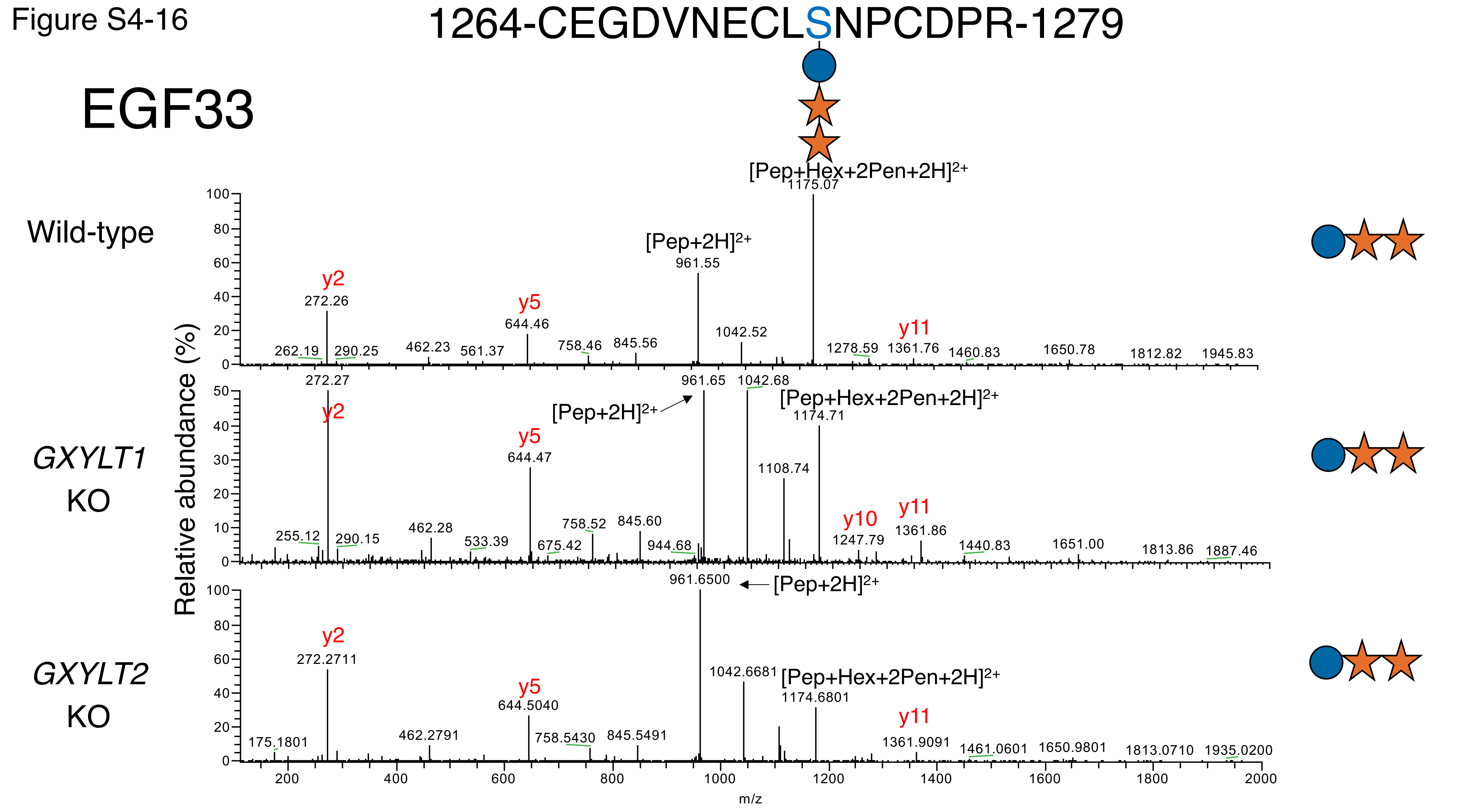

1264-CEGDVNECLSNPCDPR-1279
EGF33
Wild-type
GXYLT1 KO
GXYLT2 KO
Relative abundance (%)
m/z
[Pep+Hex+2Pen+2H]2+
1175.07
[Pep+2H]2+
961.55
y2
272.26
y5
644.46
y11
1361.76
262.19
290.25
462.23
561.37
758.46
845.56
1042.52
1278.59
1460.83
1650.78
1812.82
1945.83
272.27
961.65
1042.68
1174.71
644.47
1108.74
y10
1247.79
1361.86
255.12
290.15
462.28
533.39
675.42
758.52
845.60
944.68
1440.83
1651.00
1813.86
1887.46
961.6500
272.2711
644.5040
1042.6681
1174.6801
1361.9091
175.1801
462.2791
758.5430
845.5491
1461.0601
1650.9801
1813.0710
1935.0200

Figure S4-17

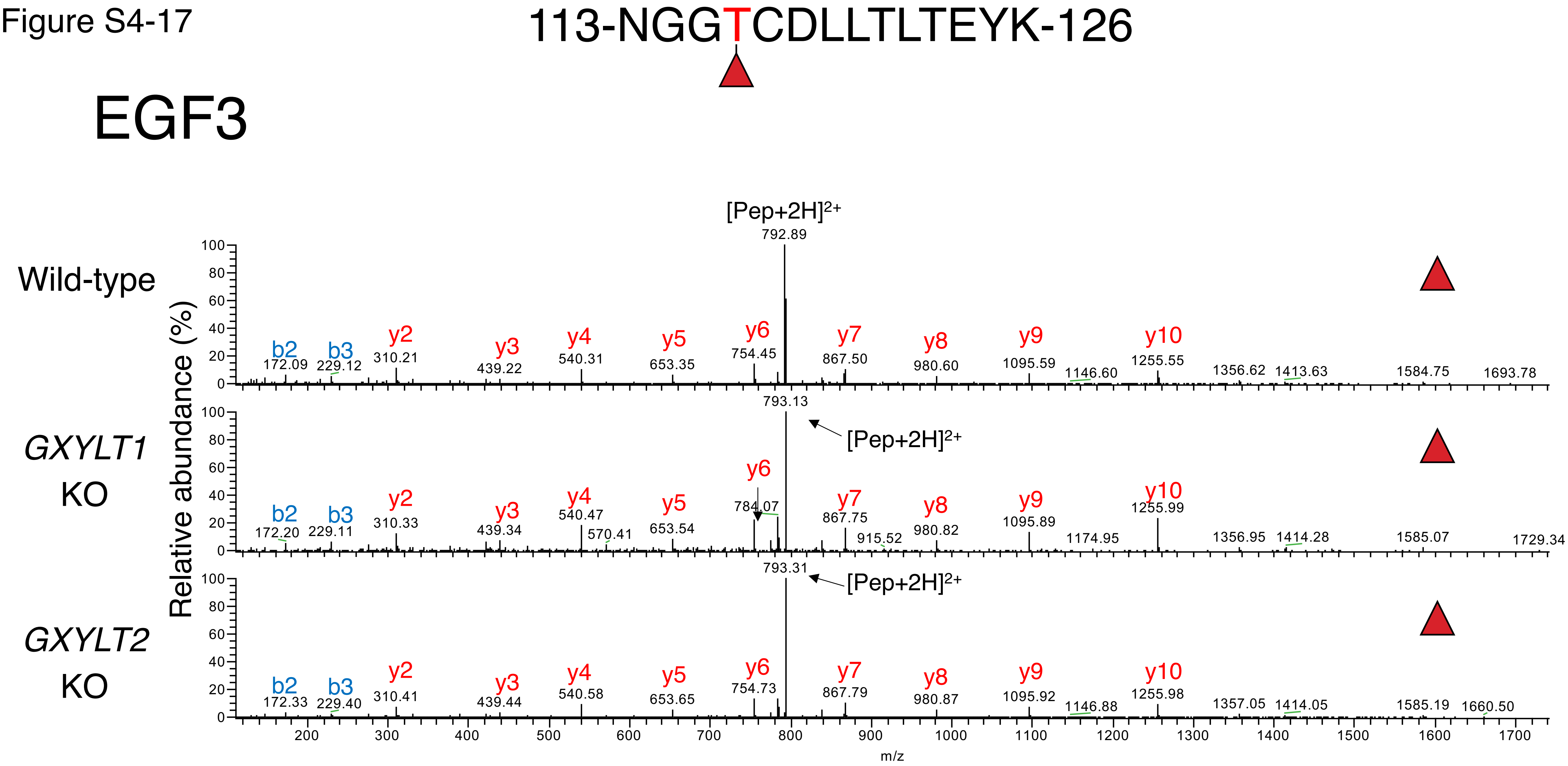

113-NGGTCDLLTLTEYK-126
EGF3
Wild-type
GXYLT1 KO
GXYLT2 KO
Relative abundance (%)
[Pep+2H]2+
792.89
793.13
793.31
b2
b3
y2
y3
y4
y5
y6
y7
y8
y9
y10
172.09
229.12
310.21
439.22
540.31
653.35
754.45
867.50
980.60
1095.59
1146.60
1255.55
1356.62
1413.63
1584.75
1693.78
172.20
229.11
310.33
439.34
540.47
570.41
653.54
784.07
867.75
915.52
980.82
1095.89
1174.95
1255.99
1356.95
1414.28
1585.07
1729.34
172.33
229.40
310.41
439.44
540.58
653.65
754.73
867.79
980.87
1095.92
1146.88
1255.98
1357.05
1414.05
1585.19
1660.50
m/z

Figure S4-18

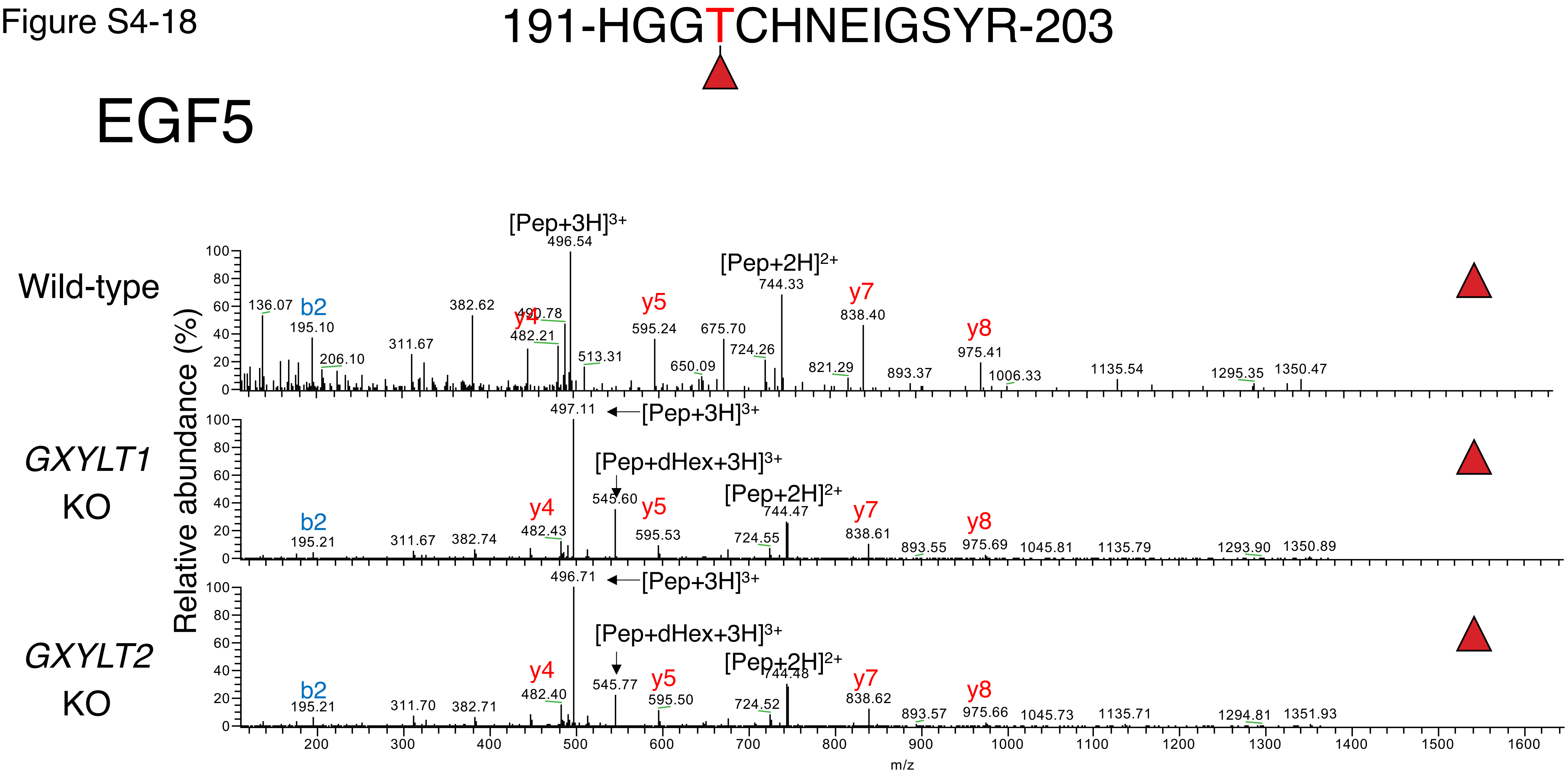

191-HGGTCHNEIGSYR-203
EGF5
Wild-type
GXYLT1 KO
GXYLT2 KO
Relative abundance (%)
m/z
[Pep+3H]3+
496.54
[Pep+2H]2+
744.33
b2
195.10
136.07
206.10
311.67
382.62
y4
482.21
490.78
513.31
y5
595.24
650.09
675.70
724.26
821.29
y7
838.40
893.37
y8
975.41
1006.33
1135.54
1295.35
1350.47
497.11
[Pep+dHex+3H]3+
545.60
744.47
195.21
311.67
382.74
482.43
595.53
724.55
838.61
893.55
975.69
1045.81
1135.79
1293.90
1350.89
496.71
545.77
744.48
195.21
311.70
382.71
482.40
595.50
724.52
838.62
893.57
975.66
1045.73
1135.71
1294.81
1351.93

Figure S4-18

1357-CLNGGTCISGPR-1368

EGF35

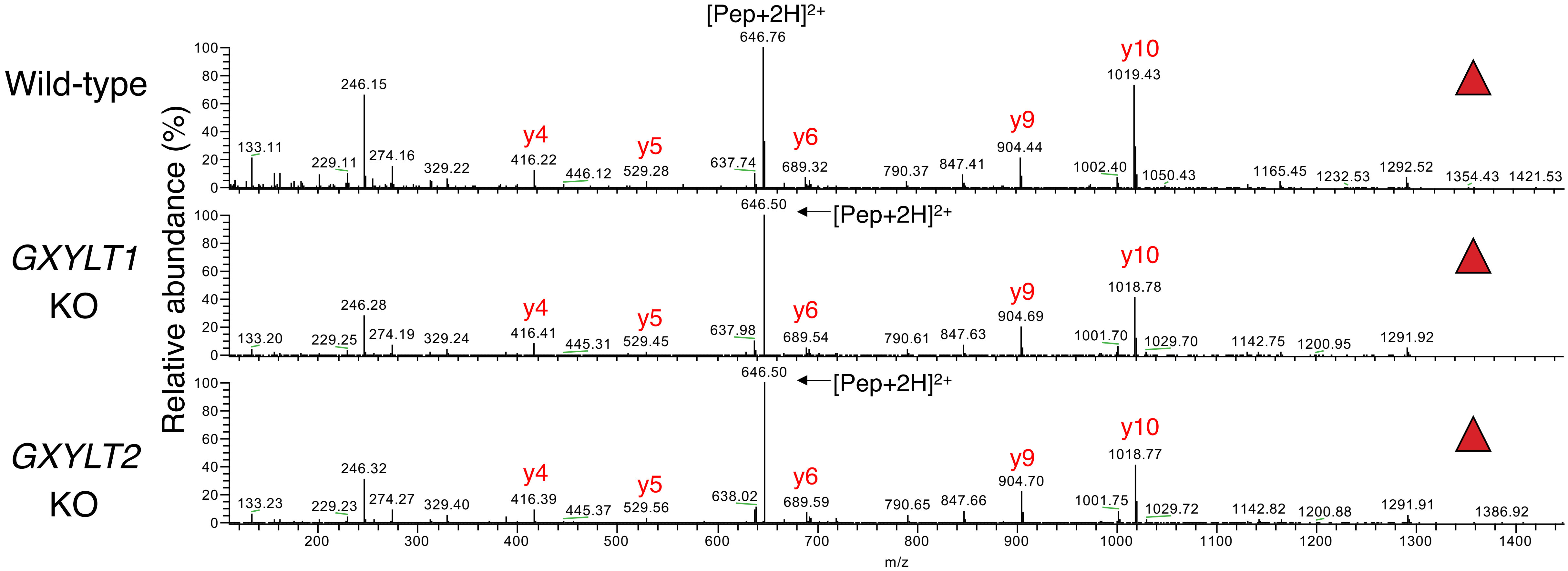

Figure S4-19

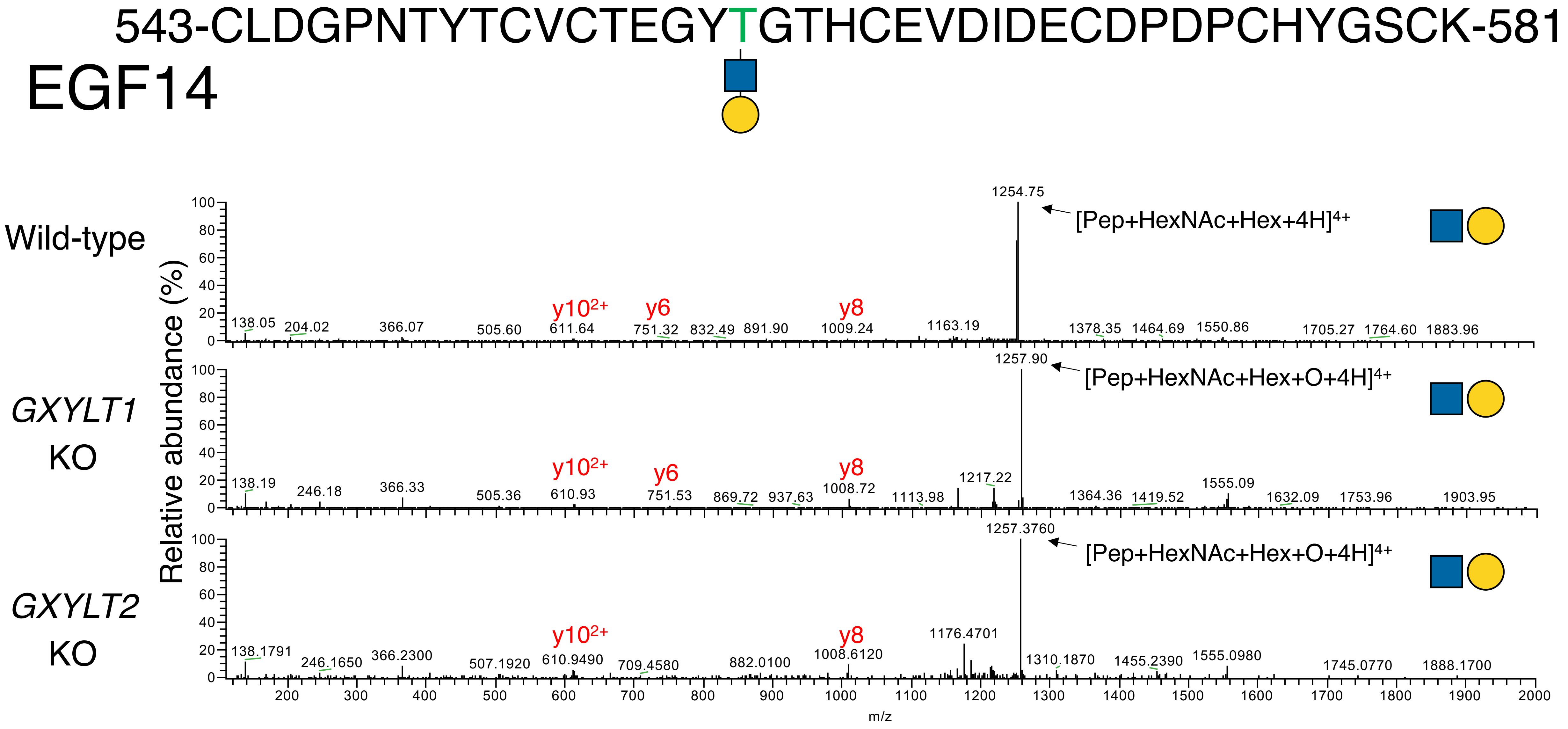

543-CLDGPNTYTCVCTEGYTGTHCEVDIDECDPDPCHYGSCK-581
EGF14
Wild-type
GXYLT1
KO
GXYLT2
KO
Relative abundance (%)
m/z
[Pep+HexNAc+Hex+4H]4+
[Pep+HexNAc+Hex+O+4H]4+
[Pep+HexNAc+Hex+O+4H]4+
1254.75
138.05
204.02
366.07
505.60
y10 2+
611.64
y6
751.32
832.49
891.90
y8
1009.24
1163.19
1378.35
1464.69
1550.86
1705.27
1764.60
1883.96
1257.90
138.19
246.18
366.33
505.36
610.93
751.53
869.72
937.63
1008.72
1113.98
1217.22
1364.36
1419.52
1555.09
1632.09
1753.96
1903.95
1257.3760
138.1791
246.1650
366.2300
507.1920
610.9490
709.4580
882.0100
1008.6120
1176.4701
1310.1870
1455.2390
1555.0980
1745.0770
1888.1700

Figure S4-20

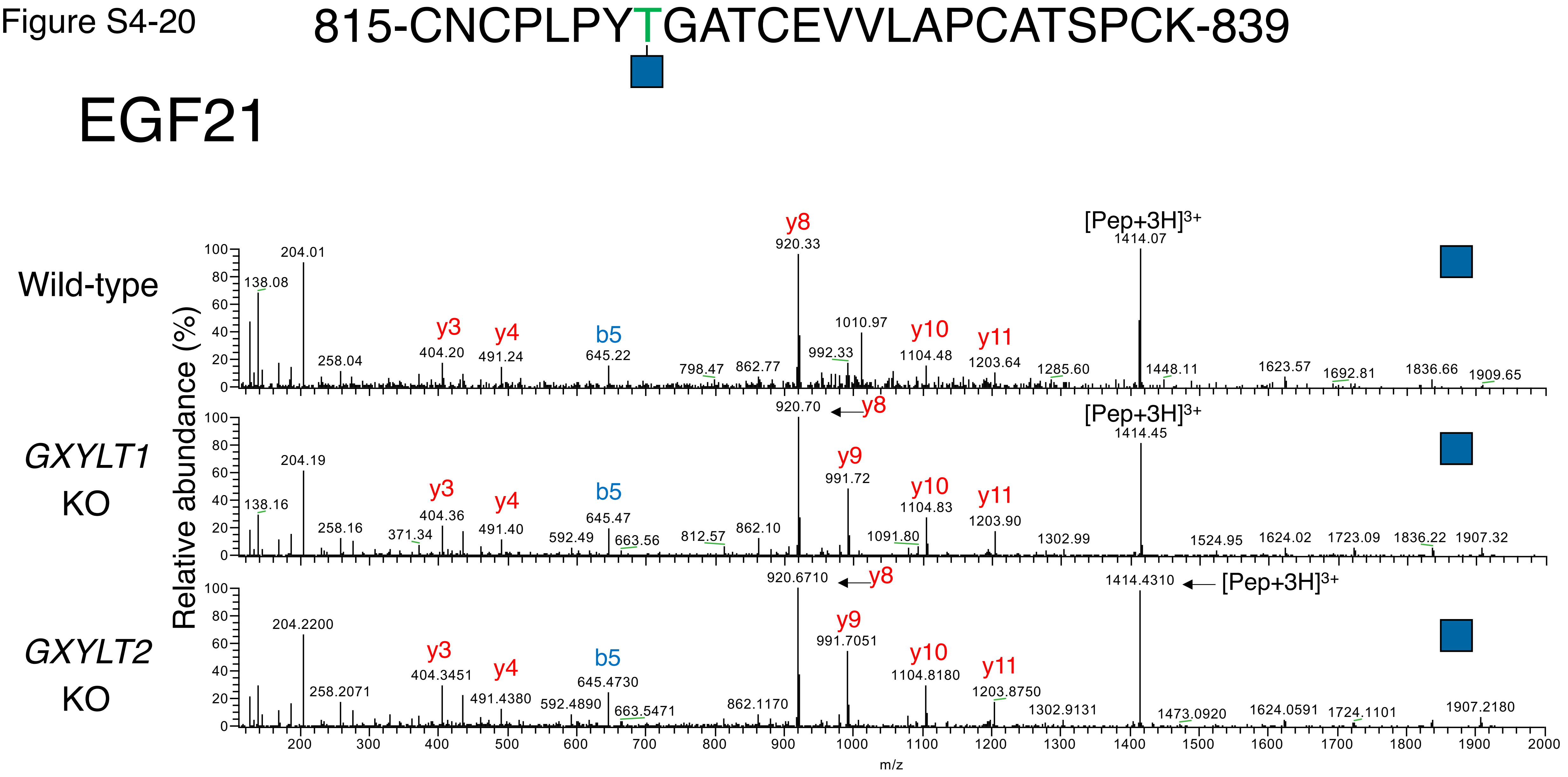

815-CNCPLPYTGATCEVVLAPCATSPCK-839
EGF21
Wild-type
GXYLT1 KO
GXYLT2 KO
Relative abundance (%)
m/z
[Pep+3H]3+
1414.07
y8
920.33
204.01
138.08
y3
404.20
y4
491.24
b5
645.22
258.04
798.47
862.77
992.33
1010.97
y10
1104.48
y11
1203.64
1285.60
1448.11
1623.57
1692.81
1836.66
1909.65
920.70
1414.45
204.19
138.16
258.16
371.34
404.36
491.40
592.49
645.47
663.56
812.57
862.10
y9
991.72
1091.80
1104.83
1203.90
1302.99
1524.95
1624.02
1723.09
1836.22
1907.32
920.6710
1414.4310
204.2200
258.2071
404.3451
491.4380
592.4890
645.4730
663.5471
862.1170
991.7051
1104.8180
1203.8750
1302.9131
1473.0920
1624.0591
1724.1101
1907.2180

Figure S4-21

EGF23

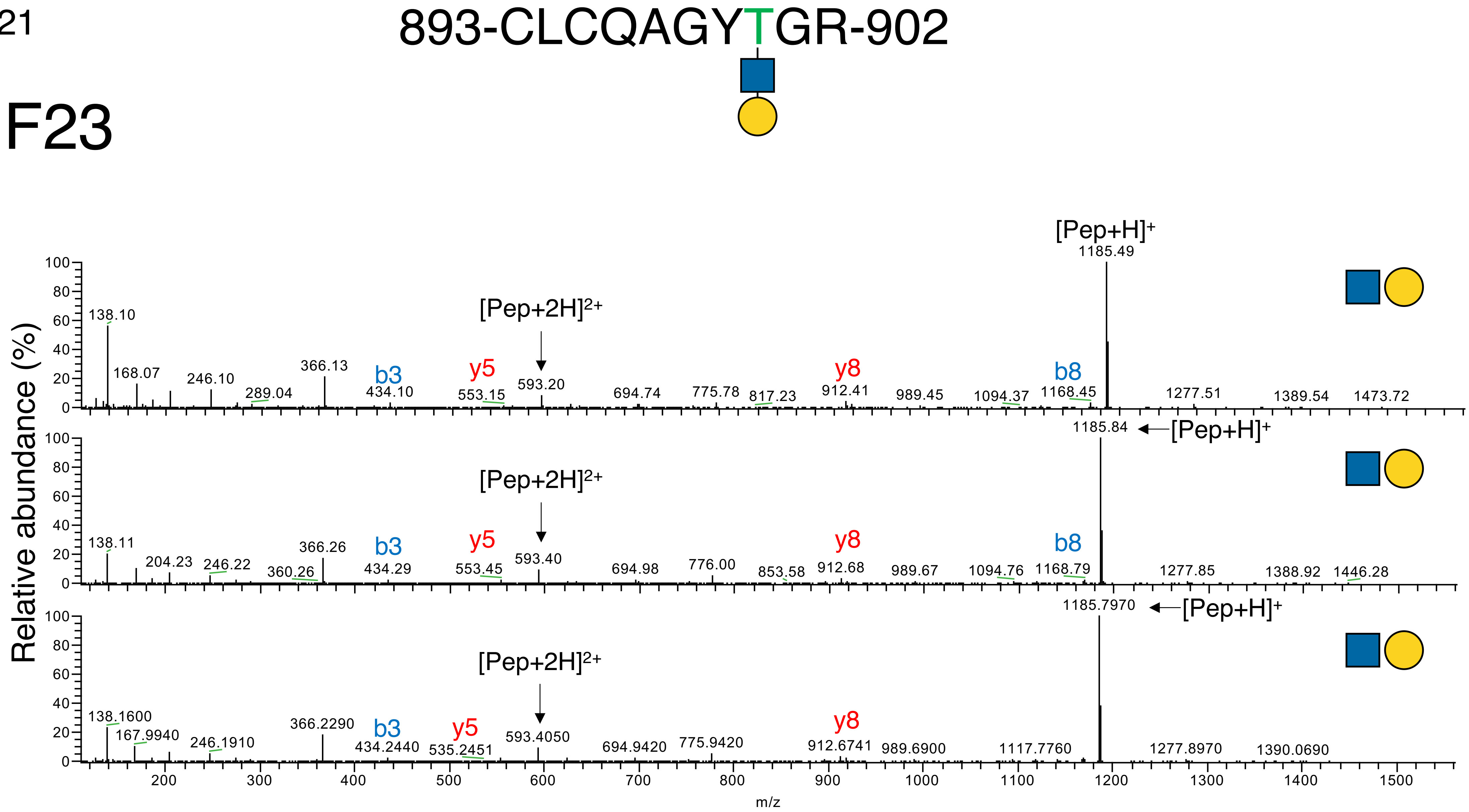

Figure S4-22

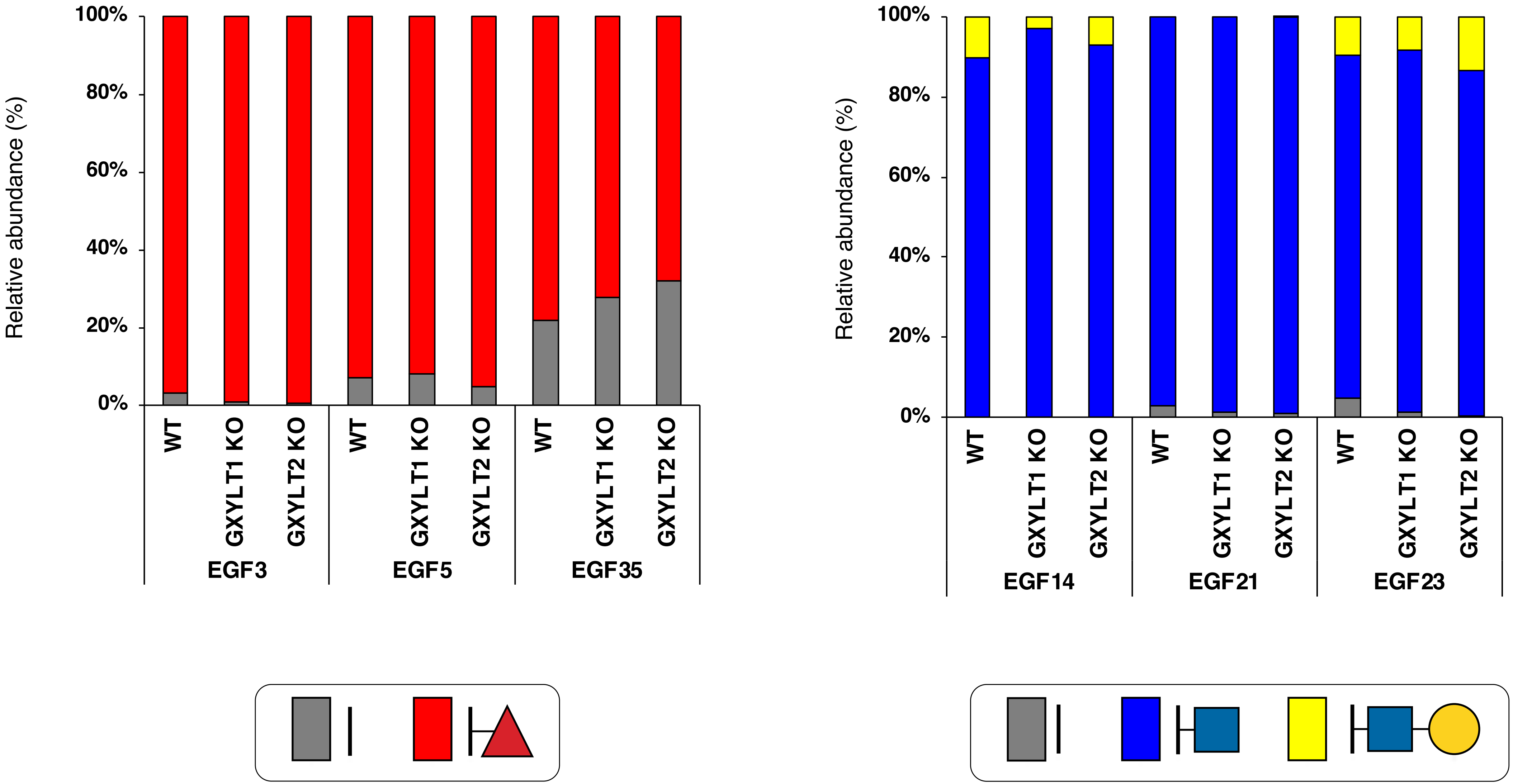
100%
80%
60%
40%
20%
0%
Relative abundance (%)
WT
GXYLT1 KO
GXYLT2 KO
EGF3
EGF5
EGF35
EGF14
EGF21
EGF23

Figure S4. MS/MS spectra of (glyco)peptides from NOTCH1.

Samples were generated in wild-type control HEK293T cells (*top*), *GXYLT1* KO cells (*middle*), or *GXYLT2* KO cells (*bottom*), transfected with the plasmids encoding mouse NOTCH1 ECDs as described in the Experimental procedure section. The data in Figures S4-1 through 12, 14, and 16-22 are derived from the analysis of mouse NOTCH1 EGF1-36. The data in Figures S4-13 and 15 are derived from the analysis of mouse NOTCH1 EGF19-36. MS/MS spectra confirmed the identity of (glyco)peptides based on the presence of peptide-specific b- and y-ions and neutral loss of predicted glycans. Amino acid sequences of the (glyco)peptides are shown at the top of the figure. MS/MS spectra of the (glyco)peptides modified with the most elongated glycoform derived from each cell clone are shown. The MS/MS spectra used for the analysis of *O*-Glc, *O*-Fuc, or *O*-GlcNAc glycans are shown in Figures S4-1 to 16, S4-17 to 19, or S4-20 to 22, respectively. The sequence of (glyco)peptides, the predicted and measured m/z, and the charge state are summarized in Tables S7 to S9. Figure S4-23 shows the stoichiometry of *O*-Fuc glycans on EGF3, EGF5, and EGF35 in NOTCH1 (*left*) and *O*-GlcNAc glycans on EGF14, EGF21, and EGF23 in NOTCH1 (*right*). Black bar, naked peptide; red triangle, Fuc; blue square, GlcNAc; yellow circle, galactose.

Figure S5-1

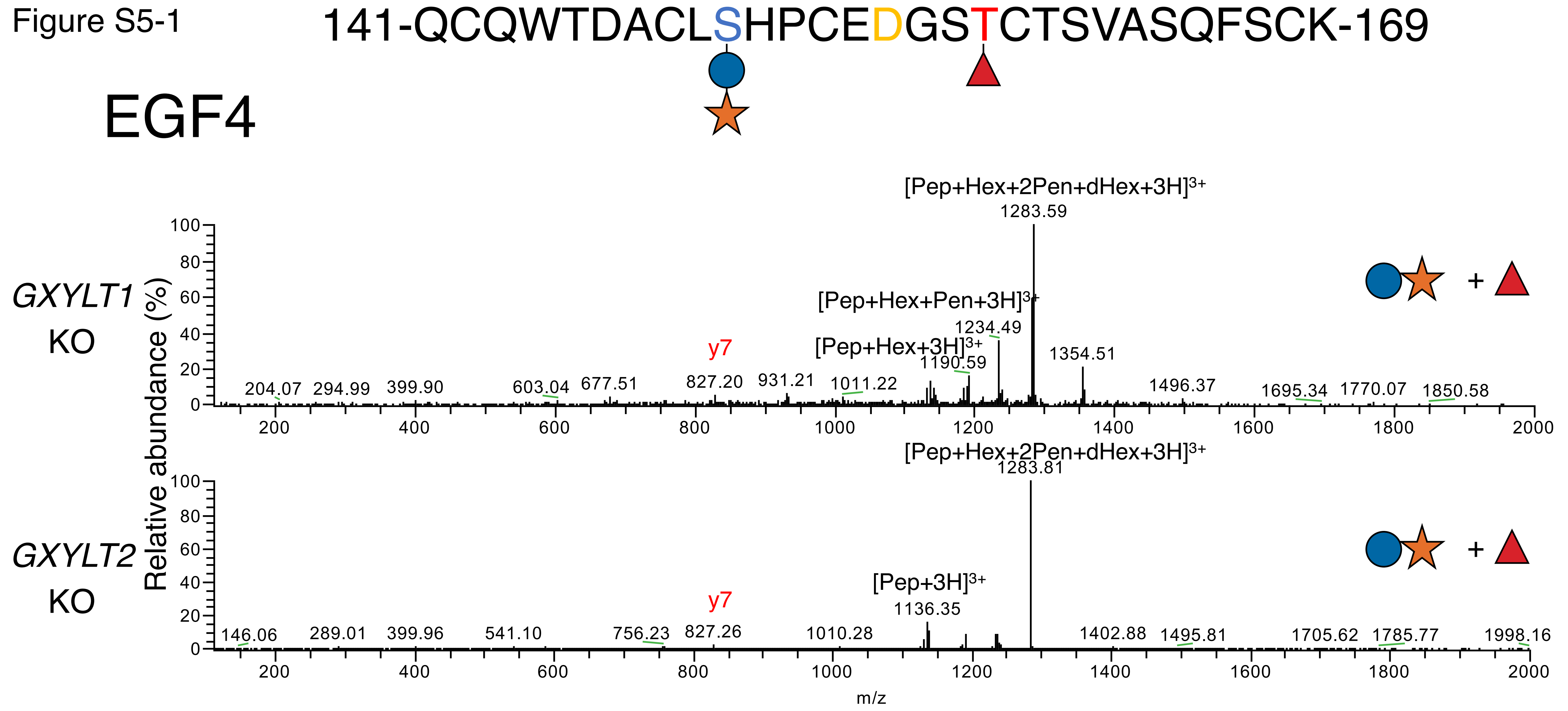

141-QCQWTDACLSHPCEDGSTCTSVASQFSCK-169
EGF4
GXYLT1
KO
GXYLT2
KO
Relative abundance (%)
m/z
[Pep+Hex+2Pen+dHex+3H]3+
1283.59
[Pep+Hex+Pen+3H]3+
1234.49
[Pep+Hex+3H]3+
1190.59
1354.51
y7
204.07
294.99
399.90
603.04
677.51
827.20
931.21
1011.22
1496.37
1695.34
1770.07
1850.58
[Pep+Hex+2Pen+dHex+3H]3+
1283.81
[Pep+3H]3+
1136.35
y7
146.06
289.01
399.96
541.10
756.23
827.26
1010.28
1402.88
1495.81
1705.62
1785.77
1998.16

Figure S5-2

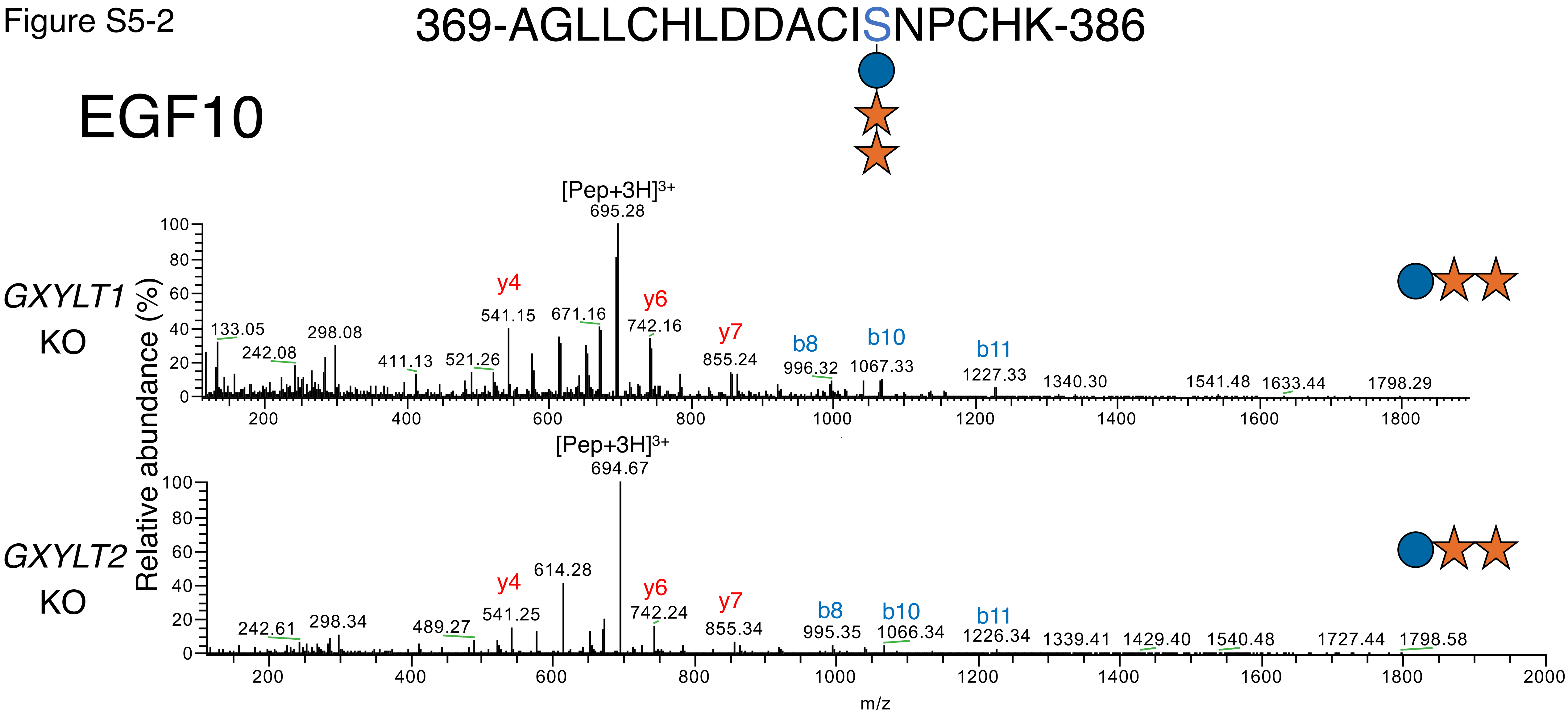

369-AGLLCHLDDACISNPCHK-386
EGF10
GXYLT1 KO
GXYLT2 KO
Relative abundance (%)
m/z
[Pep+3H]3+
695.28
694.67
y4
y6
y7
b8
b10
b11
133.05
242.08
298.08
411.13
521.26
541.15
671.16
742.16
855.24
996.32
1067.33
1227.33
1340.30
1541.48
1633.44
1798.29
242.61
298.34
489.27
541.25
614.28
742.24
855.34
995.35
1066.34
1226.34
1339.41
1429.40
1540.48
1727.44
1798.58

Figure S5-3

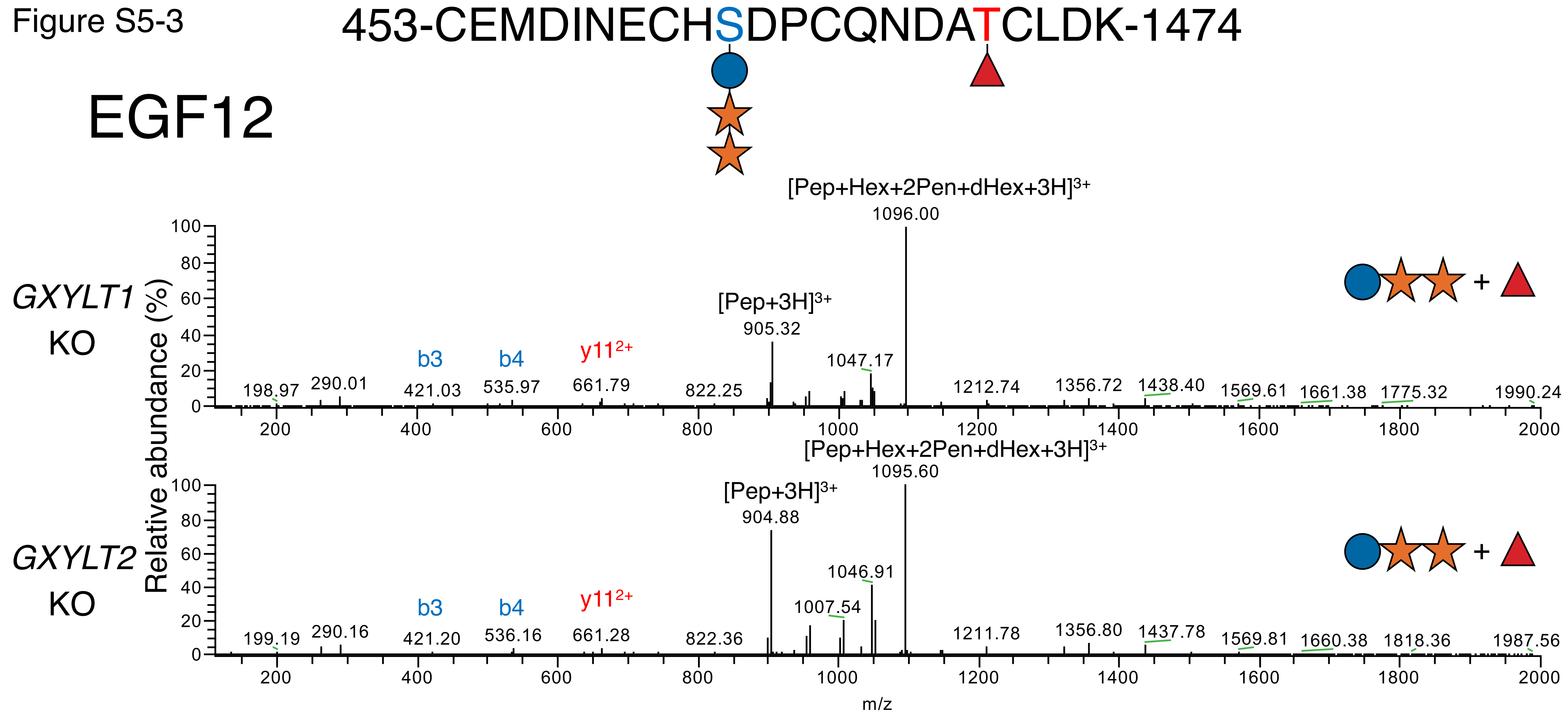
453-CEMDINECHSDPCQNDATCLDK-1474
EGF12
GXYLT1 KO
GXYLT2 KO
Relative abundance (%)
m/z
[Pep+Hex+2Pen+dHex+3H]3+
1096.00
[Pep+3H]3+
905.32
1047.17
b3
b4
y11 2+
198.97
290.01
421.03
535.97
661.79
822.25
1212.74
1356.72
1438.40
1569.61
1661.38
1775.32
1990.24
[Pep+Hex+2Pen+dHex+3H]3+
1095.60
[Pep+3H]3+
904.88
1046.91
1007.54
199.19
290.16
421.20
536.16
661.28
822.36
1211.78
1356.80
1437.78
1569.81
1660.38
1818.36
1987.56

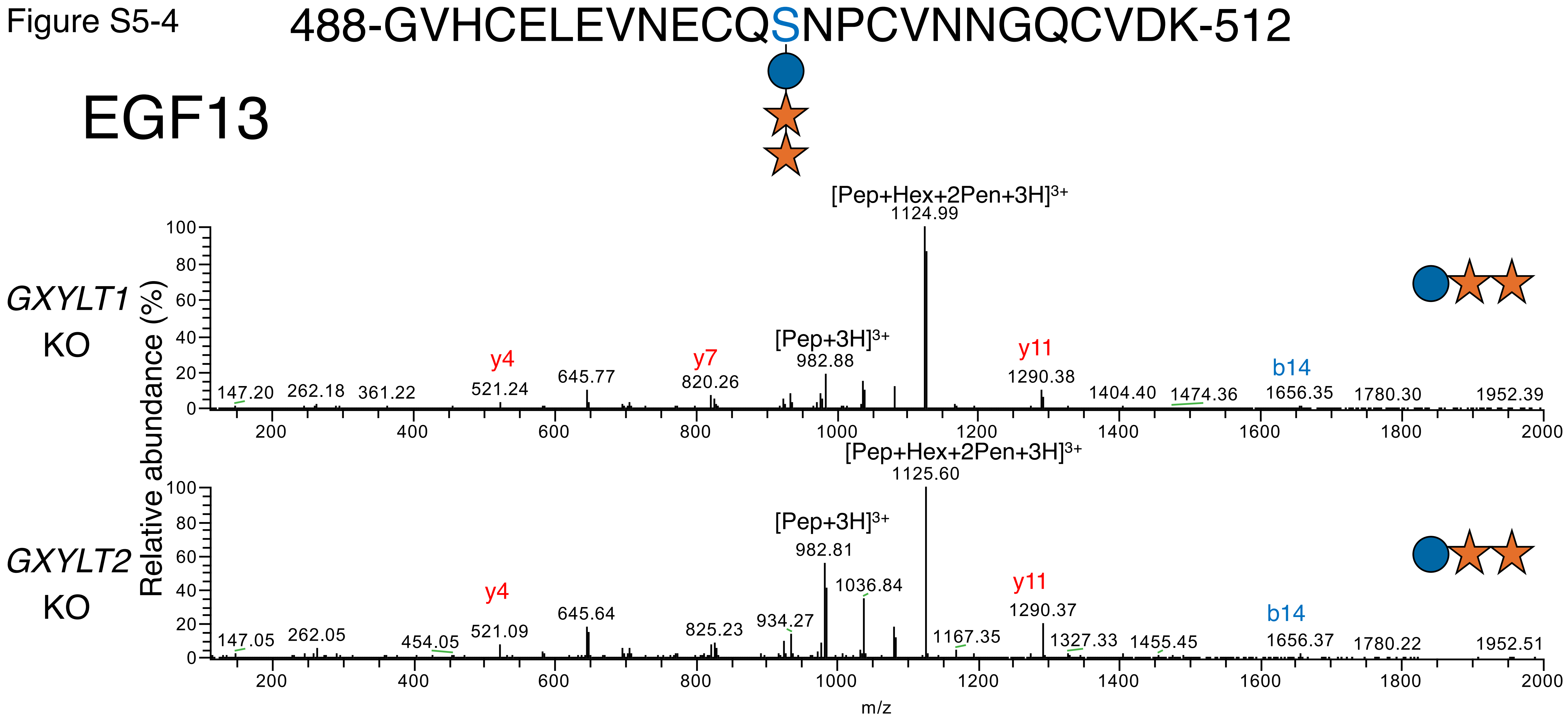

Figure S5-4
488-GVHCELEVNECQSNPCVNNGQCVDK-512
EGF13
GXYLT1
KO
GXYLT2
KO
Relative abundance (%)
[Pep+Hex+2Pen+3H]3+
1124.99
[Pep+3H]3+
982.88
y4
521.24
y7
820.26
y11
1290.38
b14
1656.35
147.20
262.18
361.22
645.77
1404.40
1474.36
1780.30
1952.39
[Pep+Hex+2Pen+3H]3+
1125.60
[Pep+3H]3+
982.81
1036.84
y4
521.09
y11
1290.37
b14
1656.37
147.05
262.05
454.05
645.64
825.23
934.27
1167.35
1327.33
1455.45
1780.22
1952.51
m/z

Figure S5-5

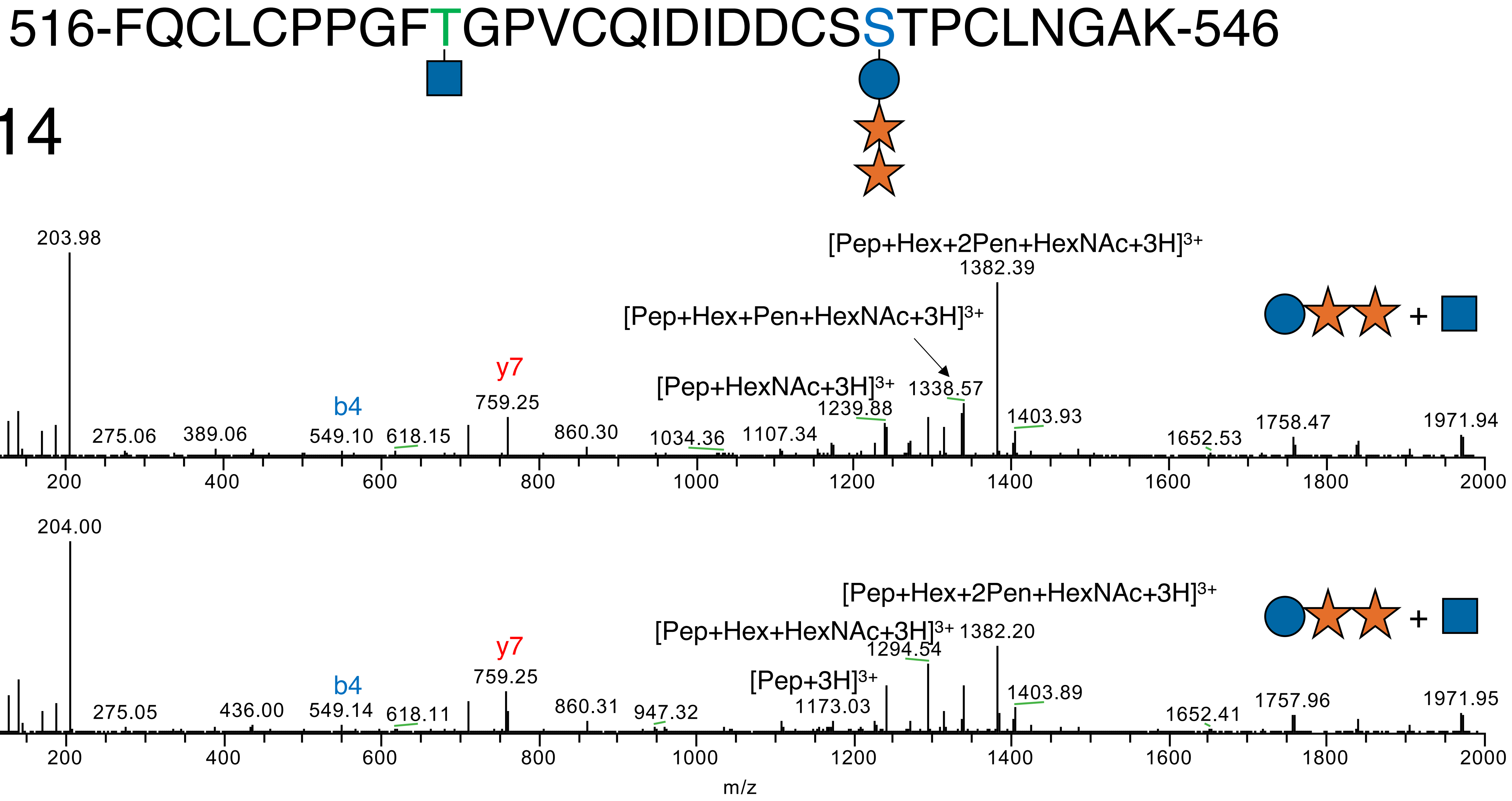

516-FQCLCPPGFTGPVCQIDIDDCSSTPCLNGAK-546
EGF14
GXYLT1 KO
GXYLT2 KO
Relative abundance (%)
m/z
203.98
[Pep+Hex+2Pen+HexNAc+3H]3+
1382.39
[Pep+Hex+Pen+HexNAc+3H]3+
y7
759.25
[Pep+HexNAc+3H]3+
1338.57
b4
1239.88
1403.93
1758.47
1971.94
275.06
389.06
549.10
618.15
860.30
1034.36
1107.34
1652.53
204.00
[Pep+Hex+2Pen+HexNAc+3H]3+
[Pep+Hex+HexNAc+3H]3+
1382.20
1294.54
[Pep+3H]3+
1403.89
1173.03
1757.96
1971.95
275.05
436.00
549.14
618.11
860.31
947.32
1652.41

Figure S5-6

EGF16

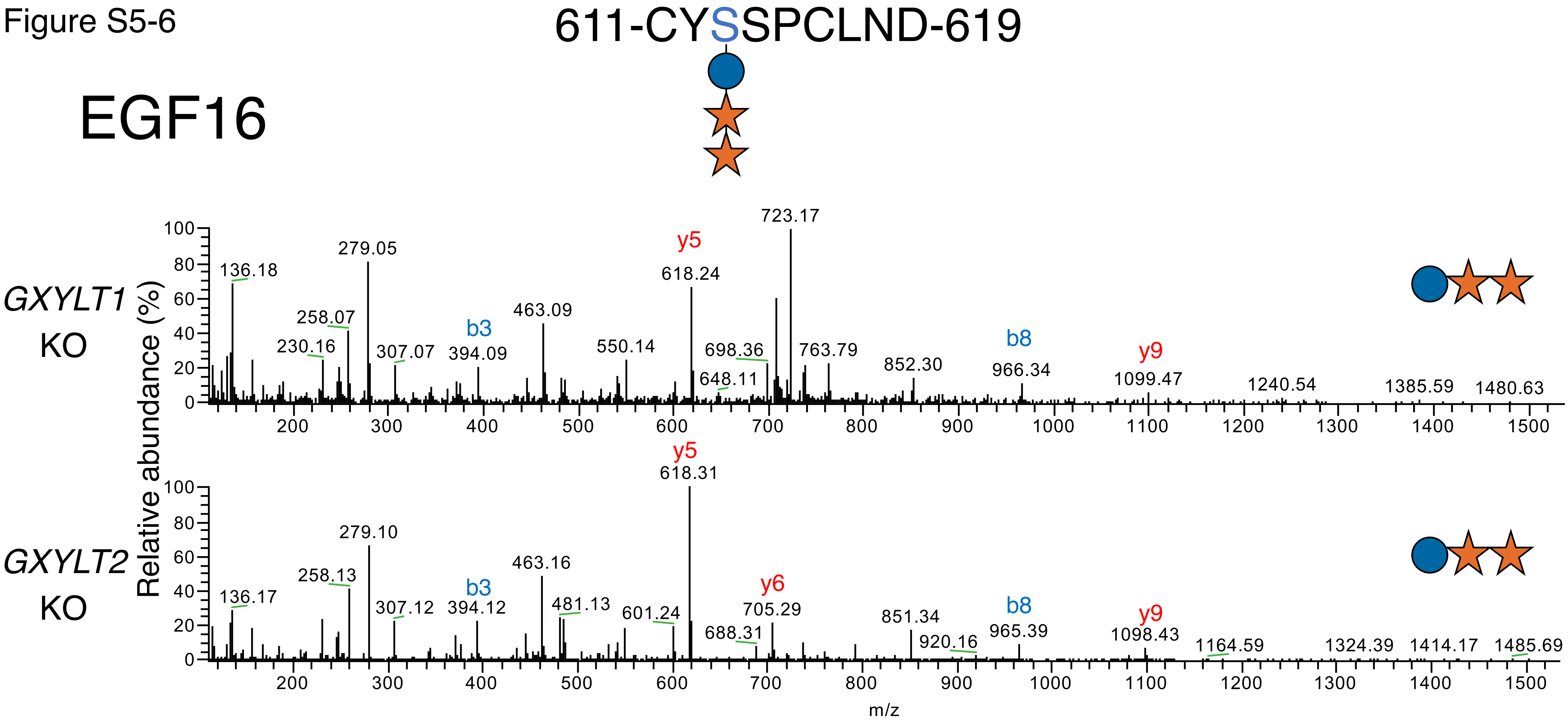

Figure S5-7

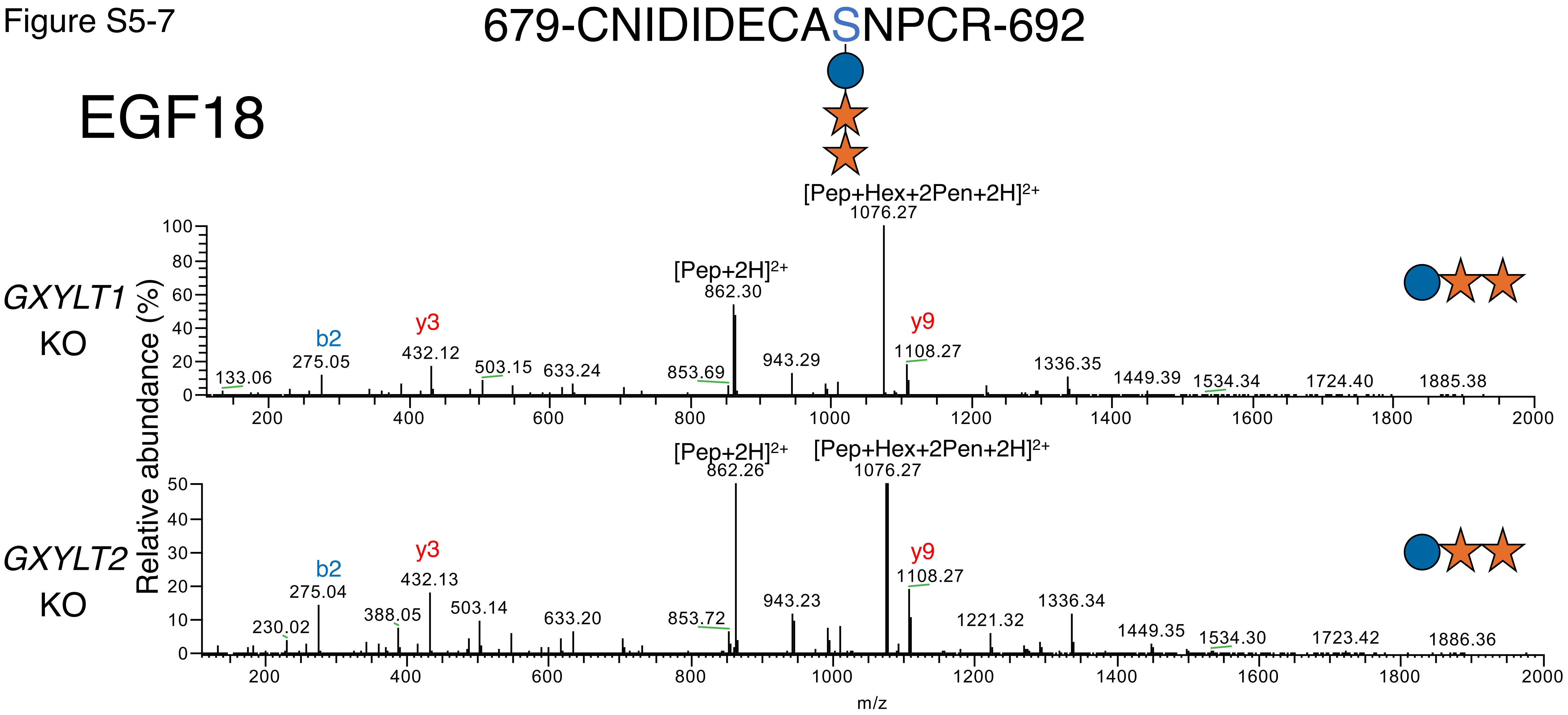
679-CNIDIDECASNPCR-692
EGF18
GXYLT1 KO
GXYLT2 KO
Relative abundance (%)
[Pep+Hex+2Pen+2H]2+
1076.27
[Pep+2H]2+
862.30
b2
275.05
y3
432.12
y9
1108.27
133.06
503.15
633.24
853.69
943.29
1336.35
1449.39
1534.34
1724.40
1885.38
[Pep+2H]2+
862.26
[Pep+Hex+2Pen+2H]2+
1076.27
b2
275.04
y3
432.13
y9
1108.27
230.02
388.05
503.14
633.20
853.72
943.23
1221.32
1336.34
1449.35
1534.30
1723.42
1886.36
m/z

Figure S5-8

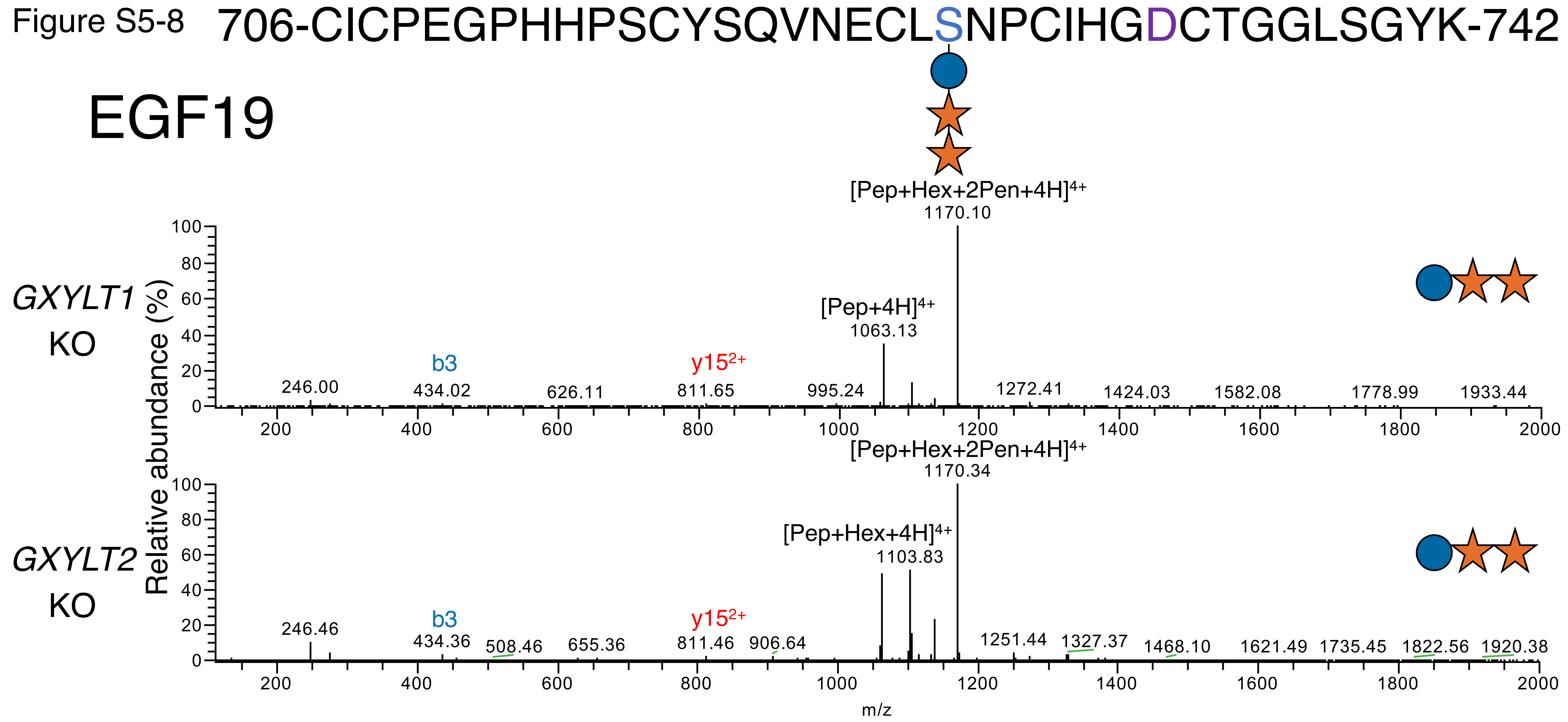
706-CICPEGPHHPSCYSQVNECLSNPCIHGDCTGGLSGYK-742
EGF19
GXYLT1 KO
GXYLT2 KO
Relative abundance (%)
m/z
[Pep+Hex+2Pen+4H]4+
1170.10
[Pep+4H]4+
1063.13
b3
434.02
y15 2+
811.65
246.00
626.11
995.24
1272.41
1424.03
1582.08
1778.99
1933.44
[Pep+Hex+2Pen+4H]4+
1170.34
[Pep+Hex+4H]4+
1103.83
b3
434.36
y15 2+
811.46
246.46
508.46
655.36
906.64
1251.44
1327.37
1468.10
1621.49
1735.45
1822.56
1920.38

Figure S5-9

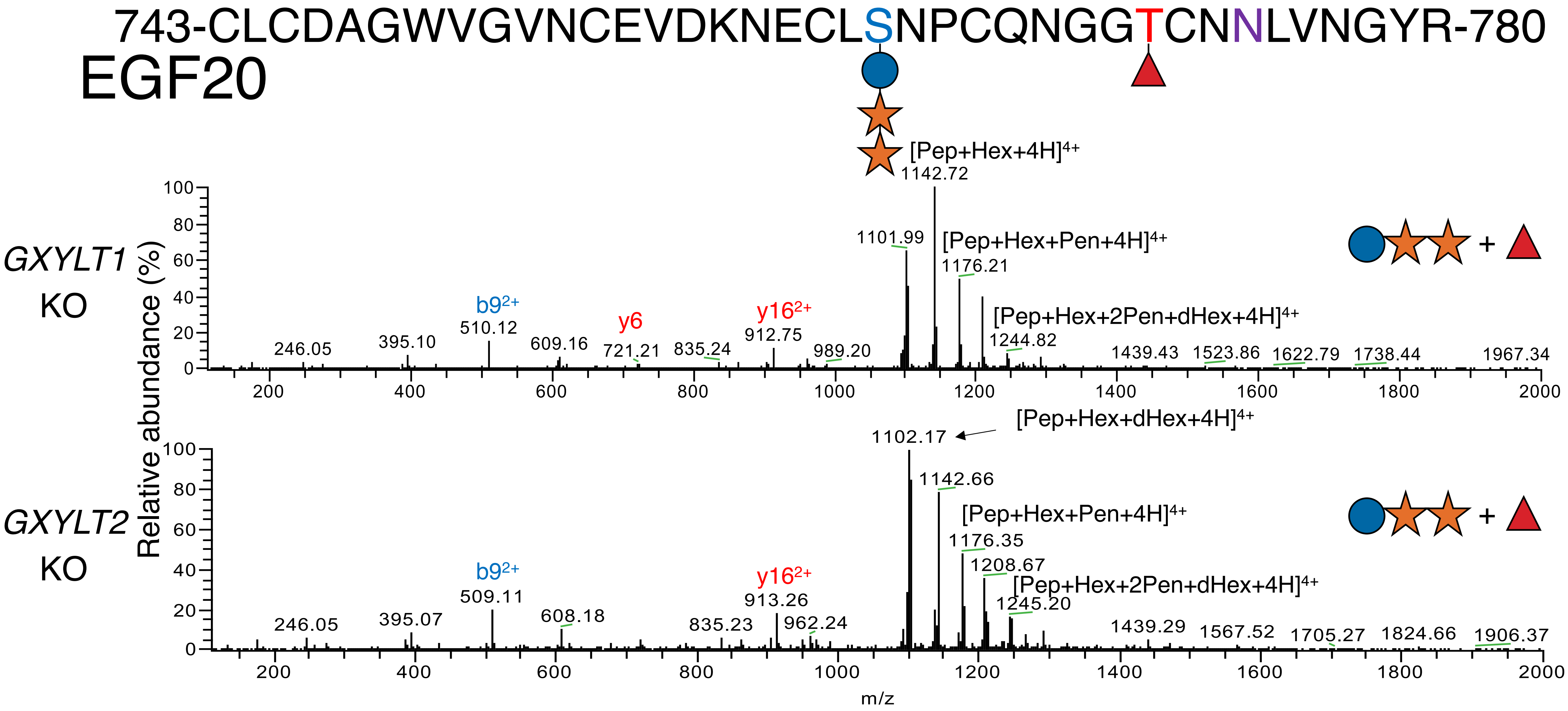

743-CLCDAGWVGVNCEVDKNECLSNPCQNGGTCNNLVNGYR-780
EGF20
GXYLT1 KO
GXYLT2 KO
Relative abundance (%)
m/z
[Pep+Hex+4H]4+
1142.72
1101.99
[Pep+Hex+Pen+4H]4+
1176.21
[Pep+Hex+2Pen+dHex+4H]4+
1244.82
b92+
510.12
y6
721.21
y162+
912.75
246.05
395.10
609.16
835.24
989.20
1439.43
1523.86
1622.79
1738.44
1967.34
[Pep+Hex+dHex+4H]4+
1102.17
1142.66
[Pep+Hex+Pen+4H]4+
1176.35
1208.67
[Pep+Hex+2Pen+dHex+4H]4+
1245.20
b92+
509.11
y162+
913.26
246.05
395.07
608.18
835.23
962.24
1439.29
1567.52
1705.27
1824.66
1906.37

Figure S5-10

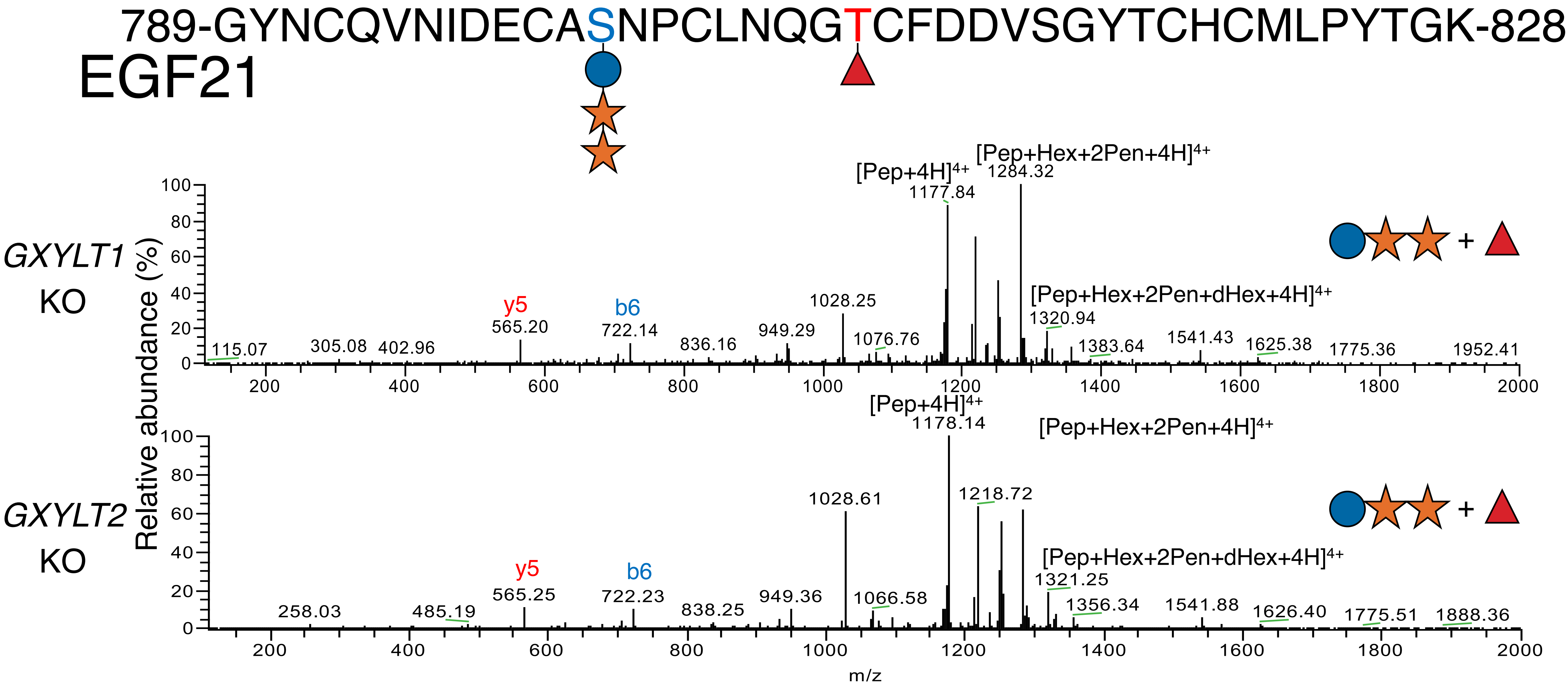

789-GYNCQVNIDECASNPCLNQGTCFDDVSGYTCHCMLPYTGK-828
EGF21
GXYLT1 KO
GXYLT2 KO
Relative abundance (%)
[Pep+4H]4+
1177.84
[Pep+Hex+2Pen+4H]4+
1284.32
[Pep+Hex+2Pen+dHex+4H]4+
1320.94
y5
565.20
b6
722.14
115.07
305.08
402.96
836.16
949.29
1028.25
1076.76
1383.64
1541.43
1625.38
1775.36
1952.41
[Pep+4H]4+
1178.14
[Pep+Hex+2Pen+4H]4+
1218.72
1028.61
[Pep+Hex+2Pen+dHex+4H]4+
1321.25
1356.34
y5
565.25
b6
722.23
258.03
485.19
838.25
949.36
1066.58
1541.88
1626.40
1775.51
1888.36
m/z

Figure S5-11

870-CTVDVDECISK-880

EGF23

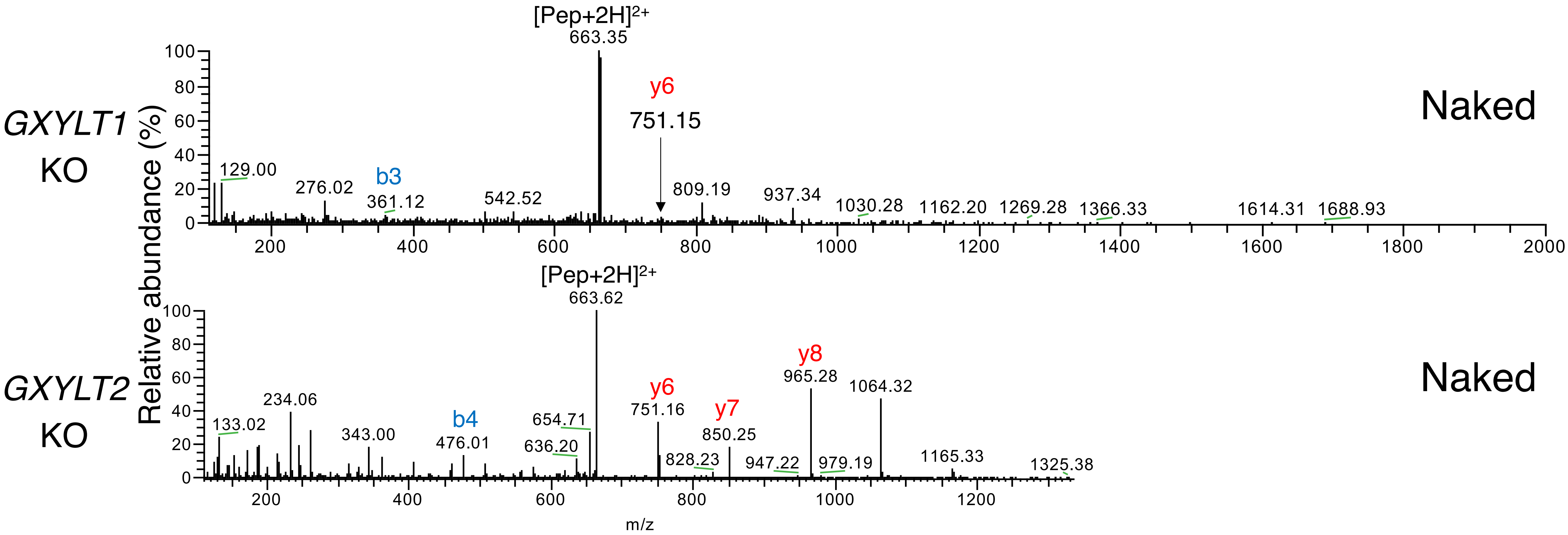

Figure S5-12

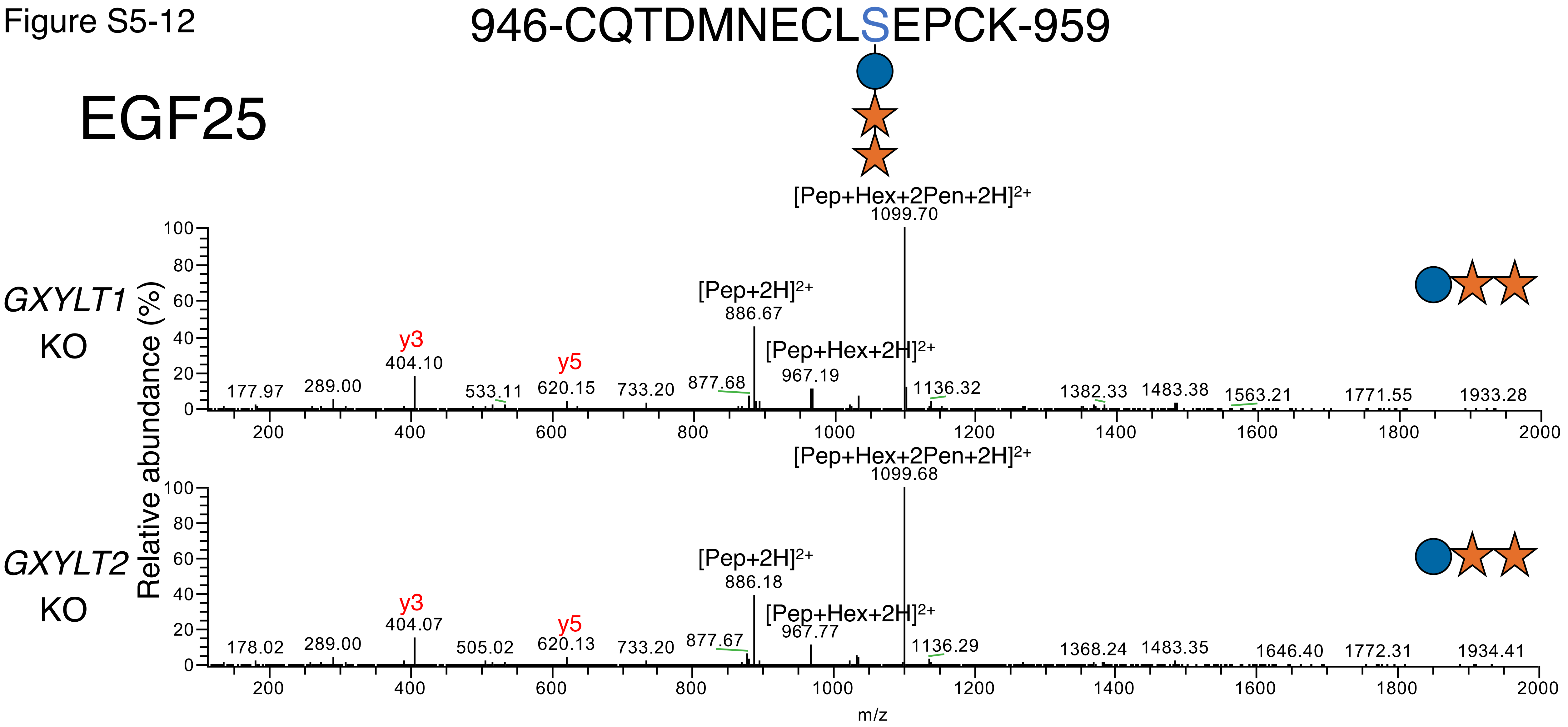

946-CQTDMNECLSEPCK-959
EGF25
GXYLT1
KO
GXYLT2
KO
Relative abundance (%)
[Pep+Hex+2Pen+2H]2+
1099.70
[Pep+2H]2+
886.67
[Pep+Hex+2H]2+
967.19
y3
404.10
y5
620.15
177.97
289.00
533.11
733.20
877.68
1136.32
1382.33
1483.38
1563.21
1771.55
1933.28
[Pep+Hex+2Pen+2H]2+
1099.68
[Pep+2H]2+
886.18
[Pep+Hex+2H]2+
967.77
y3
404.07
y5
620.13
178.02
289.00
505.02
733.20
877.67
1136.29
1368.24
1483.35
1646.40
1772.31
1934.41
m/z

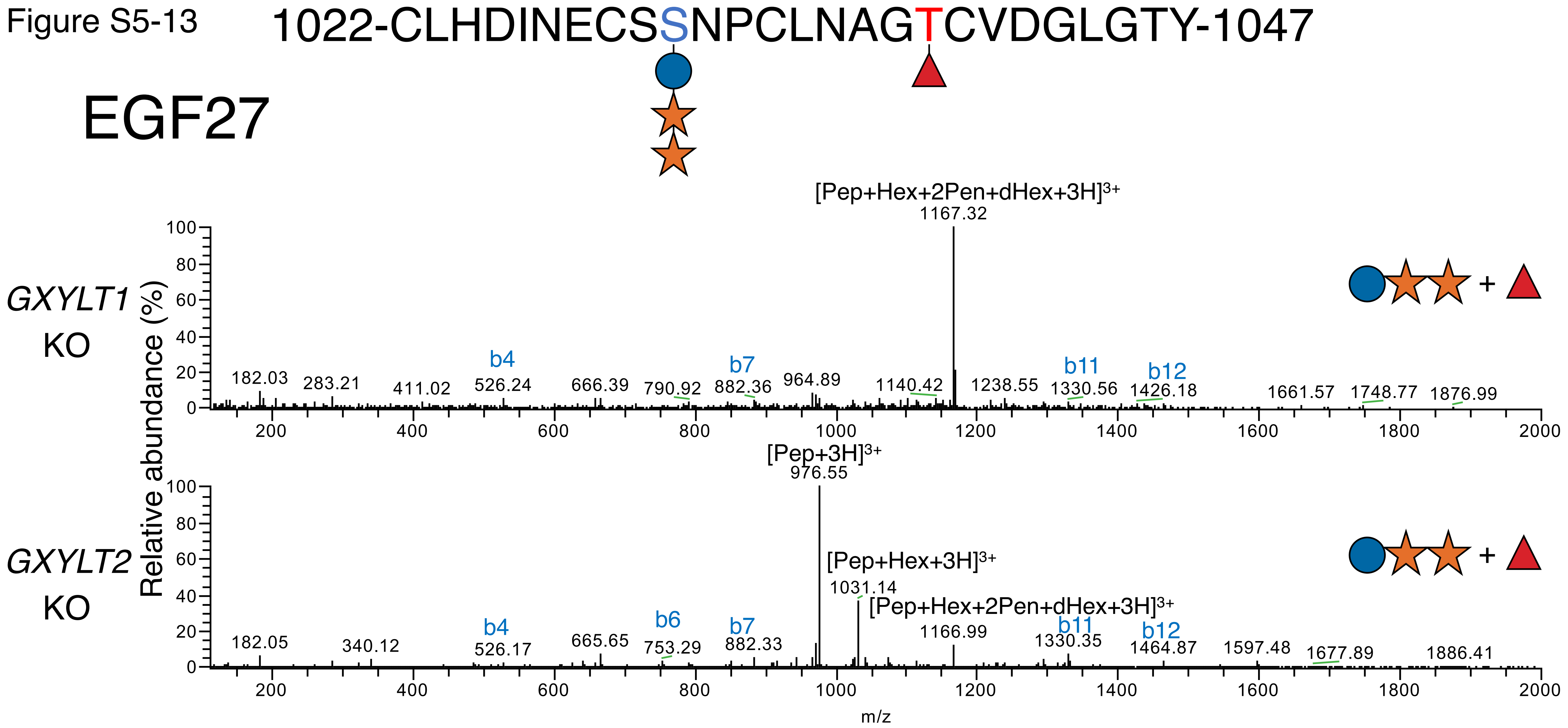
Figure S5-13
1022-CLHDINECSSNPCLNAGTCVDGLGTY-1047
EGF27
GXYLT1 KO
GXYLT2 KO
Relative abundance (%)
m/z
[Pep+Hex+2Pen+dHex+3H]3+
1167.32
182.03
283.21
411.02
b4
526.24
666.39
790.92
b7
882.36
964.89
1140.42
1238.55
b11
1330.56
b12
1426.18
1661.57
1748.77
1876.99
[Pep+3H]3+
976.55
[Pep+Hex+3H]3+
1031.14
[Pep+Hex+2Pen+dHex+3H]3+
182.05
340.12
526.17
665.65
b6
753.29
882.33
1166.99
1330.35
1464.87
1597.48
1677.89
1886.41

Figure S5-14

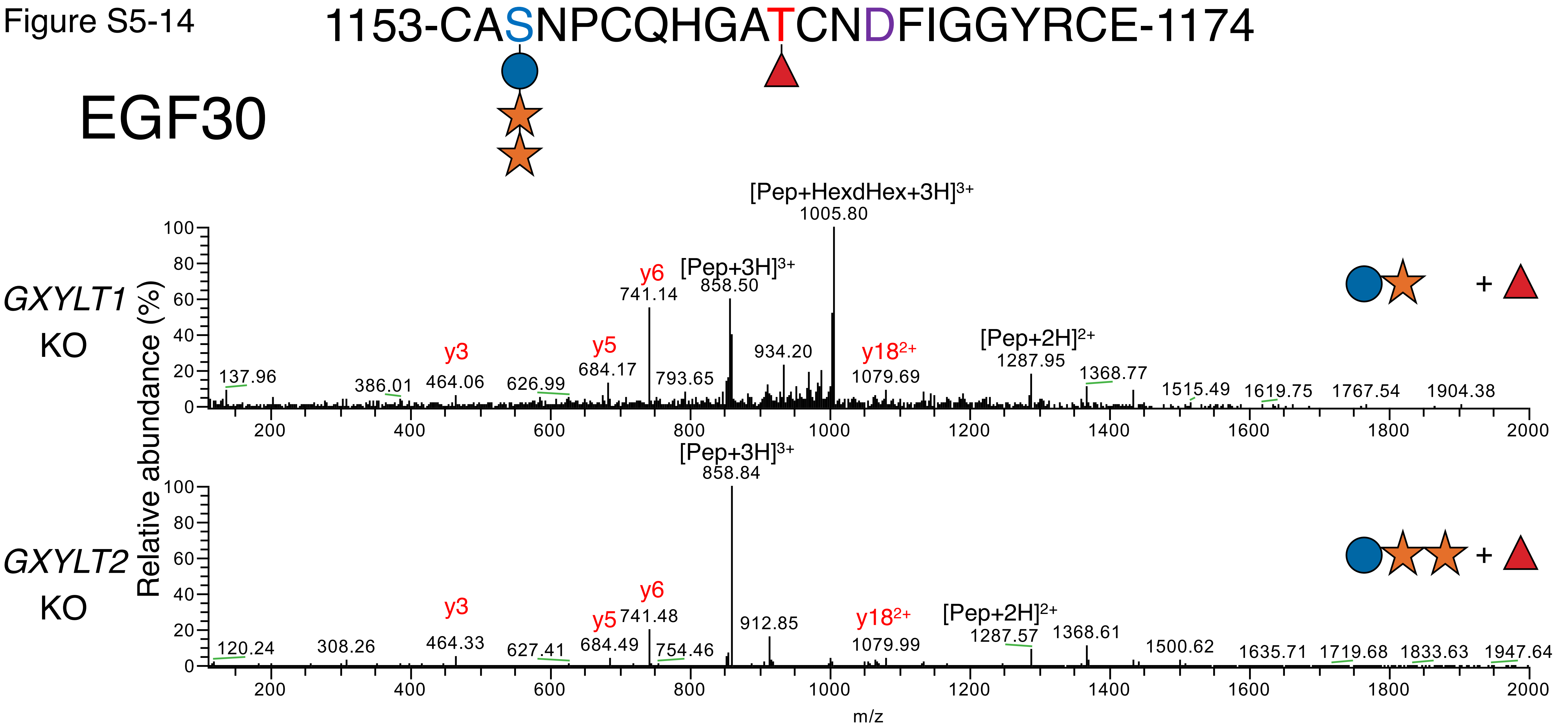
1153-CASNPCQHGATCNDFIGGYRCE-1174
EGF30
GXYLT1 KO
GXYLT2 KO
Relative abundance (%)
m/z
[Pep+HexdHex+3H]3+
1005.80
[Pep+3H]3+
858.50
y6
741.14
y5
684.17
y3
464.06
y18 2+
1079.69
[Pep+2H]2+
1287.95
137.96
386.01
626.99
793.65
934.20
1368.77
1515.49
1619.75
1767.54
1904.38
[Pep+3H]3+
858.84
y6
741.48
y5
684.49
y3
464.33
y18 2+
1079.99
[Pep+2H]2+
1287.57
120.24
308.26
627.41
754.46
912.85
1368.61
1500.62
1635.71
1719.68
1833.63
1947.64

Figure S5-15

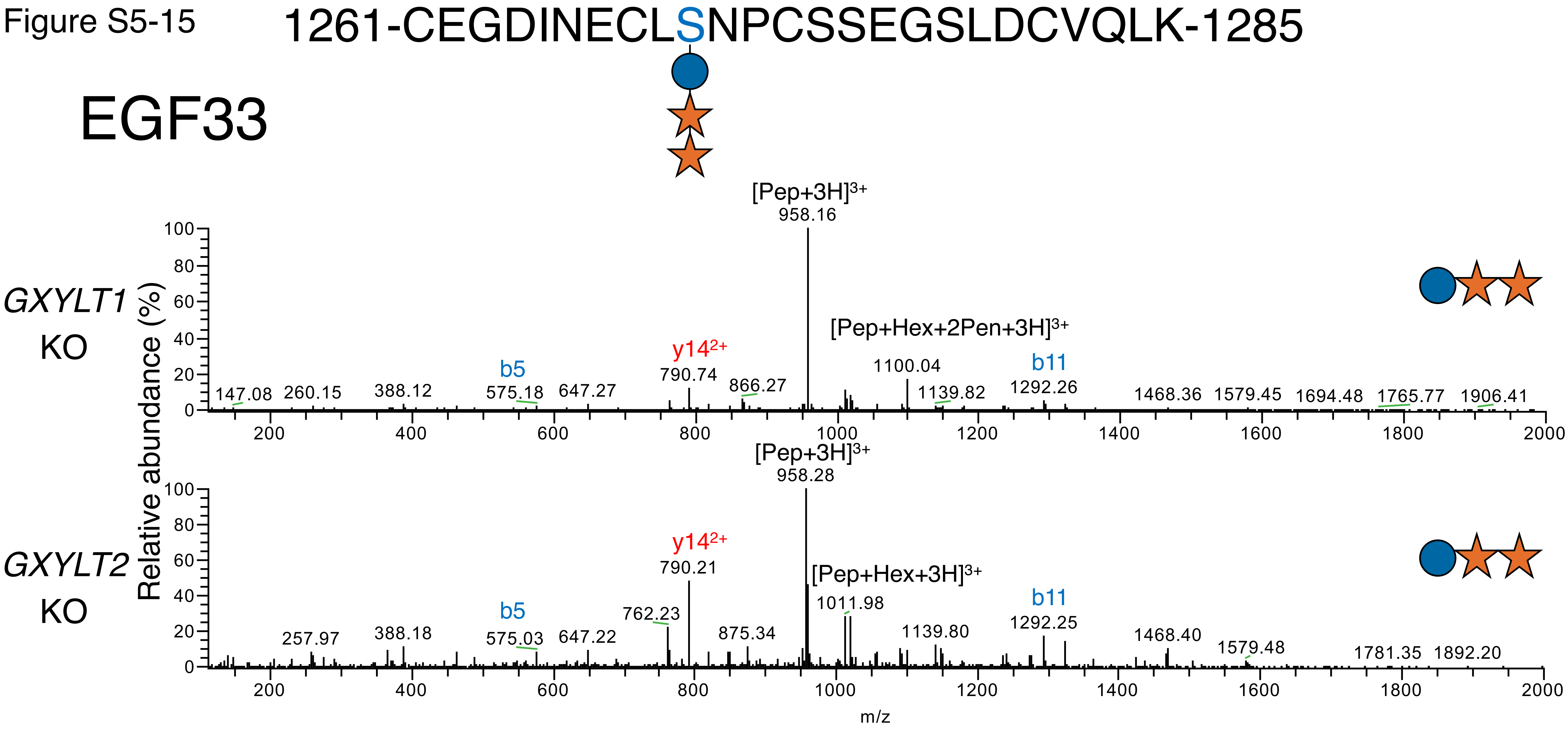

1261-CEGDINECLSNPCSSEGSLDCVQLK-1285
EGF33
GXYLT1 KO
GXYLT2 KO
Relative abundance (%)
m/z
[Pep+3H]3+
958.16
[Pep+Hex+2Pen+3H]3+
y14 2+
790.74
b5
575.18
b11
1292.26
147.08
260.15
388.12
647.27
866.27
1100.04
1139.82
1468.36
1579.45
1694.48
1765.77
1906.41
[Pep+3H]3+
958.28
[Pep+Hex+3H]3+
y14 2+
790.21
b5
575.03
b11
1292.25
257.97
388.18
647.22
762.23
875.34
1011.98
1139.80
1468.40
1579.48
1781.35
1892.20

Figure S5-16

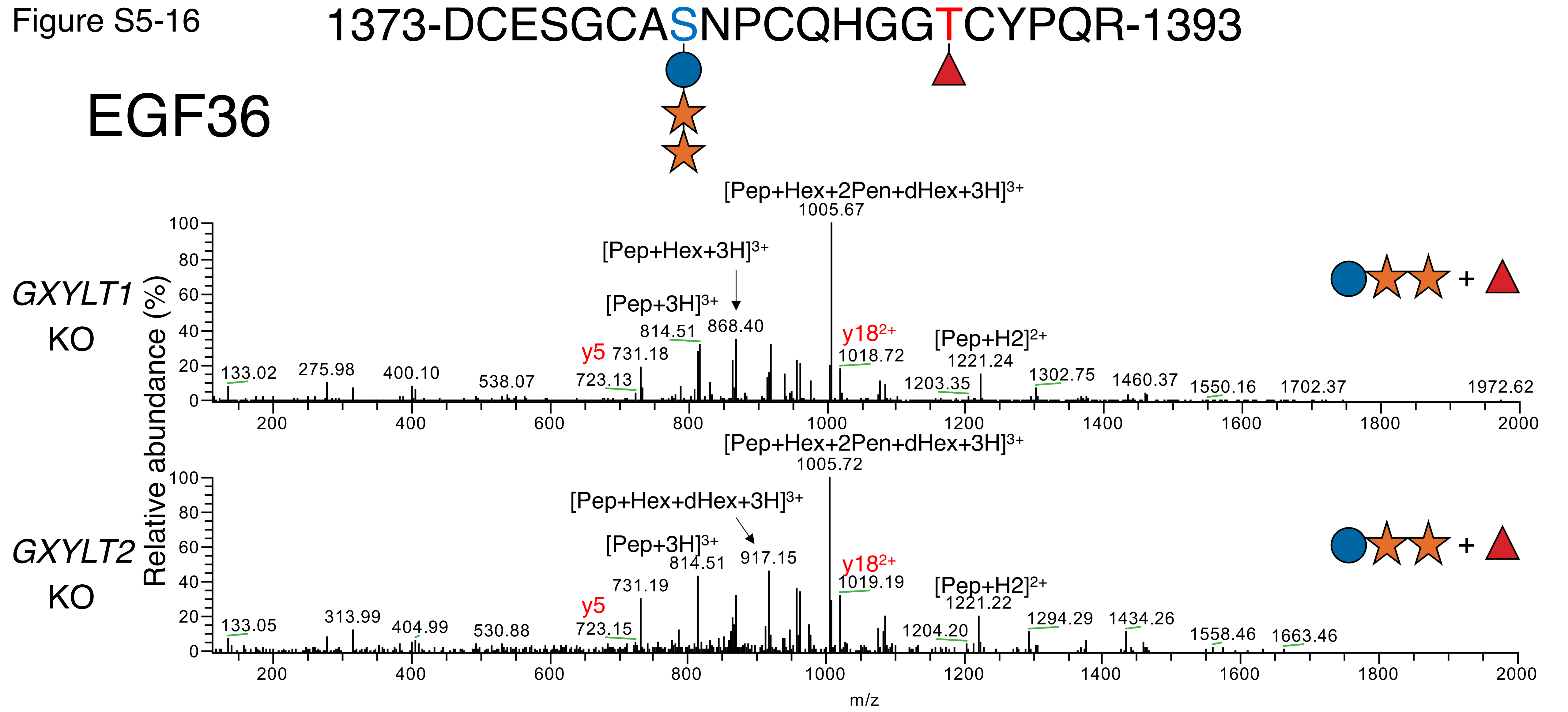

1373-DCESGCASNPCQHGGTCYPQR-1393
EGF36
GXYLT1 KO
GXYLT2 KO
Relative abundance (%)
m/z
[Pep+Hex+2Pen+dHex+3H]3+
1005.67
[Pep+Hex+3H]3+
[Pep+3H]3+
814.51
868.40
y5
731.18
723.13
y18 2+
1018.72
[Pep+H2]2+
1221.24
1203.35
1302.75
1460.37
1550.16
1702.37
1972.62
133.02
275.98
400.10
538.07
[Pep+Hex+2Pen+dHex+3H]3+
1005.72
[Pep+Hex+dHex+3H]3+
917.15
[Pep+3H]3+
814.51
731.19
y5
723.15
y18 2+
1019.19
[Pep+H2]2+
1221.22
1204.20
1294.29
1434.26
1558.46
1663.46
133.05
313.99
404.99
530.88

Figure S5-17

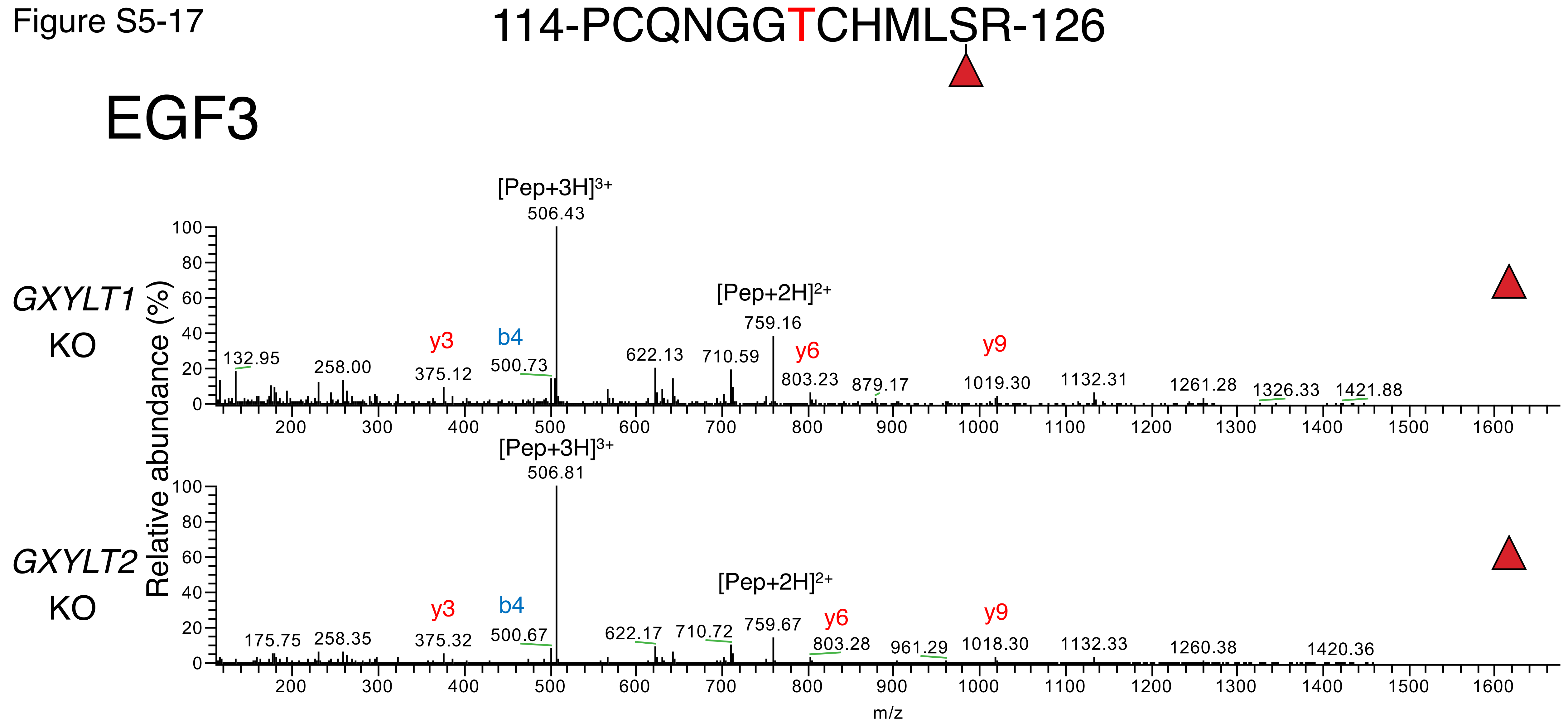
114-PCQNGGTCHMLSR-126
EGF3
GXYLT1 KO
GXYLT2 KO
Relative abundance (%)
[Pep+3H]3+
506.43
[Pep+2H]2+
759.16
y3
b4
y6
y9
132.95
258.00
375.12
500.73
622.13
710.59
803.23
879.17
1019.30
1132.31
1261.28
1326.33
1421.88
[Pep+3H]3+
506.81
[Pep+2H]2+
175.75
258.35
375.32
500.67
622.17
710.72
759.67
803.28
961.29
1018.30
1132.33
1260.38
1420.36
m/z

Figure S5-18

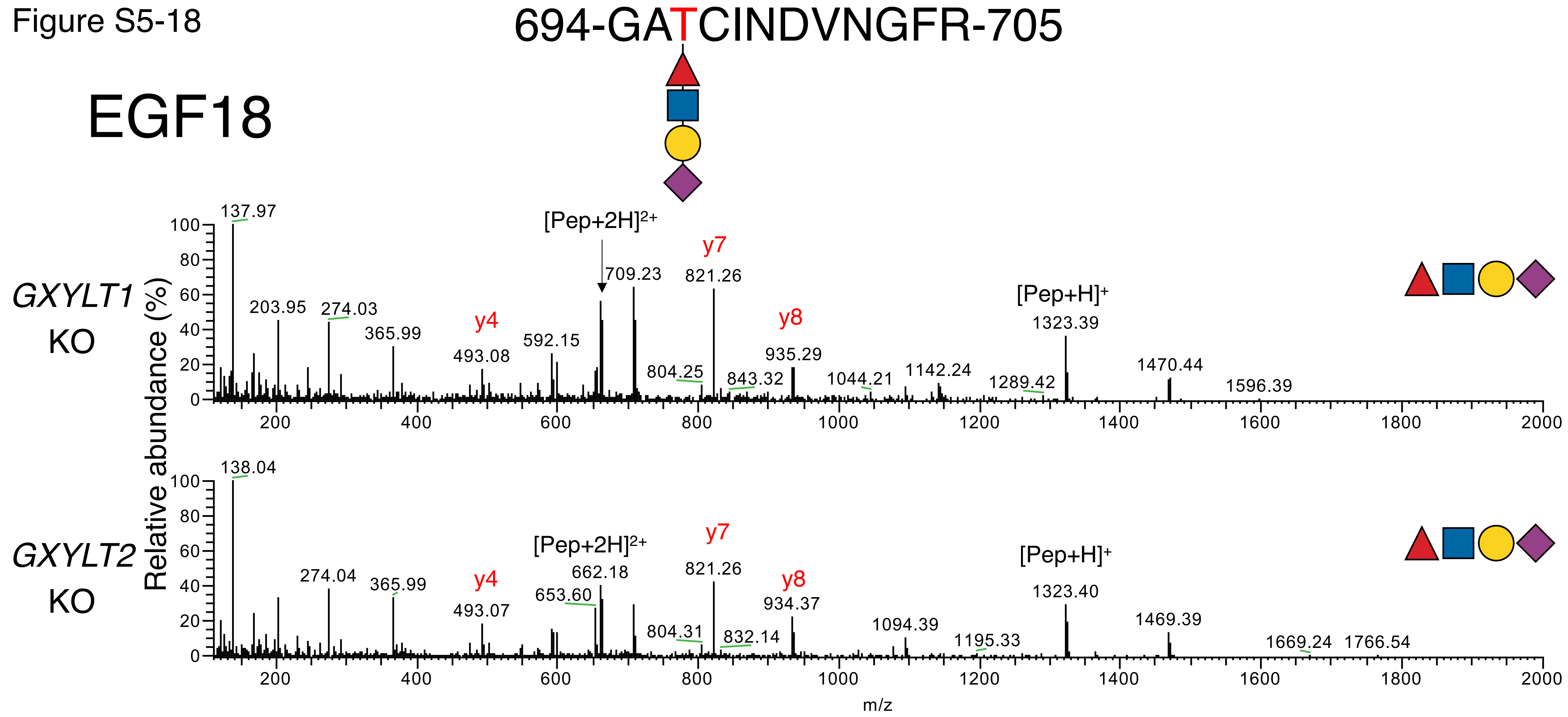
694-GATCINDVNGFR-705
EGF18
GXYLT1
KO
GXYLT2
KO
Relative abundance (%)
m/z
[Pep+2H]2+
[Pep+H]+
y4
y7
y8
137.97
203.95
274.03
365.99
493.08
592.15
709.23
821.26
804.25
843.32
935.29
1044.21
1142.24
1289.42
1323.39
1470.44
1596.39
138.04
274.04
365.99
493.07
653.60
662.18
821.26
804.31
832.14
934.37
1094.39
1195.33
1323.40
1469.39
1669.24
1766.54

Figure S5-19

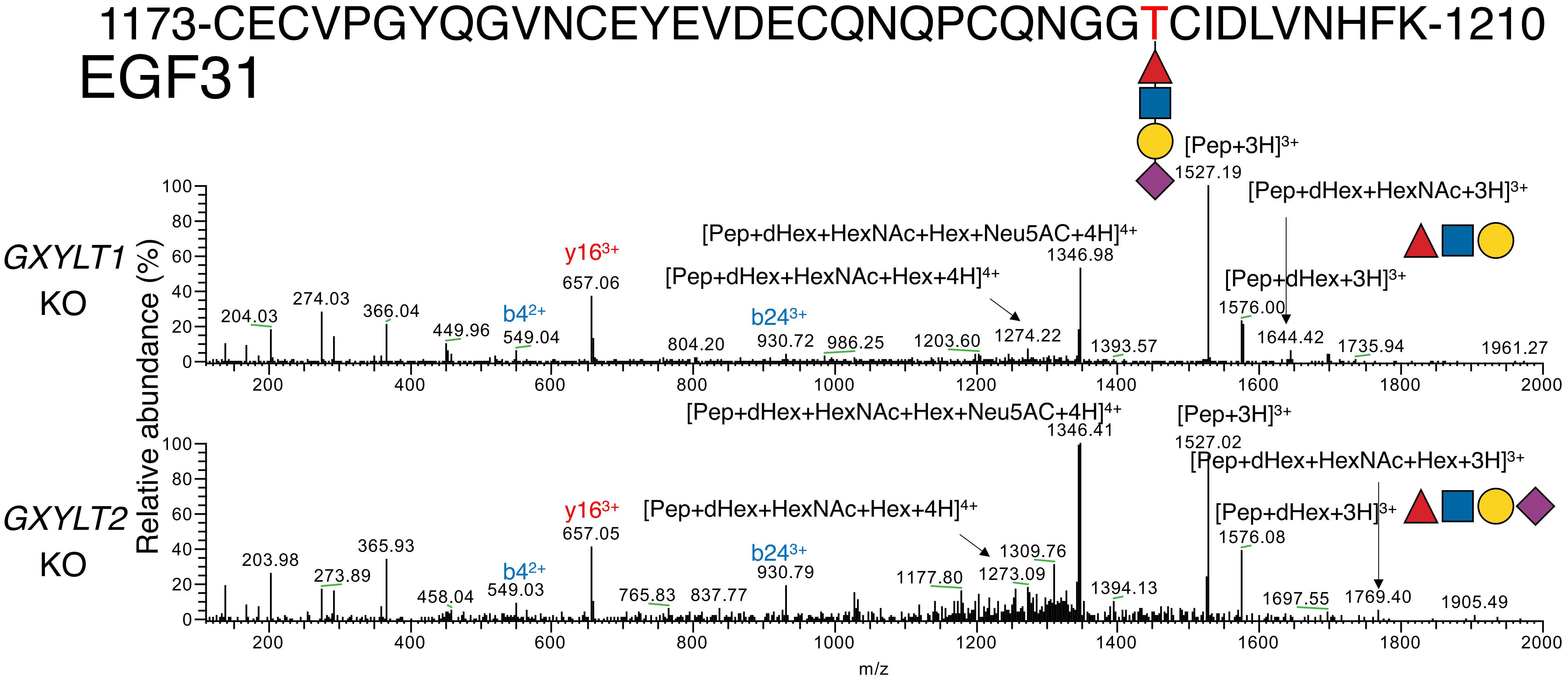

1173-CECVPGYQGVNCEYEVDECQNQPCQNGGTCIDLVNHFK-1210
EGF31
GXYLT1 KO
GXYLT2 KO
Relative abundance (%)
m/z
[Pep+3H]3+
1527.19
[Pep+dHex+HexNAc+3H]3+
[Pep+dHex+HexNAc+Hex+Neu5AC+4H]4+
1346.98
[Pep+dHex+HexNAc+Hex+4H]4+
1274.22
[Pep+dHex+3H]3+
1576.00
1644.42
y16 3+
657.06
b4 2+
549.04
b24 3+
930.72
204.03
274.03
366.04
449.96
804.20
986.25
1203.60
1393.57
1735.94
1961.27
[Pep+dHex+HexNAc+Hex+Neu5AC+4H]4+
1346.41
[Pep+3H]3+
1527.02
[Pep+dHex+HexNAc+Hex+3H]3+
[Pep+dHex+3H]3+
1576.08
1769.40
[Pep+dHex+HexNAc+Hex+4H]4+
1309.76
y16 3+
657.05
b4 2+
549.03
b24 3+
930.79
203.98
273.89
365.93
458.04
765.83
837.77
1177.80
1273.09
1394.13
1697.55
1905.49

Figure S5-20

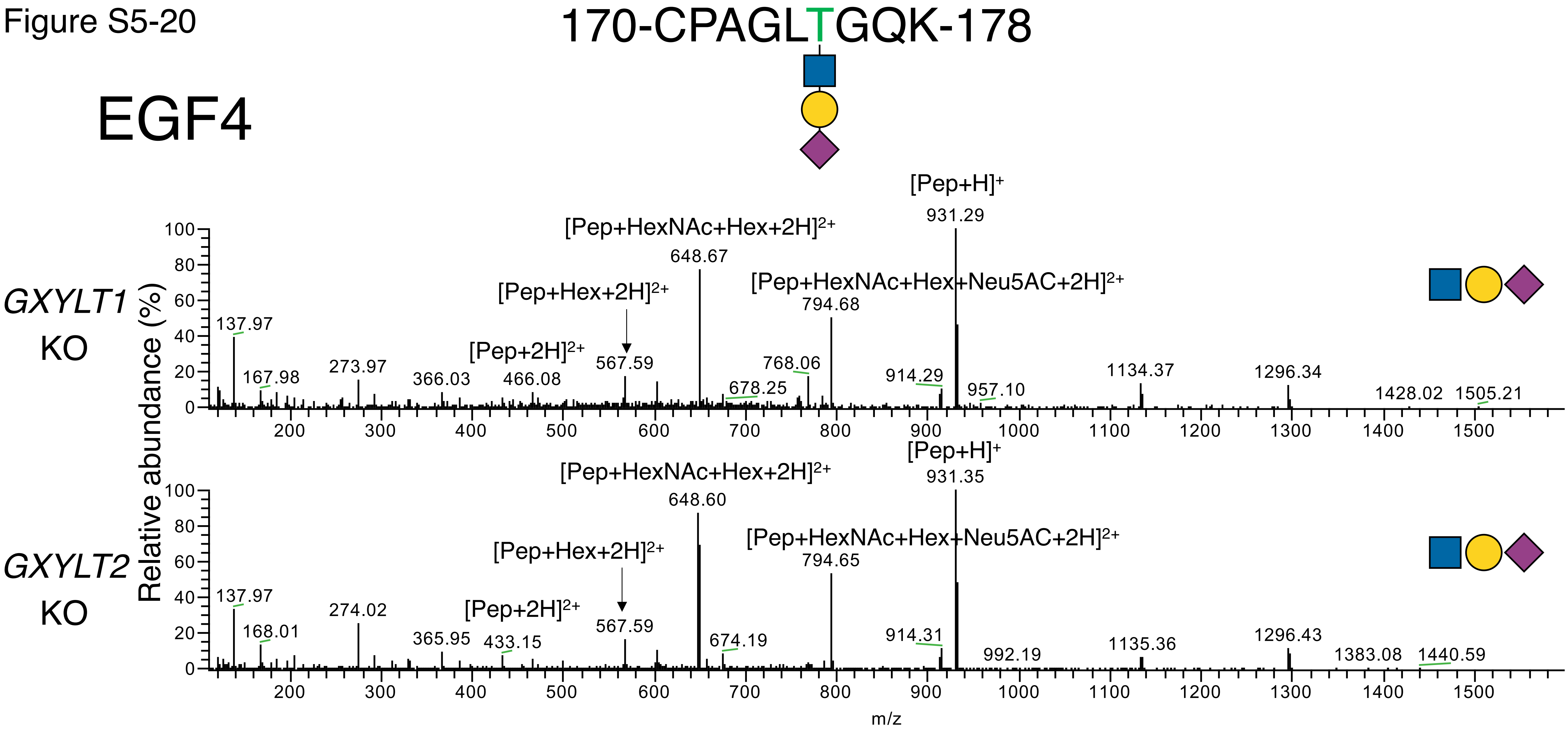
170-CPAGLTGQK-178
EGF4
GXYLT1
KO
GXYLT2
KO
Relative abundance (%)
m/z
[Pep+H]+
931.29
[Pep+HexNAc+Hex+2H]2+
648.67
[Pep+HexNAc+Hex+Neu5AC+2H]2+
794.68
[Pep+Hex+2H]2+
567.59
[Pep+2H]2+
466.08
137.97
167.98
273.97
366.03
678.25
768.06
914.29
957.10
1134.37
1296.34
1428.02
1505.21
[Pep+H]+
931.35
[Pep+HexNAc+Hex+2H]2+
648.60
[Pep+HexNAc+Hex+Neu5AC+2H]2+
794.65
[Pep+Hex+2H]2+
567.59
[Pep+2H]2+
433.15
137.97
168.01
274.02
365.95
674.19
914.31
992.19
1135.36
1296.43
1383.08
1440.59
0
20
40
60
80
100
200
300
400
500
600
700
800
900
1000
1100
1200
1300
1400
1500

Figure S5-21

666-YSCVCSPGFTGQR-678

EGF17

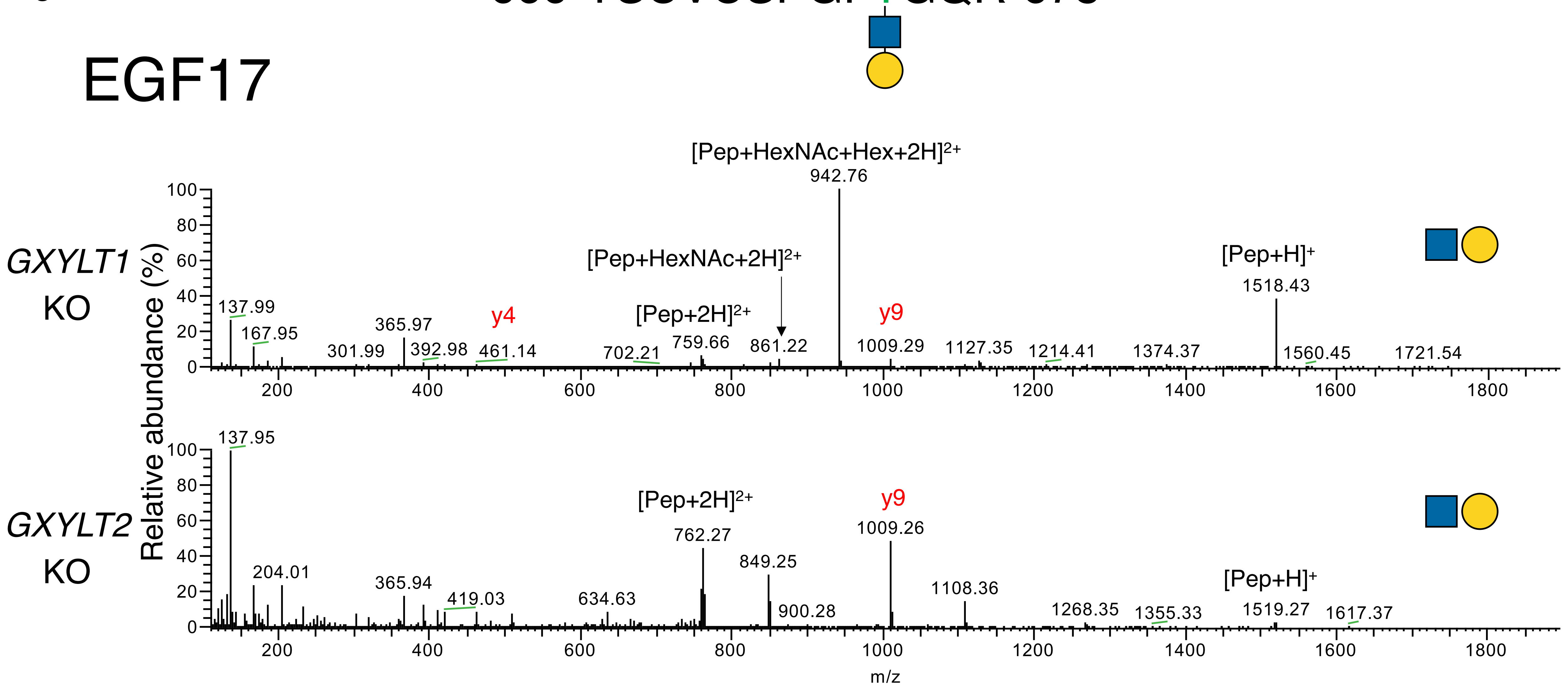

Figure S5-22

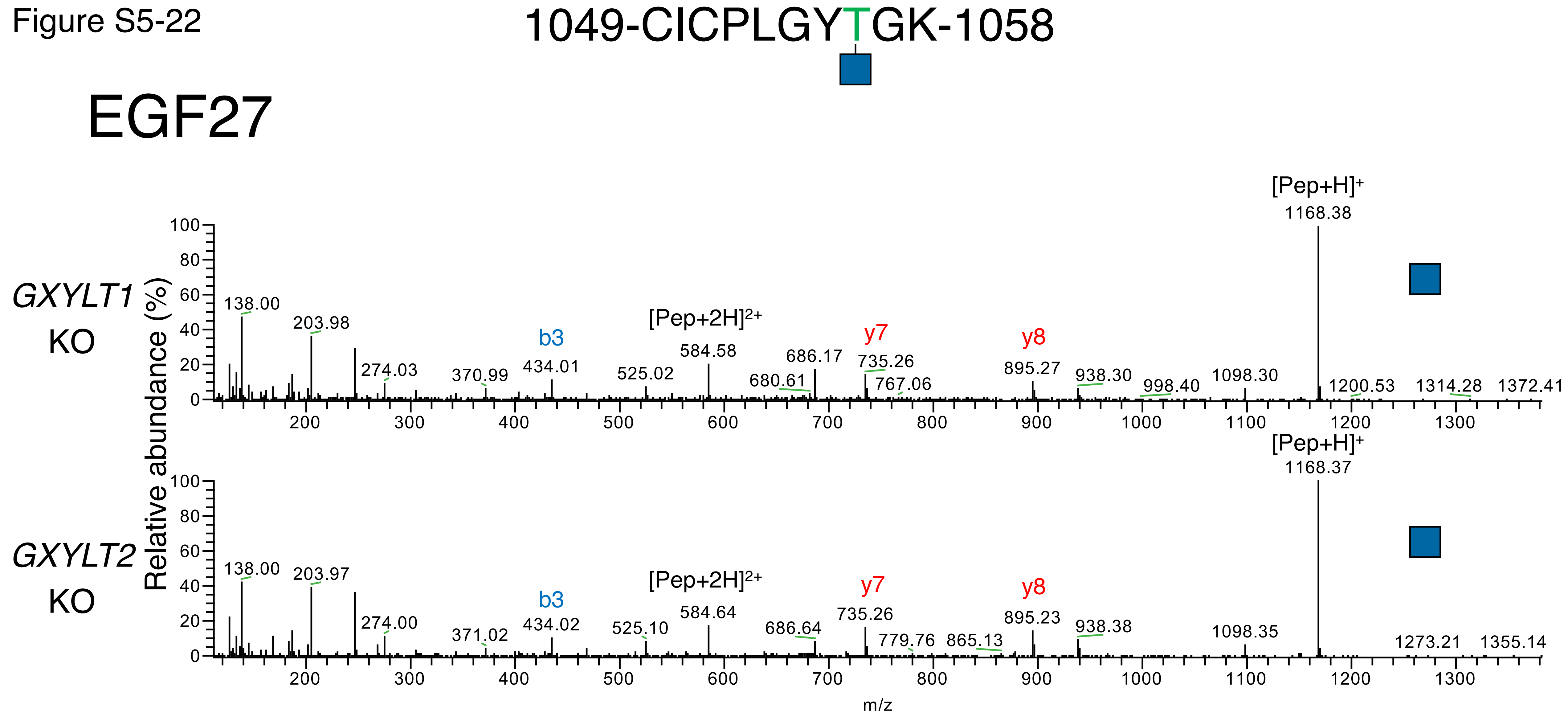
1049-CICPLGYTGK-1058
EGF27
GXYLT1 KO
GXYLT2 KO
Relative abundance (%)
[Pep+H]+
1168.38
138.00
203.98
274.03
370.99
b3
434.01
525.02
[Pep+2H]2+
584.58
680.61
686.17
y7
735.26
767.06
y8
895.27
938.30
998.40
1098.30
1200.53
1314.28
1372.41
[Pep+H]+
1168.37
138.00
203.97
274.00
371.02
b3
434.02
525.10
[Pep+2H]2+
584.64
686.64
y7
735.26
779.76
865.13
y8
895.23
938.38
1098.35
1273.21
1355.14
m/z

Figure S5. MS/MS spectra of (glyco)peptides from NOTCH2.

Samples were generated in *GXYLT1* KO HEK293T cells (*top*) and *GXYLT2* KO cells (*bottom*), transfected with a plasmid encoding mouse NOTCH2 EGF1-36 as described in the Experimental procedure section. The MS/MS spectra used for the analysis of *O*-Glc glycans are shown in Figures S5-1 to 16. MS/MS spectra confirmed the identity of (glyco)peptides based on the presence of peptide-specific b- and y-ions and neutral loss of predicted glycans. Amino acid sequences of the (glyco)peptides are shown at the top of the figure. MS/MS spectra of the (glyco)peptides modified with the most elongated glycoform derived from each cell clone are shown. The sequence of peptides, the predicted and measured mass-to-charge ratios, and the charge states are summarized in Table S11. Black bar, naked peptide; red triangle, Fuc; blue square, GlcNAc; yellow circle, galactose; purple diamond, Neu5Ac.

Figure S6

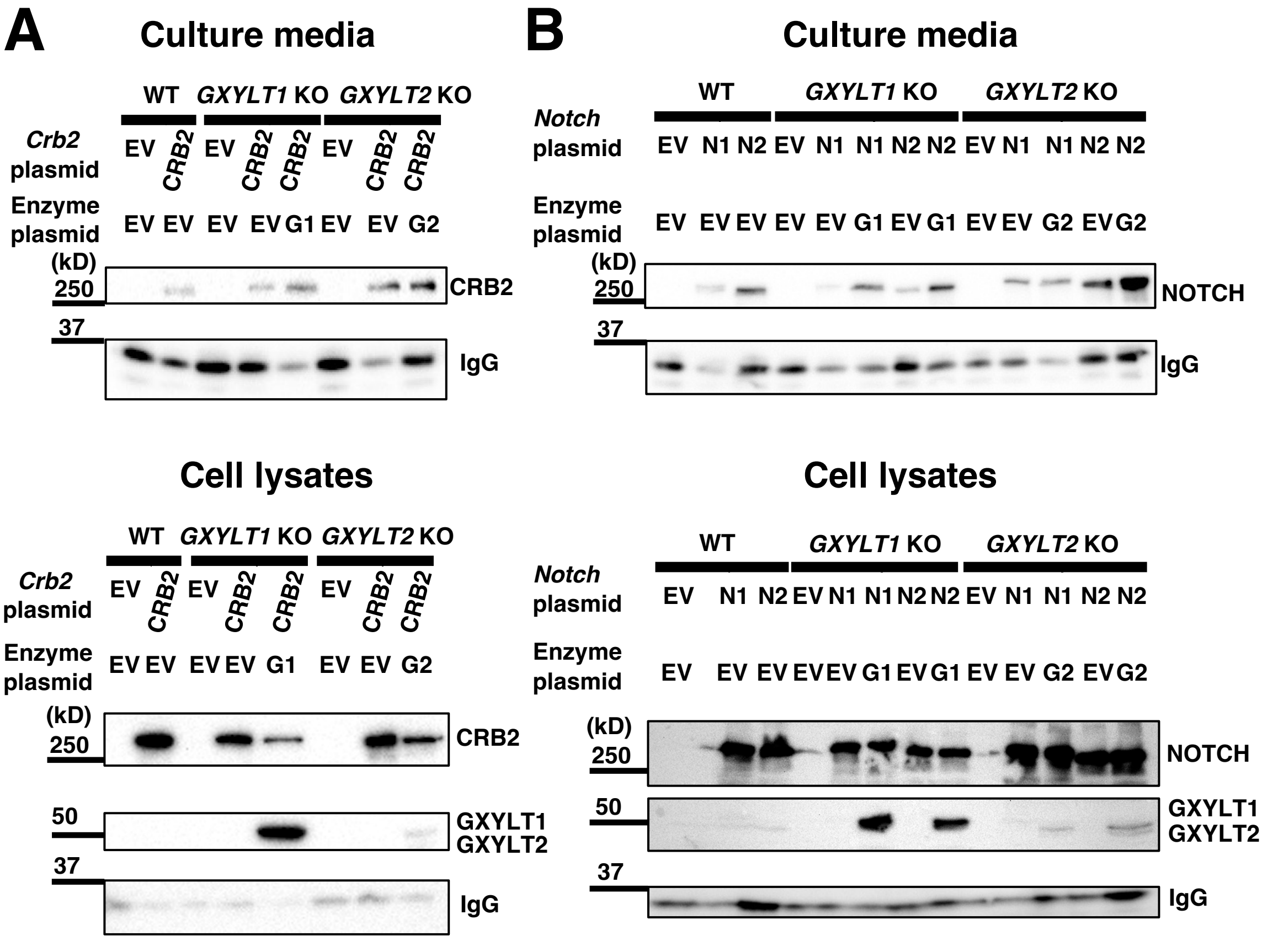
A
Culture media
WT
GXYLT1 KO
GXYLT2 KO
Crb2 plasmid
EV CRB2 EV CRB2 CRB2 EV CRB2 CRB2
Enzyme plasmid
EV EV EV EV G1 EV EV G2
(kD)
250
CRB2
37
IgG
Cell lysates
WT
GXYLT1 KO
GXYLT2 KO
Crb2 plasmid
EV CRB2 EV CRB2 CRB2 EV CRB2 CRB2
Enzyme plasmid
EV EV EV EV G1 EV EV G2
(kD)
250
CRB2
50
GXYLT1
GXYLT2
37
IgG
B
Culture media
WT
GXYLT1 KO
GXYLT2 KO
Notch plasmid
EV N1 N2 EV N1 N1 N2 N2 EV N1 N1 N2 N2
Enzyme plasmid
EV EV EV EV EV G1 EV G1 EV EV G2 EV G2
(kD)
250
NOTCH
37
IgG
Cell lysates
WT
GXYLT1 KO
GXYLT2 KO
Notch plasmid
EV N1 N2 EV N1 N1 N2 N2 EV N1 N1 N2 N2
Enzyme plasmid
EV EV EV EV EV G1 EV G1 EV EV G2 EV G2
(kD)
250
NOTCH
50
GXYLT1
GXYLT2
37
IgG

Figure S6. GXYLT1 contributes to the trafficking of CRB2, NOTCH1, and NOTCH2 more than GXYLT2 in HEK293T cells.

(A) Western blot analysis of the Myc/His-tagged extracellular domain EGF1-15 of CRB2 transfected in wild-type (WT), *GXYLT1* KO, or *GXYLT2* KO HEK293T cells. An expression plasmid of IgG was co-transfected as a transfection control. Rescue experiments with co-transfection of *GXYLT1* (G1) or *GXYLT2* (G2) were also performed. Two independent experiments were performed. Representative data are shown. (B) Western blot analysis of the Myc/His-tagged extracellular domain EGF1-36 of NOTCH1 (N1) and NOTCH2 (N2) transfected in wild-type (WT), *GXYLT1* KO, or *GXYLT2* KO HEK293T cells. An expression plasmid of IgG was co-transfected as a transfection control. Rescue experiments with co-transfection of *GXYLT1* (G1) or *GXYLT2* (G2) were also performed. Two independent experiments were performed. Representative data are shown.

Figure S7-1

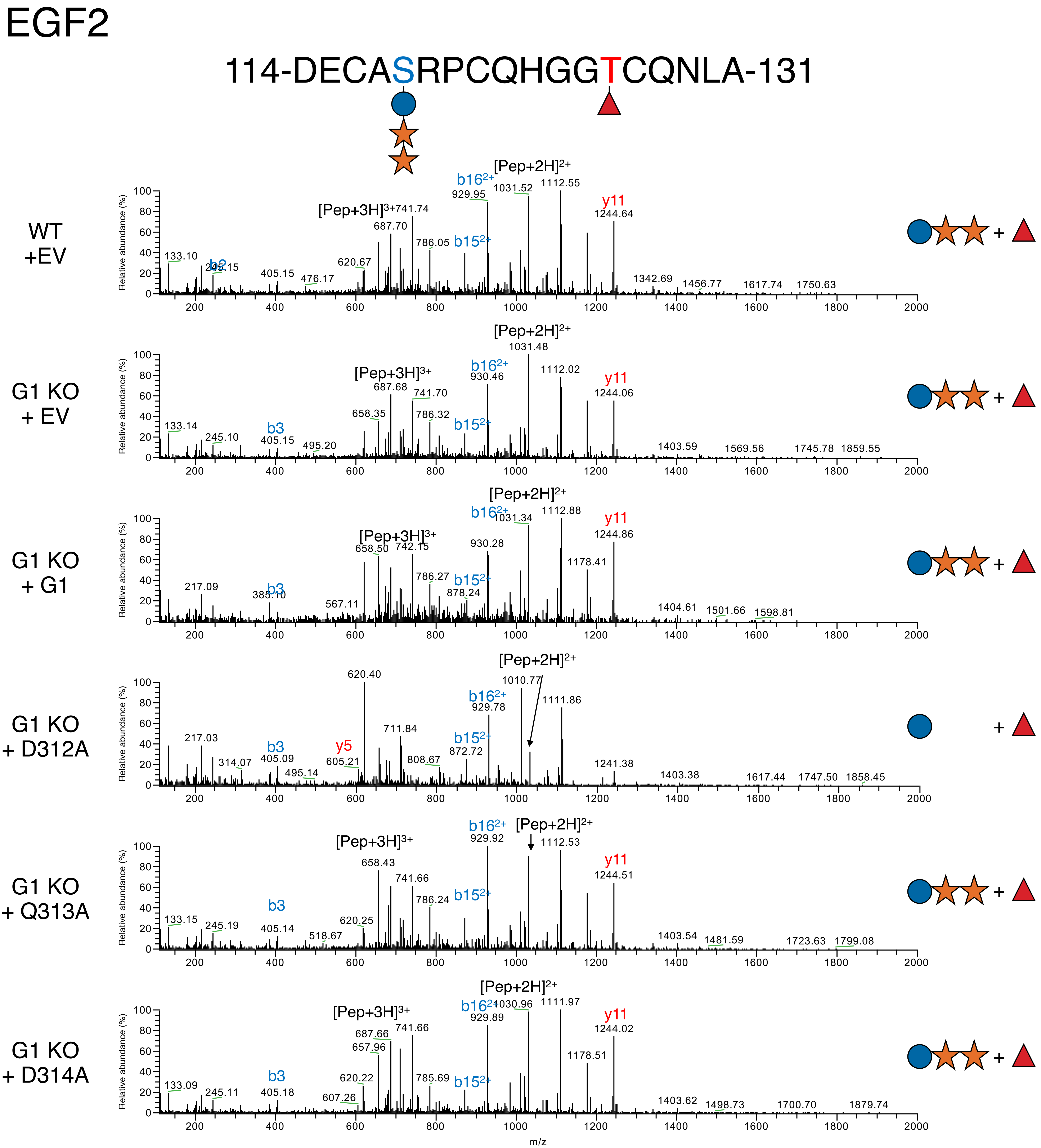

EGF2
114-DECASRPCQHGGTCQNLA-131
WT
+EV
G1 KO
+ EV
G1 KO
+ G1
G1 KO
+ D312A
G1 KO
+ Q313A
G1 KO
+ D314A
[Pep+2H]2+
[Pep+3H]3+
b16 2+
b15 2+
y11
b3
y5
Relative abundance (%)
m/z

Figure S7-2

EGF3

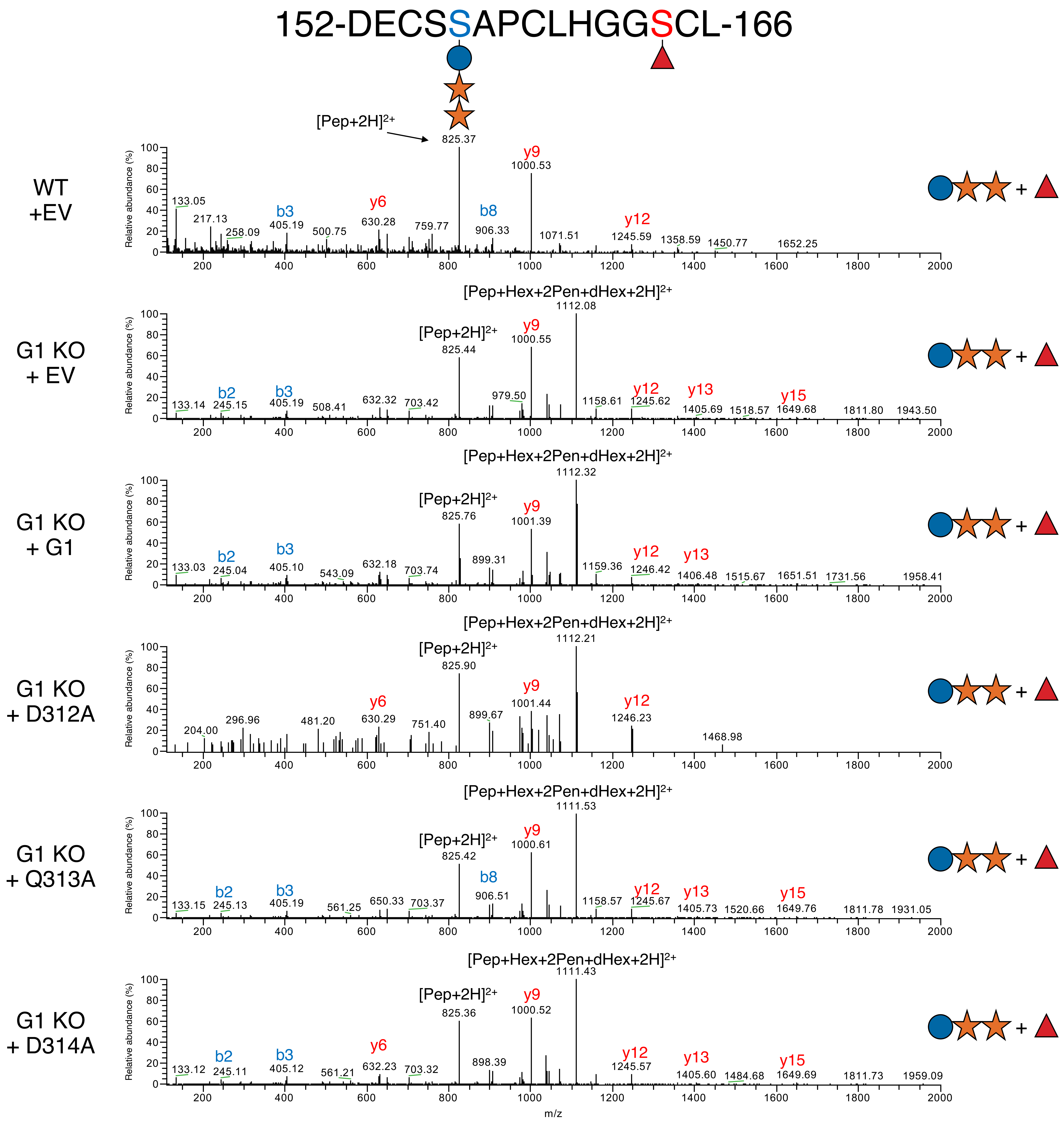

Figure S7-3

EGF4

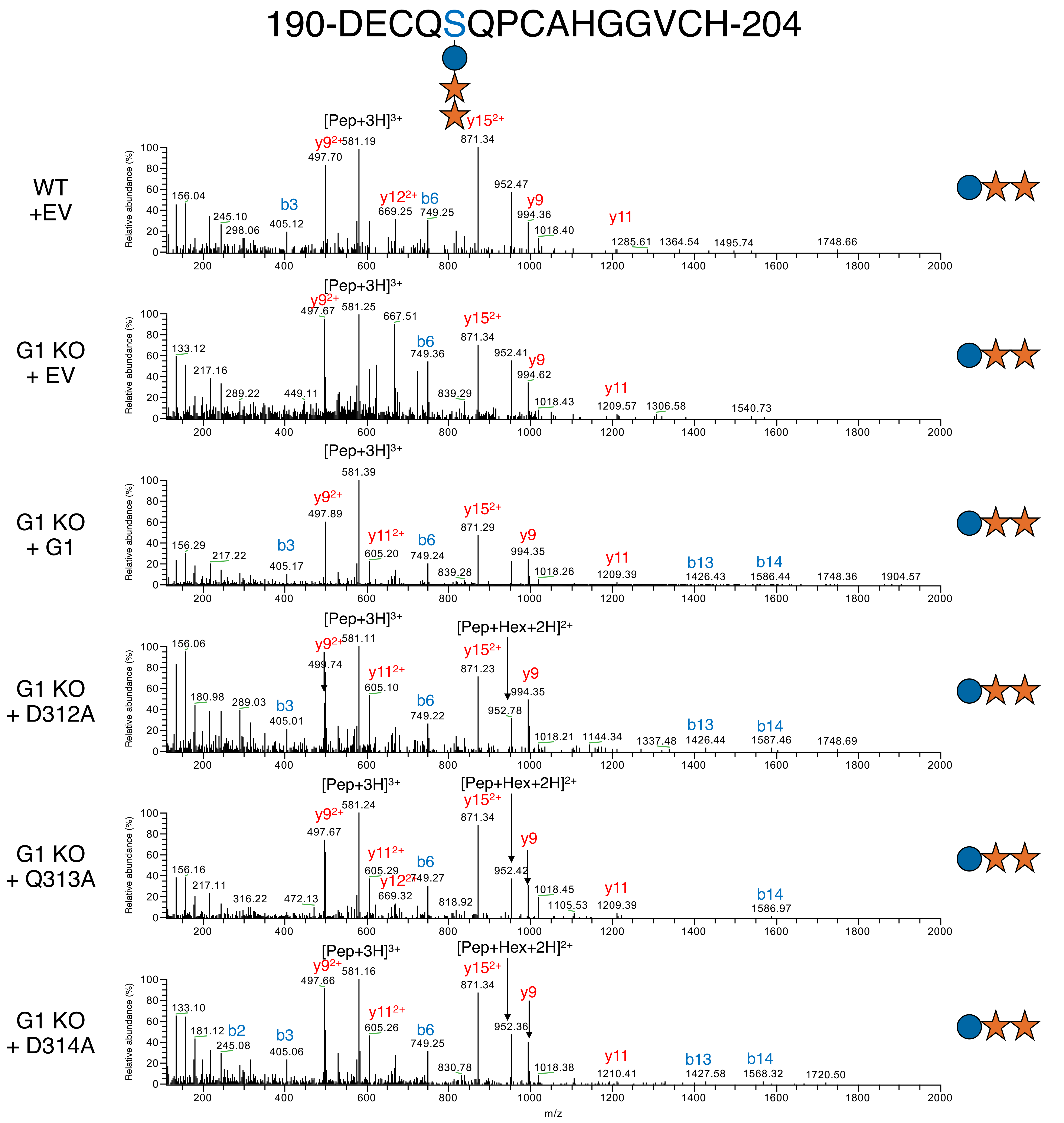

Figure S7-4

EGF5

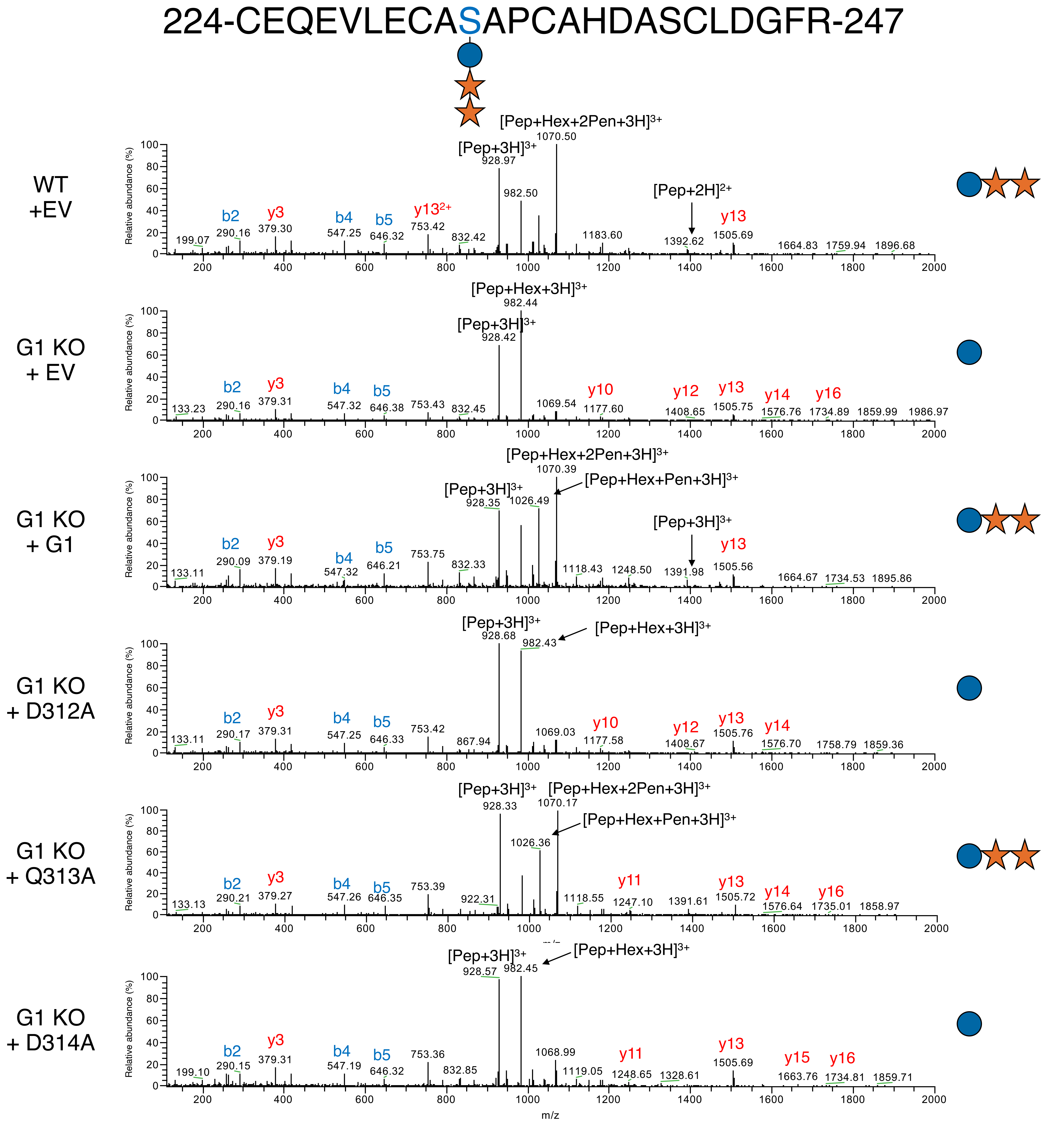

Figure S7-5

EGF6

262-CEVDEDECASGPCQNGGQCLQR-283

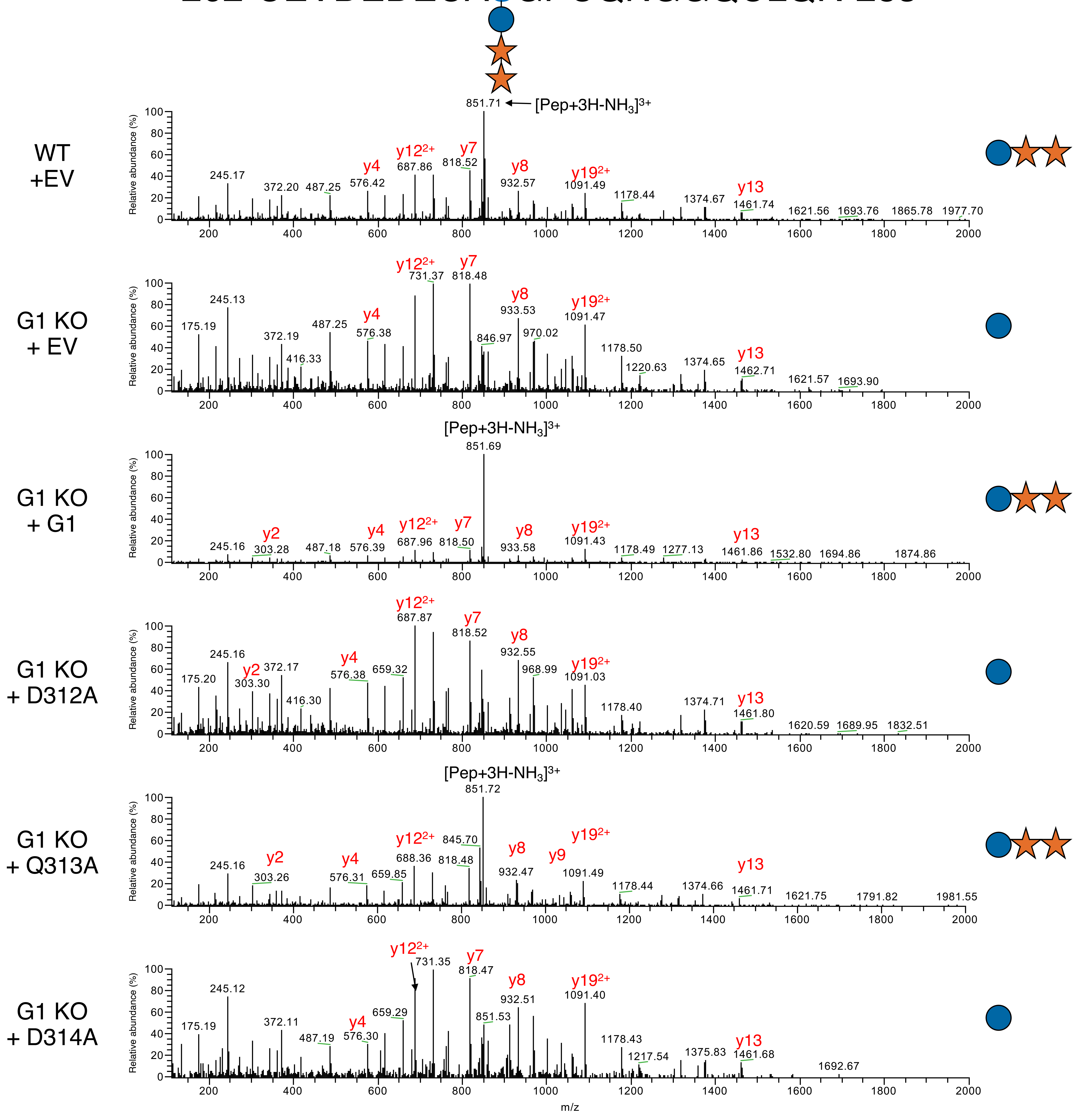

Figure S7-6

EGF7

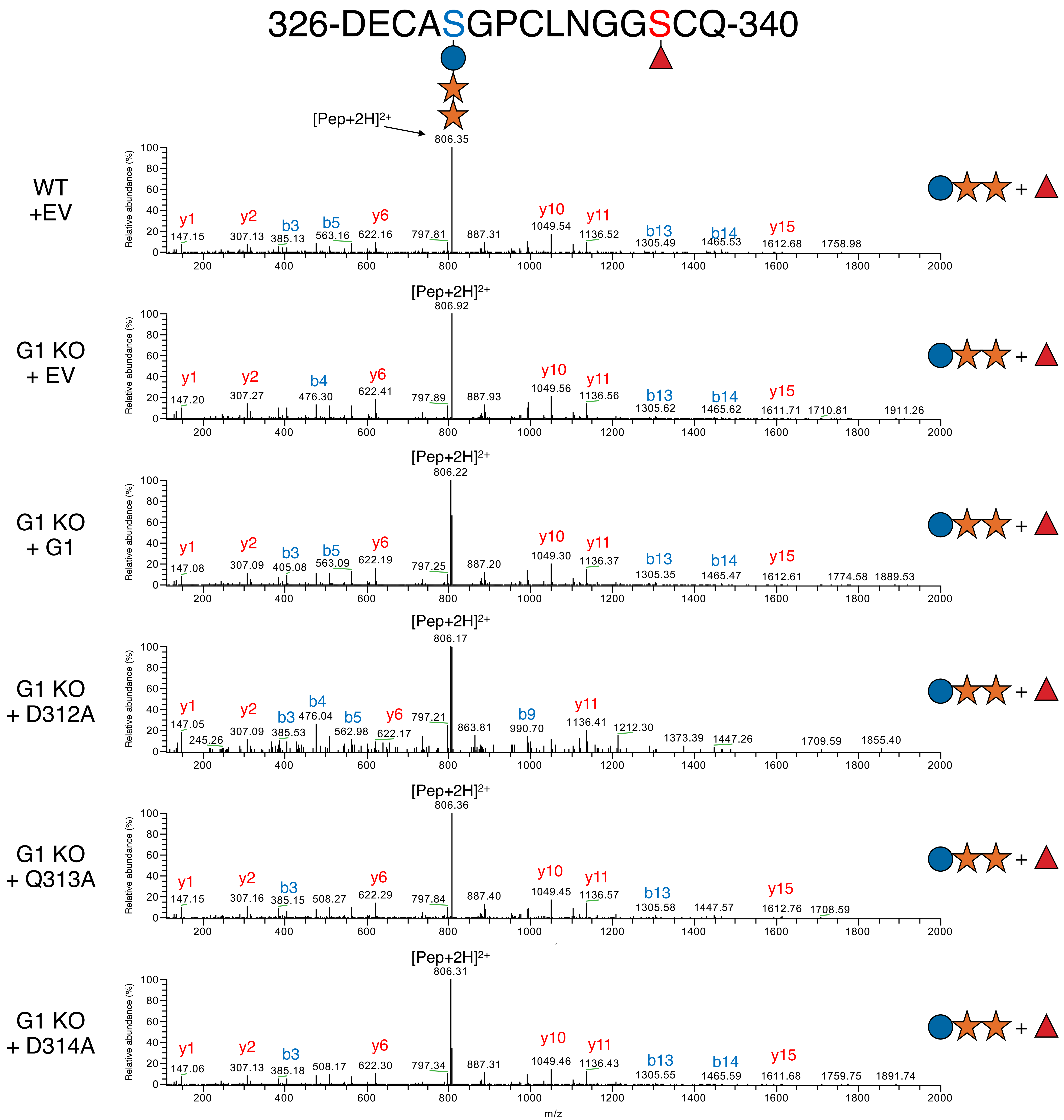

Figure S7-7

EGF8

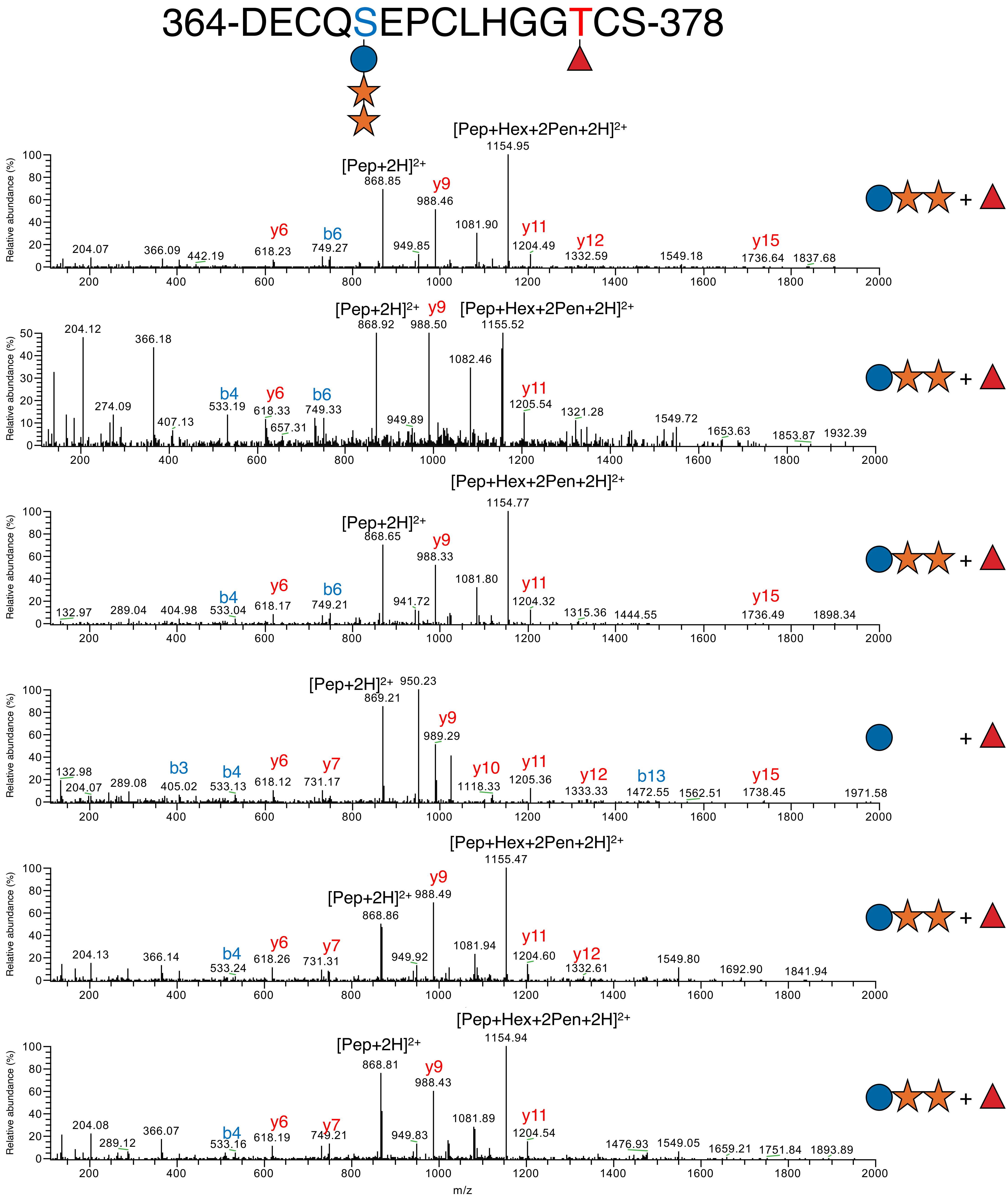

Figure S7. MS/MS spectra of (glyco)peptides from CRB2.

Samples were generated in wild-type (WT) control HEK293T cells transfected with both a plasmid encoding mouse CRB2 EGF1-8 and an empty vector (EV), or in *GXYLT1* (G1) KO cells transfected with both a plasmid encoding mouse CRB2 EGF1-8 and a plasmid encoding wild-type G1, or each of three DQD mutants (D312A, Q313A, or D314A) as indicated. MS/MS spectra confirmed the identity of (glyco)peptides based on the presence of peptide-specific b- and y-ions and the neutral loss of predicted glycans. The amino acid sequences of the (glyco)peptides are shown at the top of the figure. MS/MS spectra of the (glyco)peptides modified with the most elongated glycoform derived from each cell clone are shown. The sequence of peptides, the predicted and measured mass-to-charge ratios, and the charge states are summarized in Table S12.

Figure S8-1

EGF2

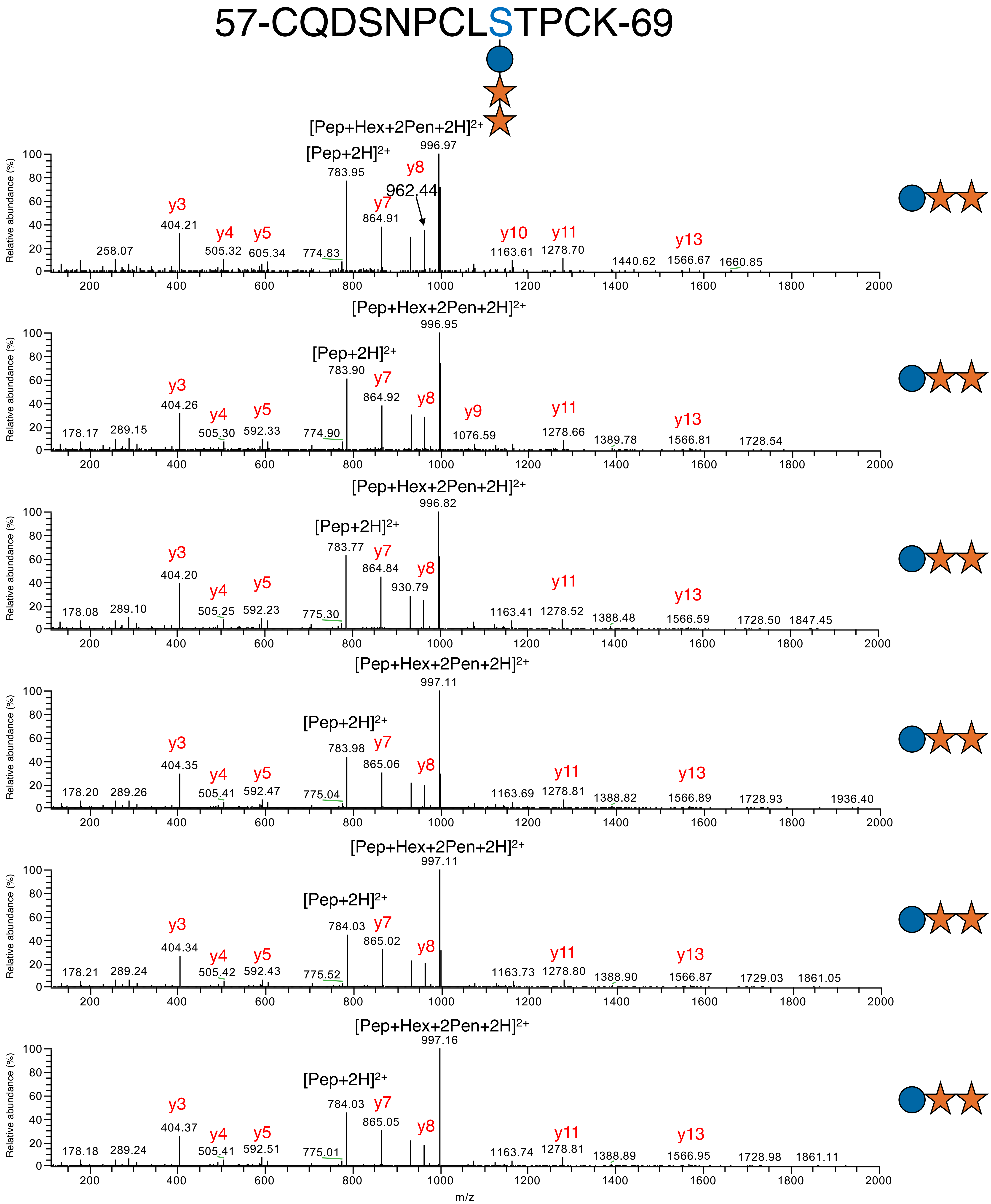

Figure S8-2

EGF4

137-SCQQADPCASNPCANGGQCLPFESSYICR-165

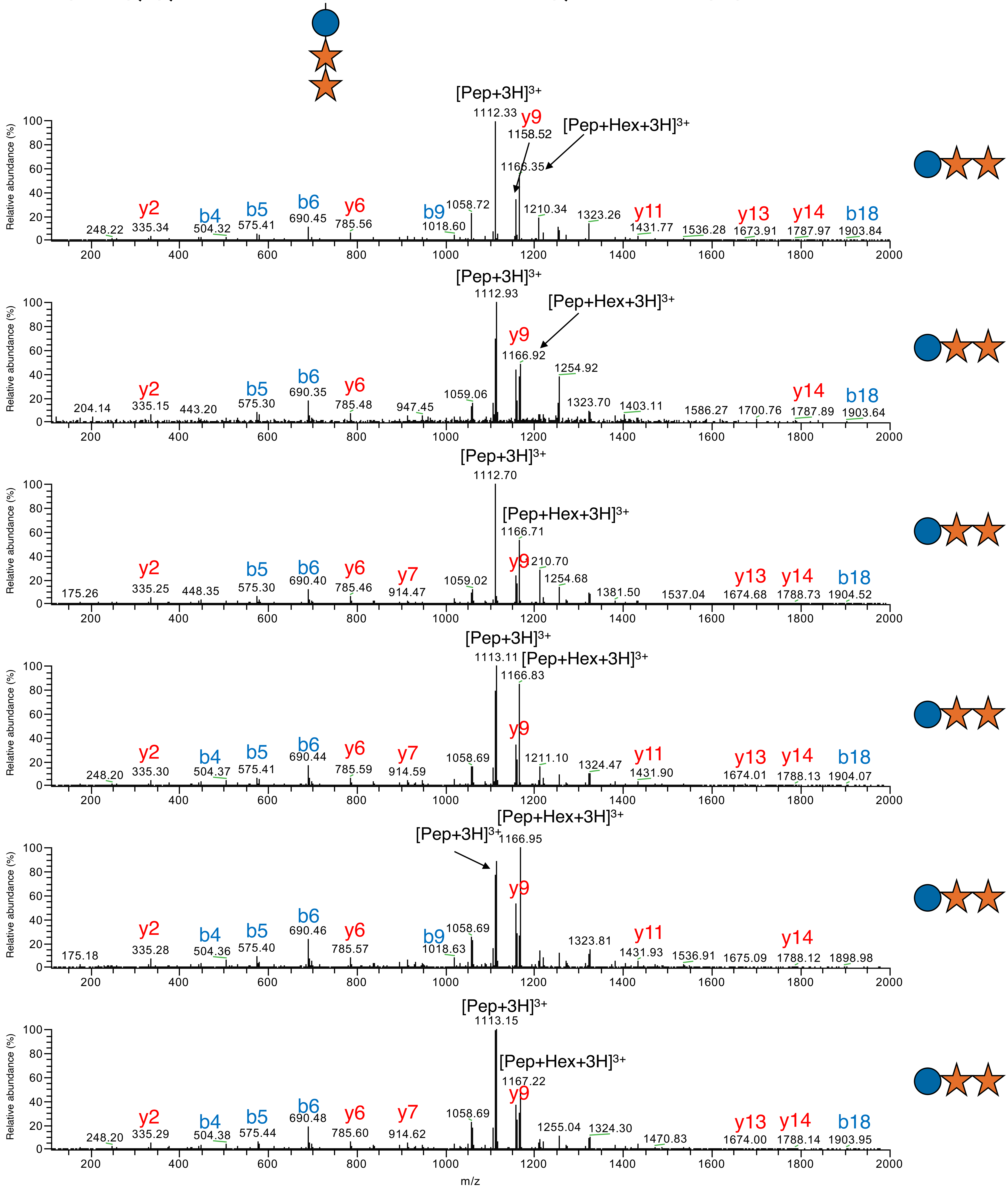

Figure S8-3

EGF10

366-TGLLCHLNDACISNPCNEGSNCDTNPVNGK-395

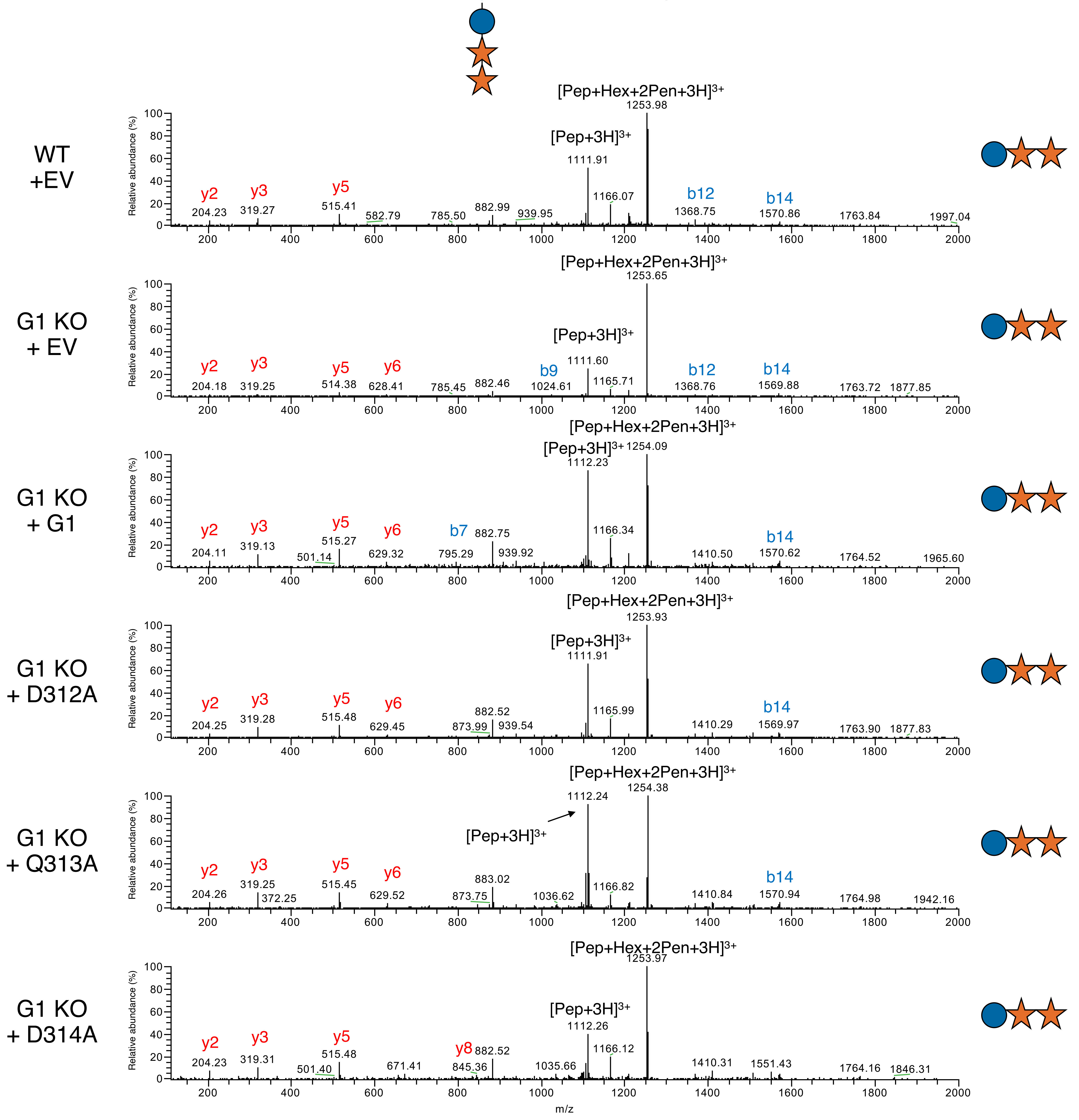

Figure S8. MS/MS spectra of (glyco)peptides from NOTCH1.

Samples were generated in wild-type (WT) control HEK293T cells transfected with both a plasmid encoding mouse NOTCH1 EGF1-12 and an empty vector (EV), or in *GXYLT1* (G1) KO cells transfected with both a plasmid encoding mouse NOTCH1 EGF1-12 and a plasmid encoding wild-type G1, or each of three DQD mutants (D312A, Q313A, or D314A) as indicated. MS/MS spectra confirmed the identity of (glyco)peptides based on the presence of peptide-specific b- and y-ions and the neutral loss of predicted glycans. The amino acid sequences of the (glyco)peptides are shown at the top of the figure. MS/MS spectra of the (glyco)peptides modified with the most elongated glycoform derived from each cell clone are shown. The sequence of peptides, the predicted and measured mass-to-charge ratios, and the charge states are summarized in Table S13.

Figure S9-1

EGF4

141-QCQWTDACLSHPCEDGSTCTSVASQFSCK-169

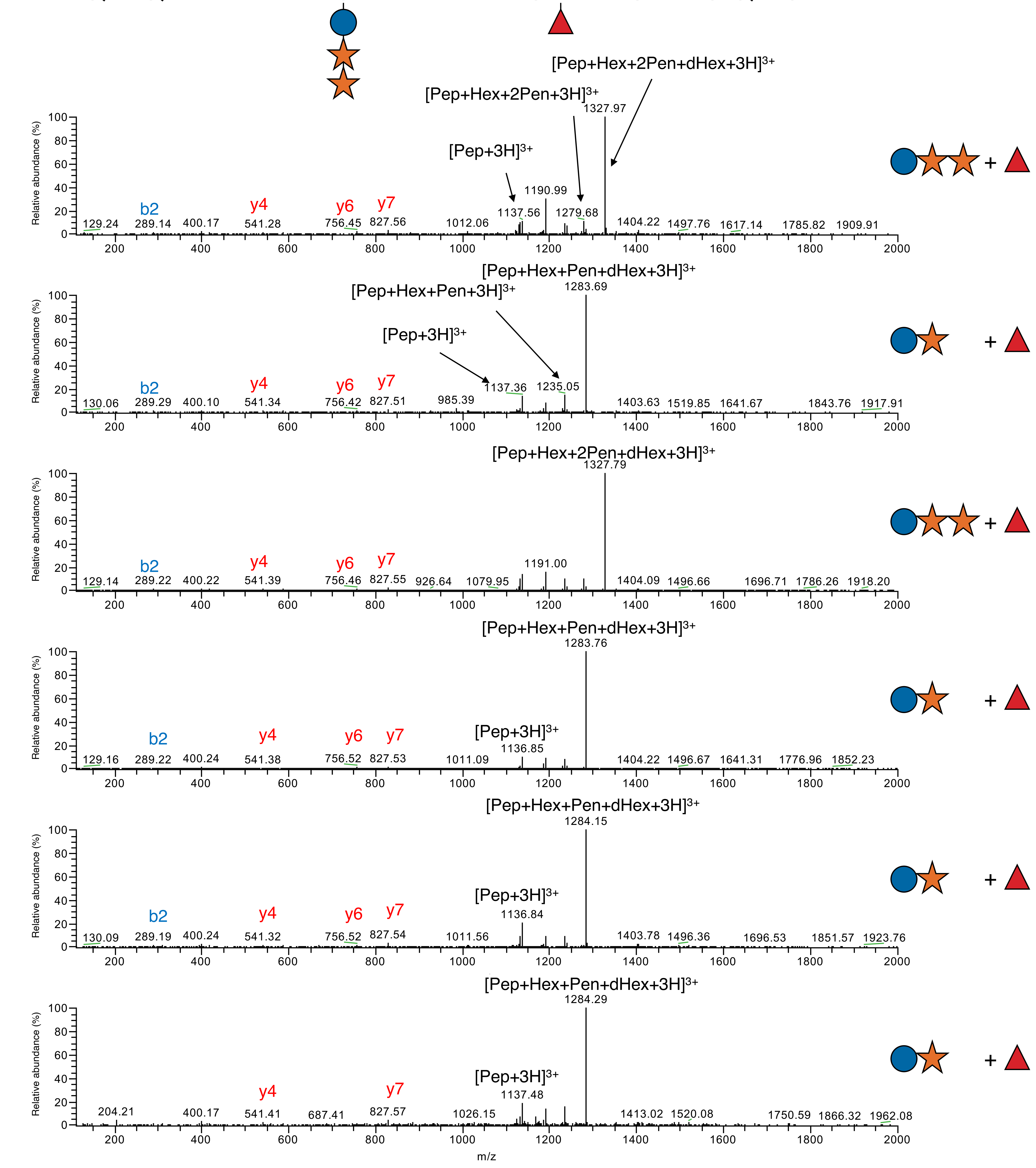

Figure S9-2

EGF10

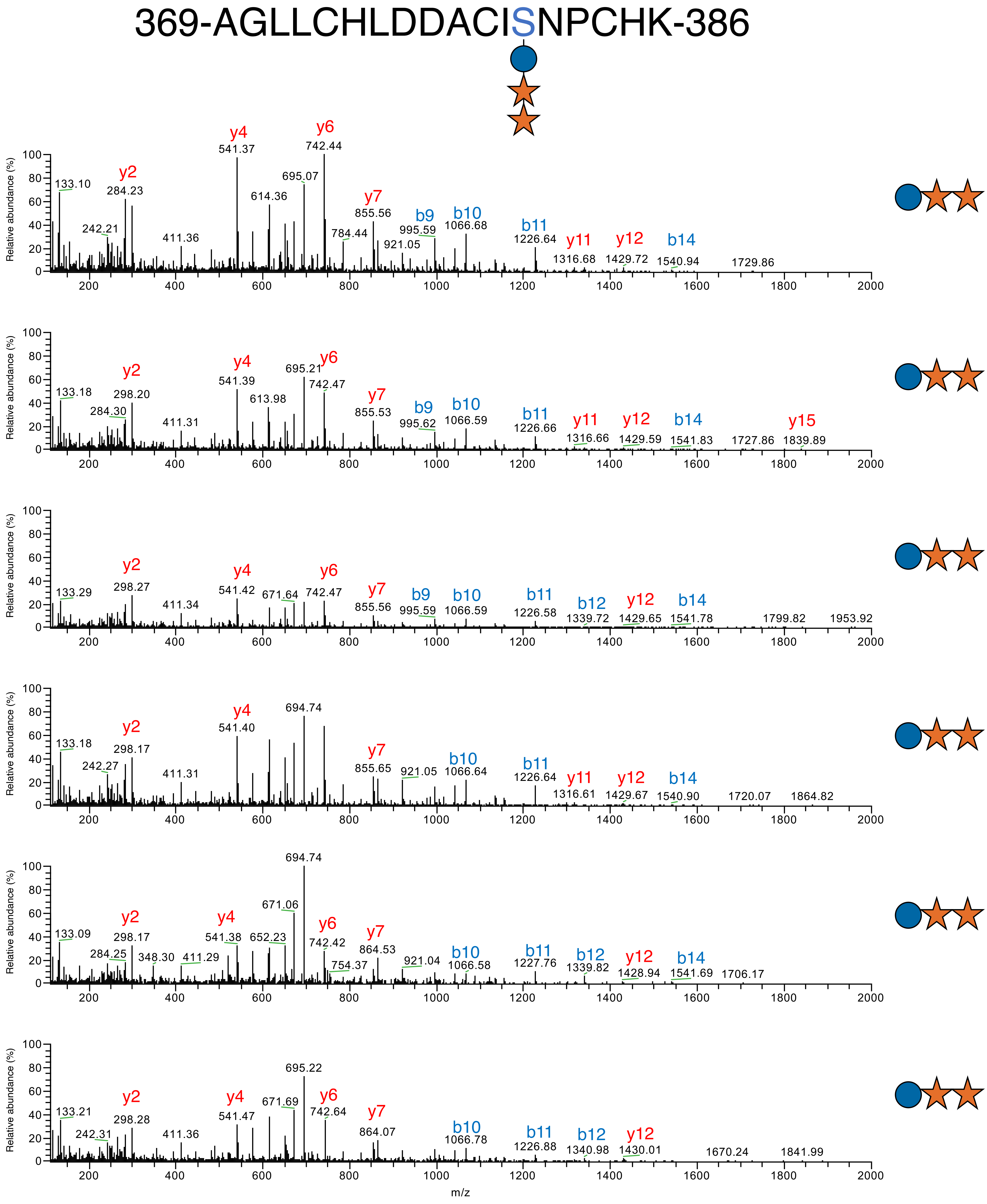

Figure S9-3

EGF12

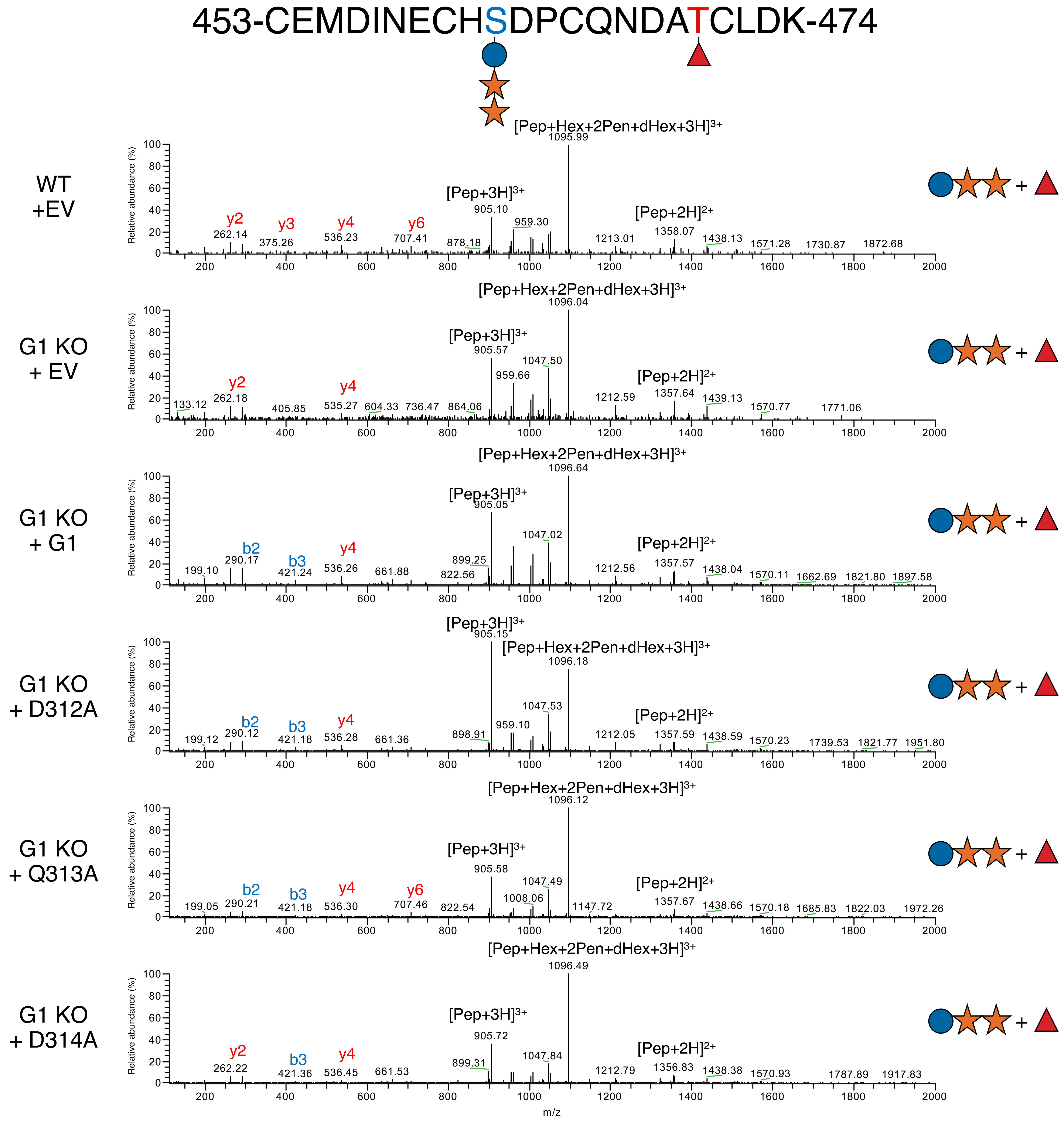

Figure S9. MS/MS spectra of (glyco)peptides from NOTCH2.

Samples were generated in wild-type (WT) control HEK293T cells transfected with both a plasmid encoding mouse NOTCH2 EGF1-12 and an empty vector (EV), or in *GXYLT1* (G1) KO cells transfected with both a plasmid encoding mouse NOTCH2 EGF1-12 and a plasmid encoding wild-type G1, or each of three DQD mutants (D312A, Q313A, or D314A) as indicated. MS/MS spectra confirmed the identity of (glyco)peptides based on the presence of peptide-specific b- and y-ions and the neutral loss of predicted glycans. The amino acid sequences of the (glyco)peptides are shown at the top of the figure. MS/MS spectra of the (glyco)peptides modified with the most elongated glycoform derived from each cell clone are shown. The sequence of peptides, the predicted and measured mass-to-charge ratios, and the charge states are summarized in Table S14.
